# Supplementary figures and images for: Isolation and characterization of a Halomonas species for non-axenic growth-associated production of bio-polyesters from sustainable feedstocks (part 2 of 2)
Source: Appl Environ Microbiol. 2024 Jul 26;90(8):e00603-24. doi: 10.1128/aem.00603-24 (PMC11338360; doi:10.1128/aem.00603-24)

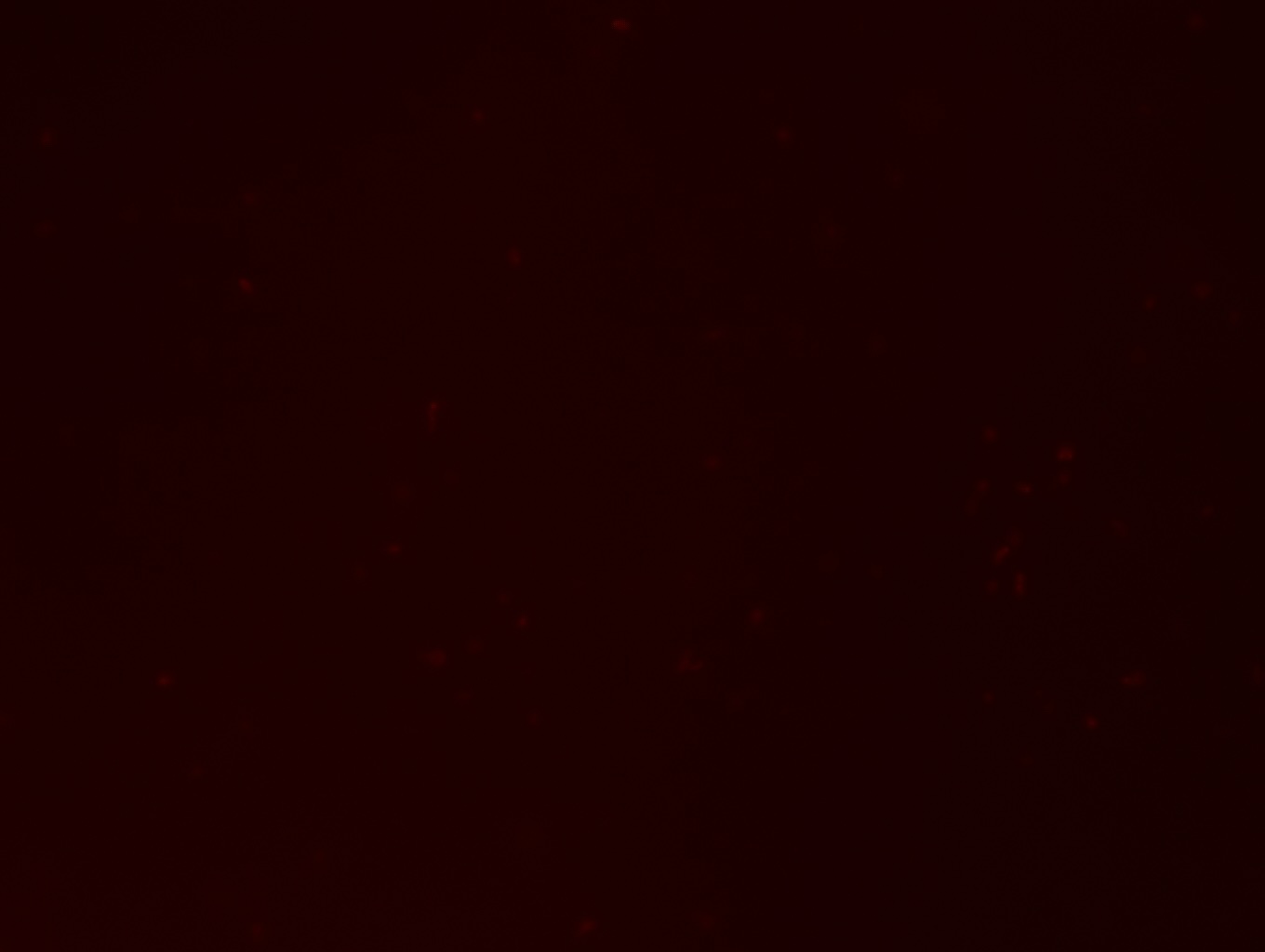

Supplement: File SI2 — Microscopy images of Halomonas sp. CUBES01. [file aem.00603-24-s0002.zip › Microscopy/NB_2nd_0004.jpg]

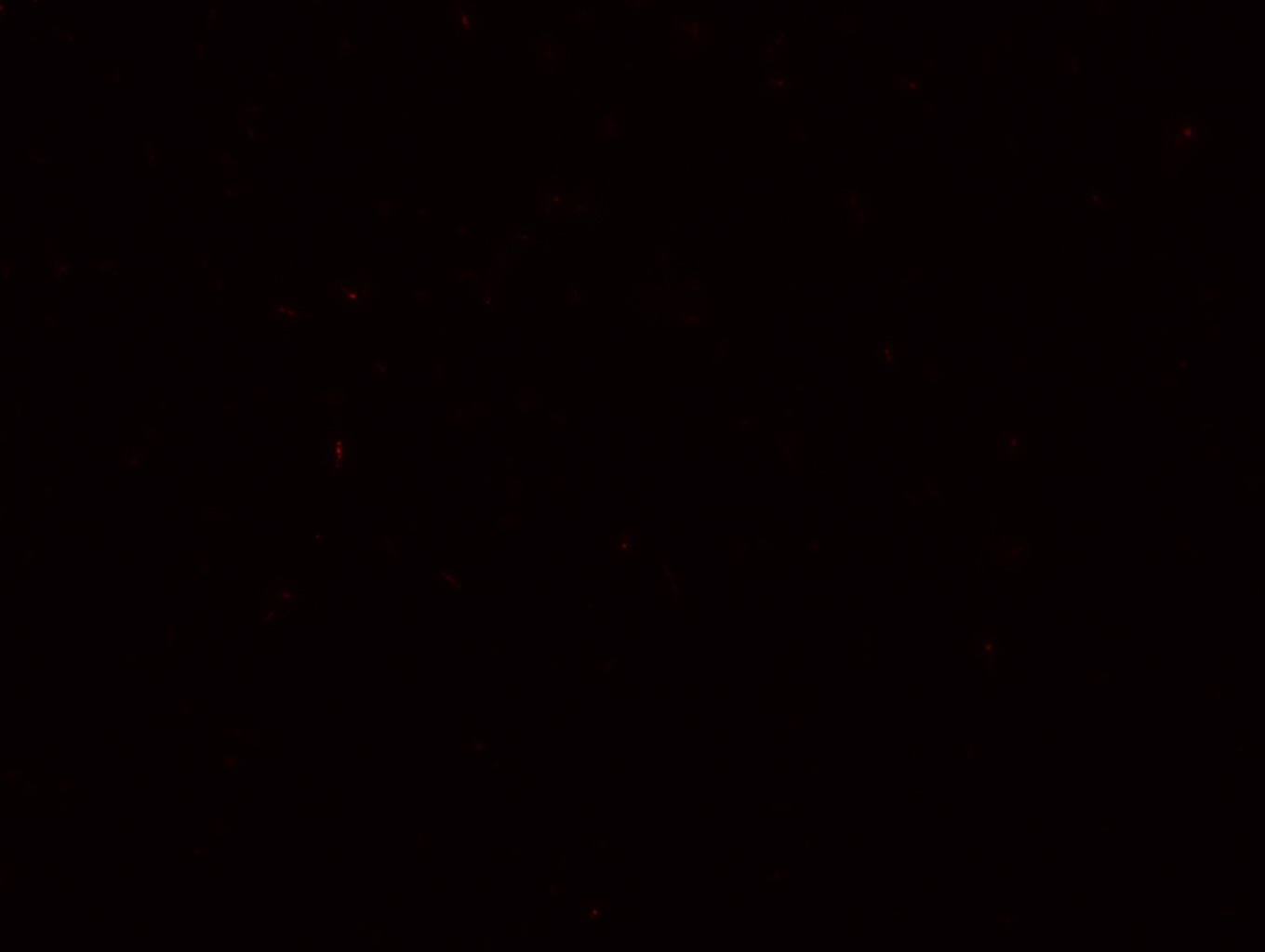

Supplement: File SI2 — Microscopy images of Halomonas sp. CUBES01. [file aem.00603-24-s0002.zip › Microscopy/Sucrose_1st_0001.jpg]

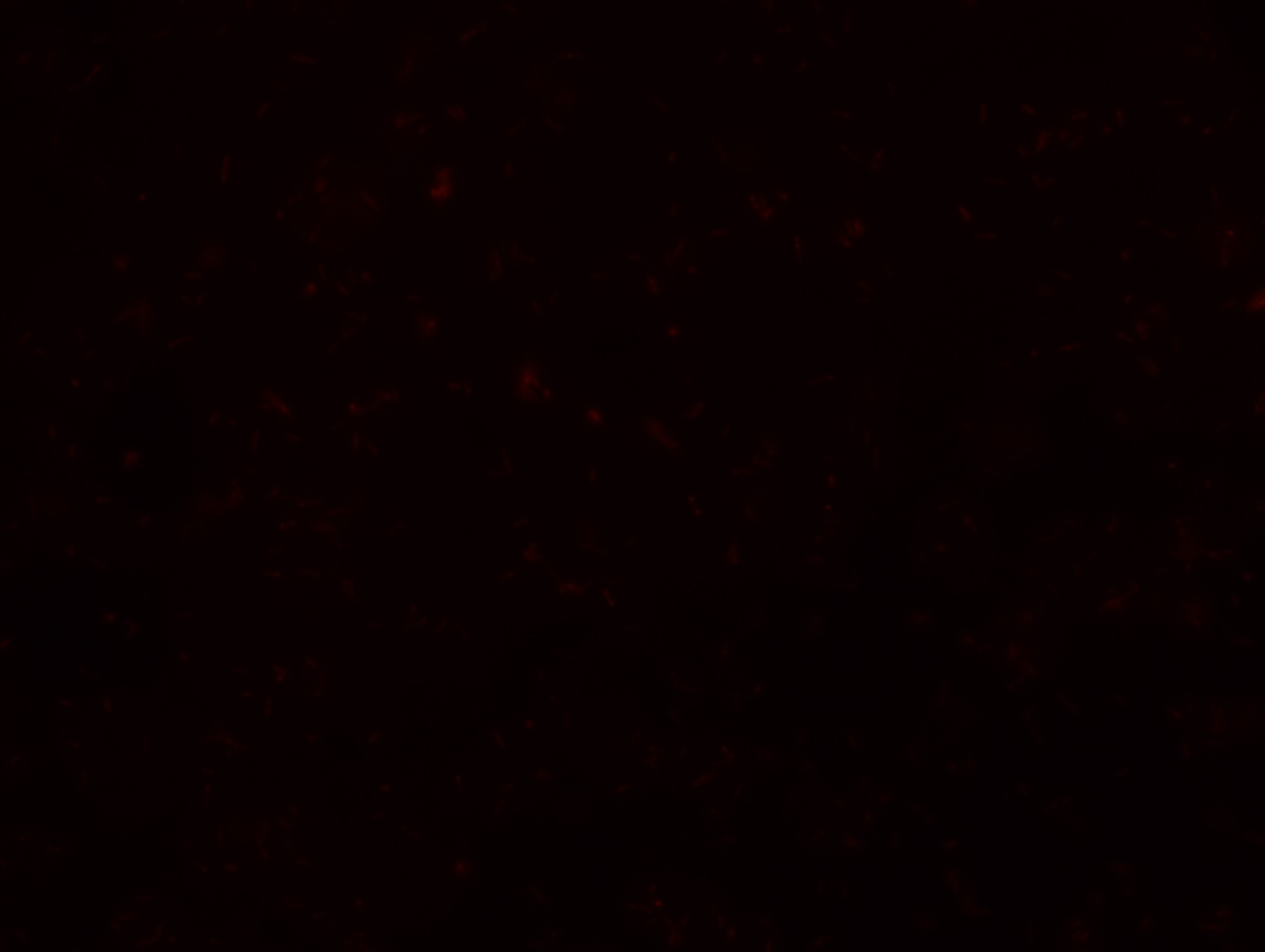

Supplement: File SI2 — Microscopy images of Halomonas sp. CUBES01. [file aem.00603-24-s0002.zip › Microscopy/Propionate_1st_0004.jpg]

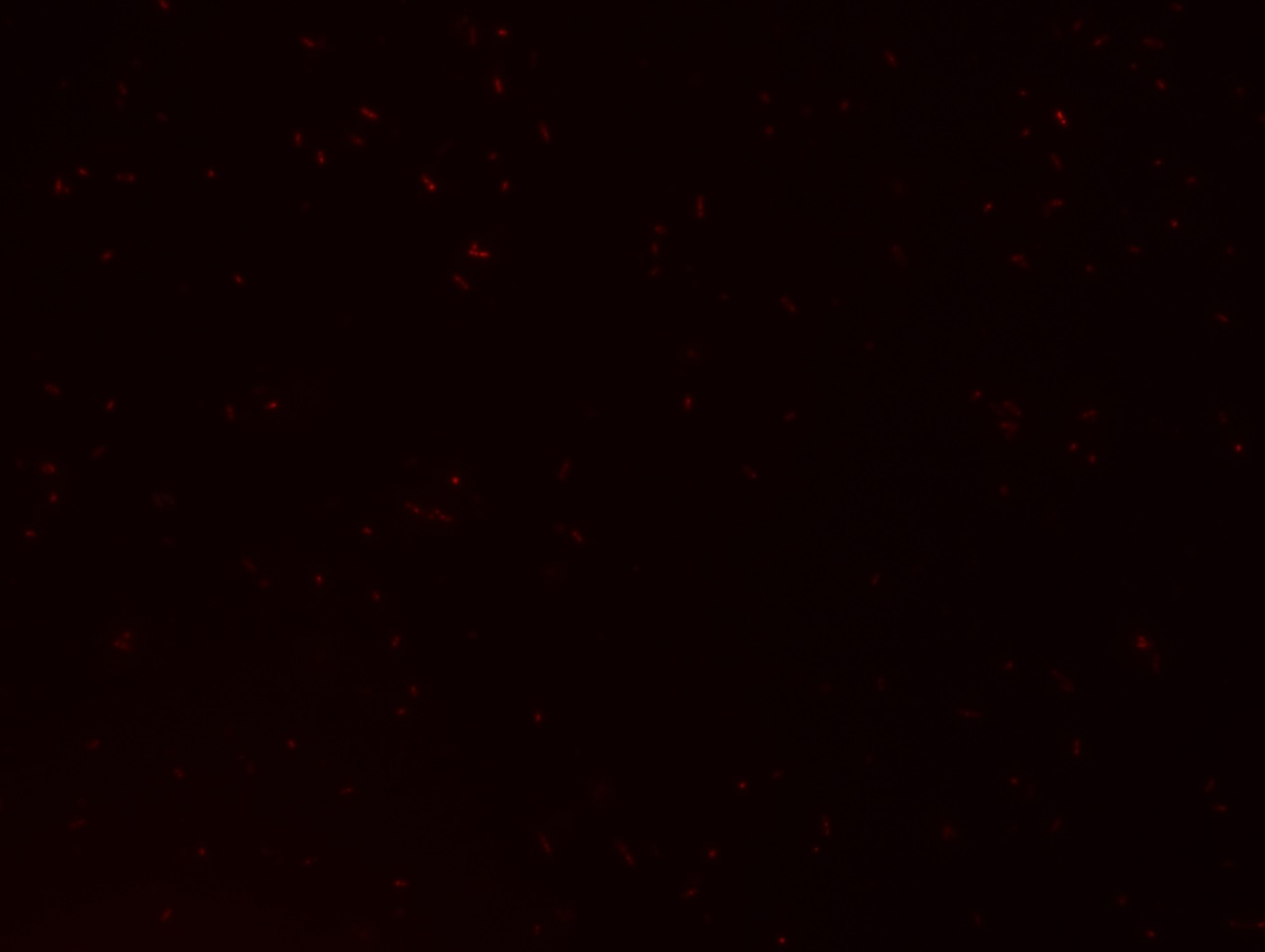

Supplement: File SI2 — Microscopy images of Halomonas sp. CUBES01. [file aem.00603-24-s0002.zip › Microscopy/Acetyl-Glucosamine_1st_0004.jpg]
