# Supplementary figures and images for: Isolation and characterization of a Halomonas species for non-axenic growth-associated production of bio-polyesters from sustainable feedstocks (part 1 of 2)
Source: Appl Environ Microbiol. 2024 Jul 26;90(8):e00603-24. doi: 10.1128/aem.00603-24 (PMC11338360; doi:10.1128/aem.00603-24)

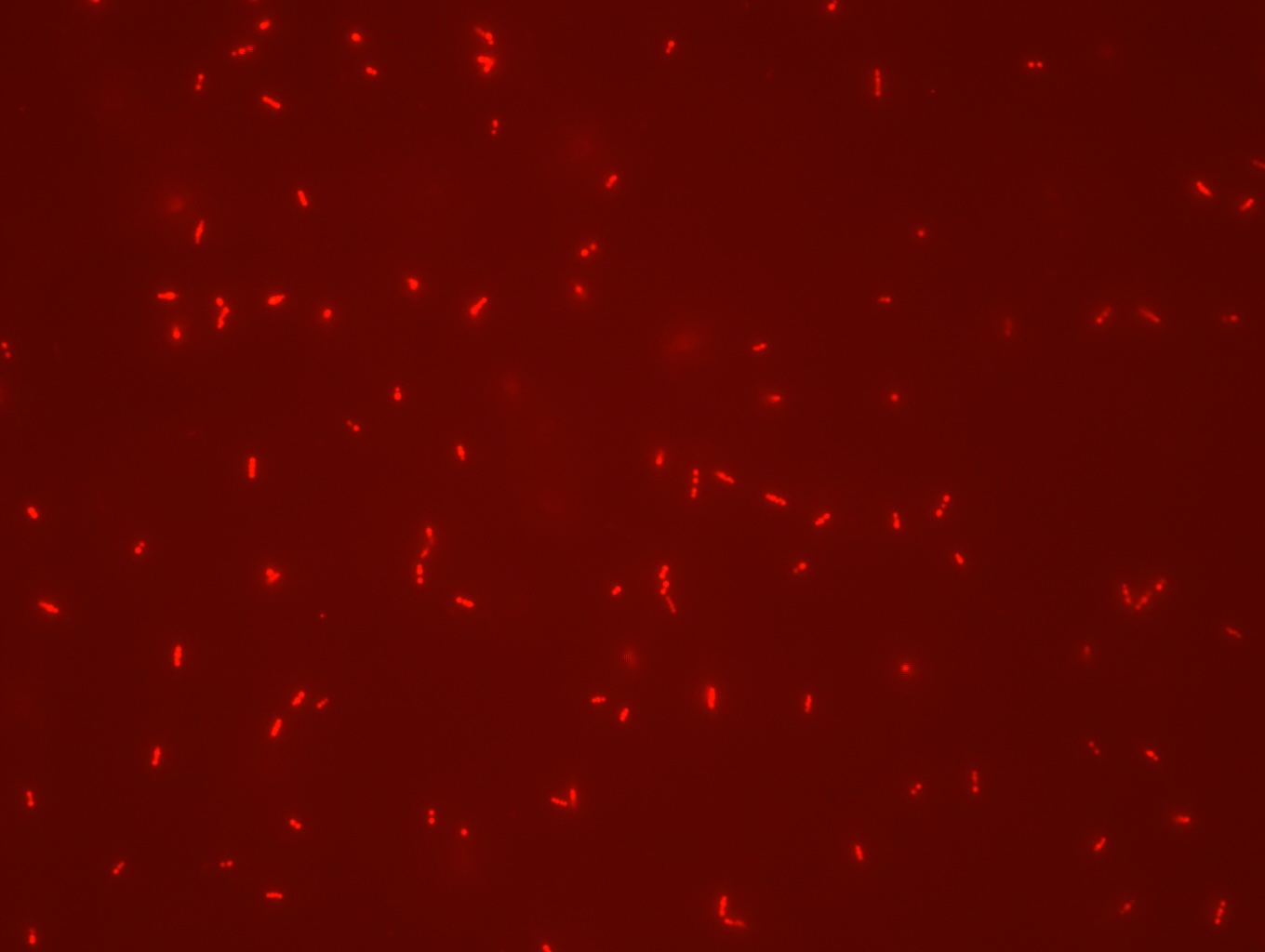

Supplement: File SI2 — Microscopy images of Halomonas sp. CUBES01. [file aem.00603-24-s0002.zip › Microscopy/Sucrose_2nd_0003.jpg]

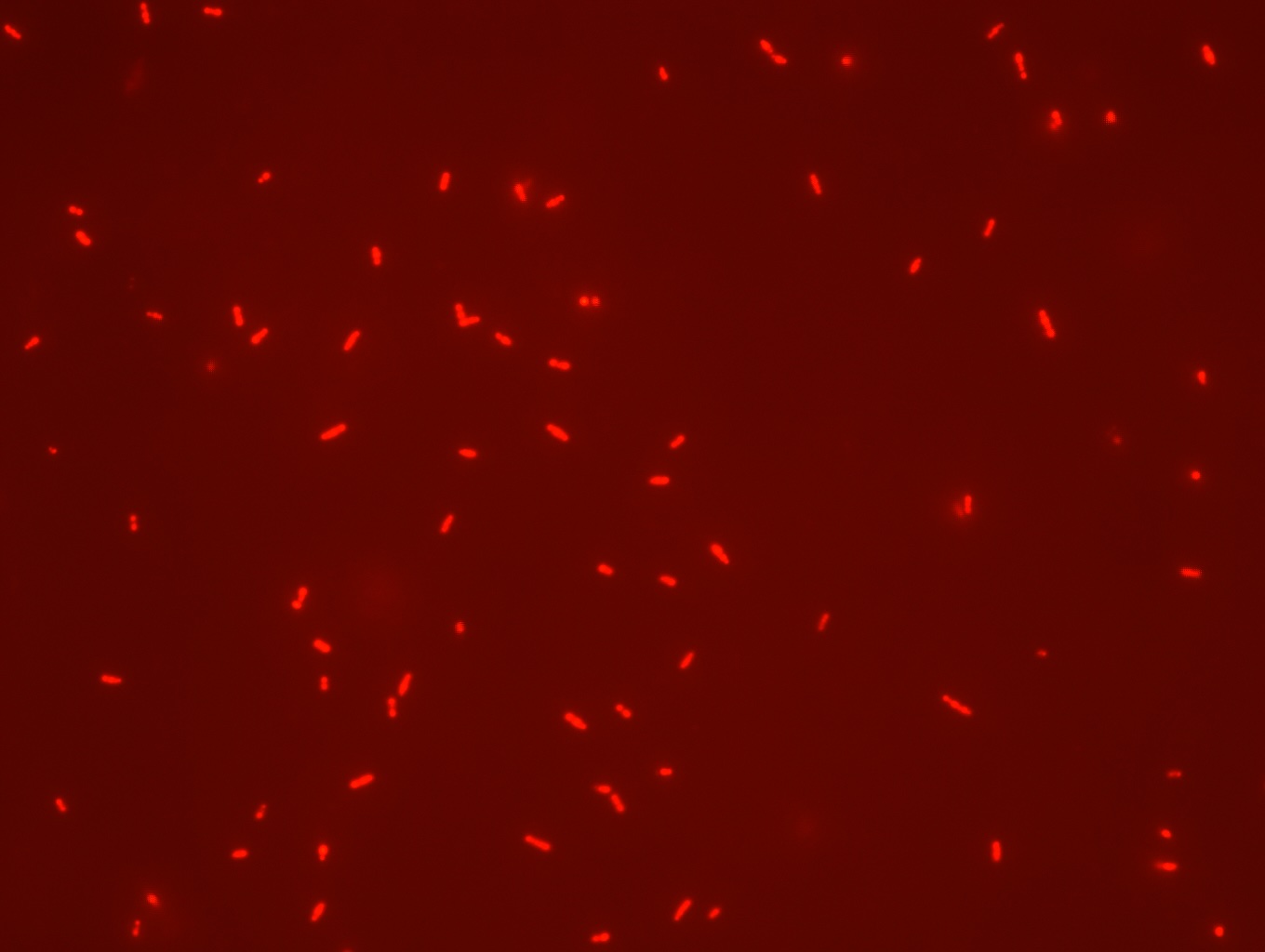

Supplement: File SI2 — Microscopy images of Halomonas sp. CUBES01. [file aem.00603-24-s0002.zip › Microscopy/Sucrose_3rd_0001.jpg]

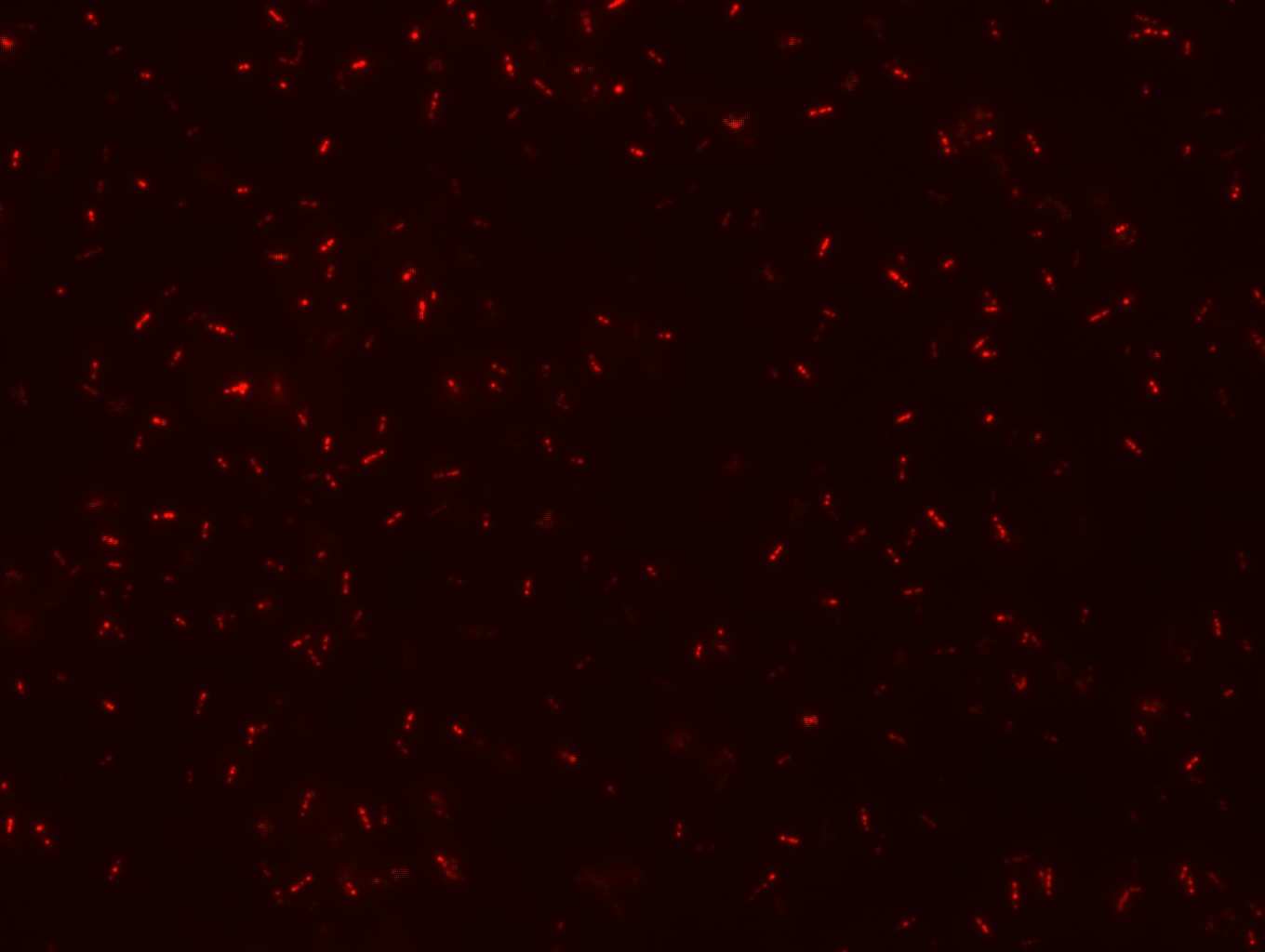

Supplement: File SI2 — Microscopy images of Halomonas sp. CUBES01. [file aem.00603-24-s0002.zip › Microscopy/Propionate_3rd_0004.jpg]

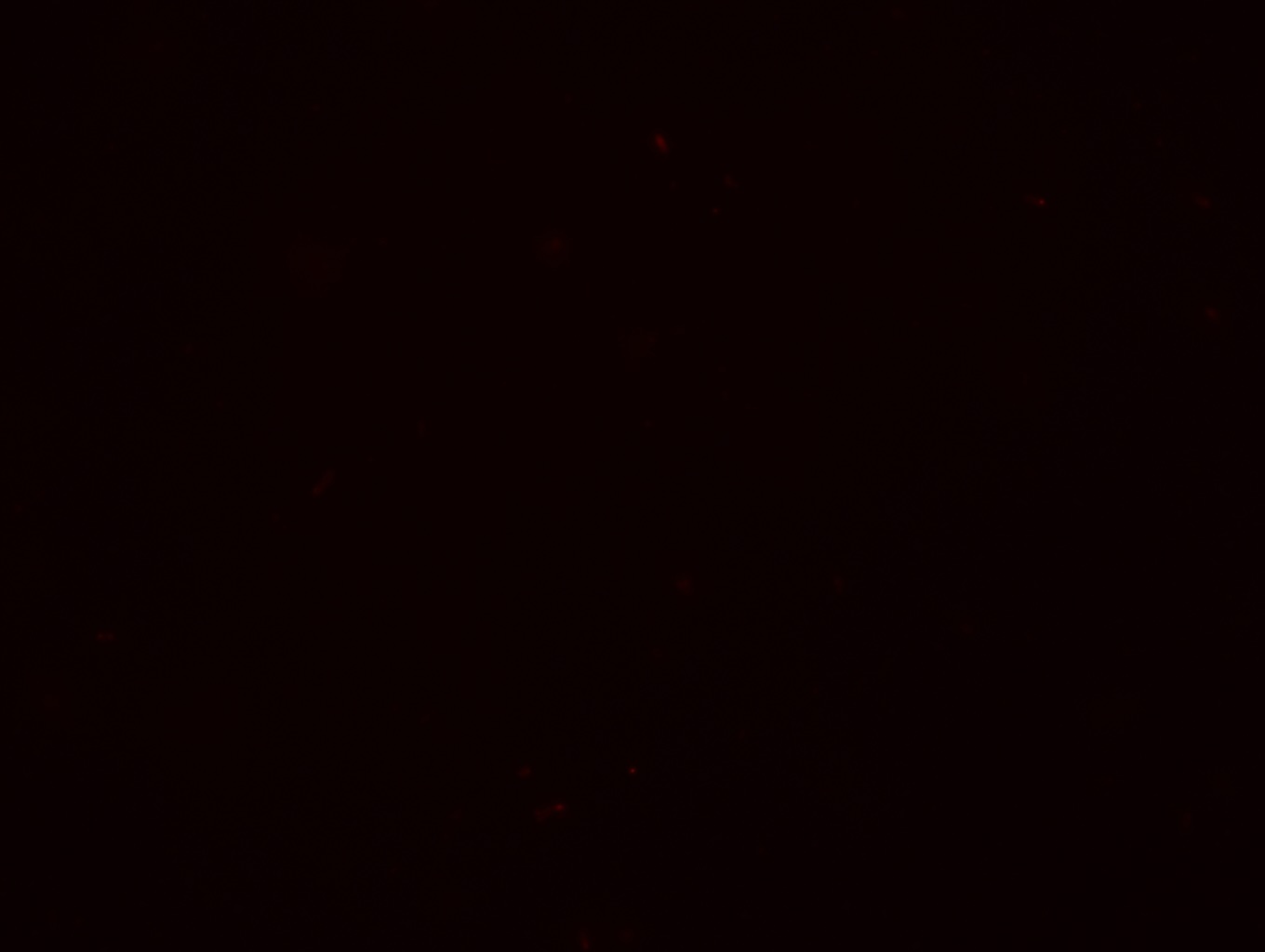

Supplement: File SI2 — Microscopy images of Halomonas sp. CUBES01. [file aem.00603-24-s0002.zip › Microscopy/NB_1st_0004.jpg]

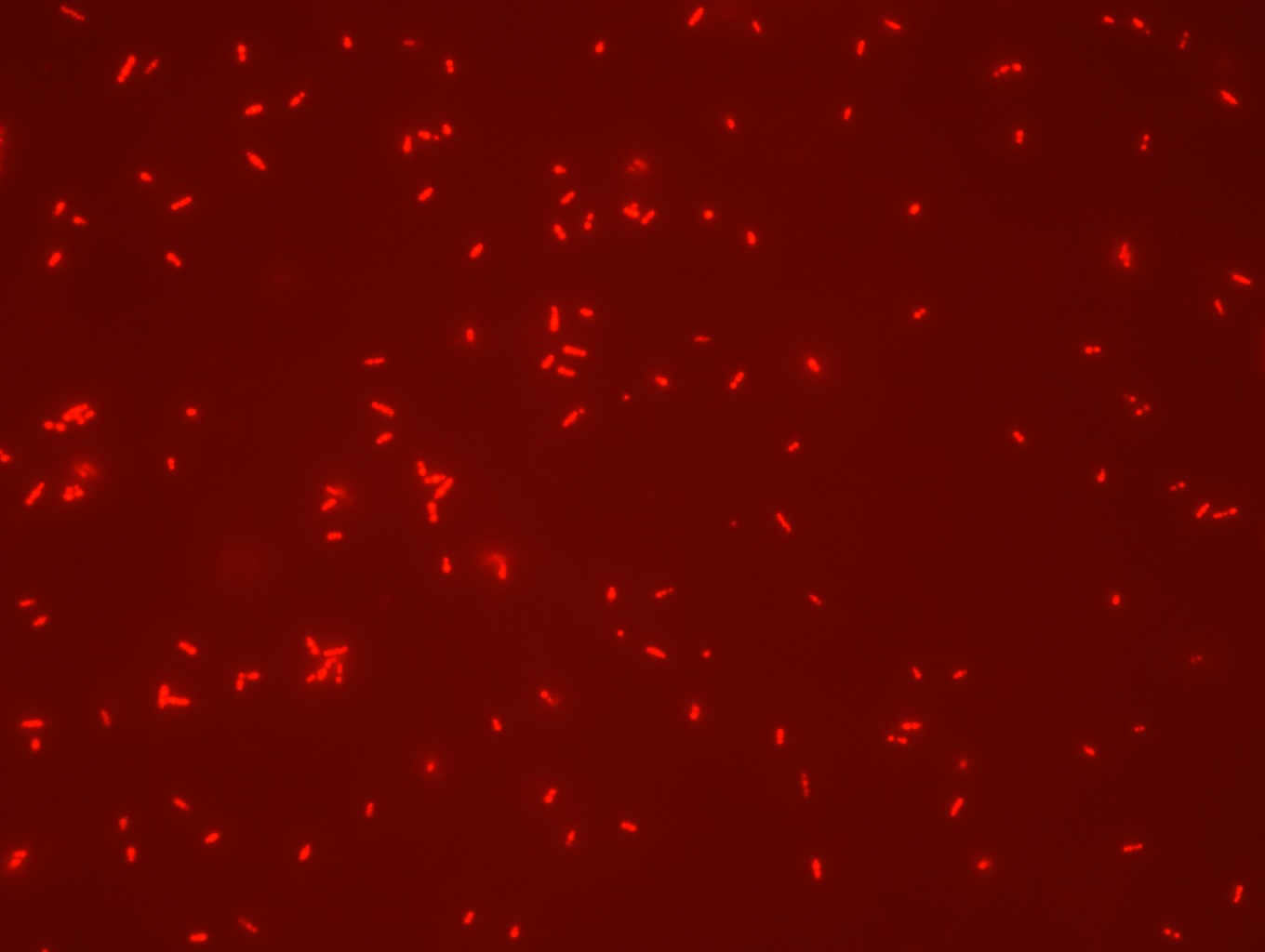

Supplement: File SI2 — Microscopy images of Halomonas sp. CUBES01. [file aem.00603-24-s0002.zip › Microscopy/Sucrose_2nd_0002.jpg]

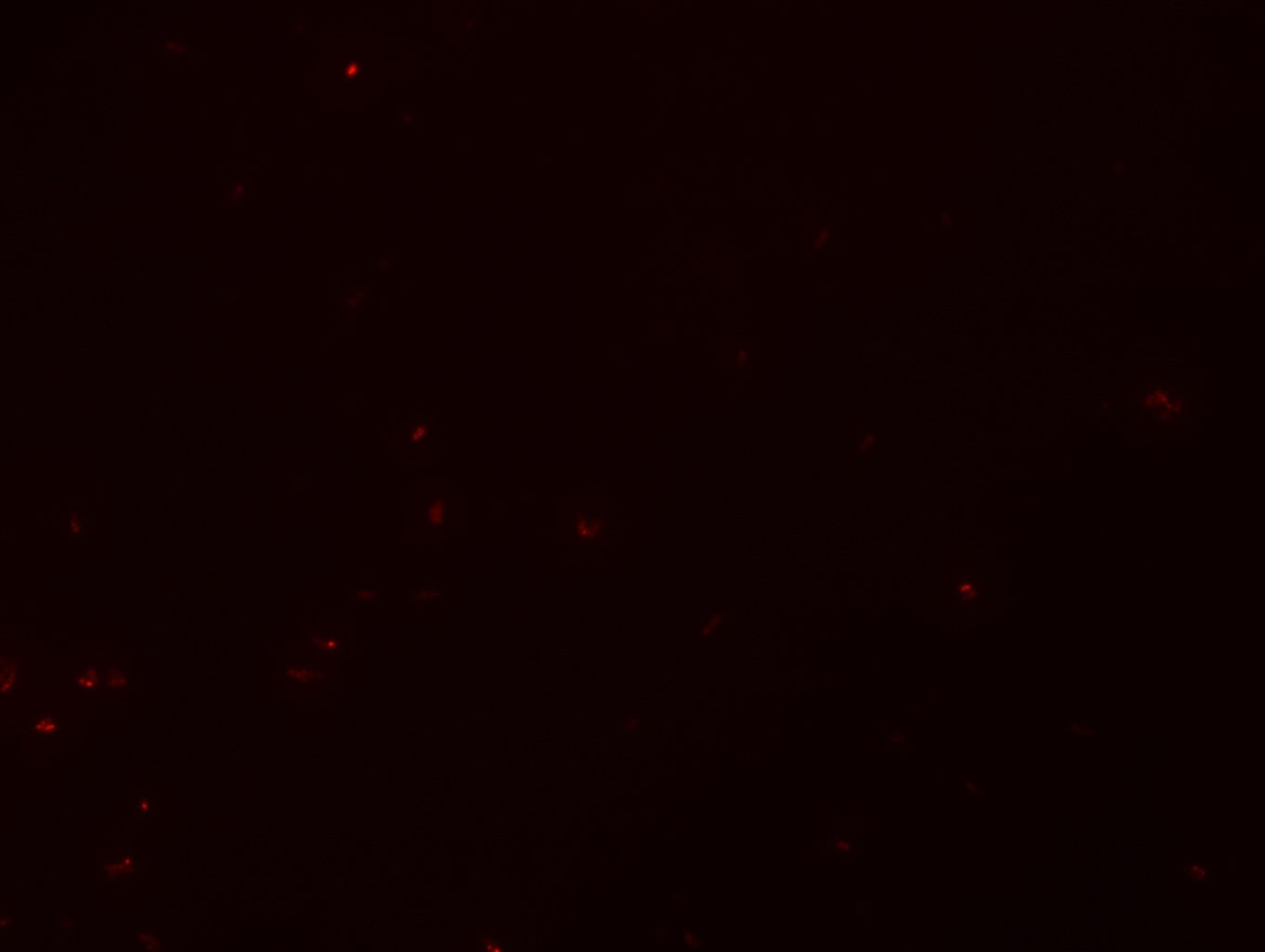

Supplement: File SI2 — Microscopy images of Halomonas sp. CUBES01. [file aem.00603-24-s0002.zip › Microscopy/Acetyl-Glucosamine_3rd_0004.jpg]

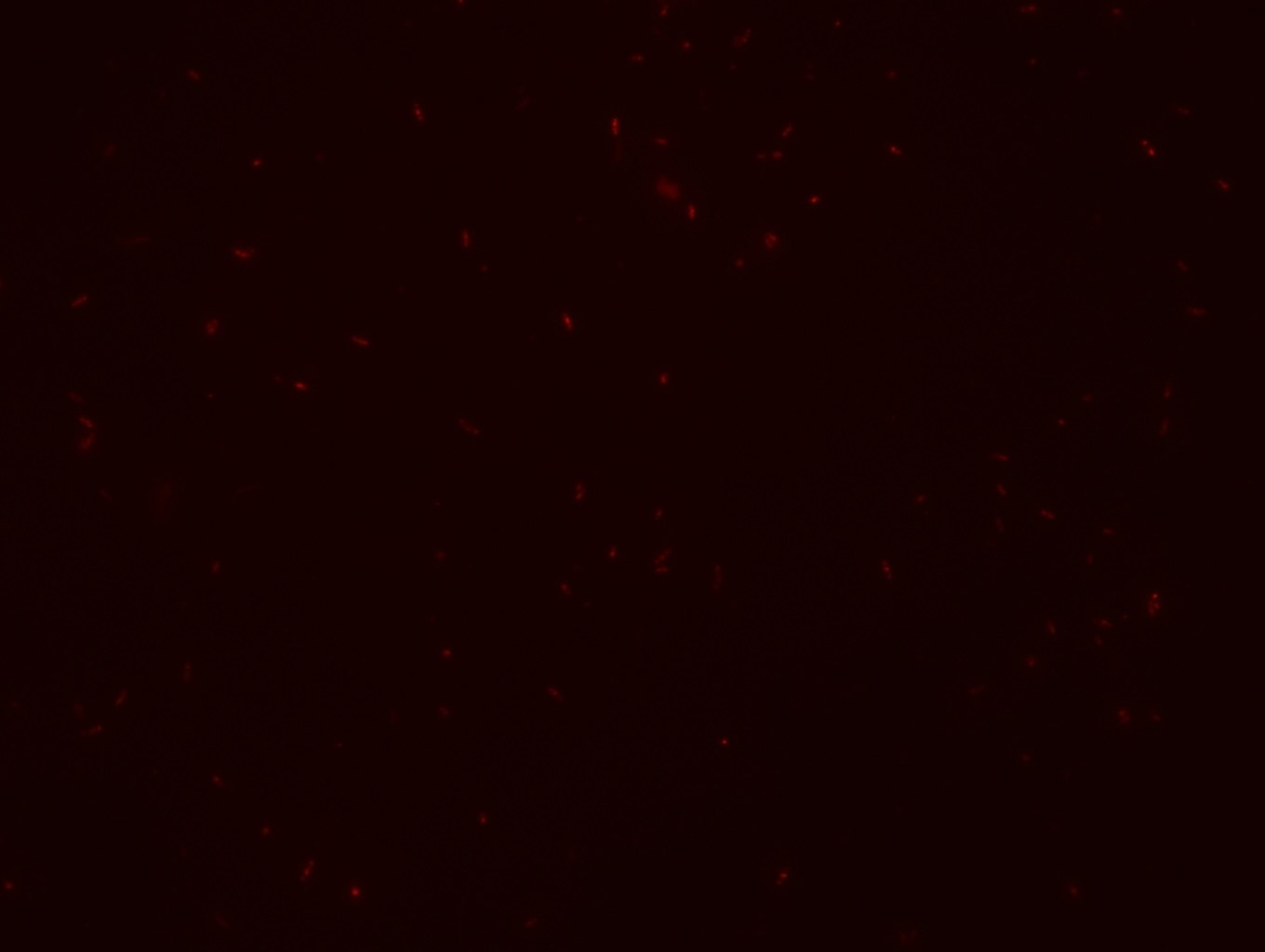

Supplement: File SI2 — Microscopy images of Halomonas sp. CUBES01. [file aem.00603-24-s0002.zip › Microscopy/Acetyl-Glucosamine_2nd_0004.jpg]

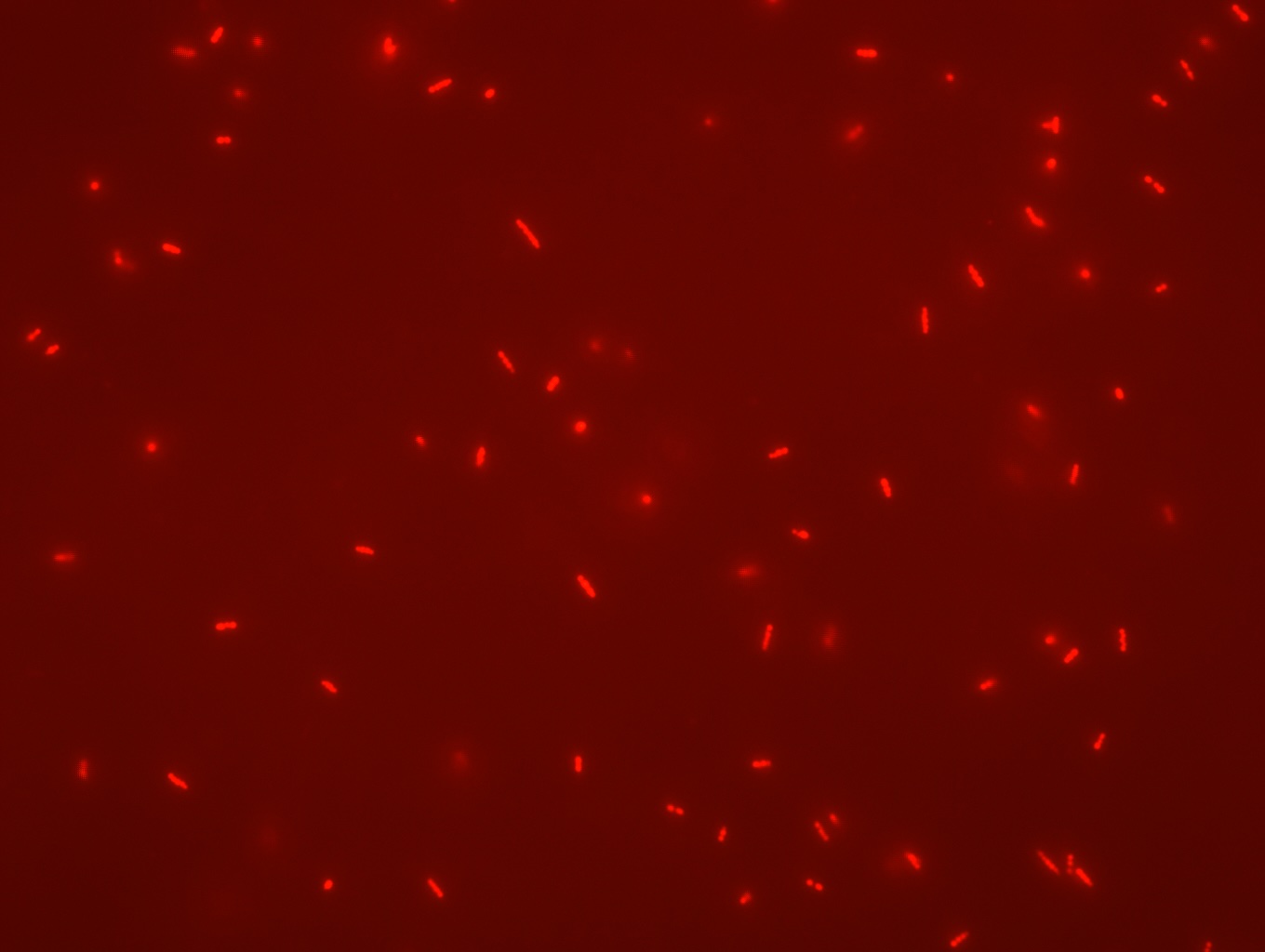

Supplement: File SI2 — Microscopy images of Halomonas sp. CUBES01. [file aem.00603-24-s0002.zip › Microscopy/Sucrose_3rd_0002.jpg]

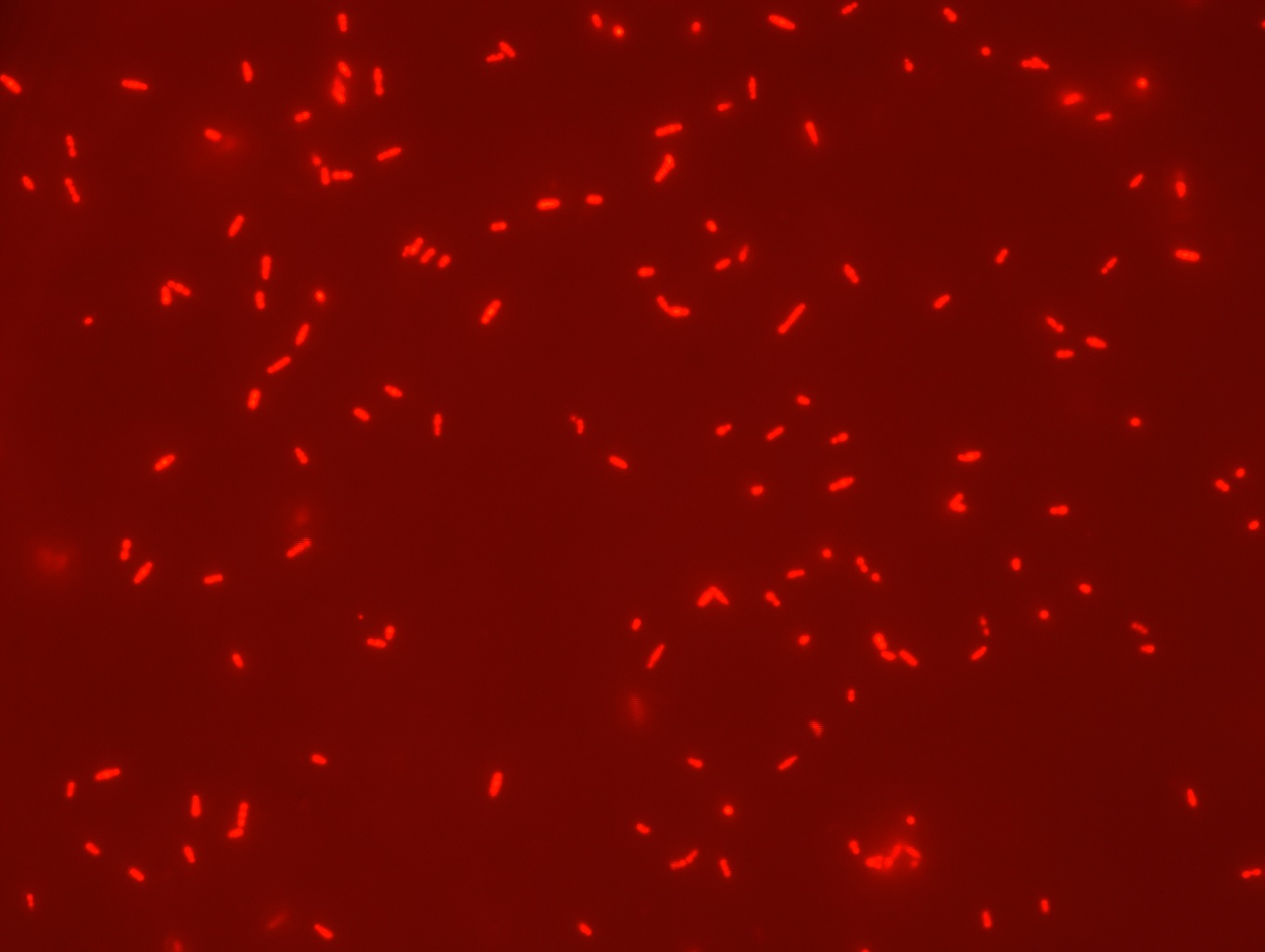

Supplement: File SI2 — Microscopy images of Halomonas sp. CUBES01. [file aem.00603-24-s0002.zip › Microscopy/Sucrose_3rd_0003.jpg]

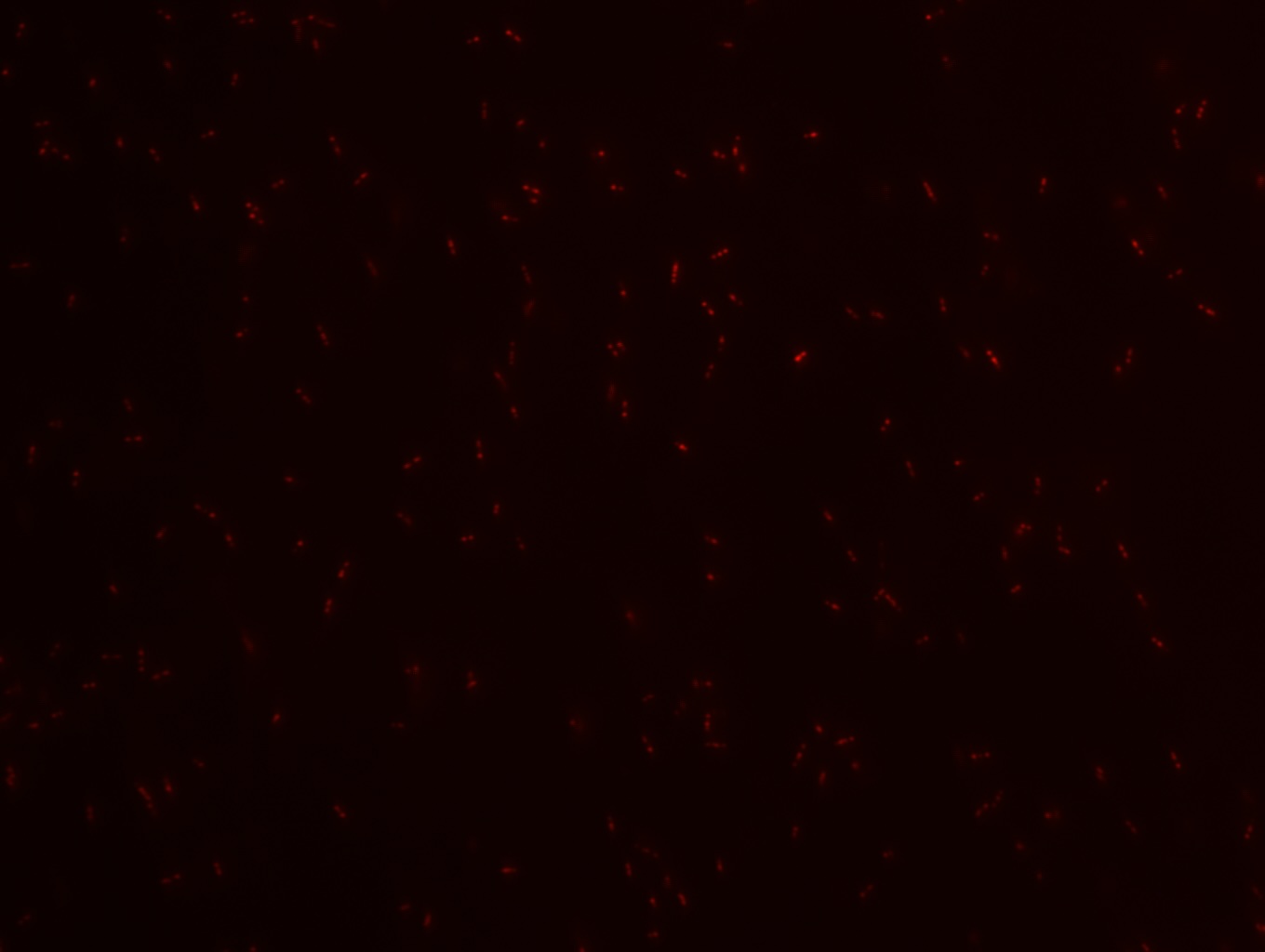

Supplement: File SI2 — Microscopy images of Halomonas sp. CUBES01. [file aem.00603-24-s0002.zip › Microscopy/Propionate_2nd_0004.jpg]

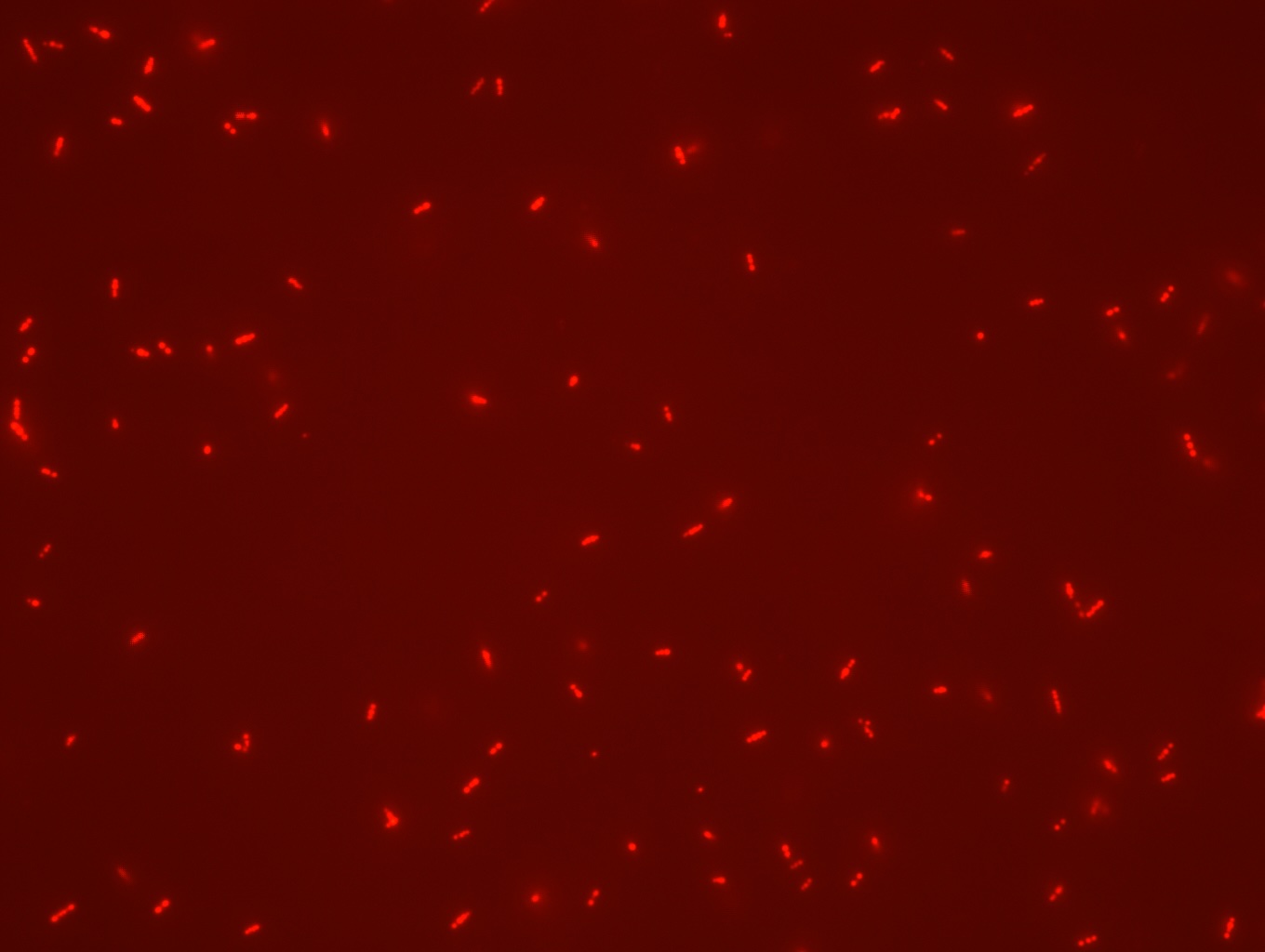

Supplement: File SI2 — Microscopy images of Halomonas sp. CUBES01. [file aem.00603-24-s0002.zip › Microscopy/Sucrose_2nd_0001.jpg]

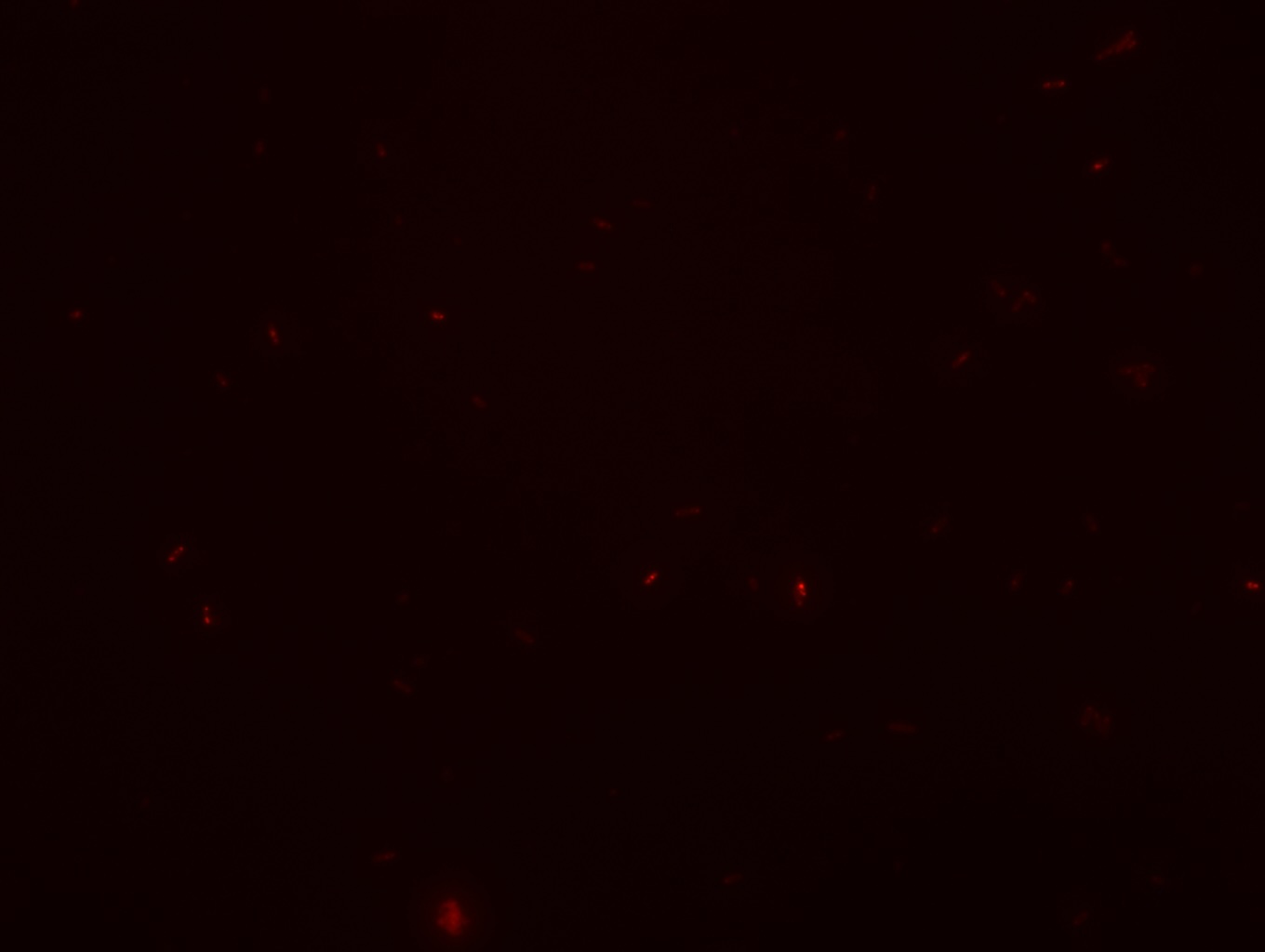

Supplement: File SI2 — Microscopy images of Halomonas sp. CUBES01. [file aem.00603-24-s0002.zip › Microscopy/Acetyl-Glucosamine_3rd_0003.jpg]

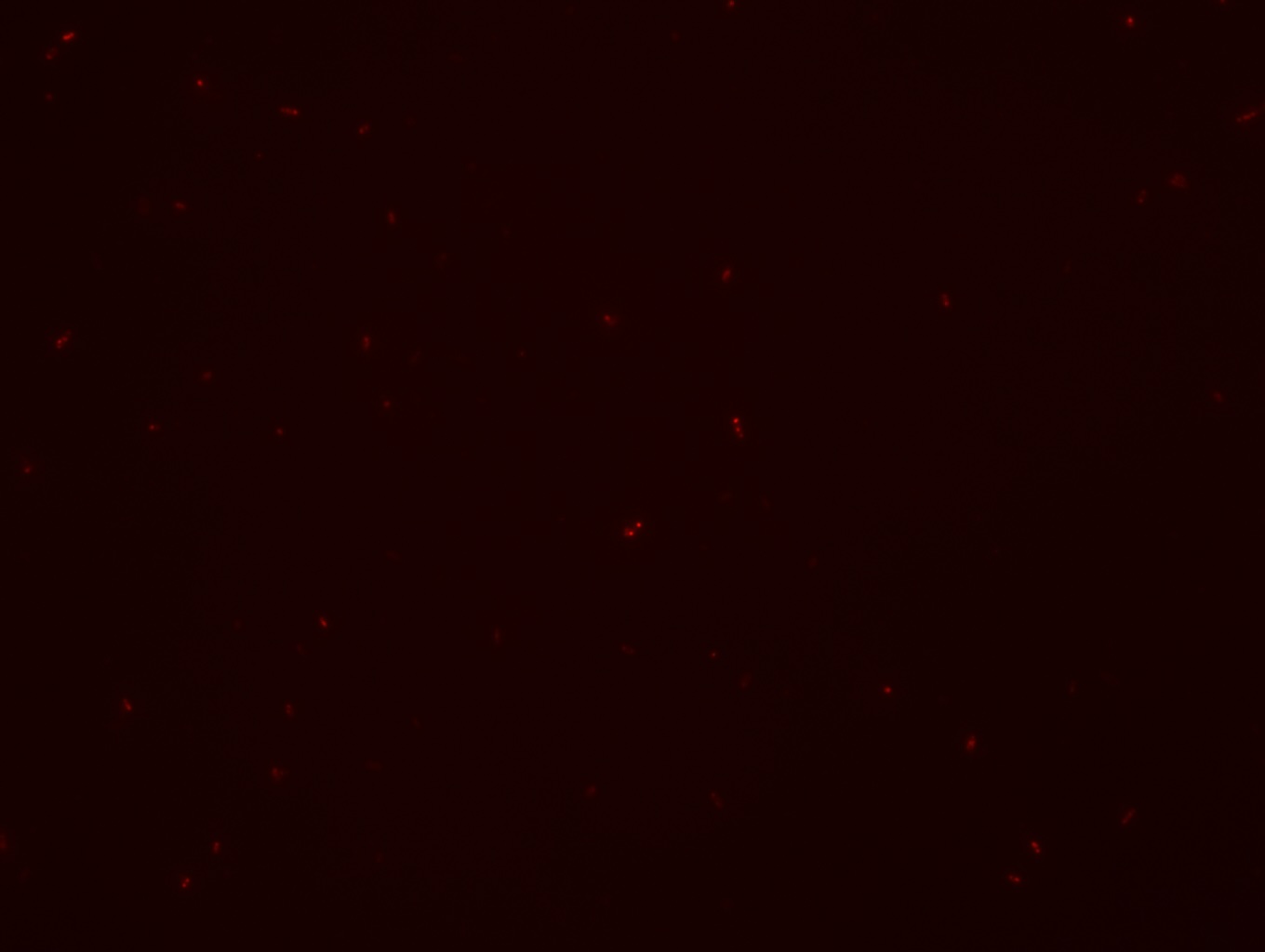

Supplement: File SI2 — Microscopy images of Halomonas sp. CUBES01. [file aem.00603-24-s0002.zip › Microscopy/Acetyl-Glucosamine_2nd_0001.jpg]

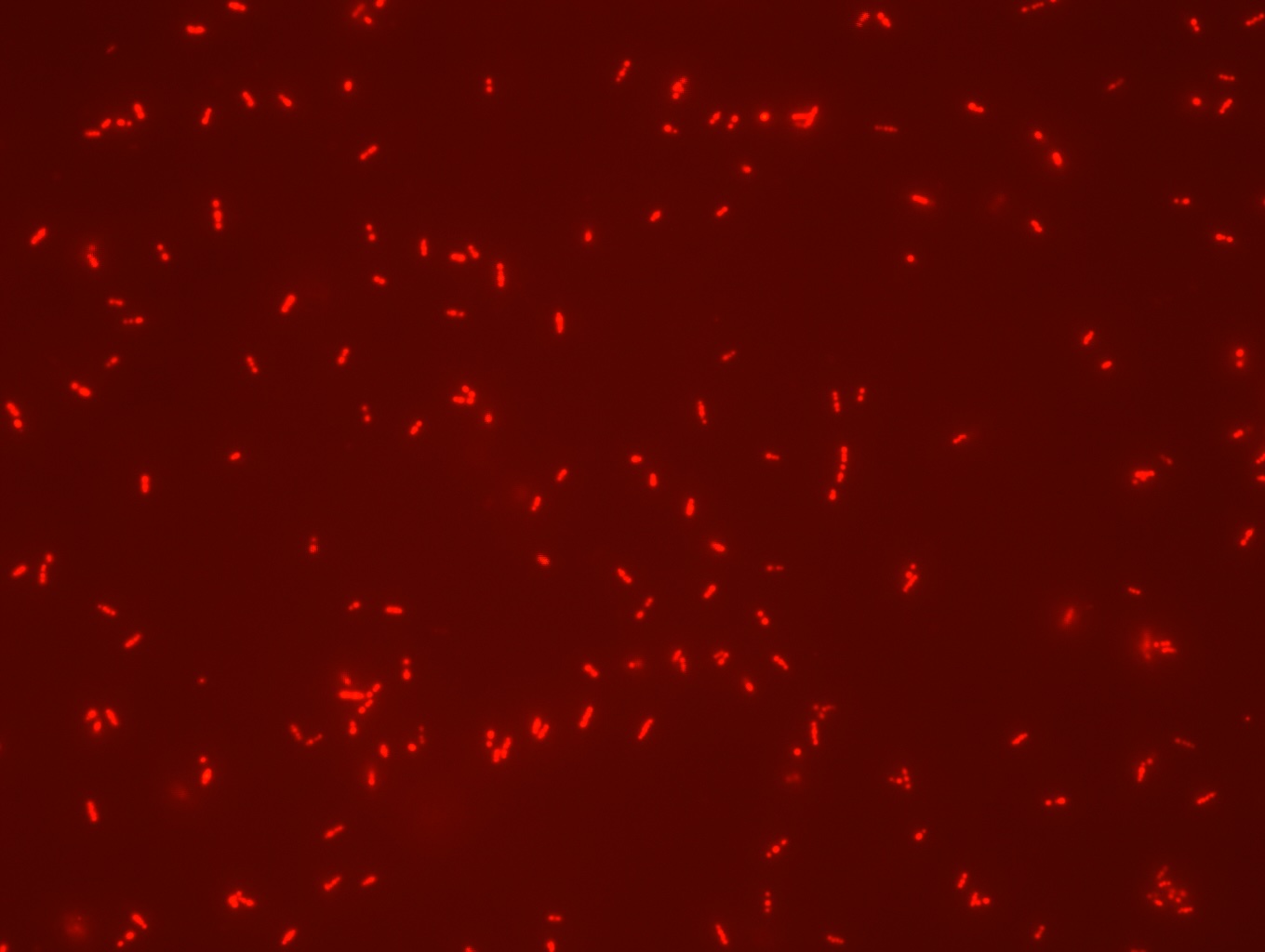

Supplement: File SI2 — Microscopy images of Halomonas sp. CUBES01. [file aem.00603-24-s0002.zip › Microscopy/Sucrose_2nd_0005.jpg]

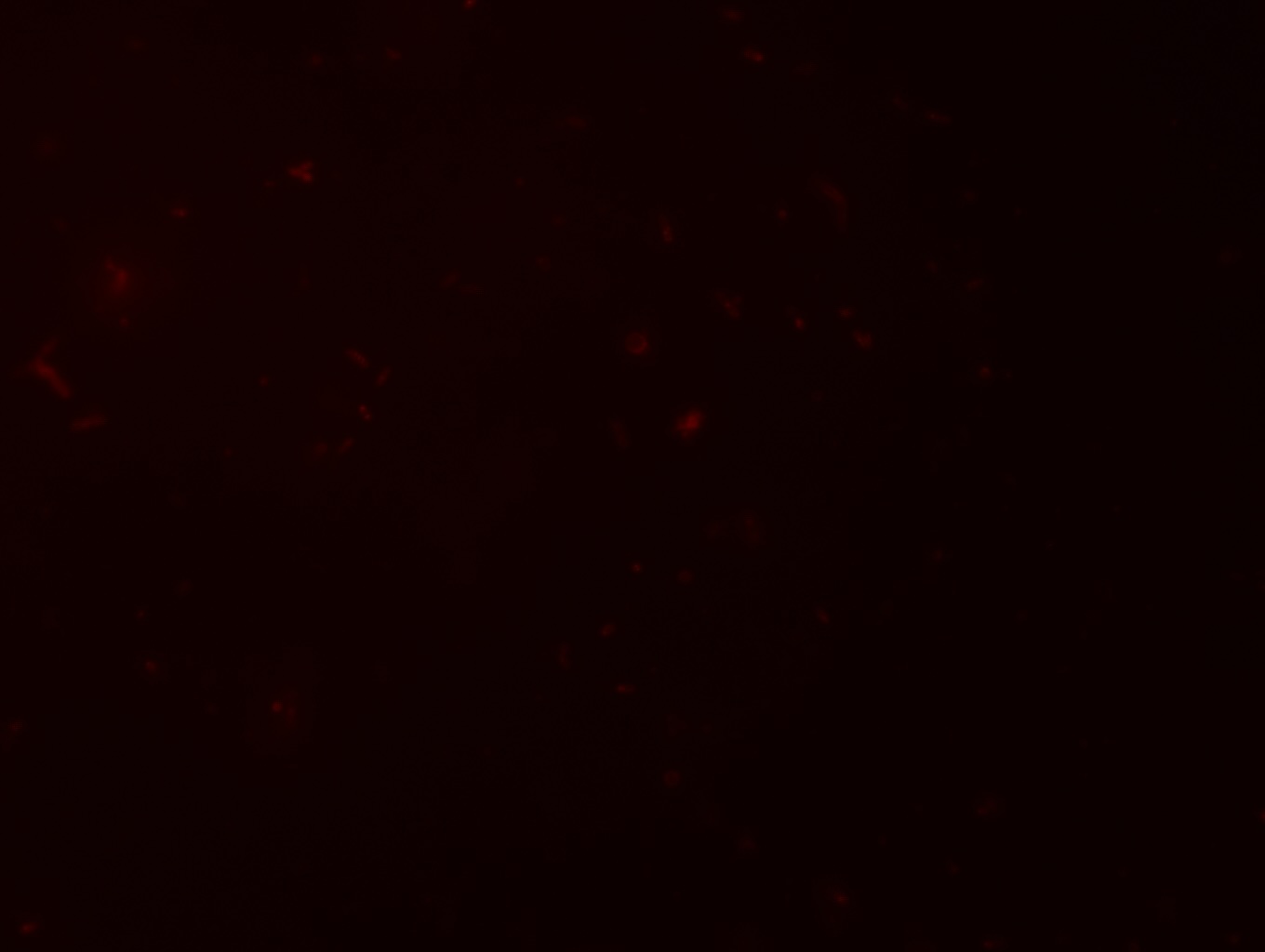

Supplement: File SI2 — Microscopy images of Halomonas sp. CUBES01. [file aem.00603-24-s0002.zip › Microscopy/NB_1st_0002.jpg]

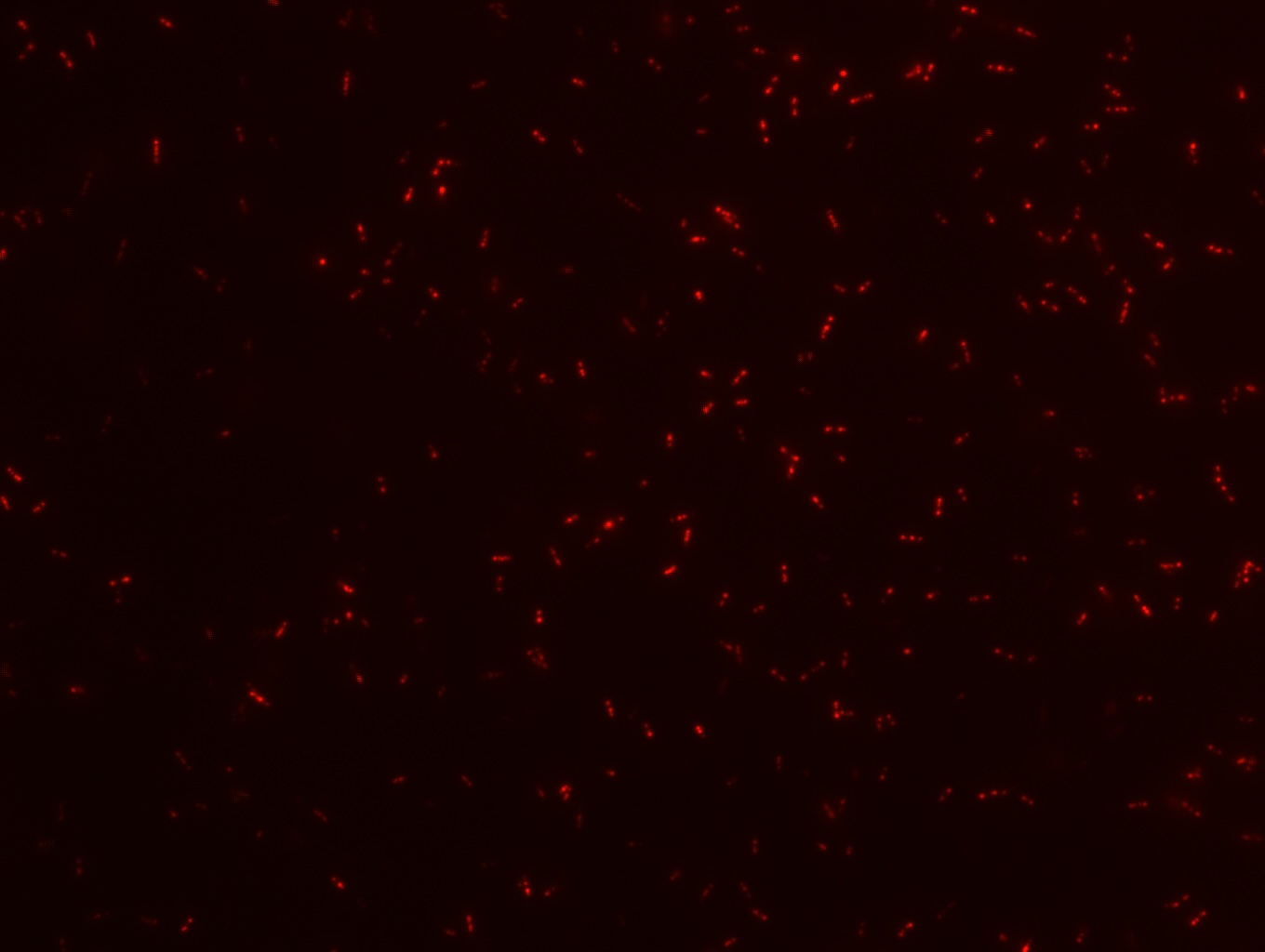

Supplement: File SI2 — Microscopy images of Halomonas sp. CUBES01. [file aem.00603-24-s0002.zip › Microscopy/Propionate_3rd_0002.jpg]

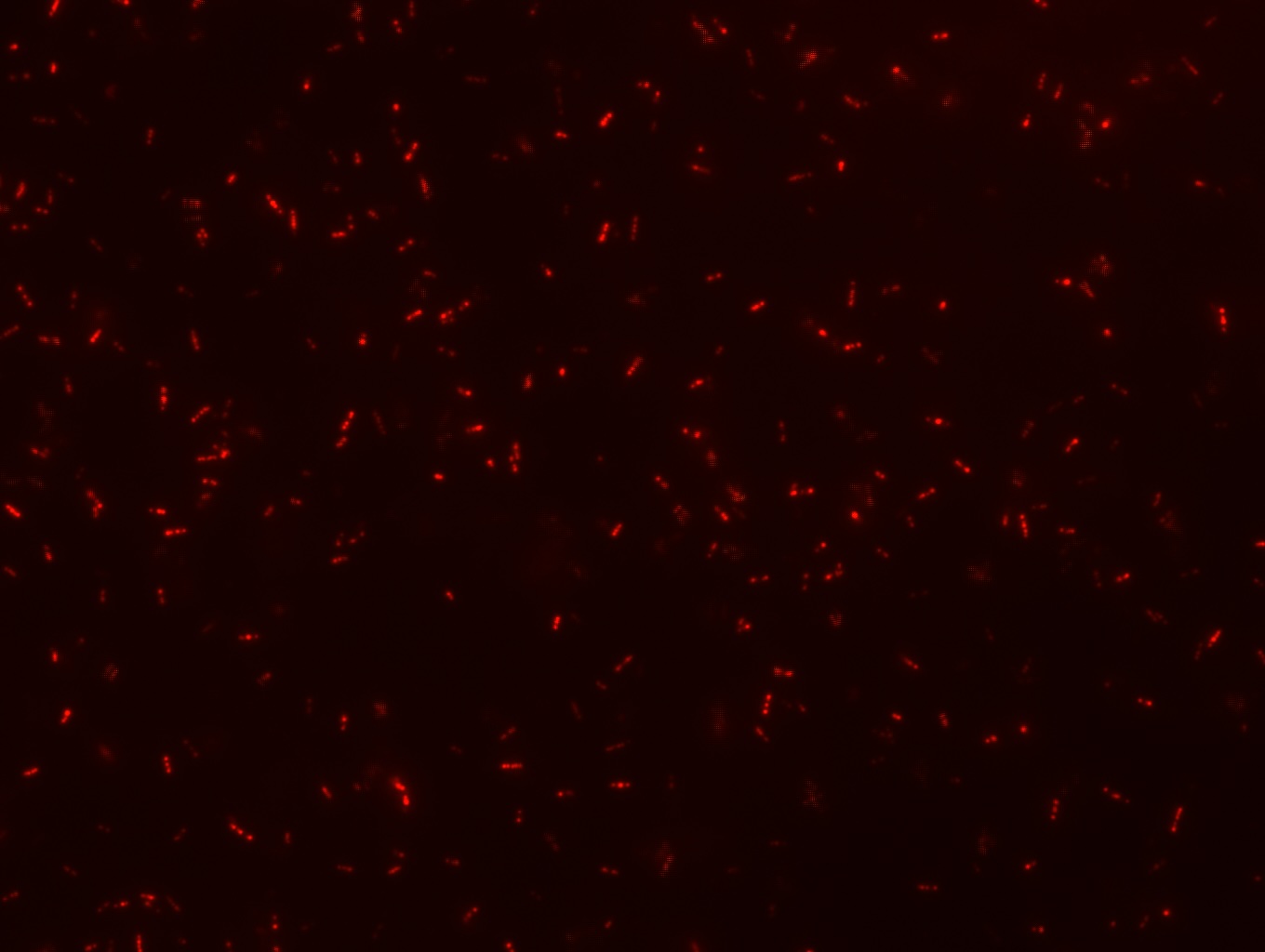

Supplement: File SI2 — Microscopy images of Halomonas sp. CUBES01. [file aem.00603-24-s0002.zip › Microscopy/Propionate_3rd_0003.jpg]

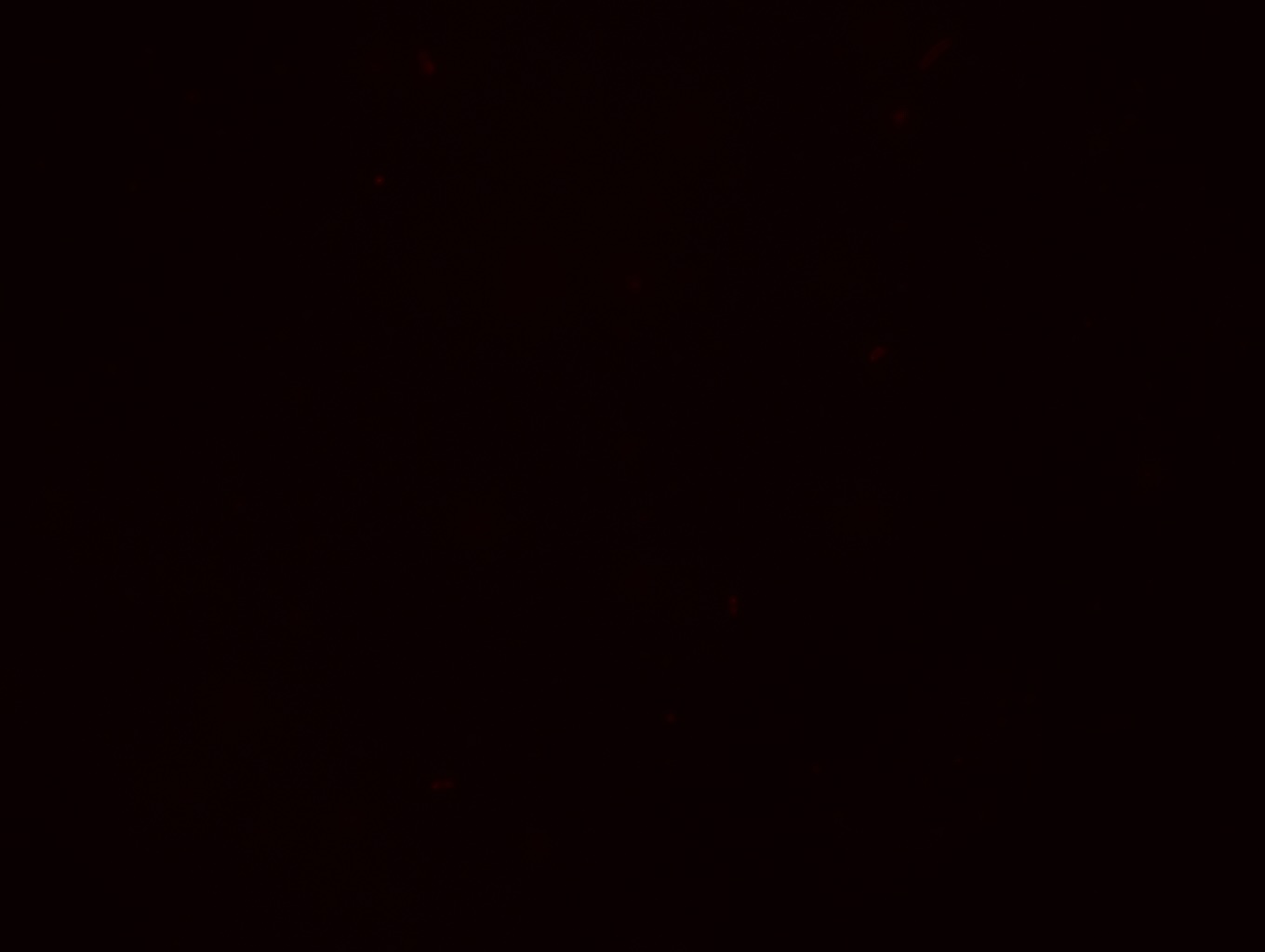

Supplement: File SI2 — Microscopy images of Halomonas sp. CUBES01. [file aem.00603-24-s0002.zip › Microscopy/NB_1st_0003.jpg]

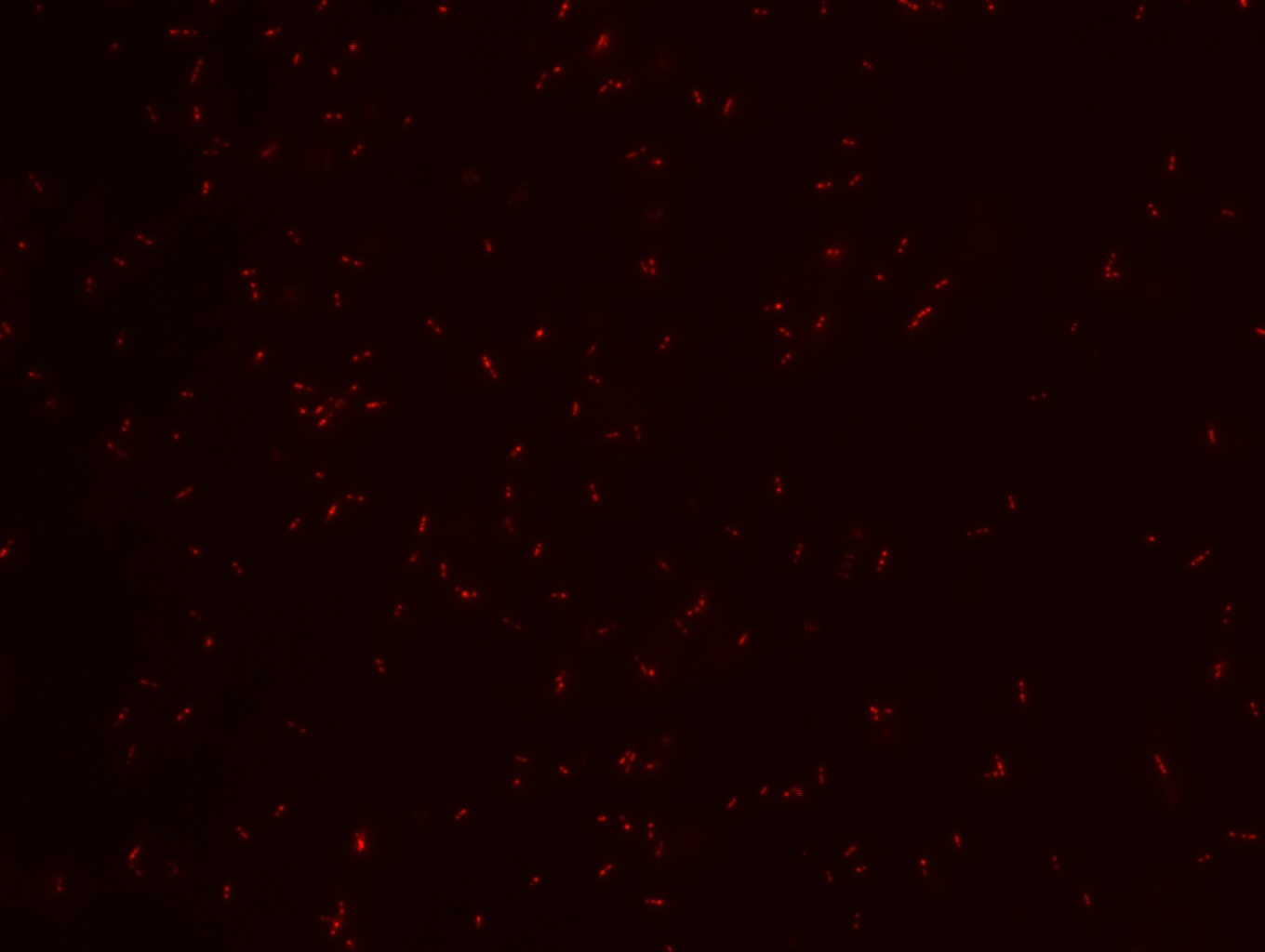

Supplement: File SI2 — Microscopy images of Halomonas sp. CUBES01. [file aem.00603-24-s0002.zip › Microscopy/Propionate_2nd_0001.jpg]

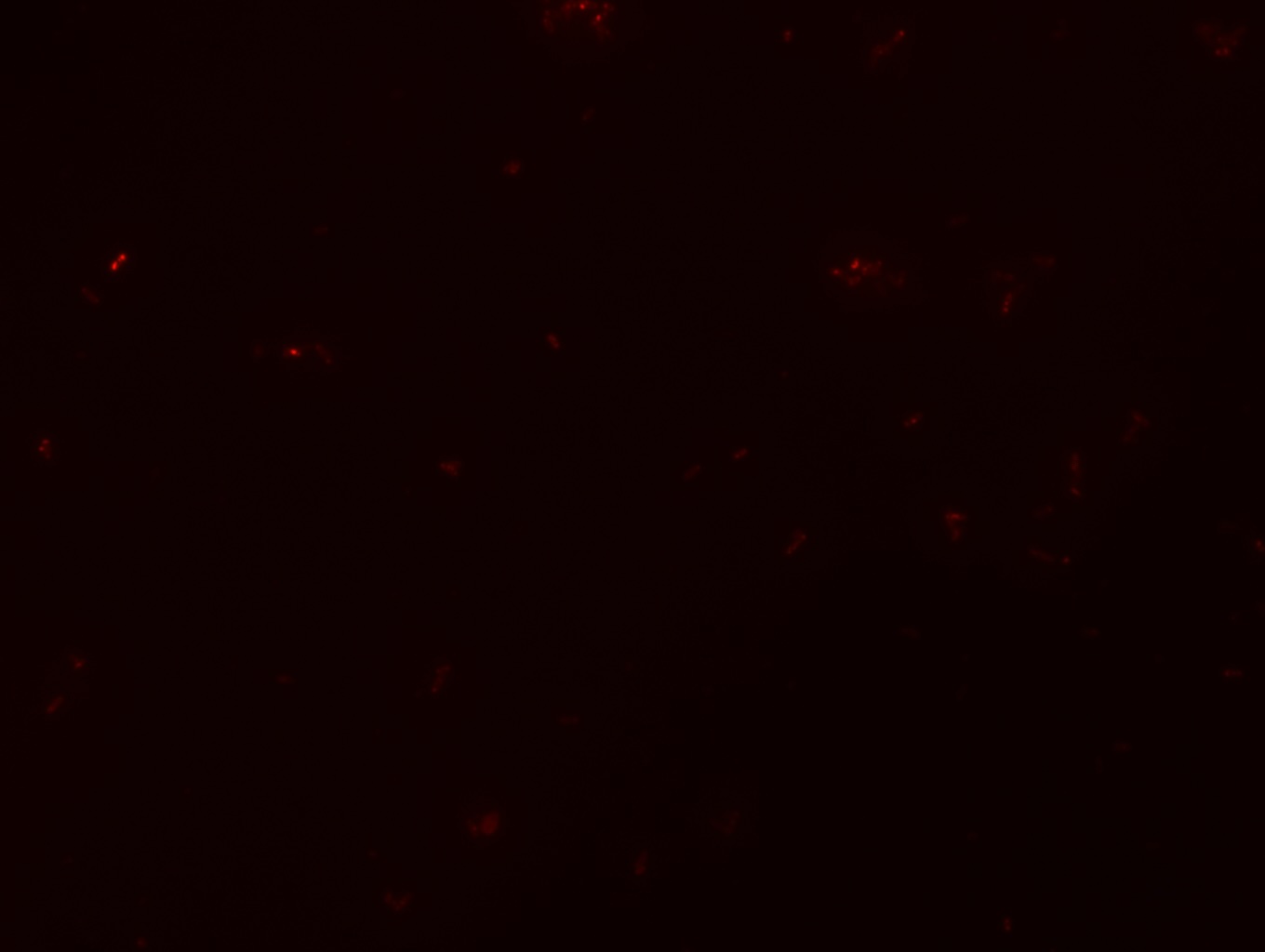

Supplement: File SI2 — Microscopy images of Halomonas sp. CUBES01. [file aem.00603-24-s0002.zip › Microscopy/Acetyl-Glucosamine_3rd_0002.jpg]

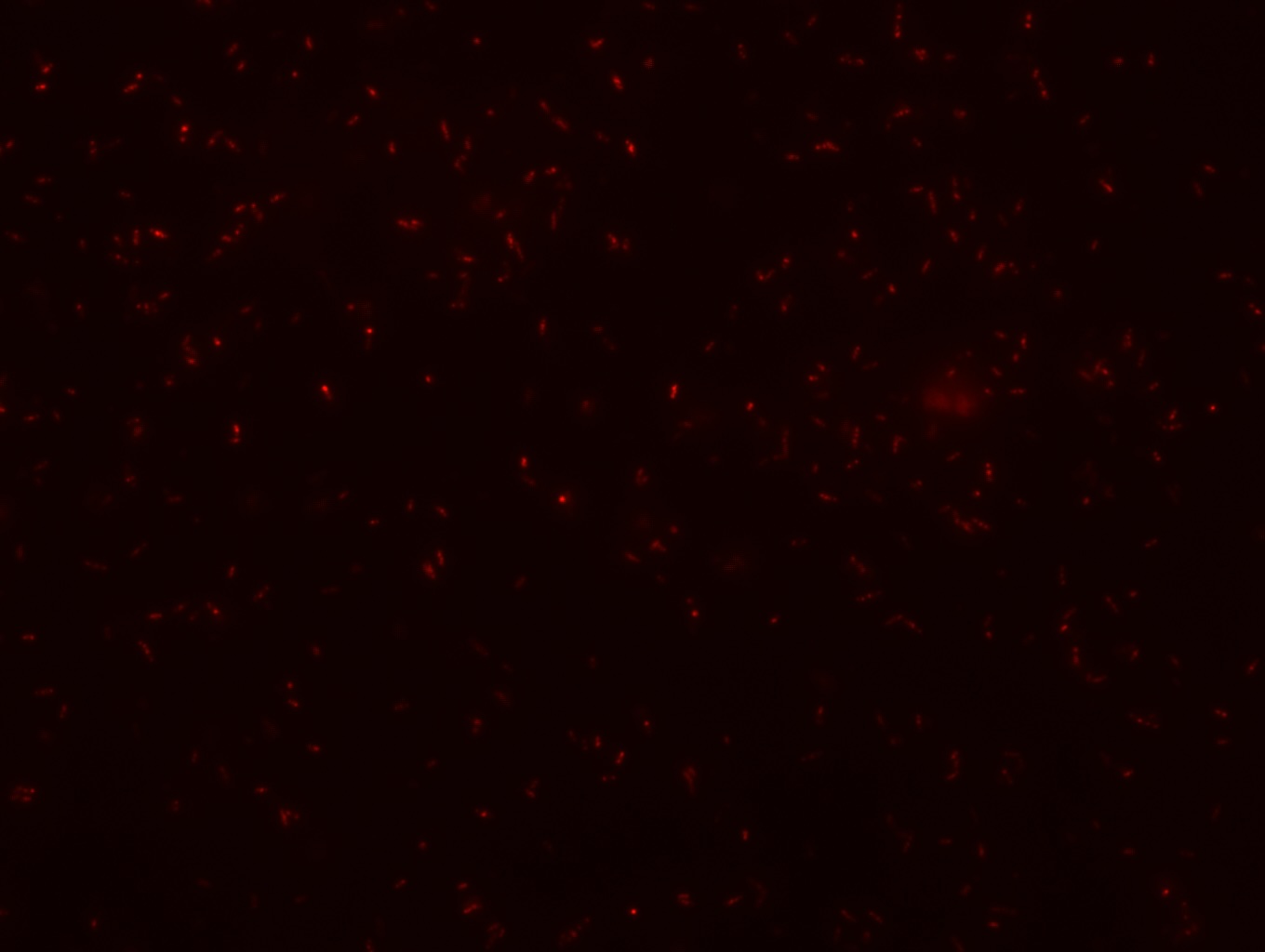

Supplement: File SI2 — Microscopy images of Halomonas sp. CUBES01. [file aem.00603-24-s0002.zip › Microscopy/Acetyl-Glucosamine_2nd_0002.jpg]

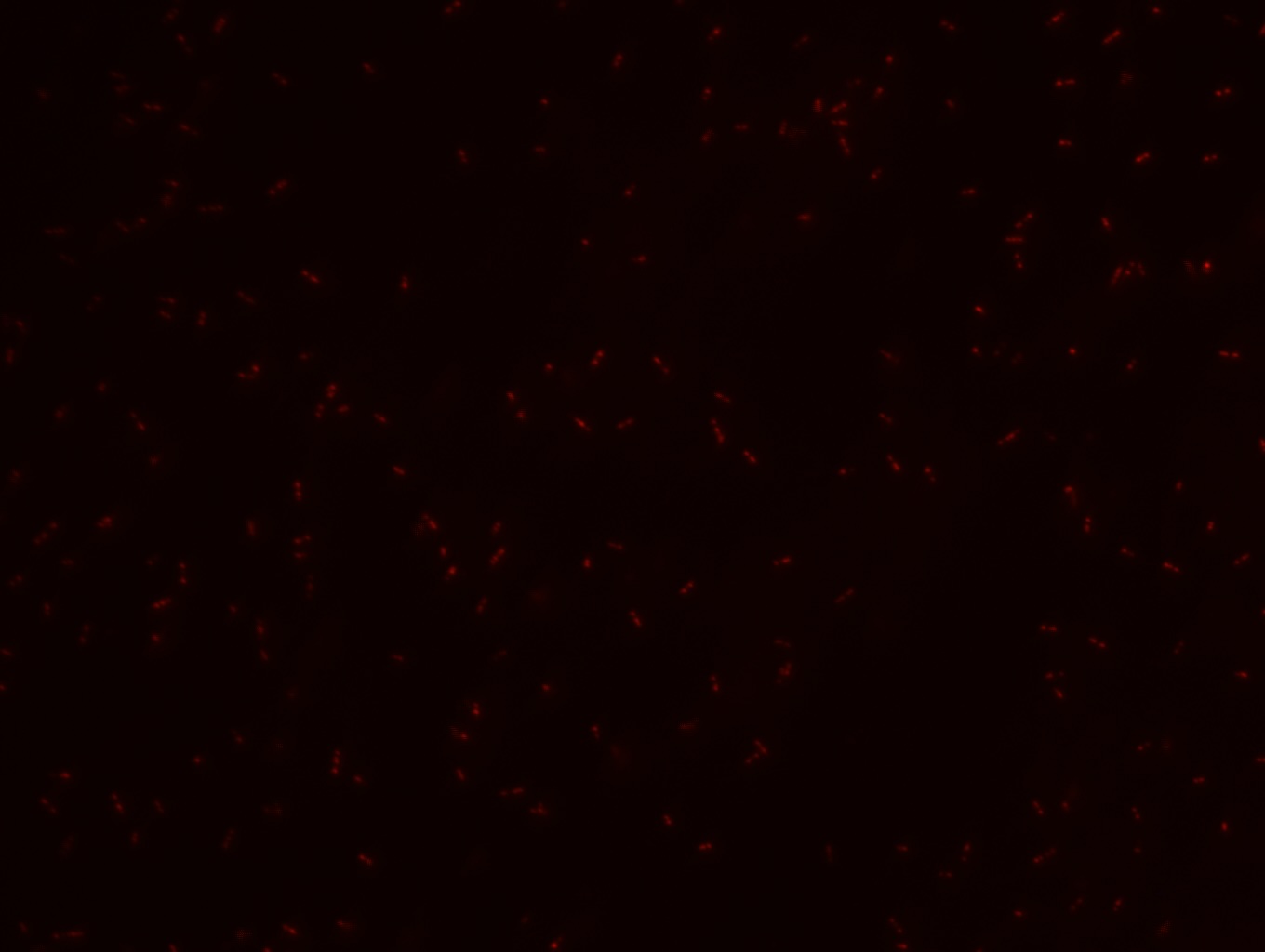

Supplement: File SI2 — Microscopy images of Halomonas sp. CUBES01. [file aem.00603-24-s0002.zip › Microscopy/Propionate_2nd_0003.jpg]

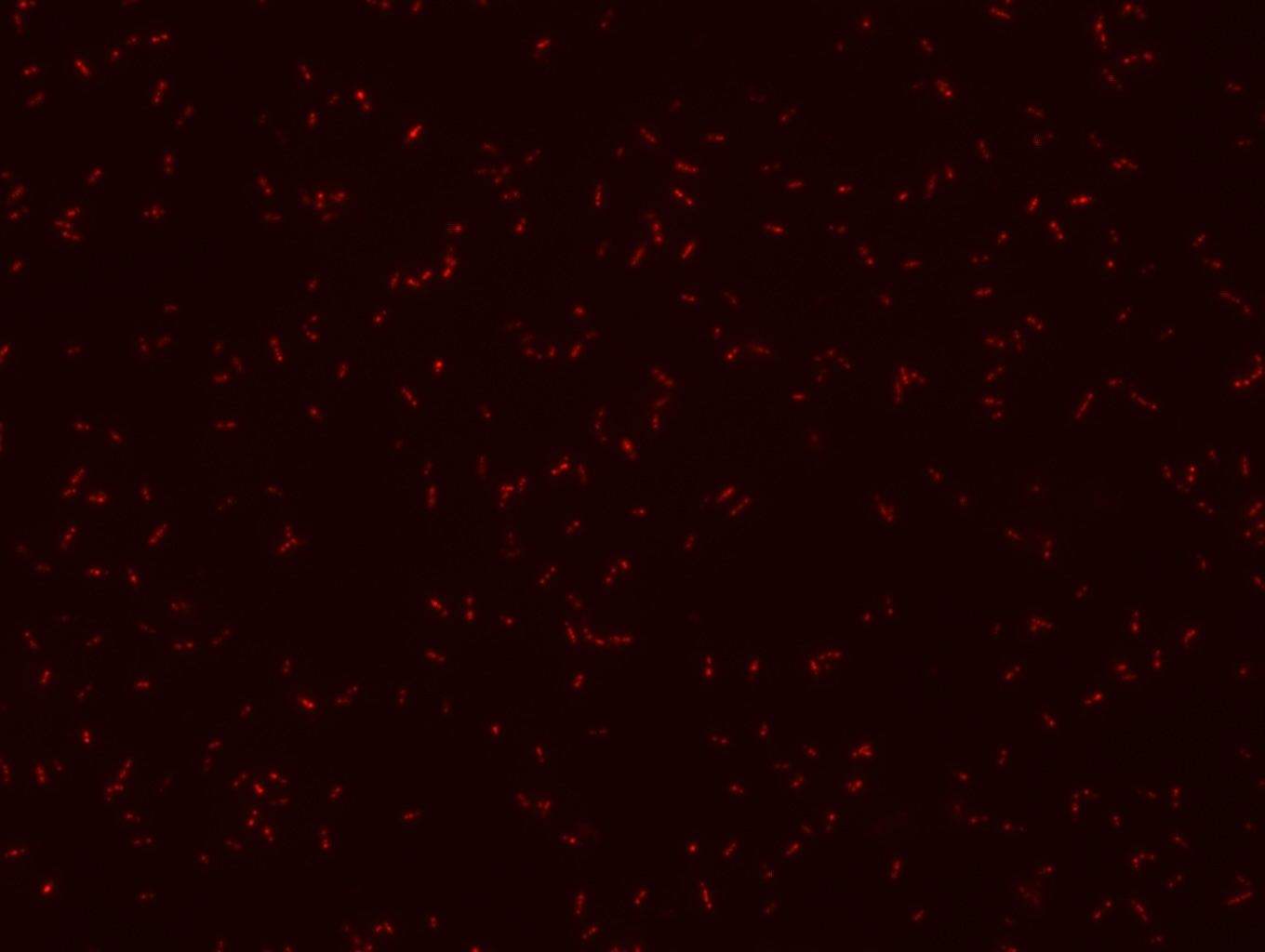

Supplement: File SI2 — Microscopy images of Halomonas sp. CUBES01. [file aem.00603-24-s0002.zip › Microscopy/Propionate_3rd_0001.jpg]

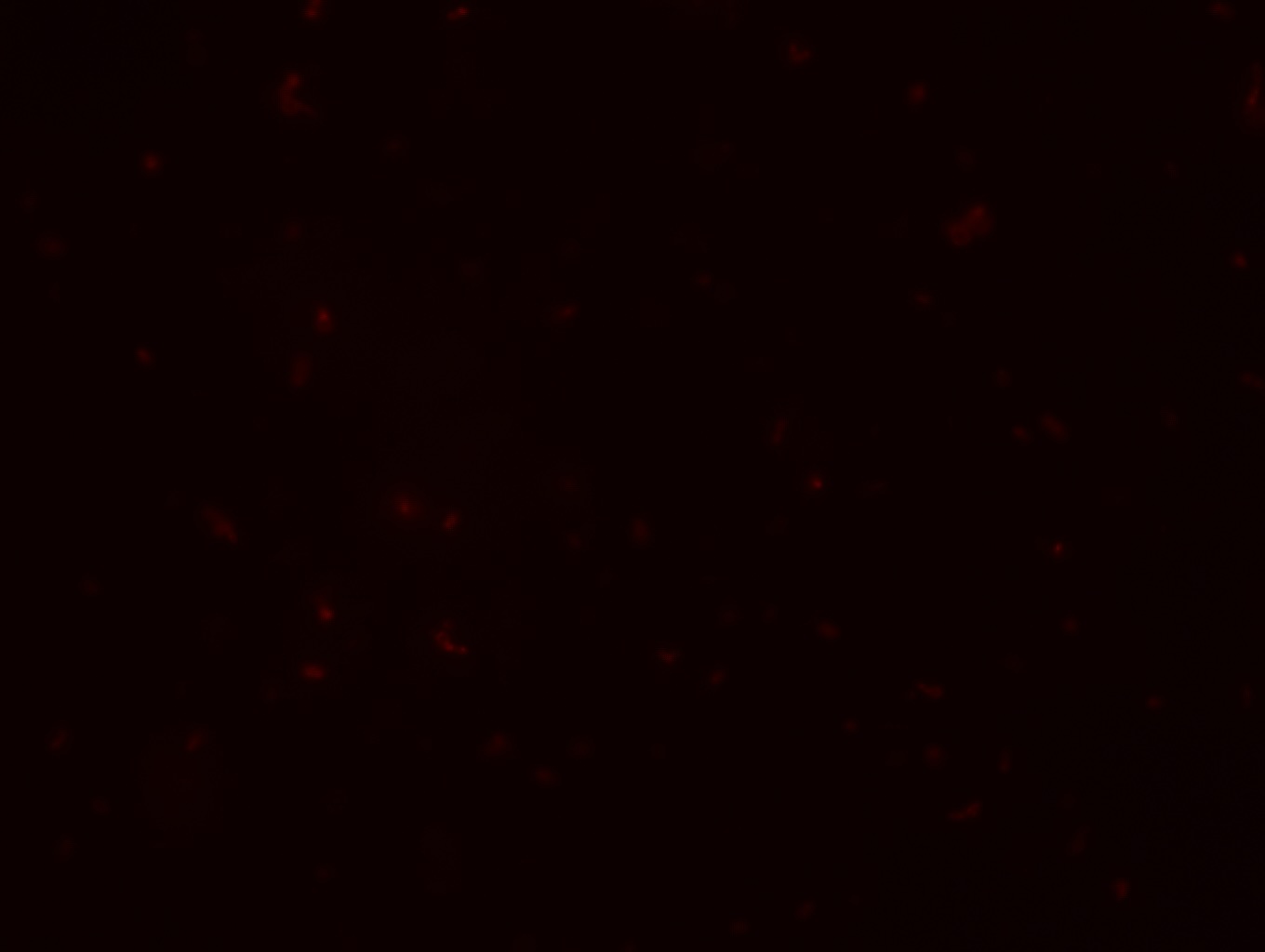

Supplement: File SI2 — Microscopy images of Halomonas sp. CUBES01. [file aem.00603-24-s0002.zip › Microscopy/NB_1st_0001.jpg]

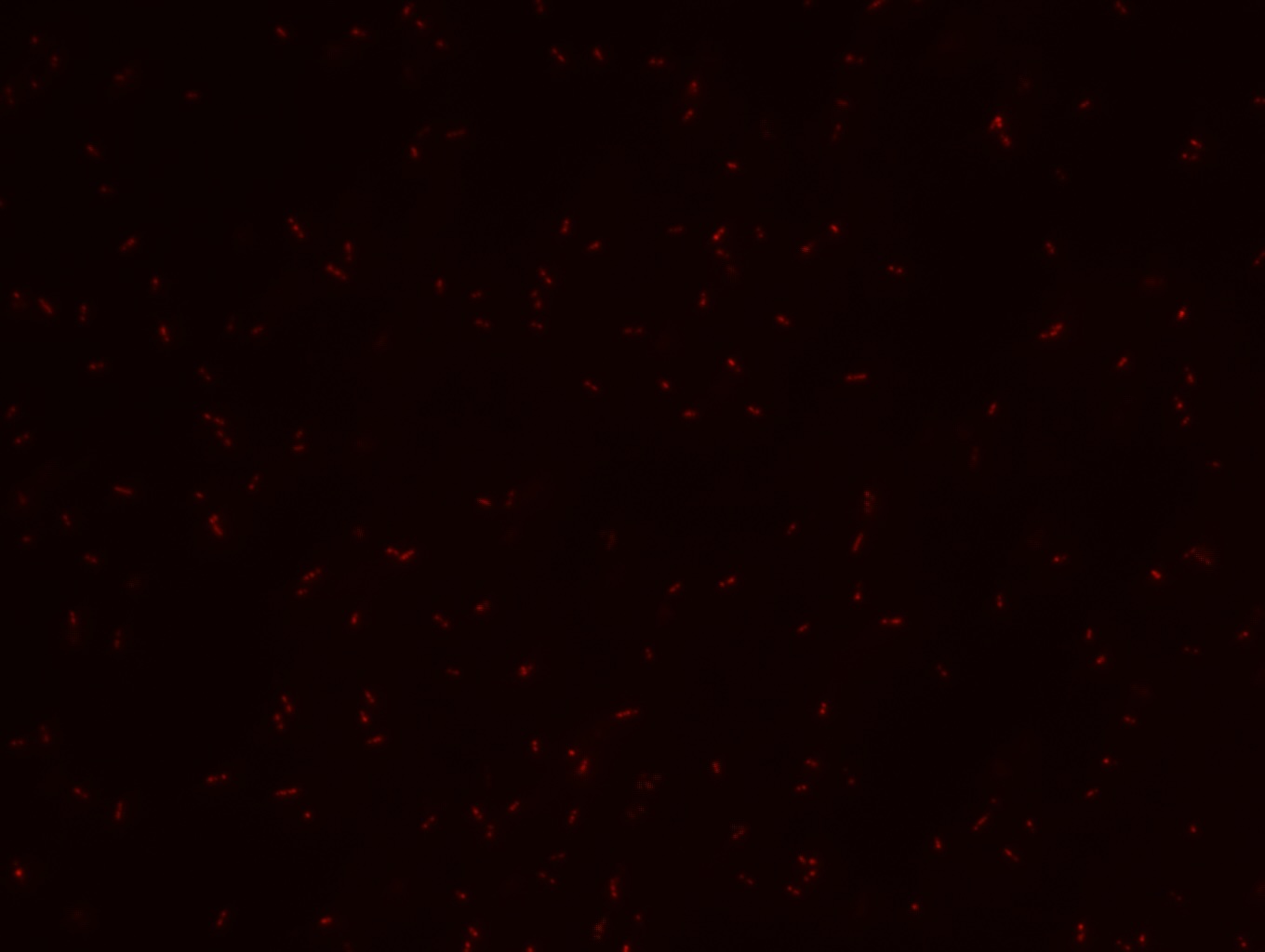

Supplement: File SI2 — Microscopy images of Halomonas sp. CUBES01. [file aem.00603-24-s0002.zip › Microscopy/Propionate_2nd_0002.jpg]

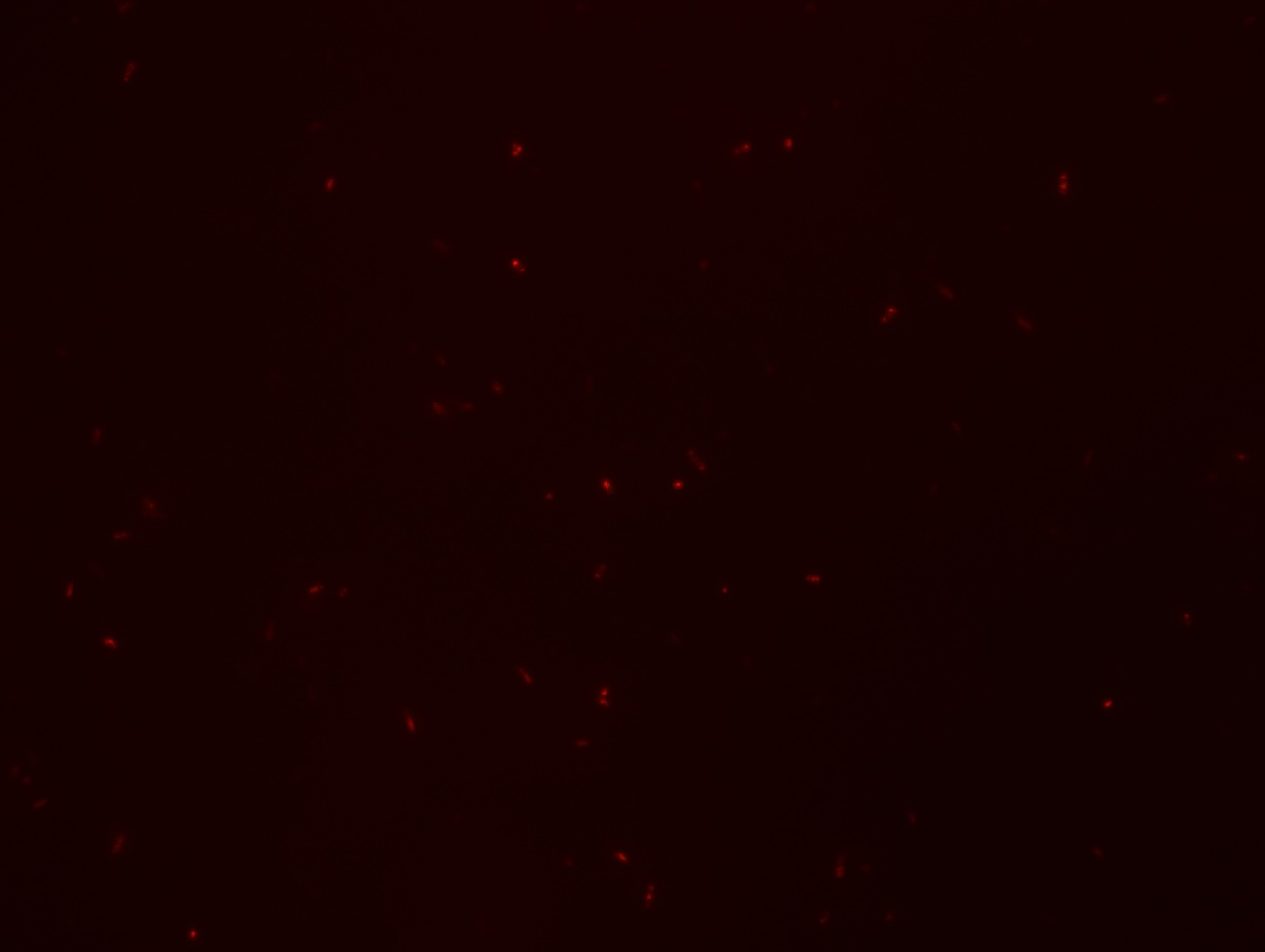

Supplement: File SI2 — Microscopy images of Halomonas sp. CUBES01. [file aem.00603-24-s0002.zip › Microscopy/Acetyl-Glucosamine_2nd_0003.jpg]

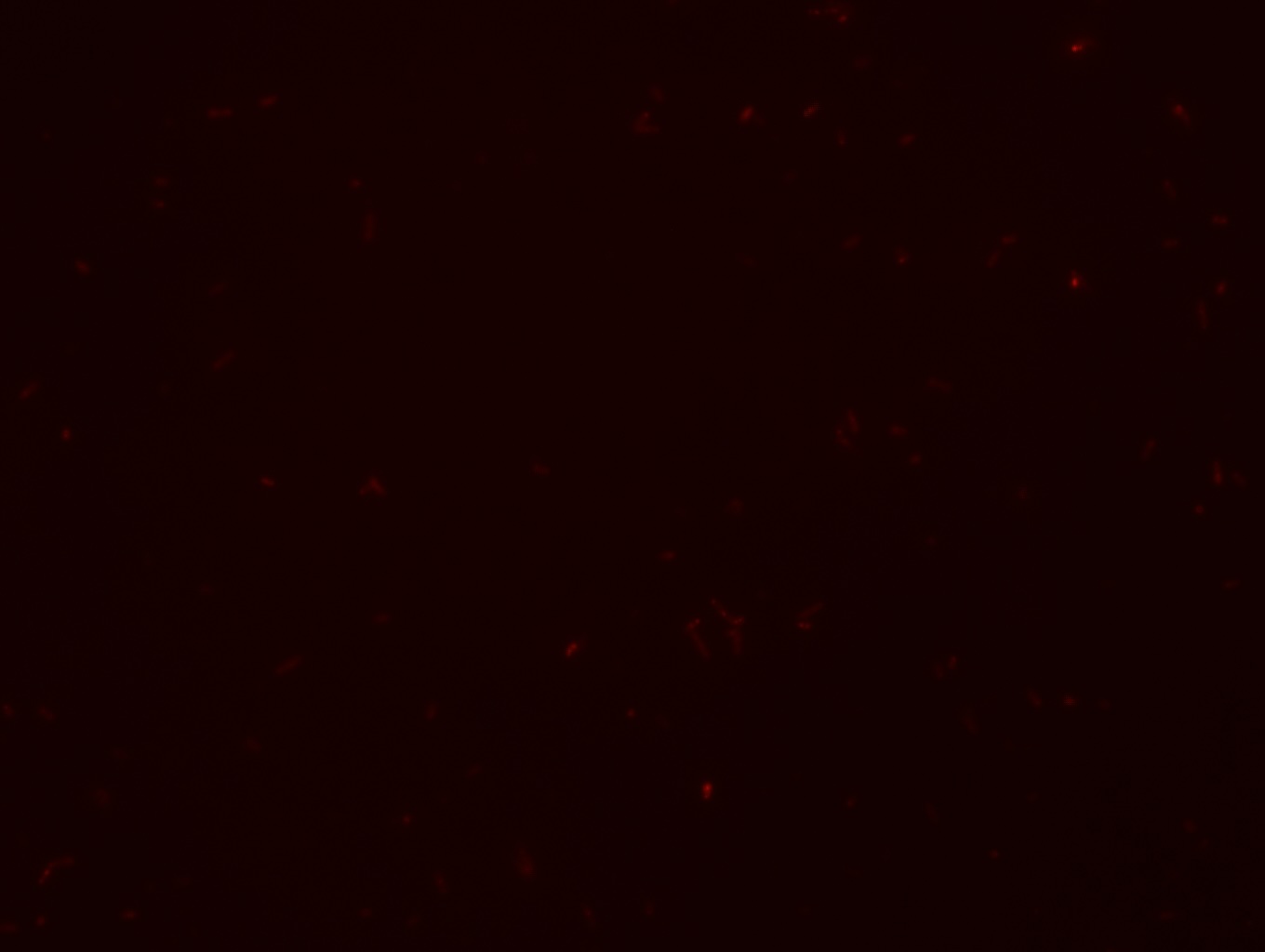

Supplement: File SI2 — Microscopy images of Halomonas sp. CUBES01. [file aem.00603-24-s0002.zip › Microscopy/Acetyl-Glucosamine_3rd_0001.jpg]

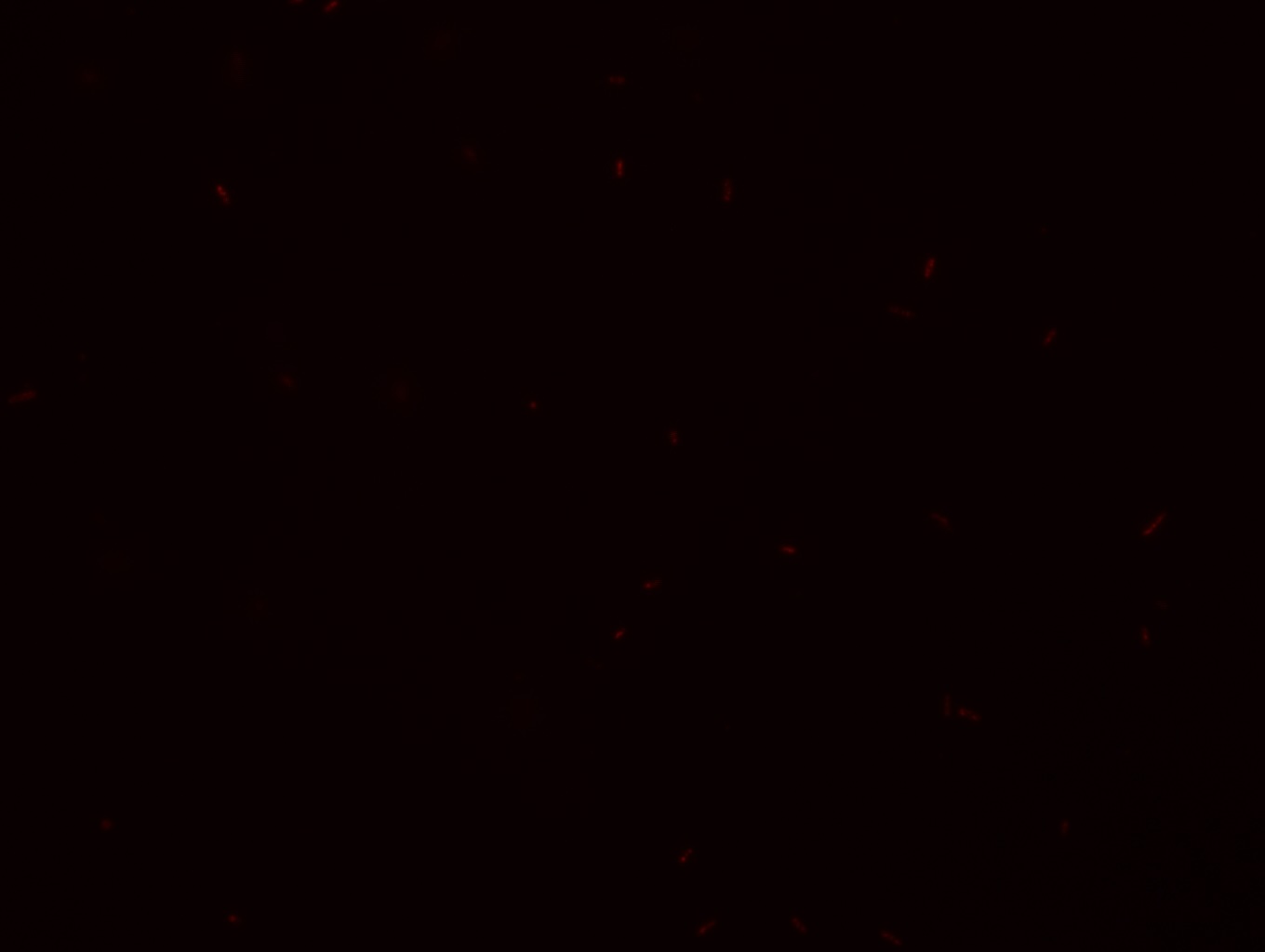

Supplement: File SI2 — Microscopy images of Halomonas sp. CUBES01. [file aem.00603-24-s0002.zip › Microscopy/Glucosamine_1st_0004.jpg]

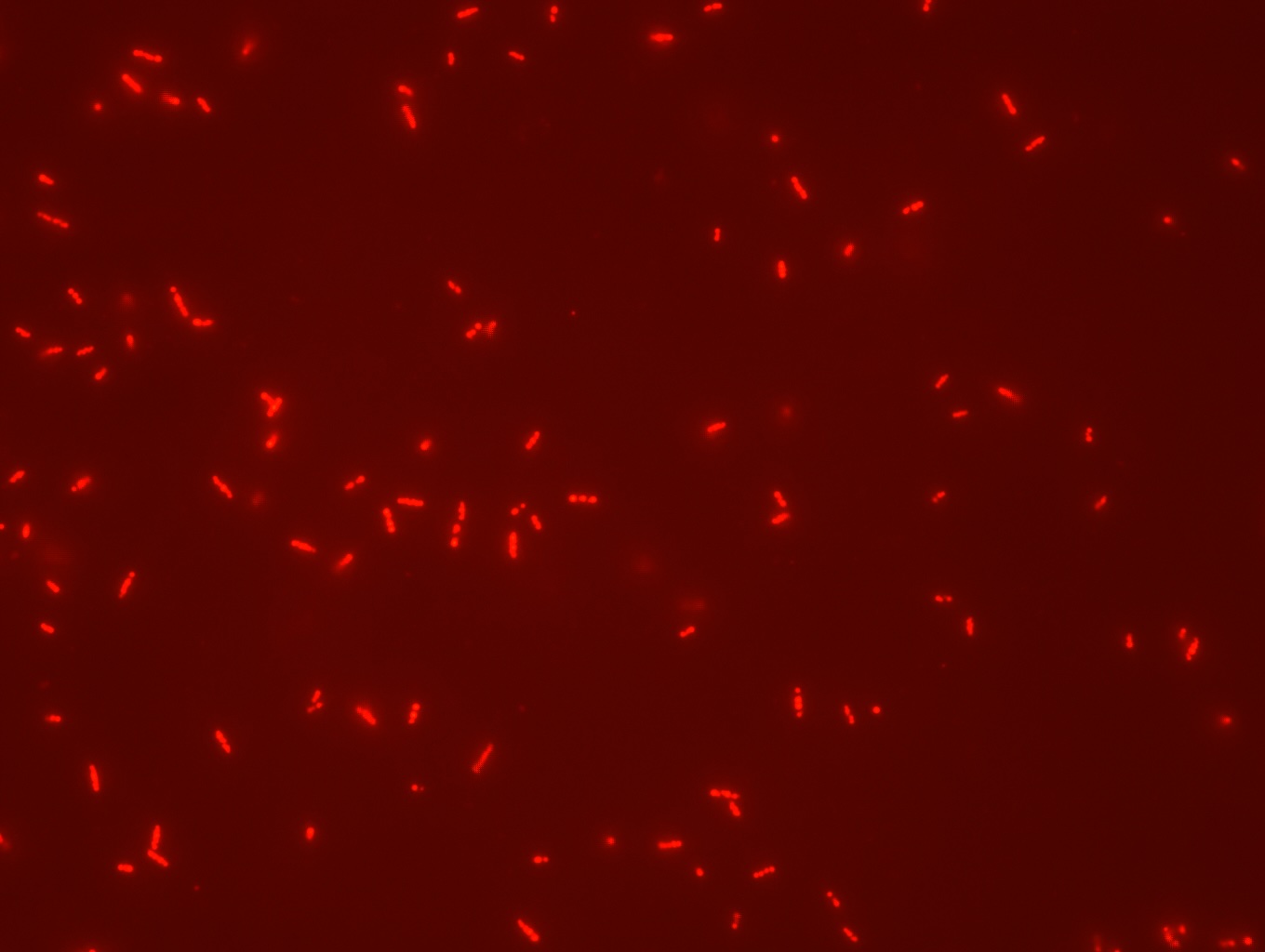

Supplement: File SI2 — Microscopy images of Halomonas sp. CUBES01. [file aem.00603-24-s0002.zip › Microscopy/Acetate_3rd_0004.jpg]

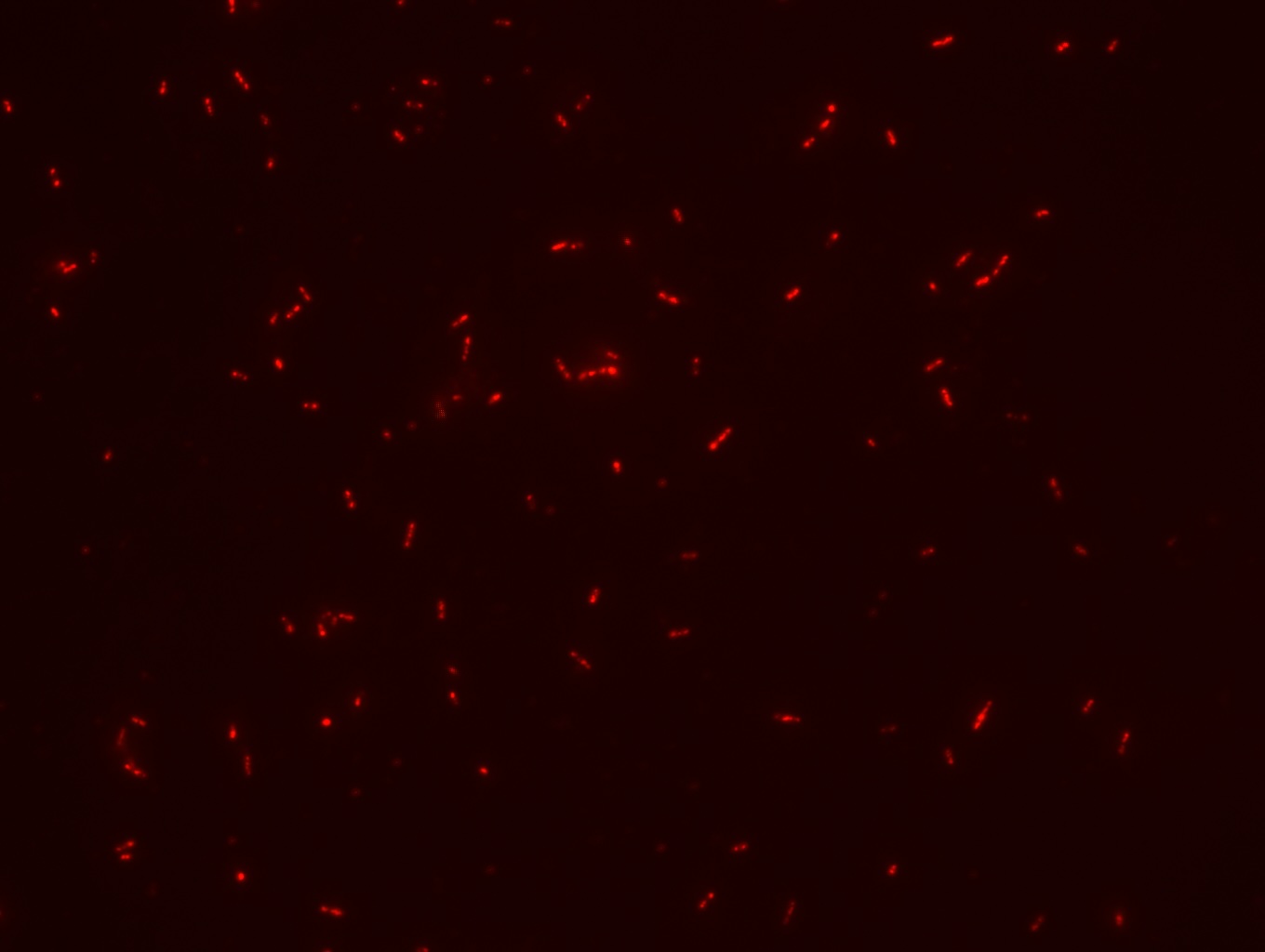

Supplement: File SI2 — Microscopy images of Halomonas sp. CUBES01. [file aem.00603-24-s0002.zip › Microscopy/Glycerol_1st_0004.jpg]

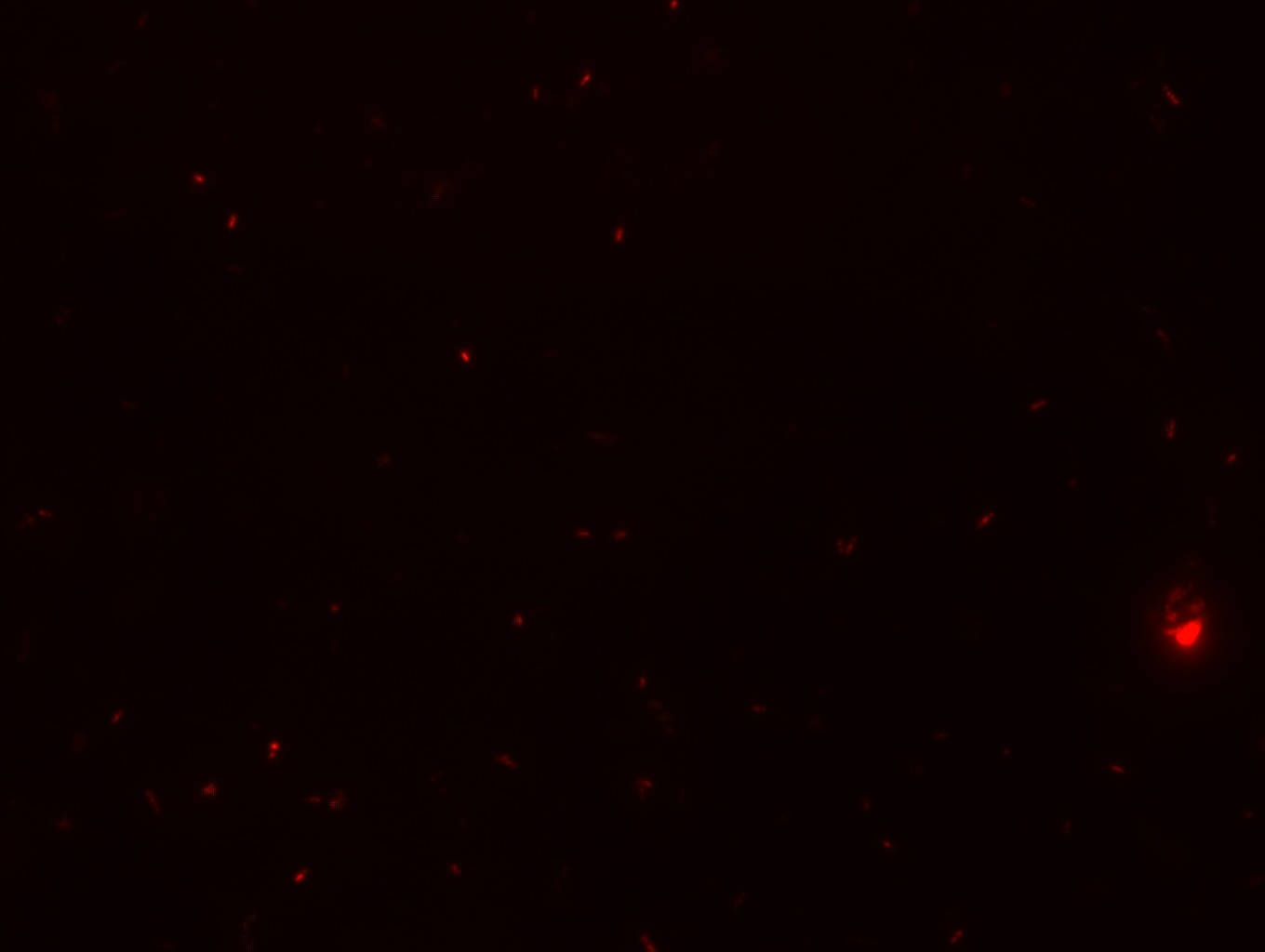

Supplement: File SI2 — Microscopy images of Halomonas sp. CUBES01. [file aem.00603-24-s0002.zip › Microscopy/Fructose_3rd_0004.jpg]

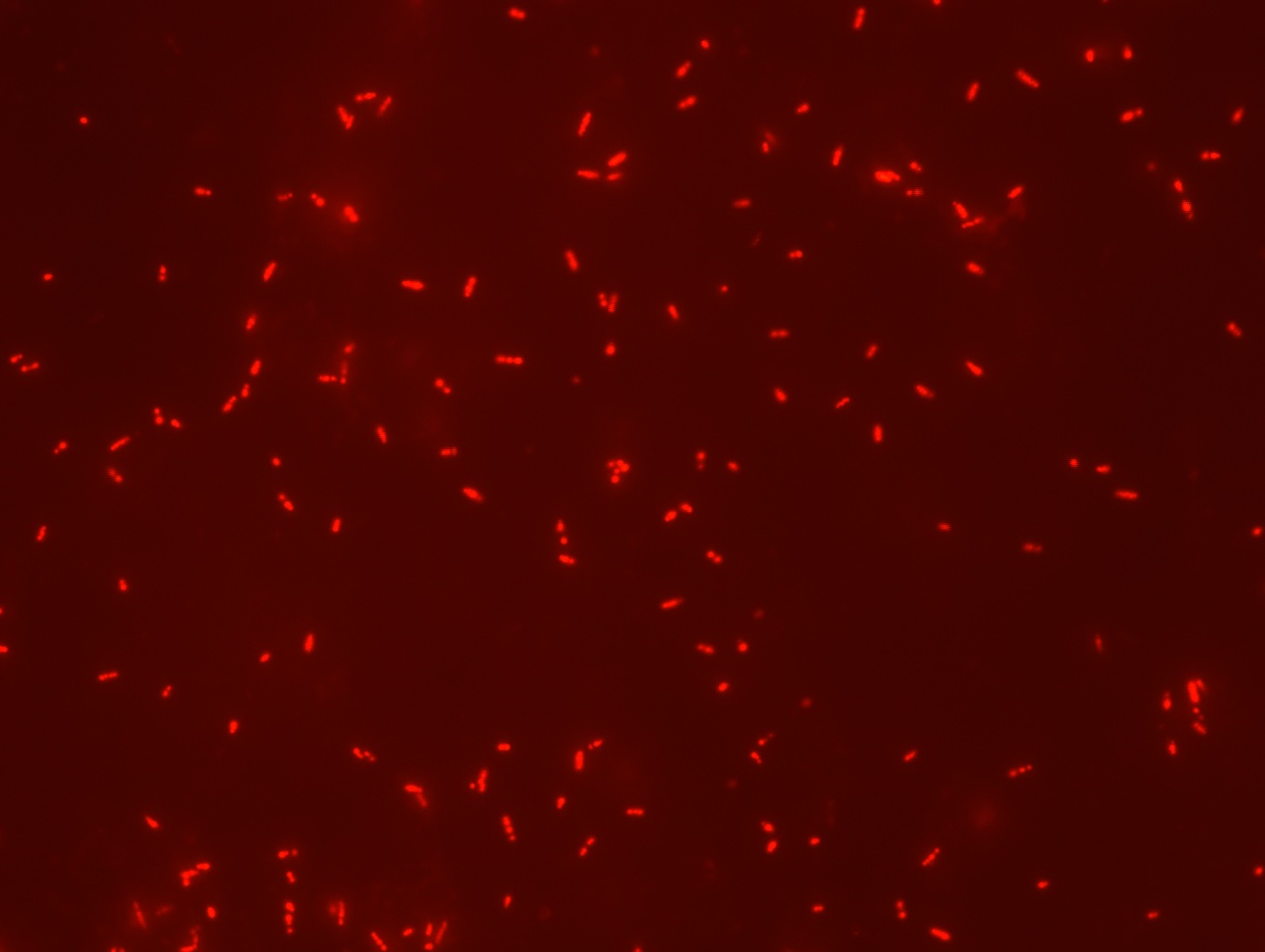

Supplement: File SI2 — Microscopy images of Halomonas sp. CUBES01. [file aem.00603-24-s0002.zip › Microscopy/Glucose_2nd_0004.jpg]

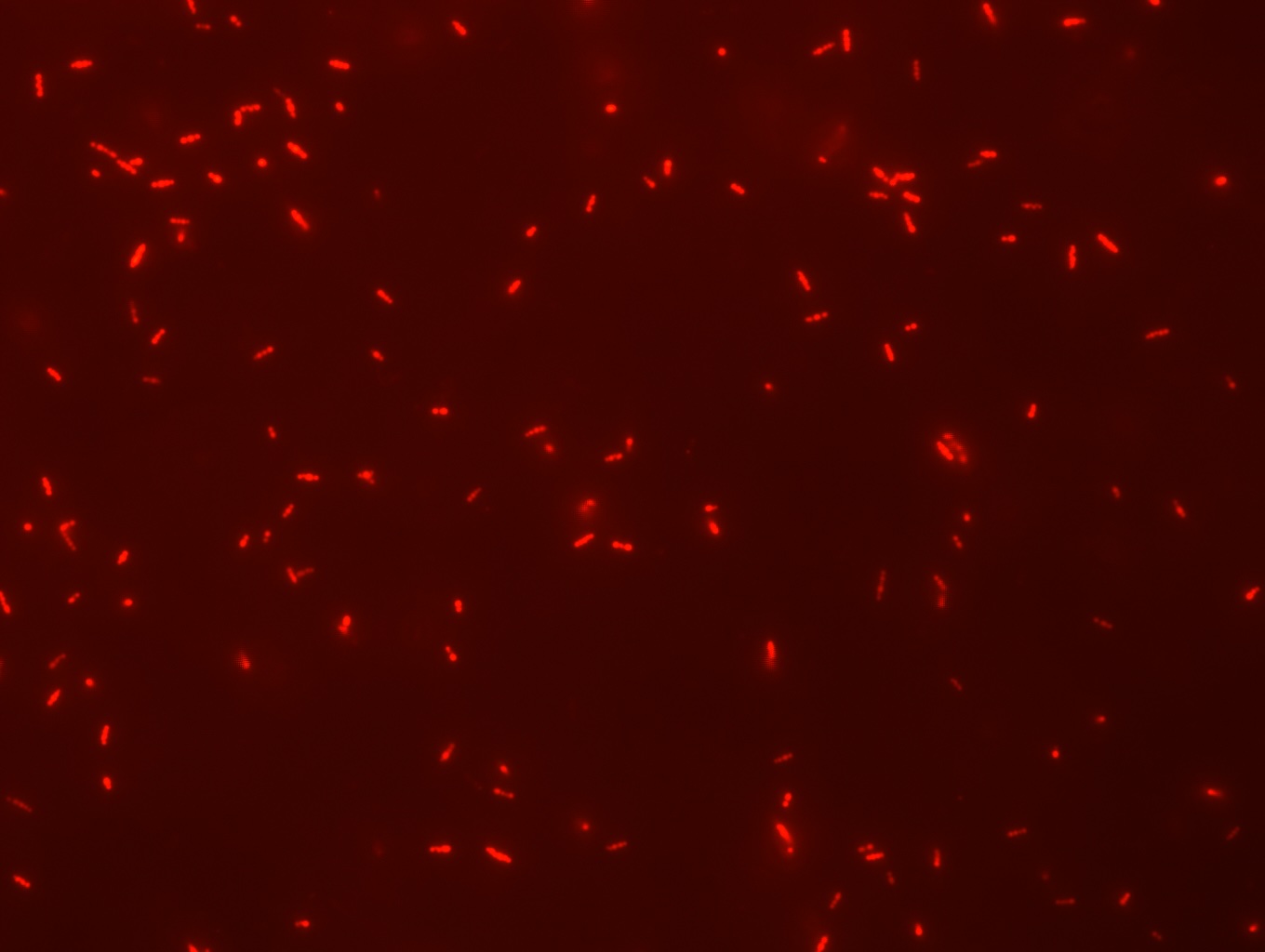

Supplement: File SI2 — Microscopy images of Halomonas sp. CUBES01. [file aem.00603-24-s0002.zip › Microscopy/Glucose_3rd_0004.jpg]

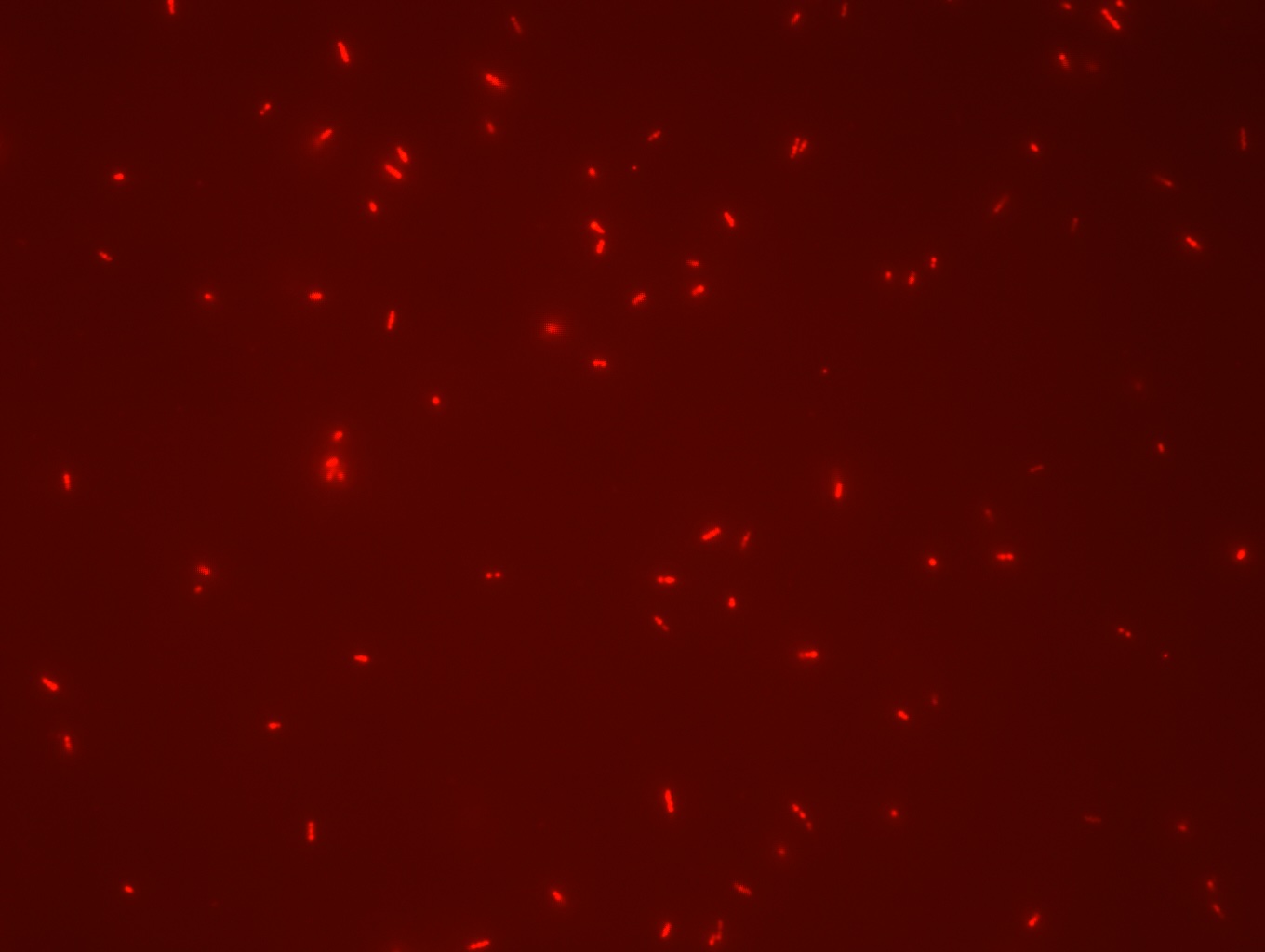

Supplement: File SI2 — Microscopy images of Halomonas sp. CUBES01. [file aem.00603-24-s0002.zip › Microscopy/Acetate_2nd_0004.jpg]

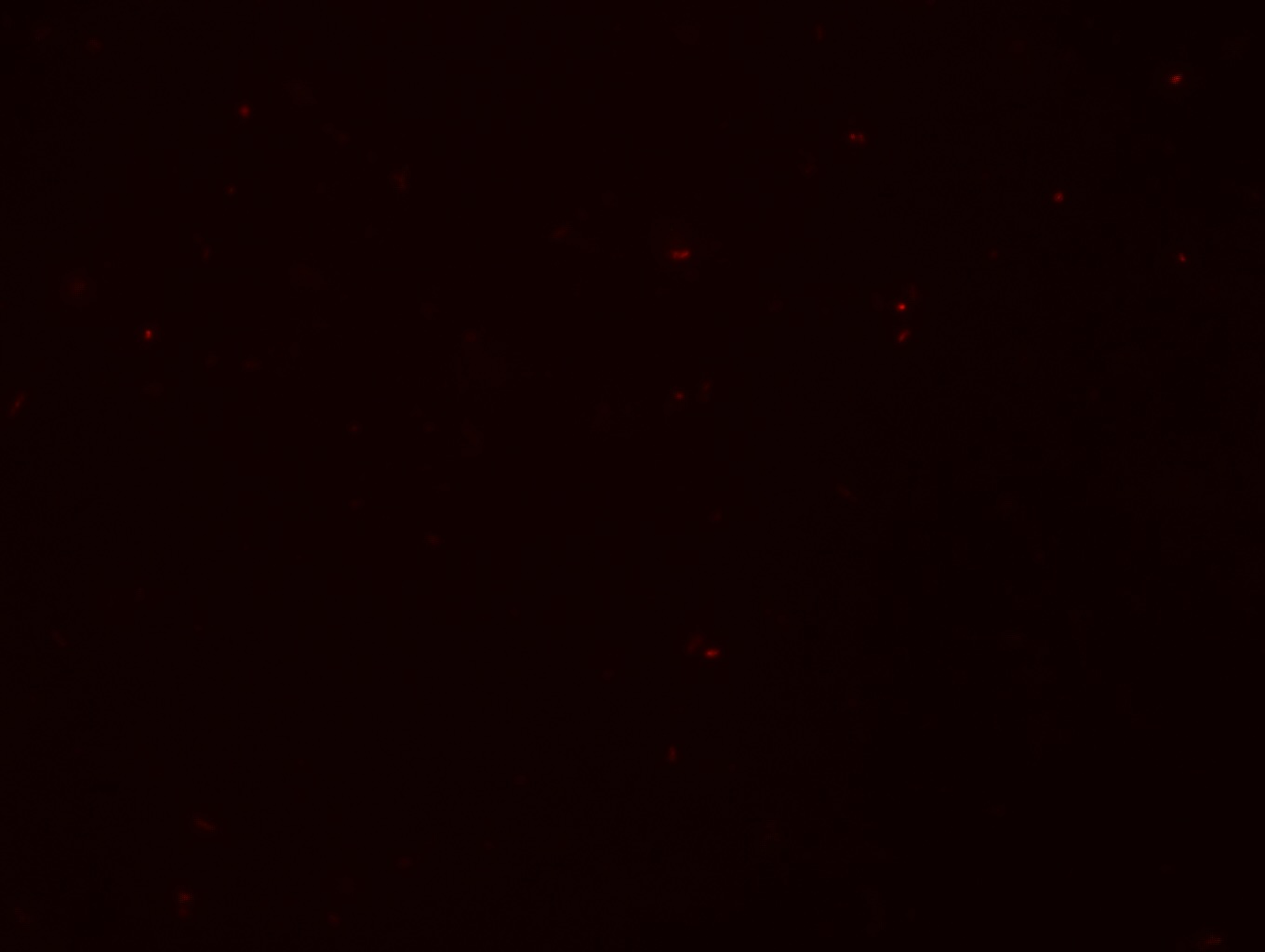

Supplement: File SI2 — Microscopy images of Halomonas sp. CUBES01. [file aem.00603-24-s0002.zip › Microscopy/Fructose_2nd_0004.jpg]

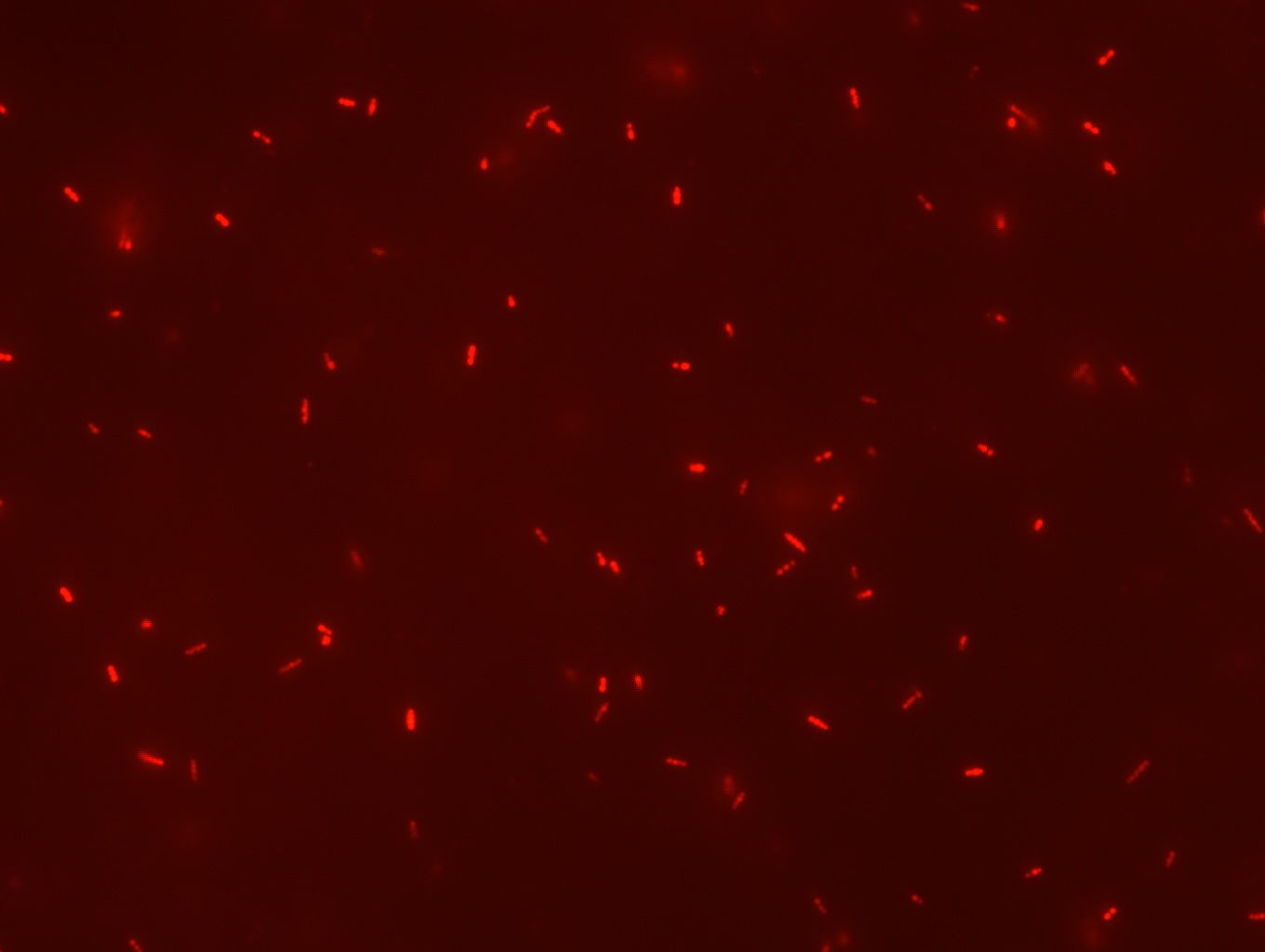

Supplement: File SI2 — Microscopy images of Halomonas sp. CUBES01. [file aem.00603-24-s0002.zip › Microscopy/Glucose_3rd_0001.jpg]

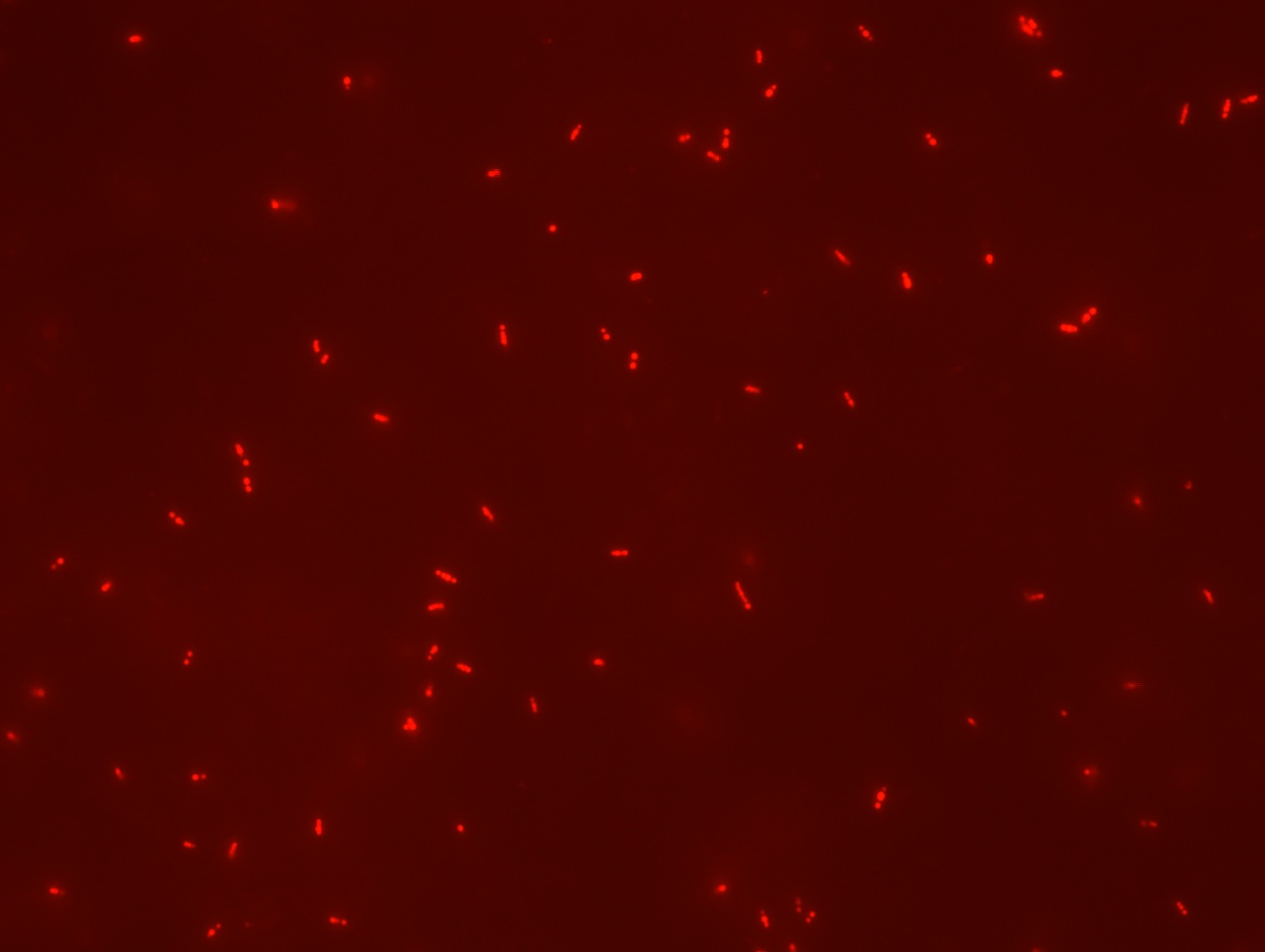

Supplement: File SI2 — Microscopy images of Halomonas sp. CUBES01. [file aem.00603-24-s0002.zip › Microscopy/Glucose_2nd_0003.jpg]

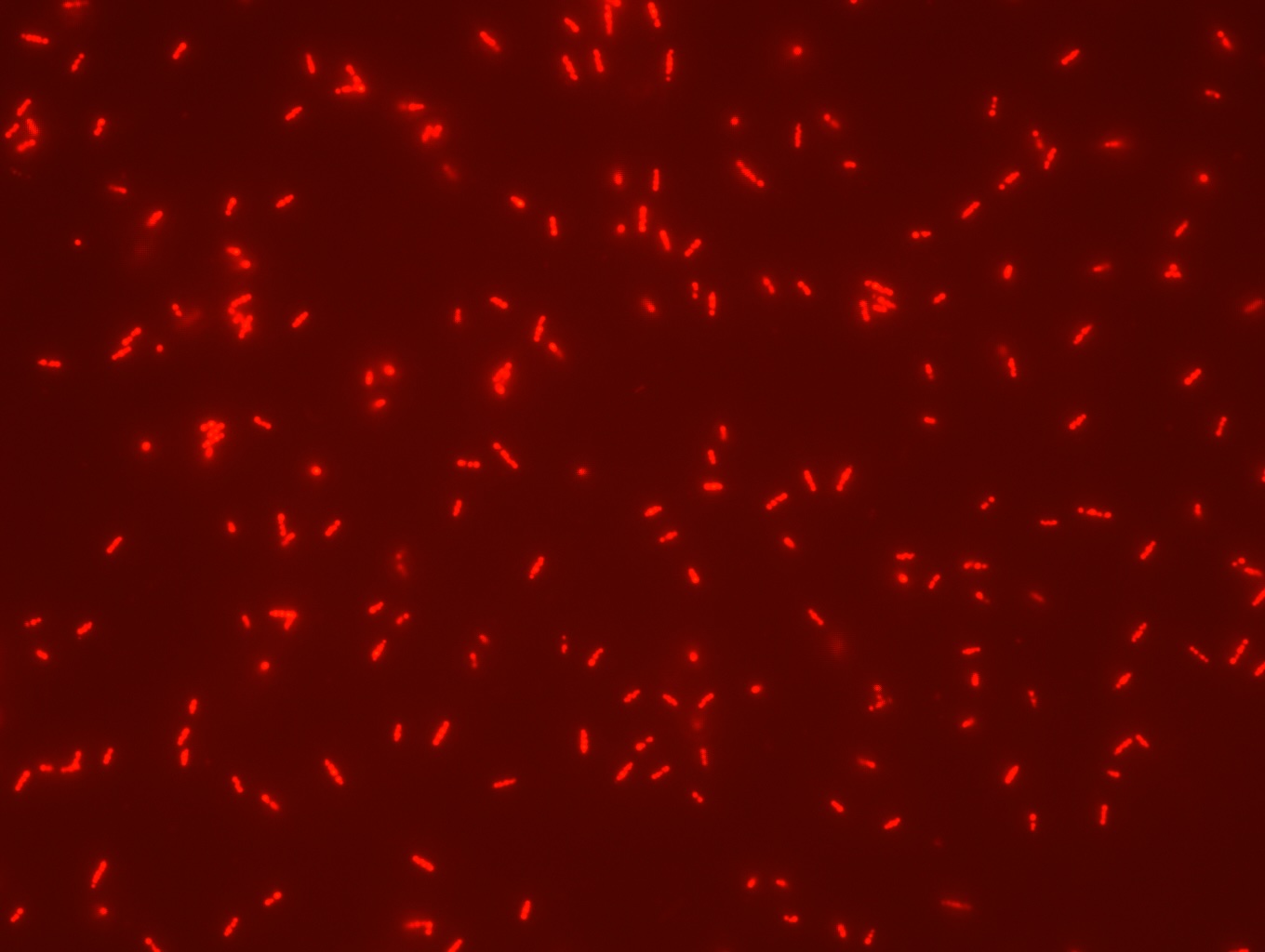

Supplement: File SI2 — Microscopy images of Halomonas sp. CUBES01. [file aem.00603-24-s0002.zip › Microscopy/Acetate_3rd_0002.jpg]

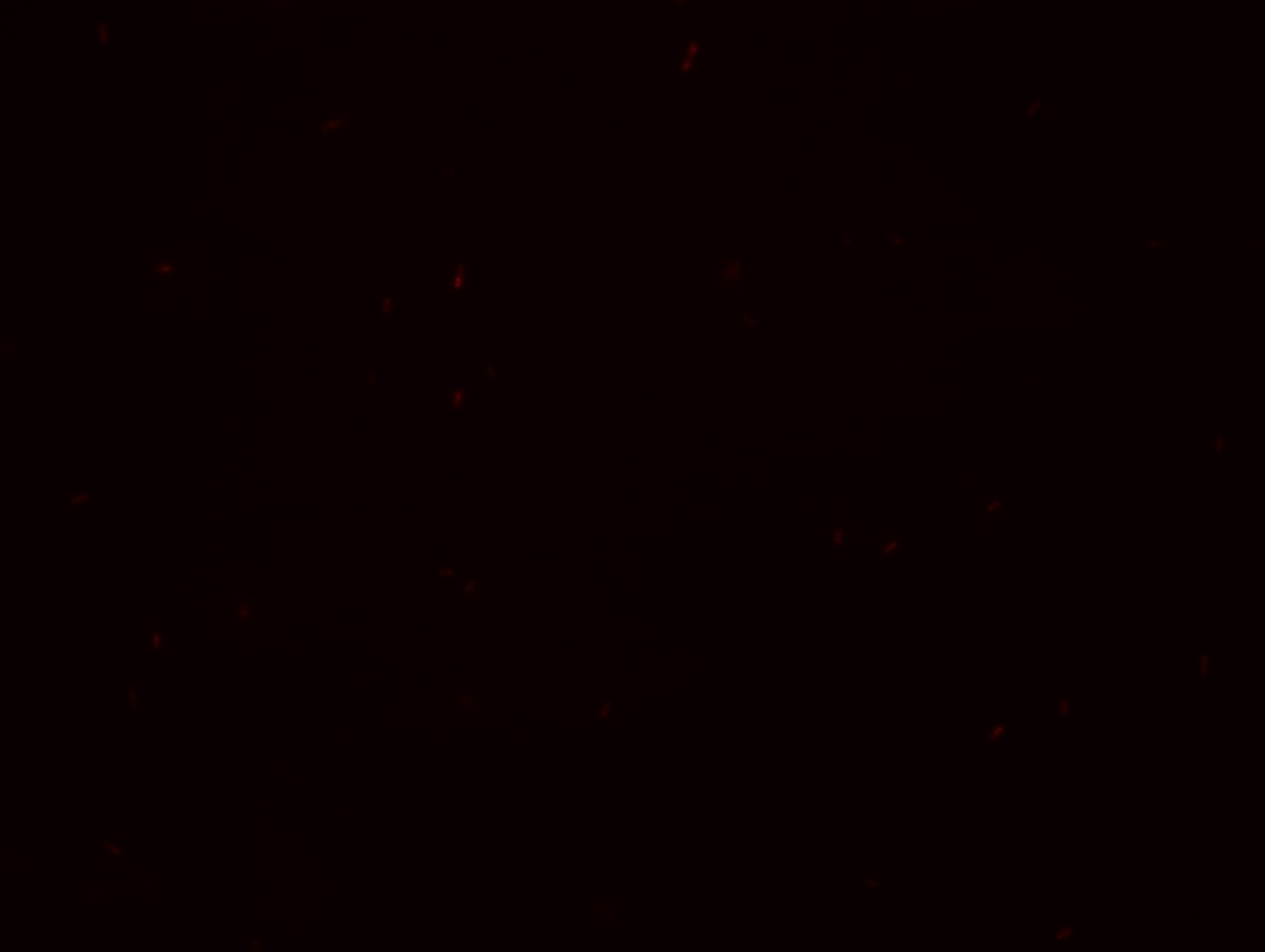

Supplement: File SI2 — Microscopy images of Halomonas sp. CUBES01. [file aem.00603-24-s0002.zip › Microscopy/Glucosamine_1st_0002.jpg]

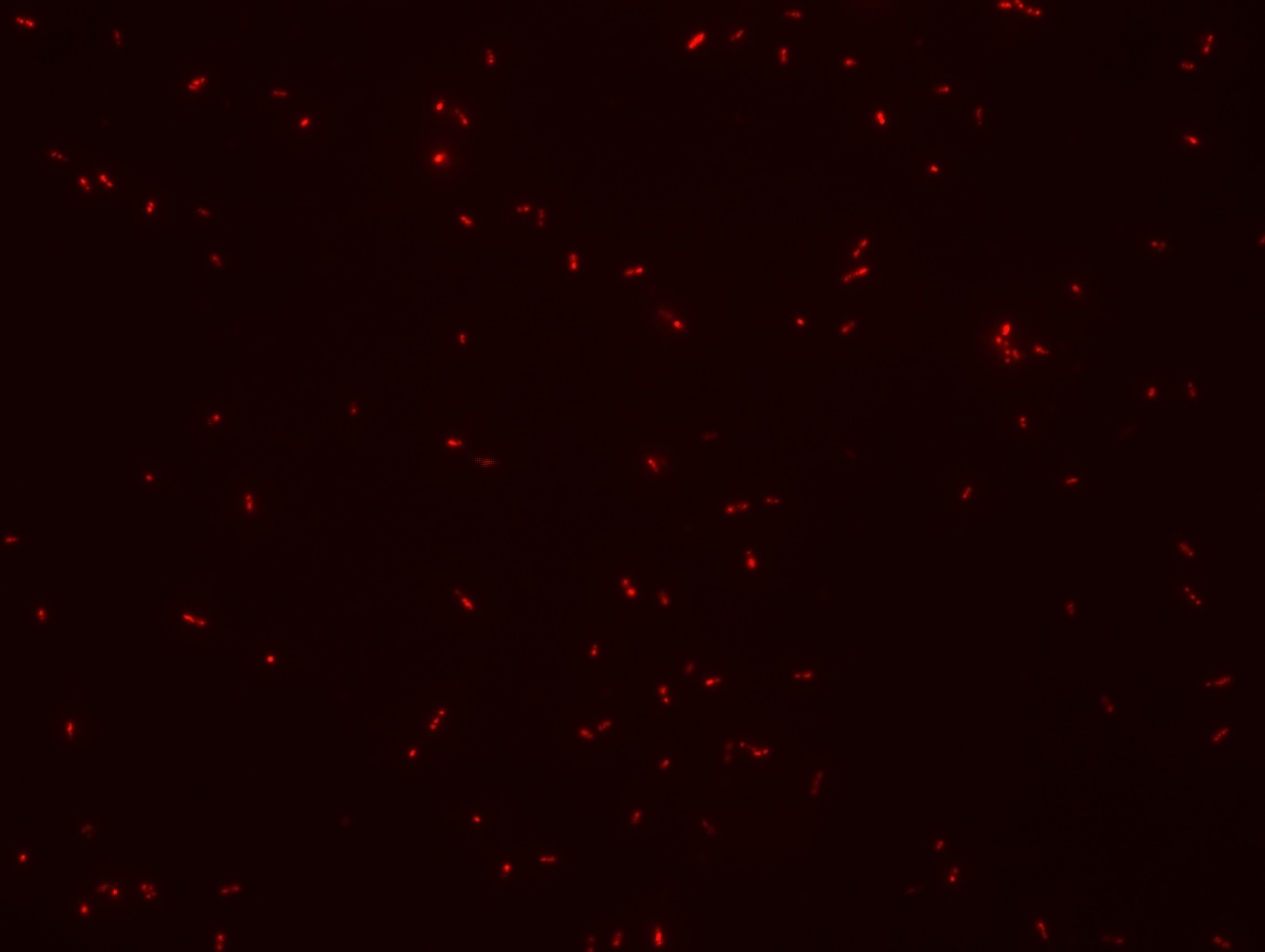

Supplement: File SI2 — Microscopy images of Halomonas sp. CUBES01. [file aem.00603-24-s0002.zip › Microscopy/Glycerol_1st_0002.jpg]

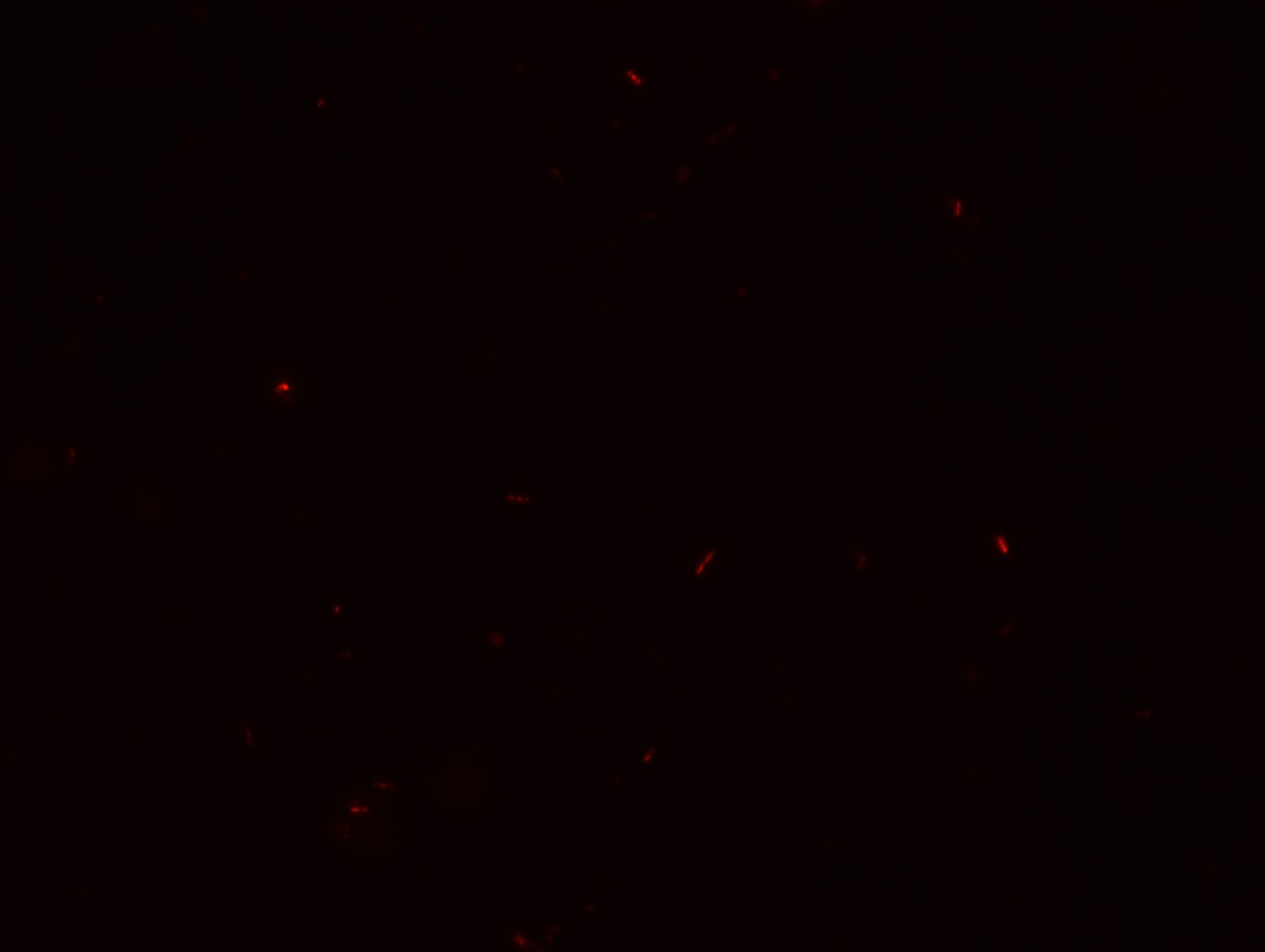

Supplement: File SI2 — Microscopy images of Halomonas sp. CUBES01. [file aem.00603-24-s0002.zip › Microscopy/Fructose_3rd_0002.jpg]

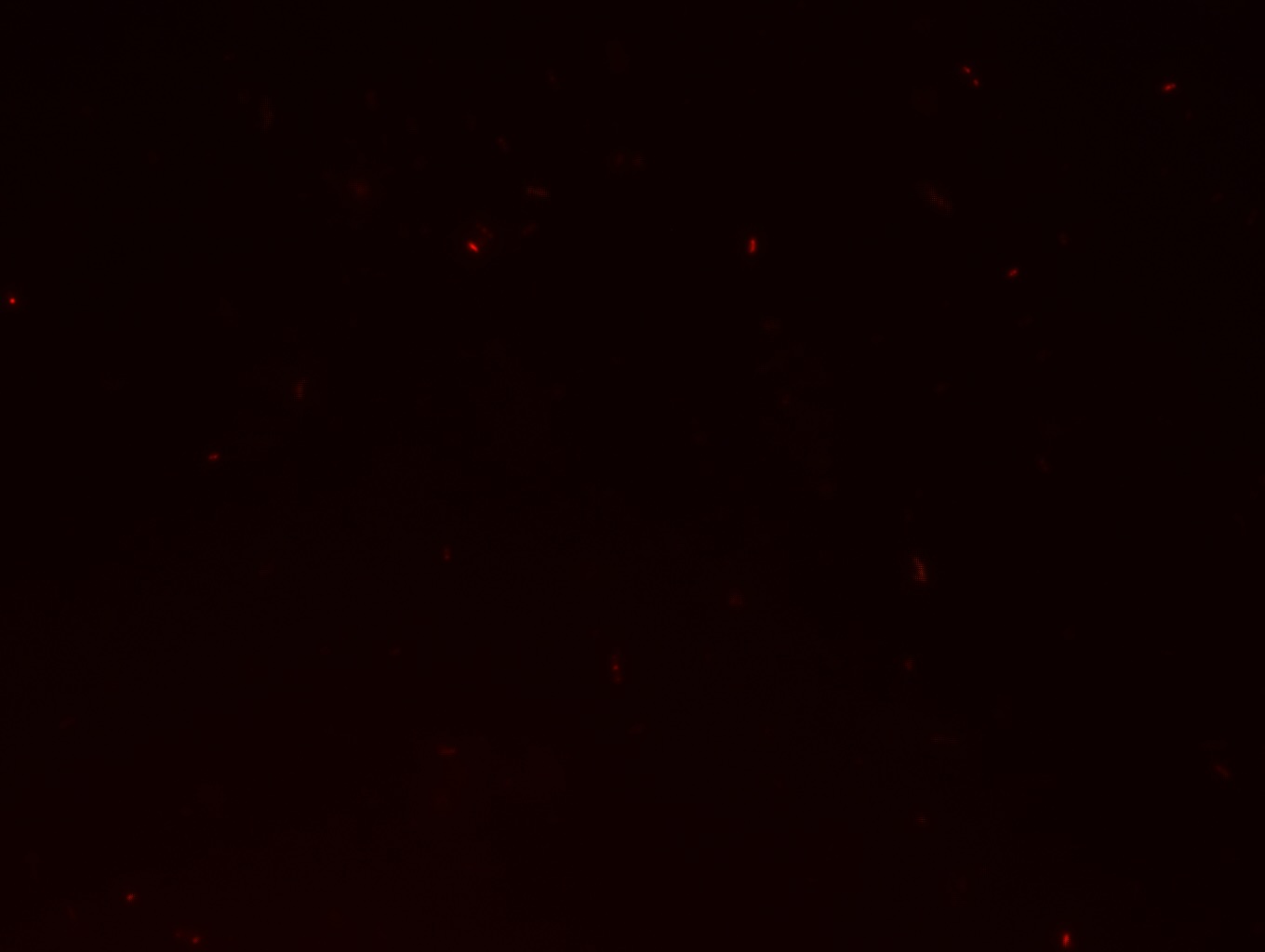

Supplement: File SI2 — Microscopy images of Halomonas sp. CUBES01. [file aem.00603-24-s0002.zip › Microscopy/Fructose_3rd_0003.jpg]

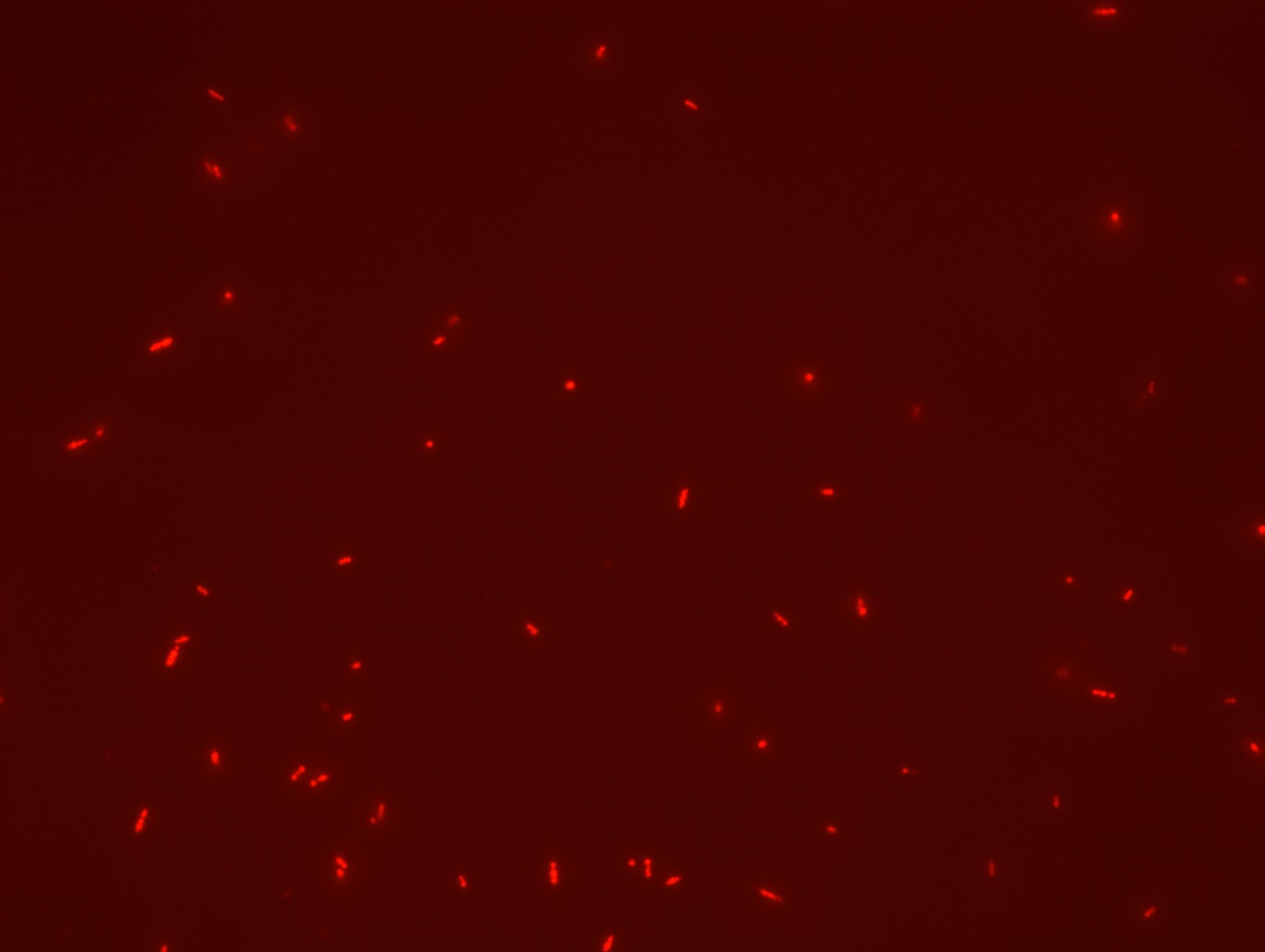

Supplement: File SI2 — Microscopy images of Halomonas sp. CUBES01. [file aem.00603-24-s0002.zip › Microscopy/Acetate_2nd_0001.jpg]

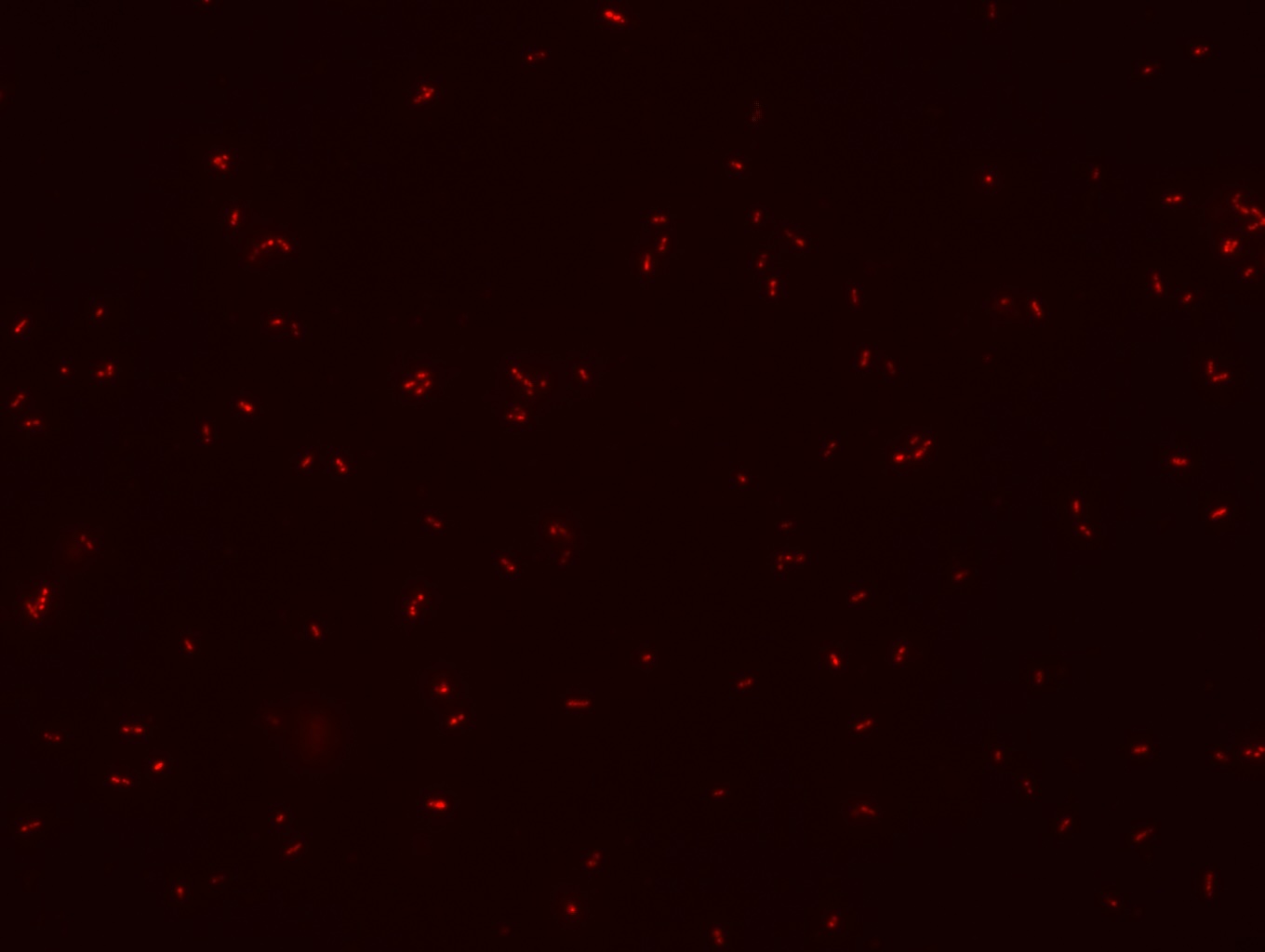

Supplement: File SI2 — Microscopy images of Halomonas sp. CUBES01. [file aem.00603-24-s0002.zip › Microscopy/Glycerol_1st_0003.jpg]

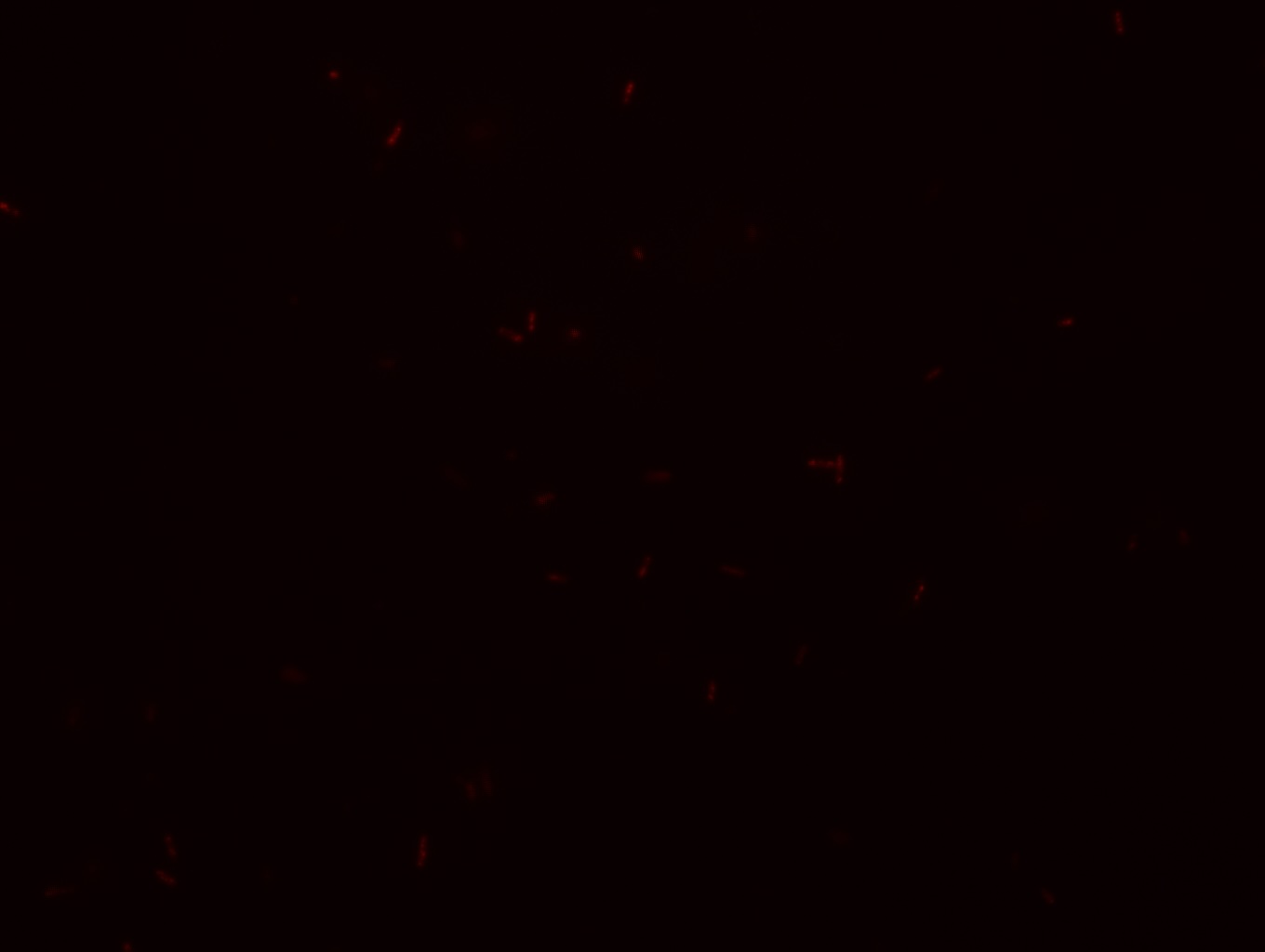

Supplement: File SI2 — Microscopy images of Halomonas sp. CUBES01. [file aem.00603-24-s0002.zip › Microscopy/Glucosamine_1st_0003.jpg]

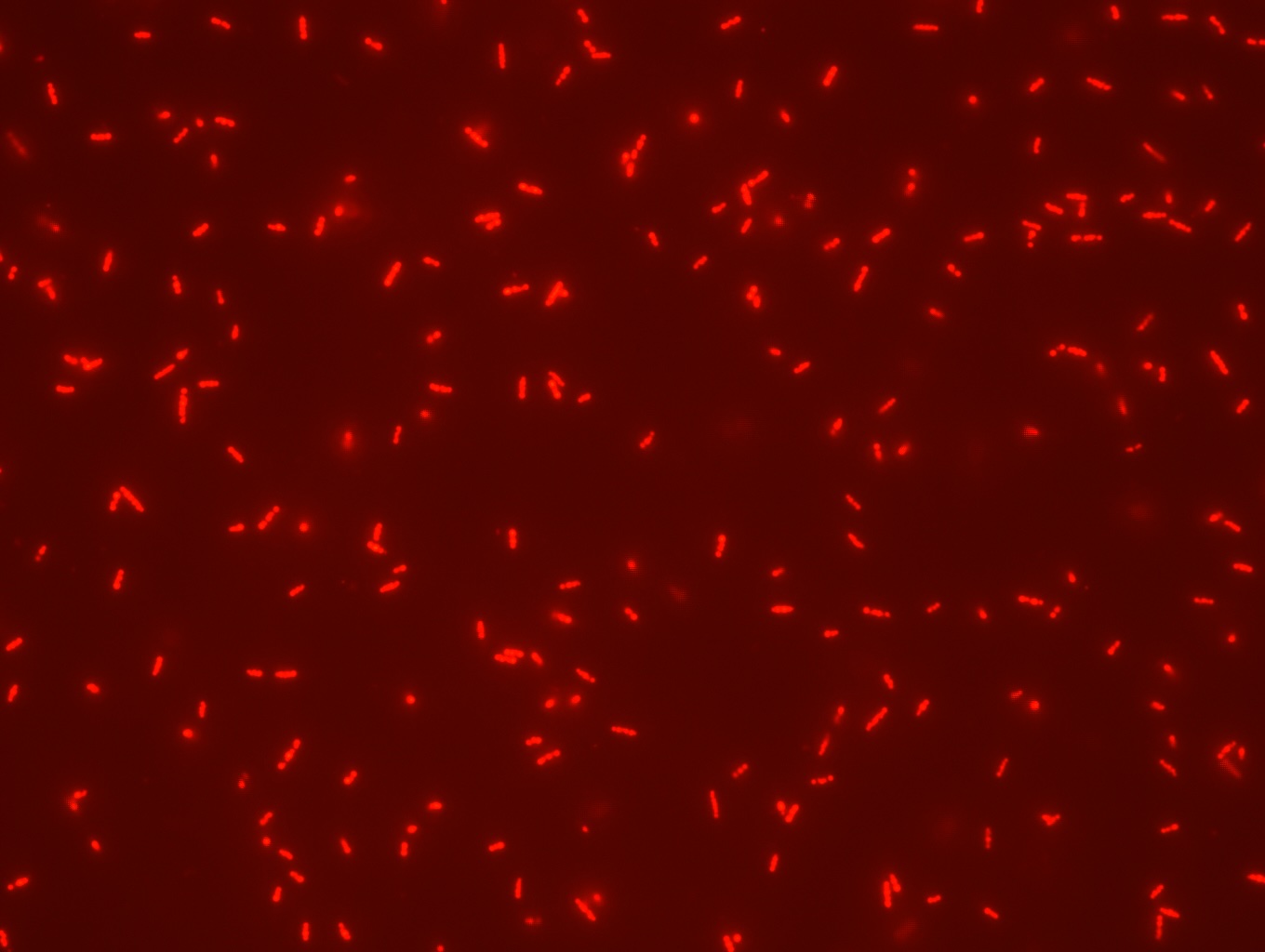

Supplement: File SI2 — Microscopy images of Halomonas sp. CUBES01. [file aem.00603-24-s0002.zip › Microscopy/Acetate_3rd_0003.jpg]

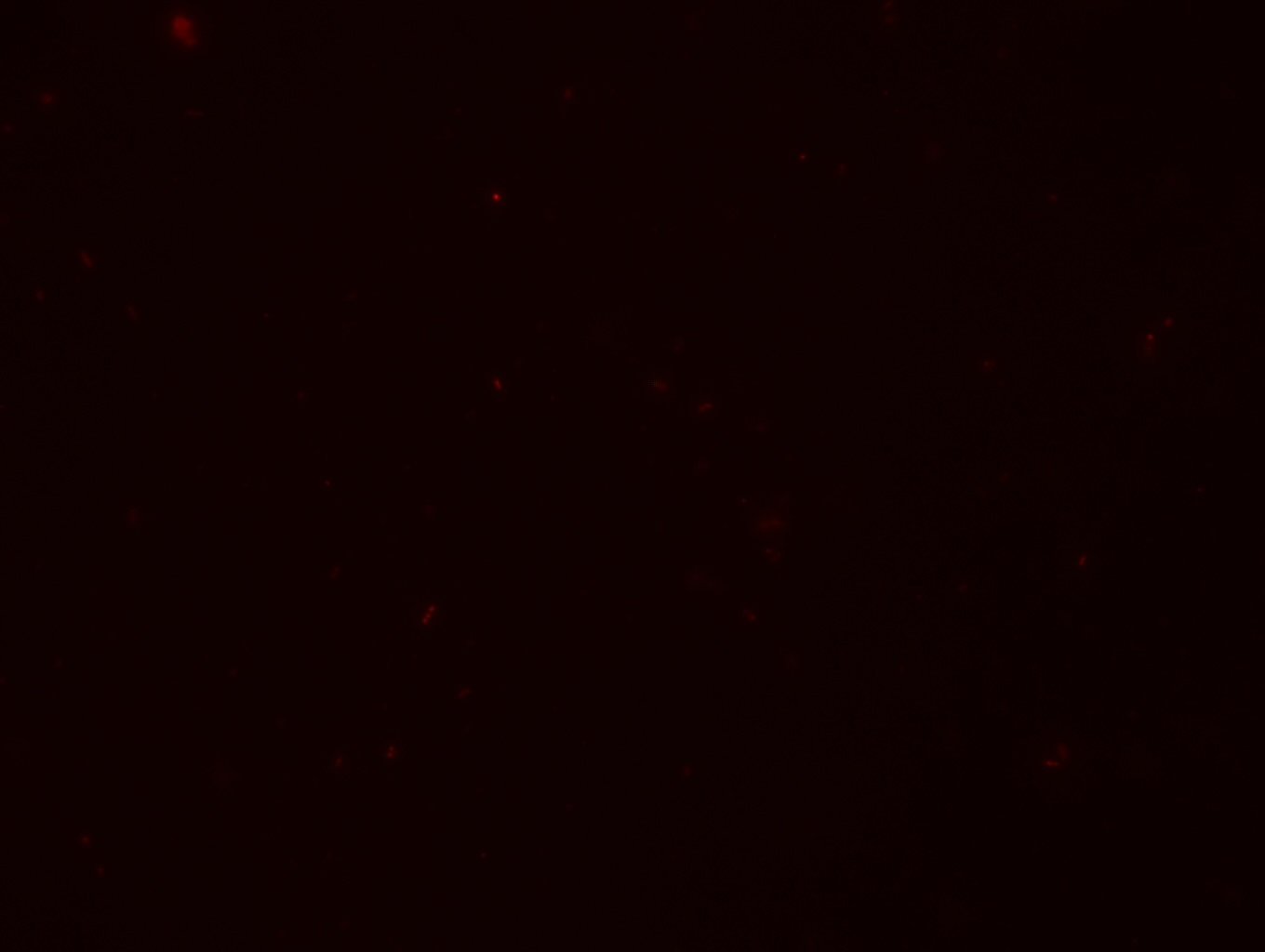

Supplement: File SI2 — Microscopy images of Halomonas sp. CUBES01. [file aem.00603-24-s0002.zip › Microscopy/Fructose_2nd_0001.jpg]

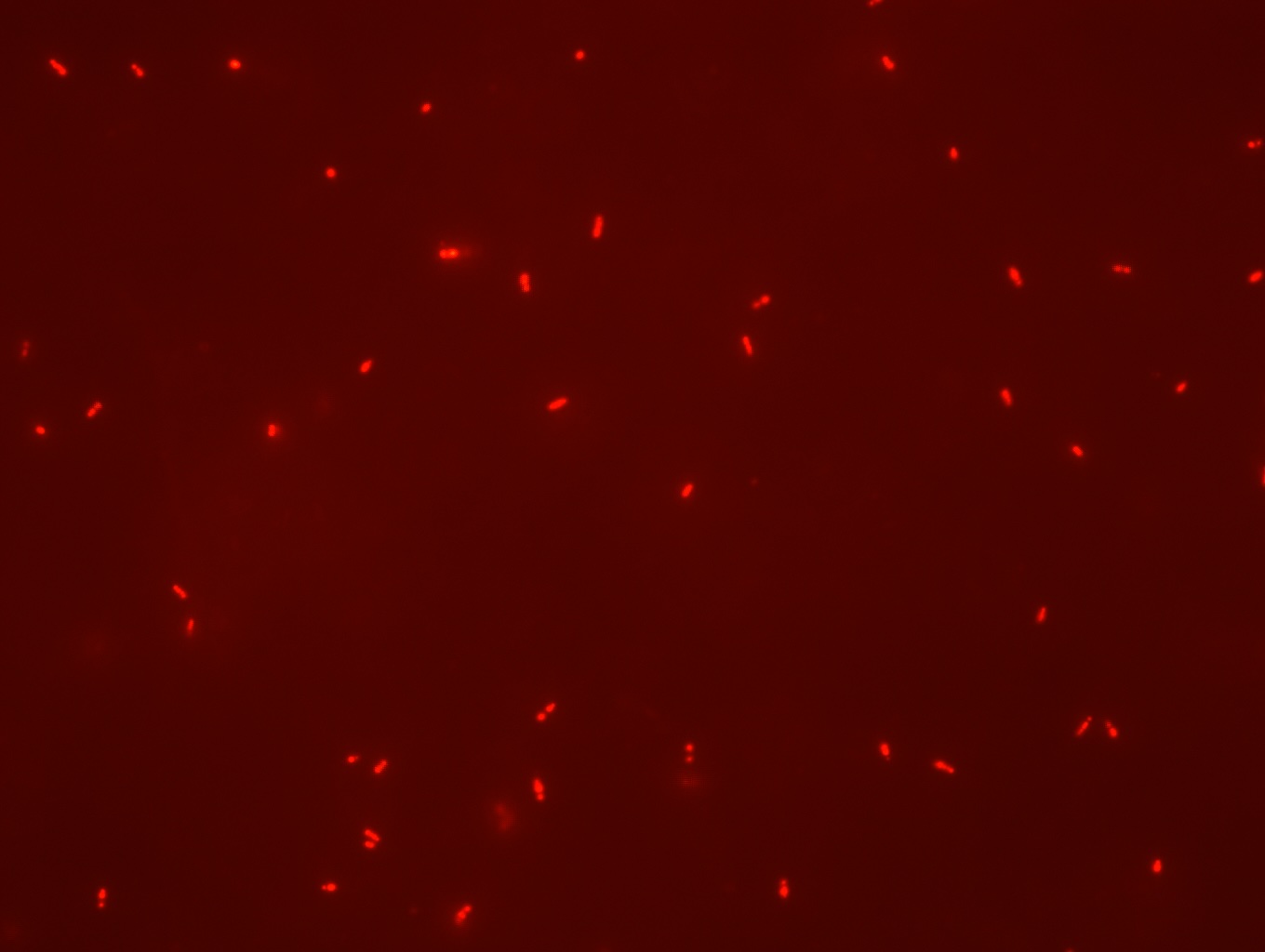

Supplement: File SI2 — Microscopy images of Halomonas sp. CUBES01. [file aem.00603-24-s0002.zip › Microscopy/Glucose_2nd_0002.jpg]

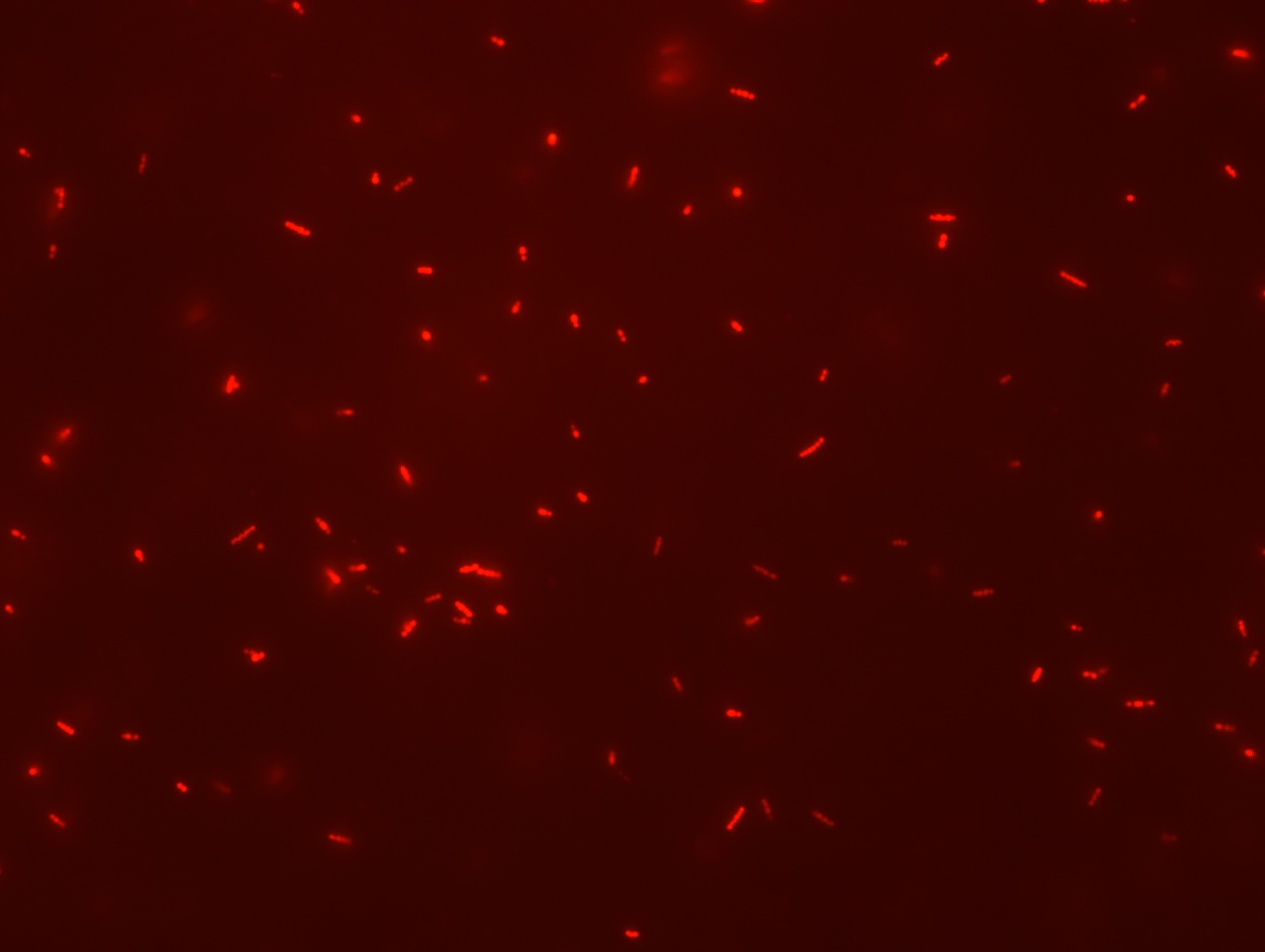

Supplement: File SI2 — Microscopy images of Halomonas sp. CUBES01. [file aem.00603-24-s0002.zip › Microscopy/Glucose_3rd_0002.jpg]

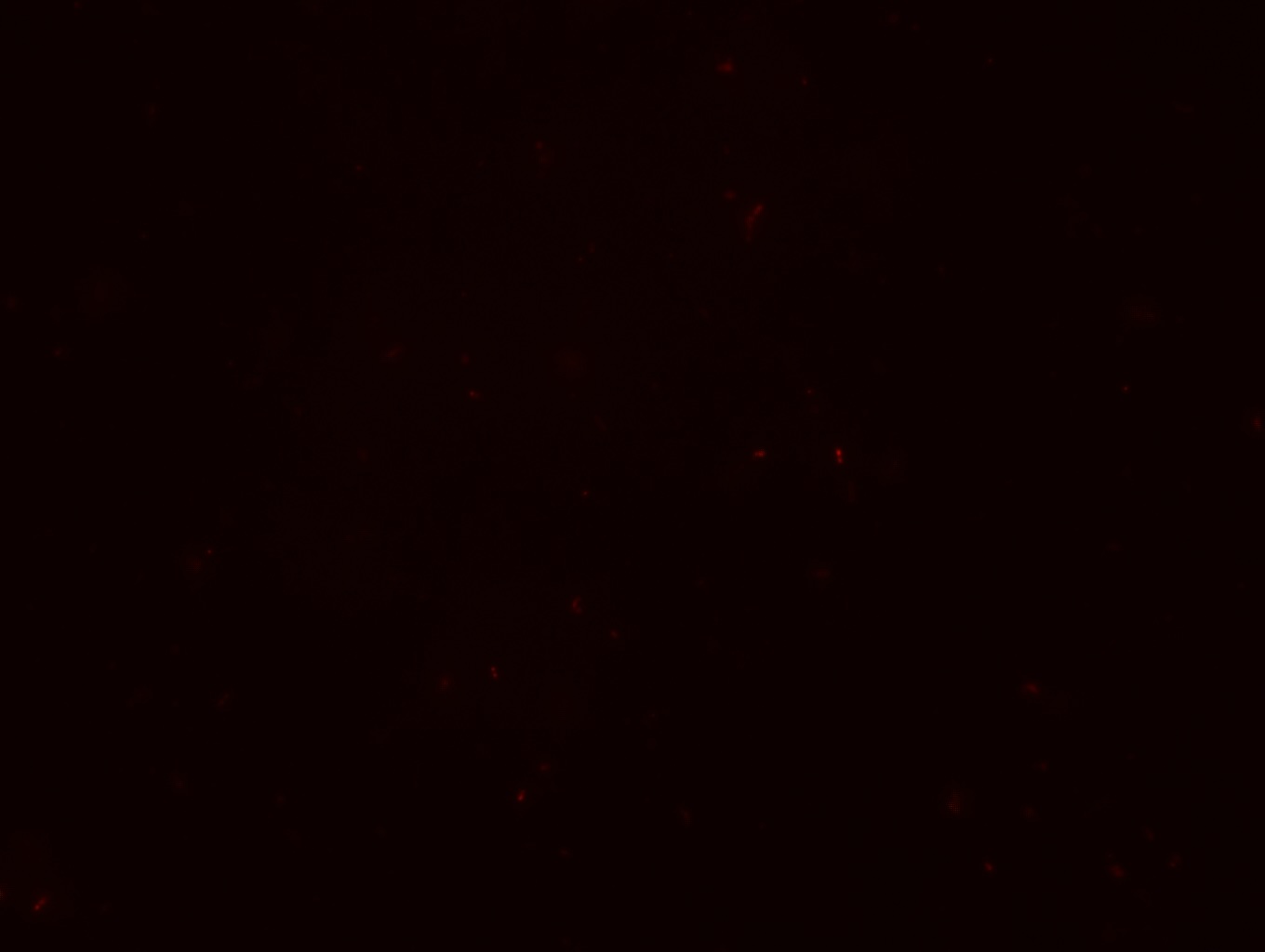

Supplement: File SI2 — Microscopy images of Halomonas sp. CUBES01. [file aem.00603-24-s0002.zip › Microscopy/Fructose_2nd_0003.jpg]

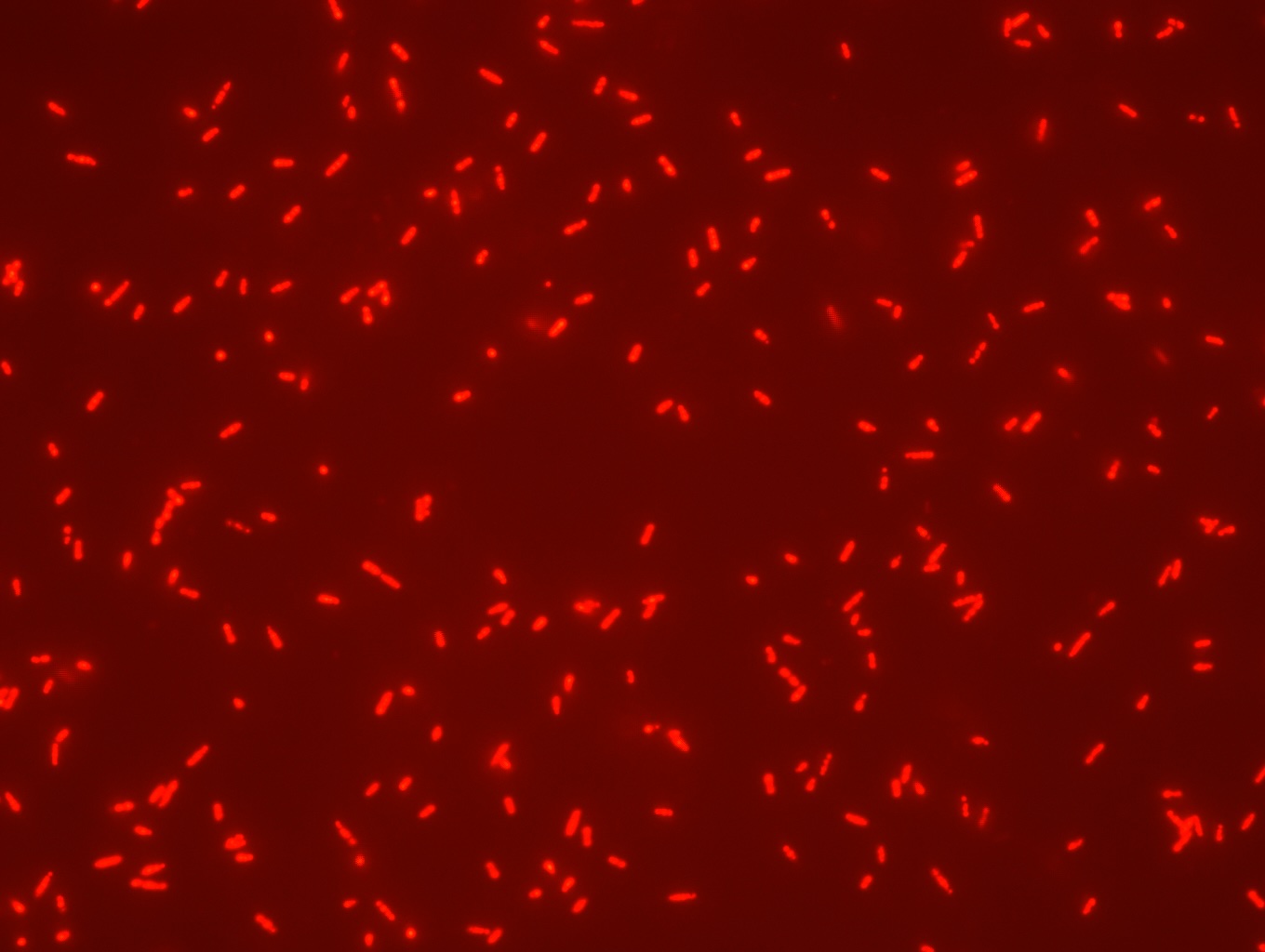

Supplement: File SI2 — Microscopy images of Halomonas sp. CUBES01. [file aem.00603-24-s0002.zip › Microscopy/Acetate_3rd_0001.jpg]

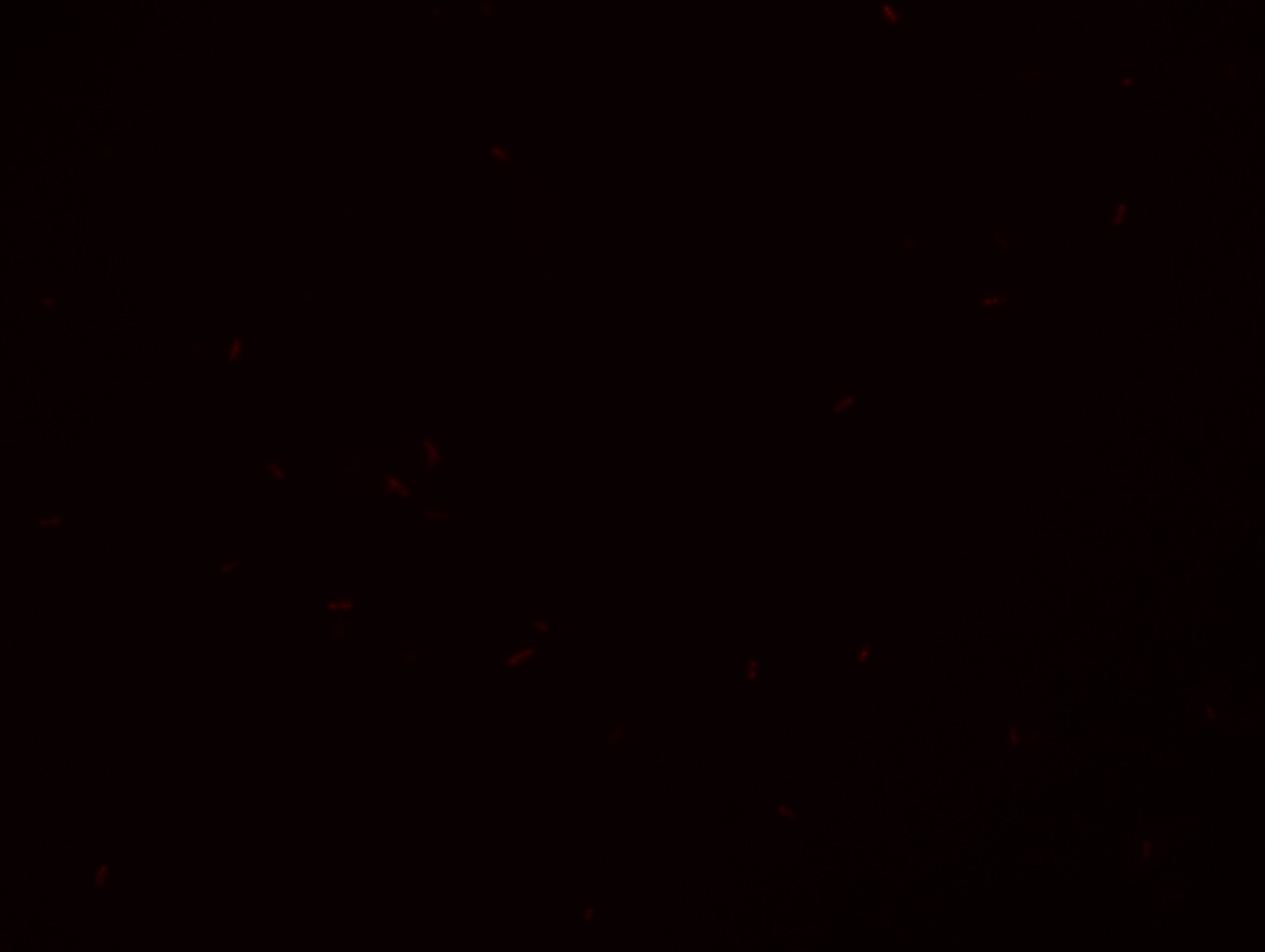

Supplement: File SI2 — Microscopy images of Halomonas sp. CUBES01. [file aem.00603-24-s0002.zip › Microscopy/Glucosamine_1st_0001.jpg]

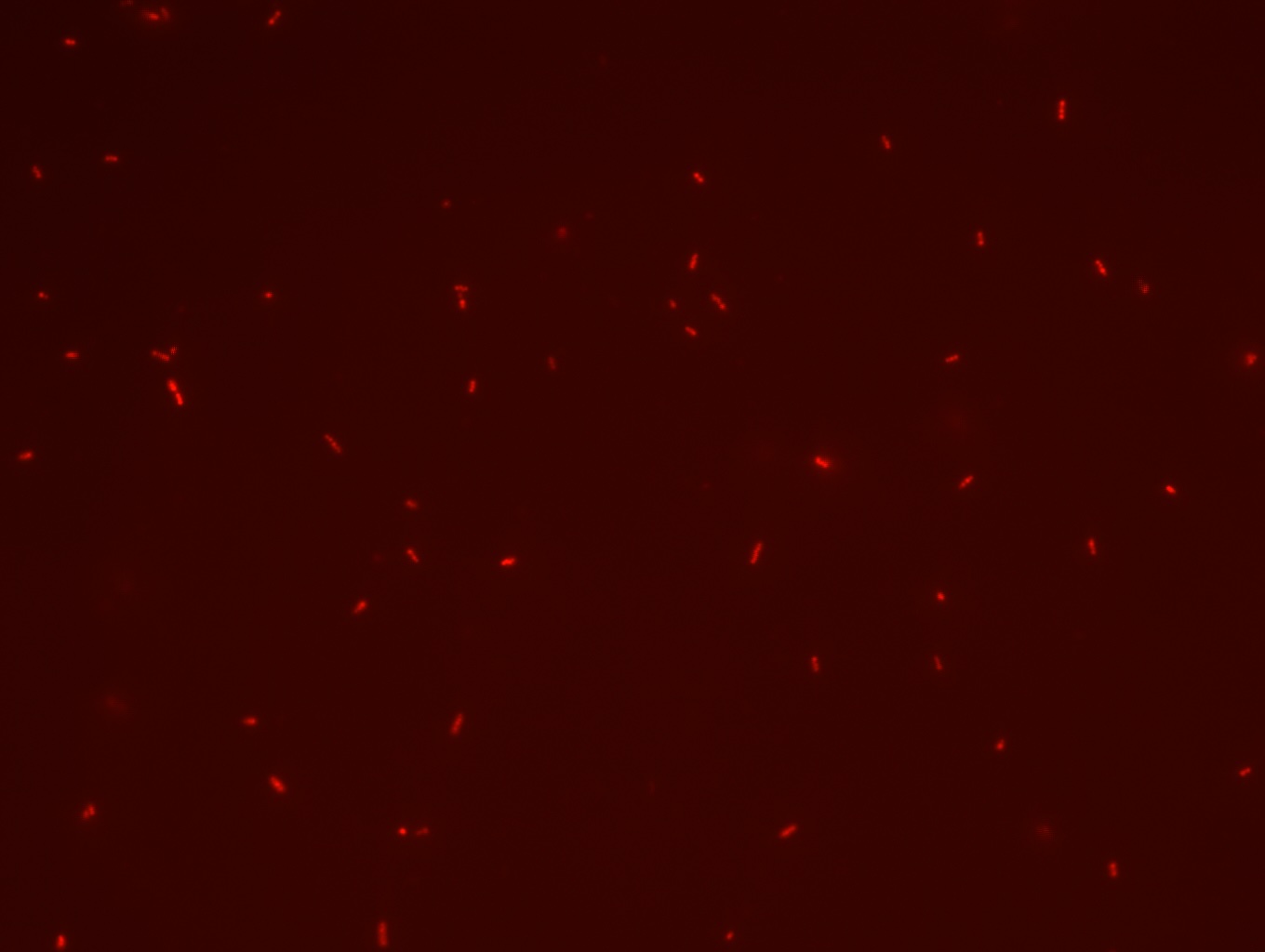

Supplement: File SI2 — Microscopy images of Halomonas sp. CUBES01. [file aem.00603-24-s0002.zip › Microscopy/Acetate_2nd_0003.jpg]

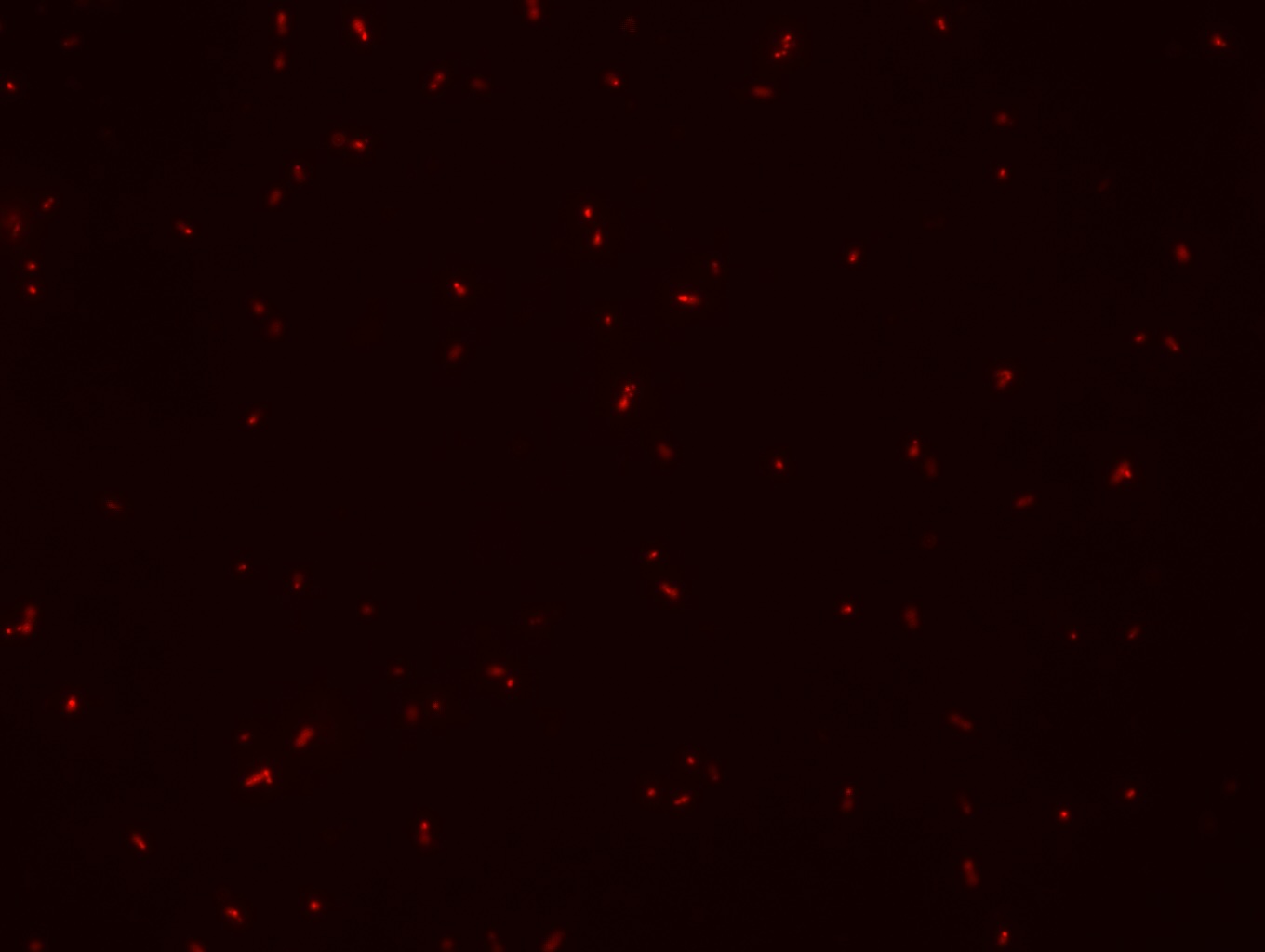

Supplement: File SI2 — Microscopy images of Halomonas sp. CUBES01. [file aem.00603-24-s0002.zip › Microscopy/Glycerol_1st_0001.jpg]

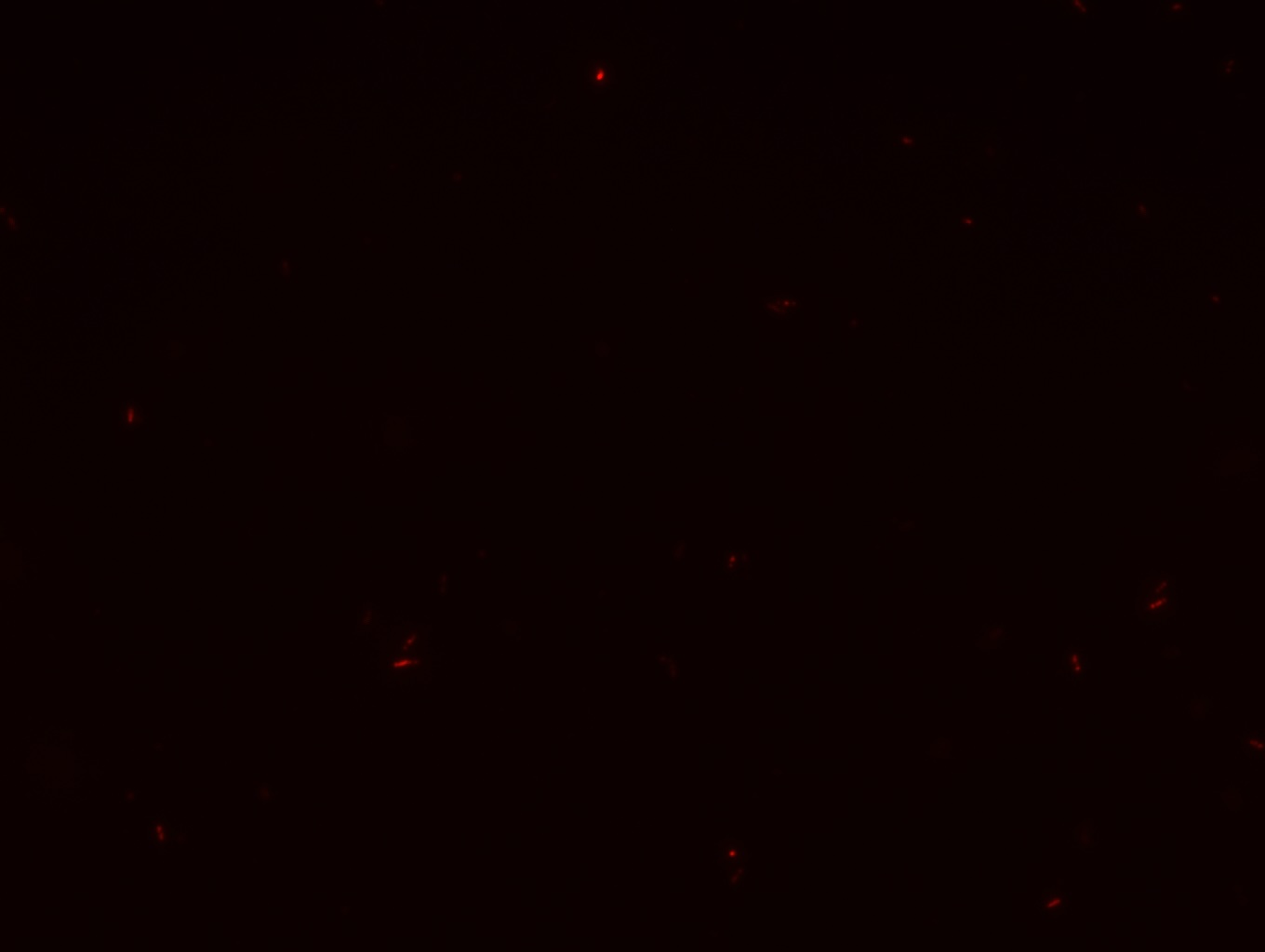

Supplement: File SI2 — Microscopy images of Halomonas sp. CUBES01. [file aem.00603-24-s0002.zip › Microscopy/Fructose_3rd_0001.jpg]

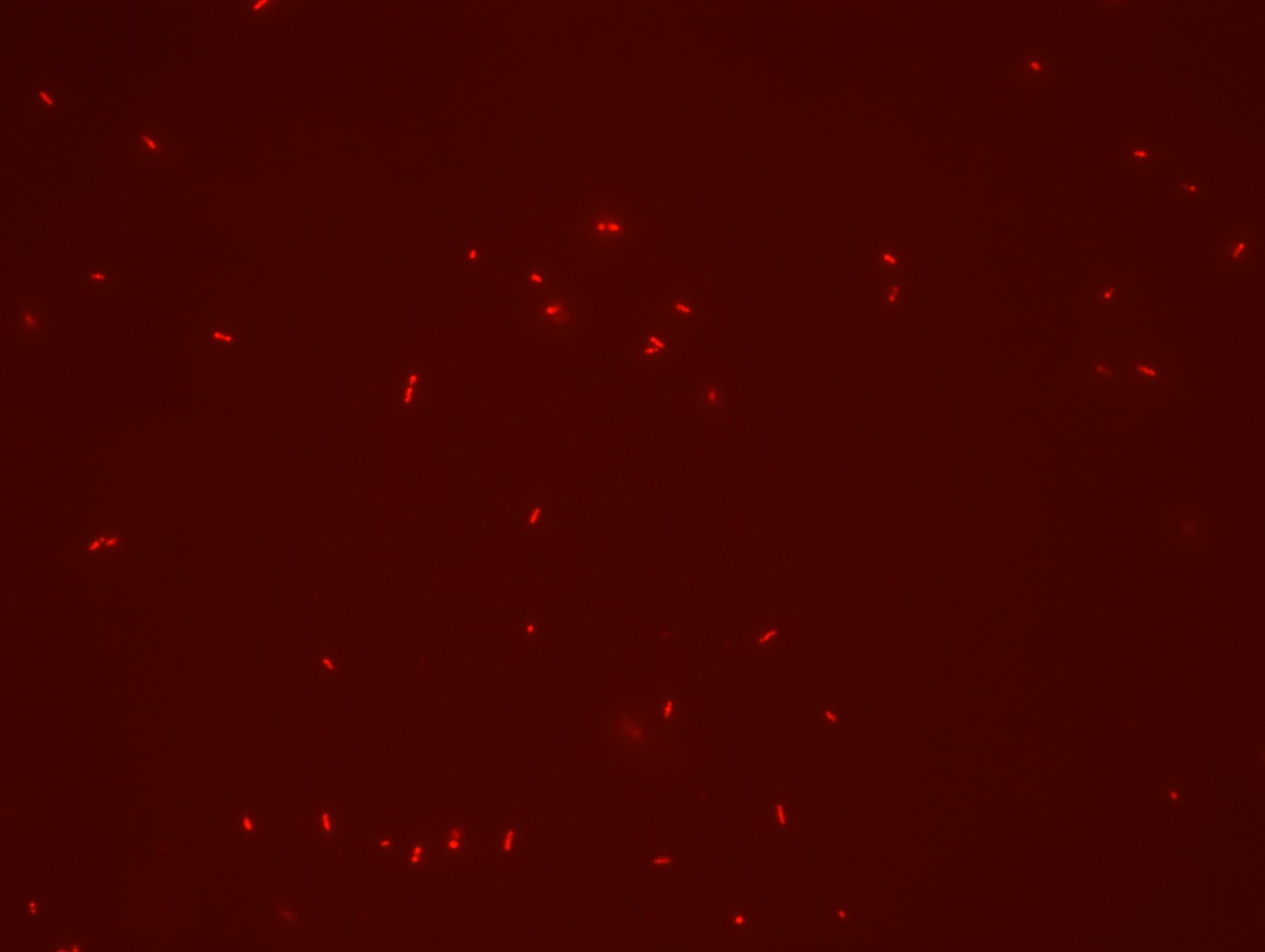

Supplement: File SI2 — Microscopy images of Halomonas sp. CUBES01. [file aem.00603-24-s0002.zip › Microscopy/Acetate_2nd_0002.jpg]

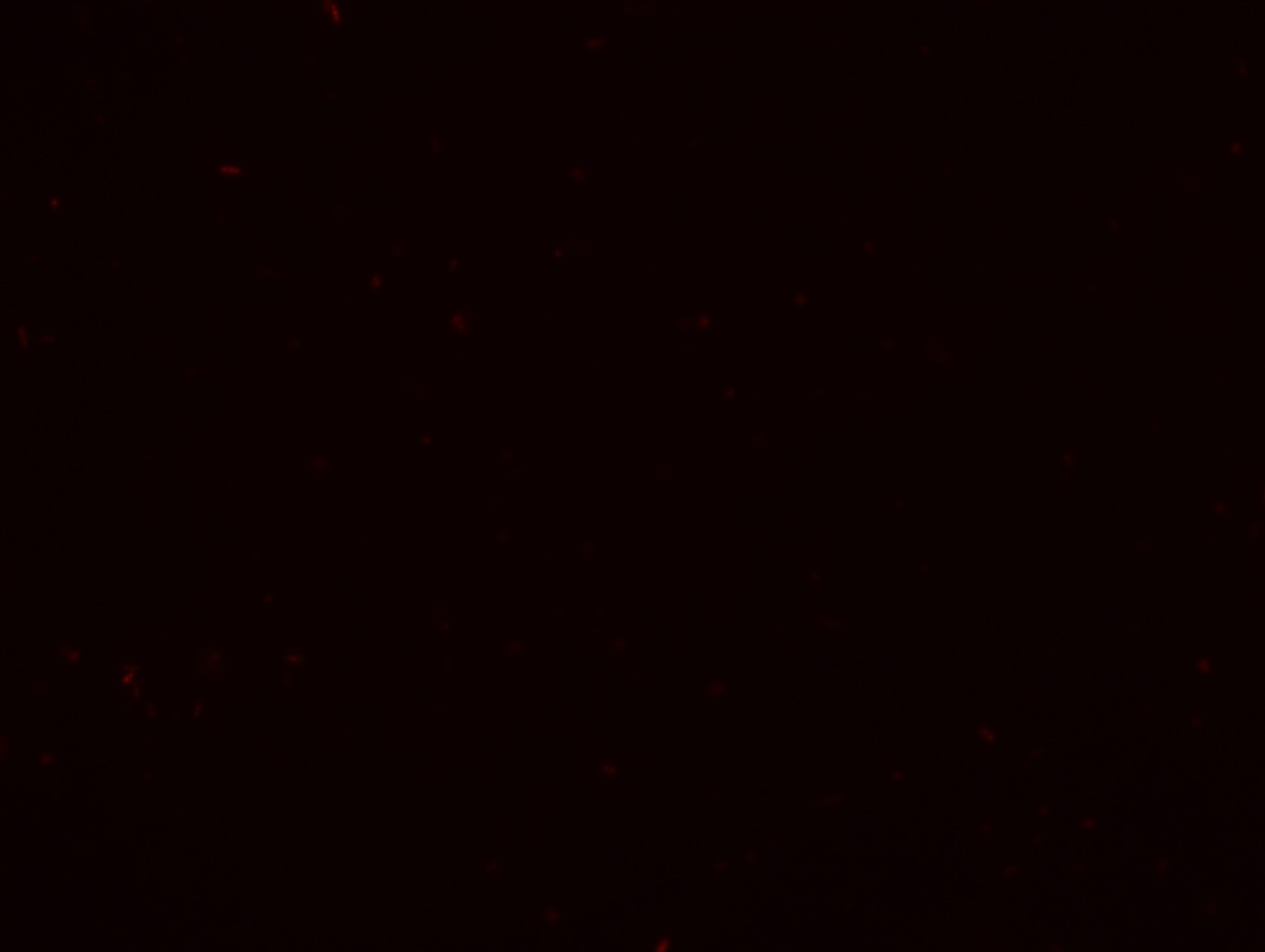

Supplement: File SI2 — Microscopy images of Halomonas sp. CUBES01. [file aem.00603-24-s0002.zip › Microscopy/Fructose_2nd_0002.jpg]

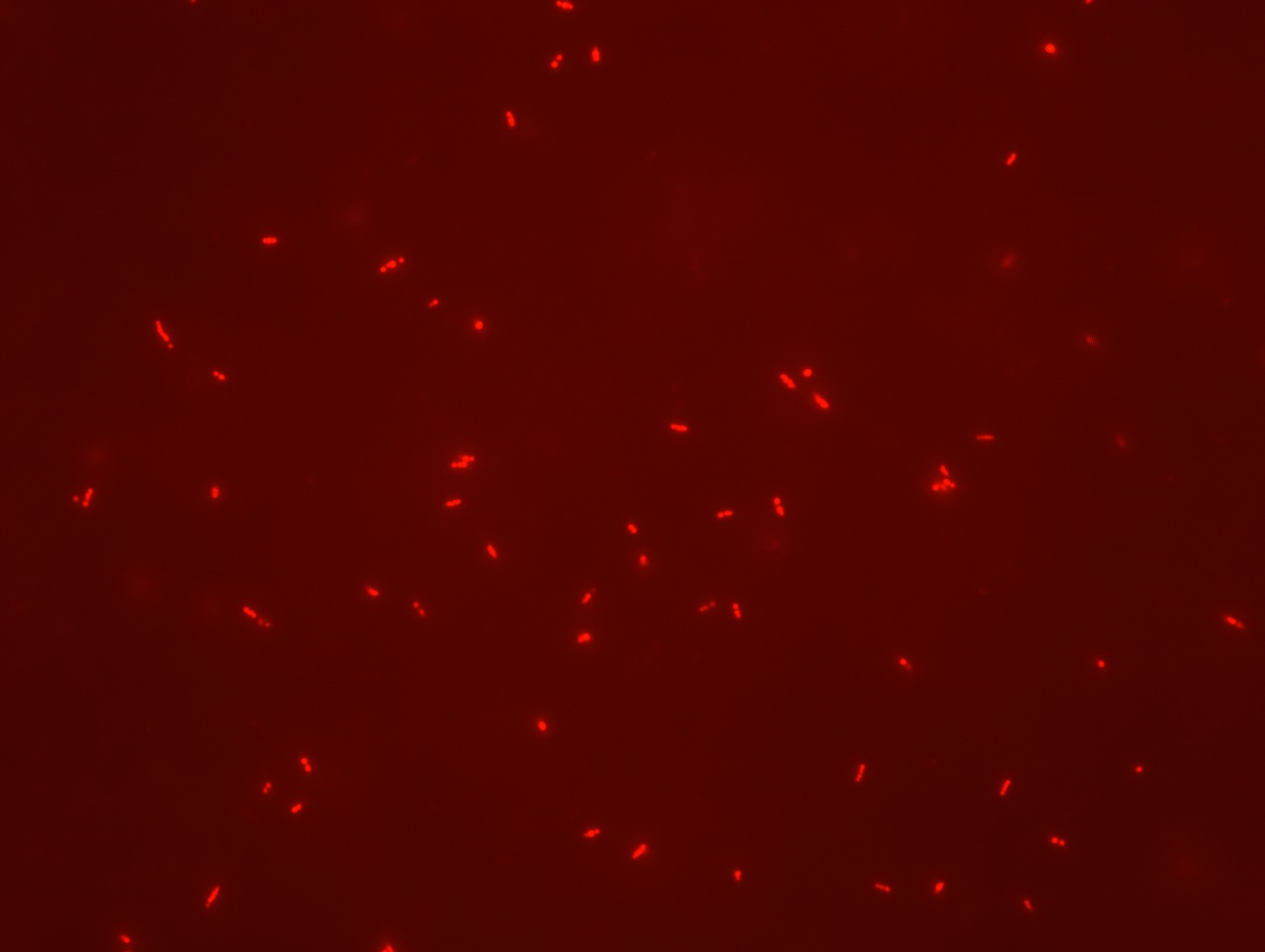

Supplement: File SI2 — Microscopy images of Halomonas sp. CUBES01. [file aem.00603-24-s0002.zip › Microscopy/Glucose_2nd_0001.jpg]

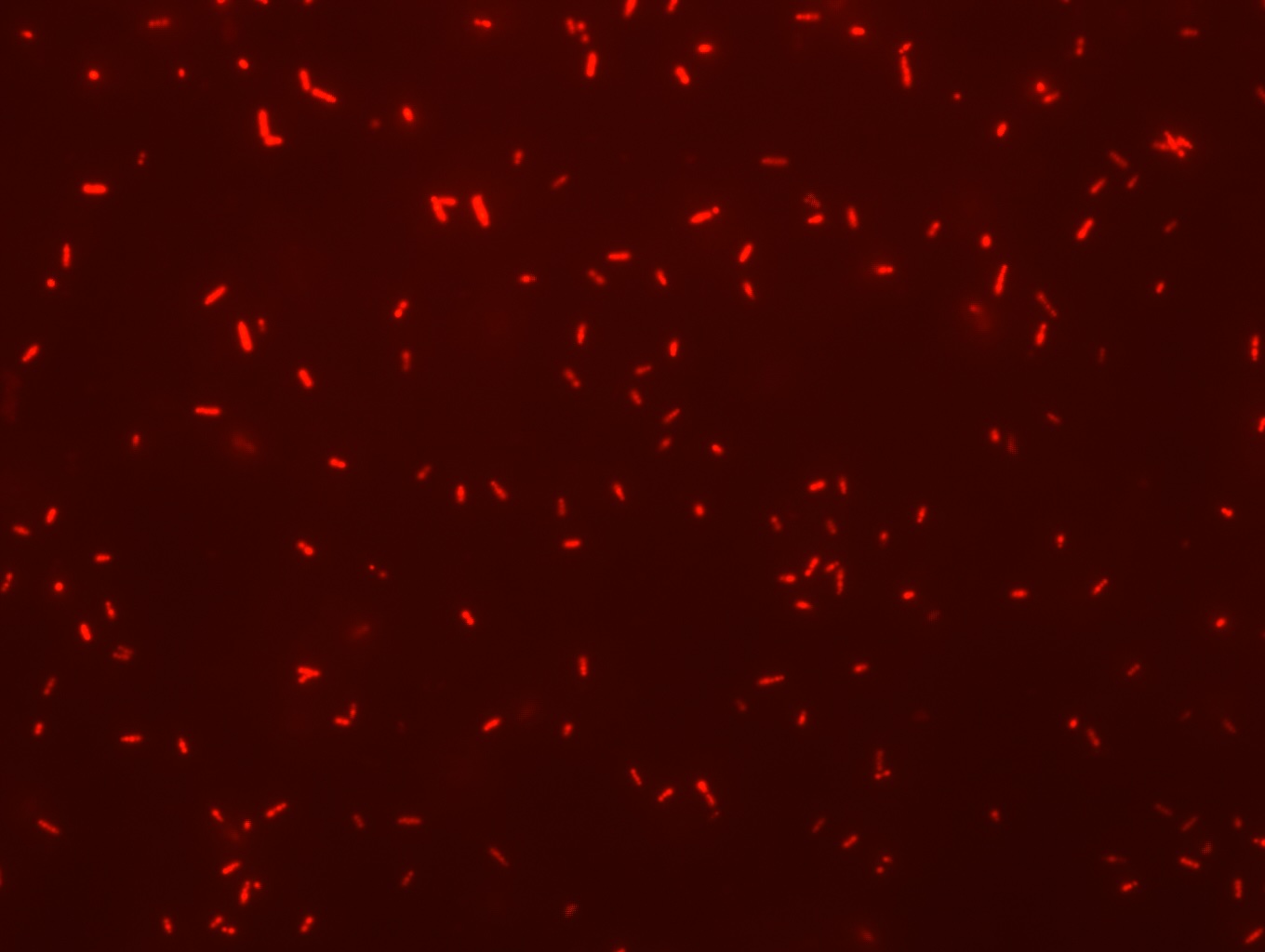

Supplement: File SI2 — Microscopy images of Halomonas sp. CUBES01. [file aem.00603-24-s0002.zip › Microscopy/Glucose_3rd_0003.jpg]

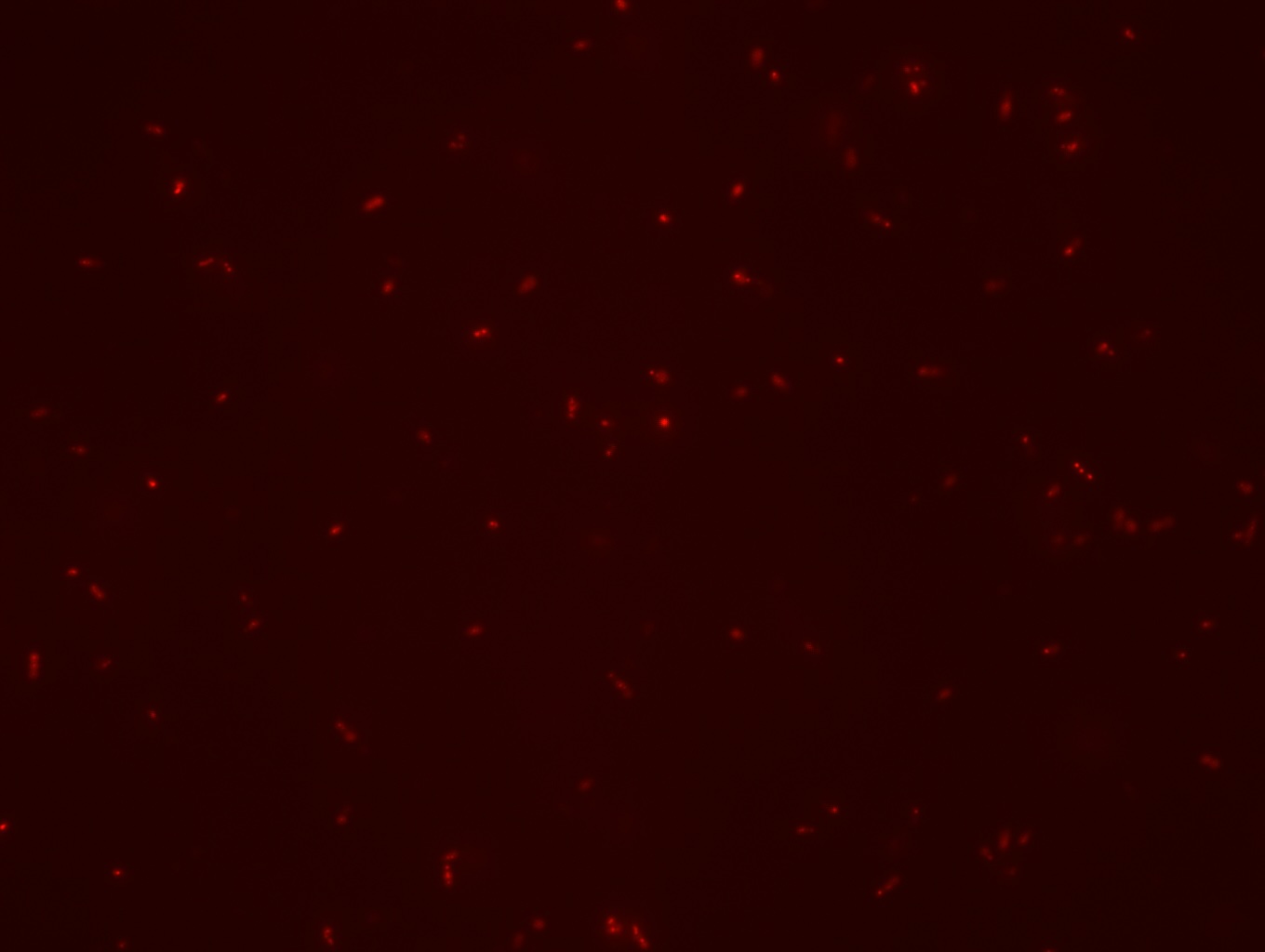

Supplement: File SI2 — Microscopy images of Halomonas sp. CUBES01. [file aem.00603-24-s0002.zip › Microscopy/Glucose_1st_0002.jpg]

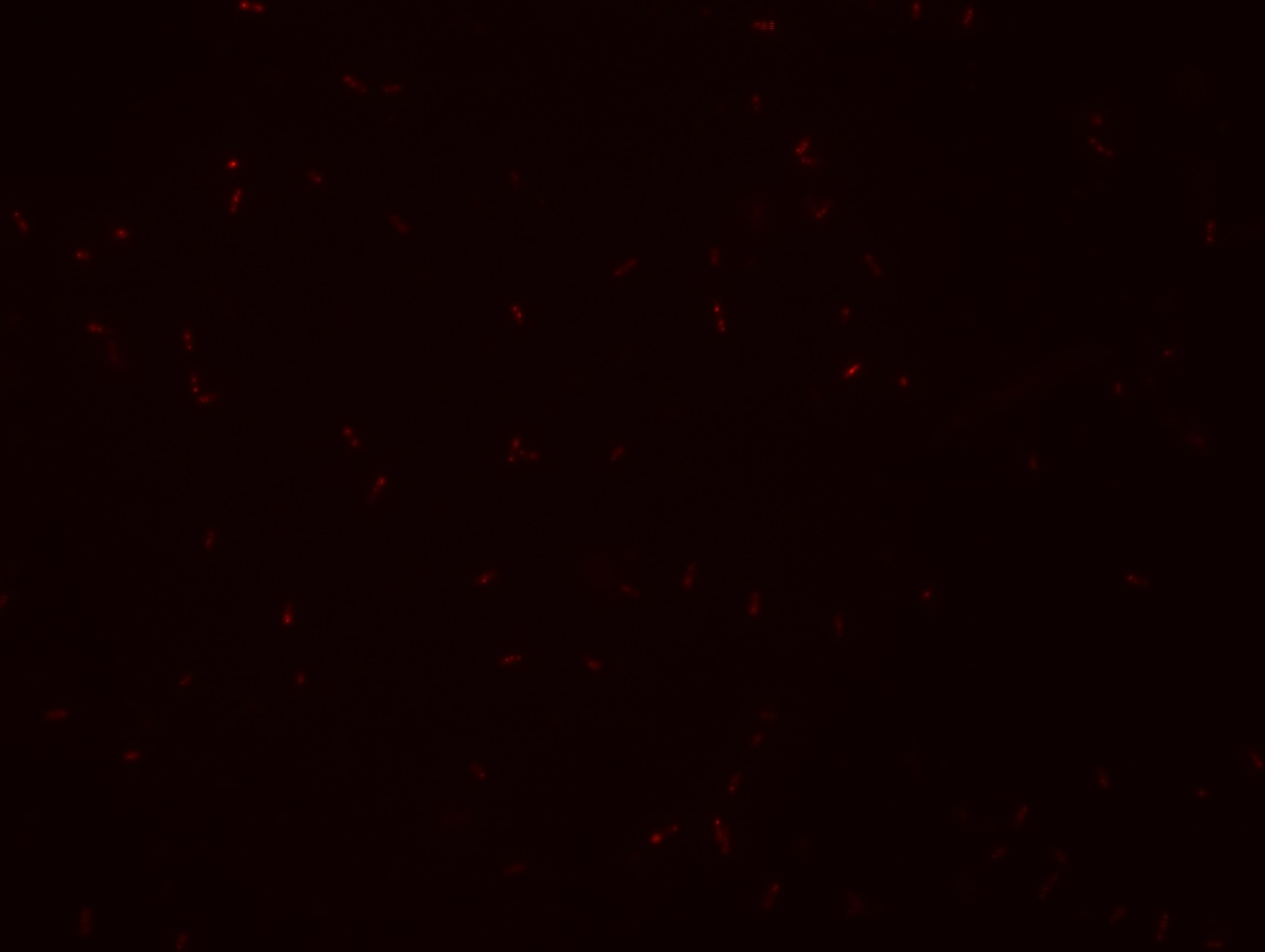

Supplement: File SI2 — Microscopy images of Halomonas sp. CUBES01. [file aem.00603-24-s0002.zip › Microscopy/Acetate_1st_0001.jpg]

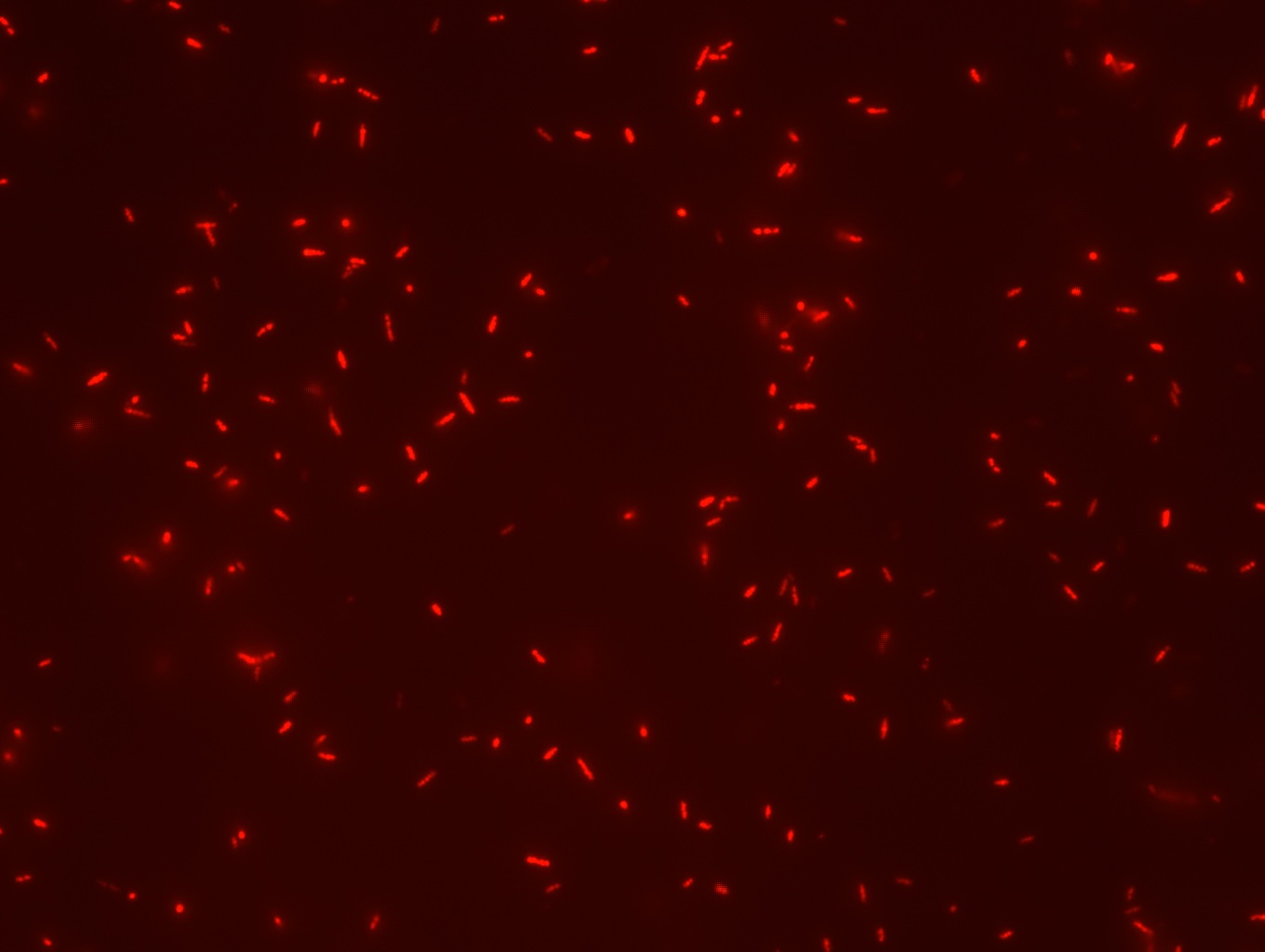

Supplement: File SI2 — Microscopy images of Halomonas sp. CUBES01. [file aem.00603-24-s0002.zip › Microscopy/Glycerol_2nd_0003.jpg]

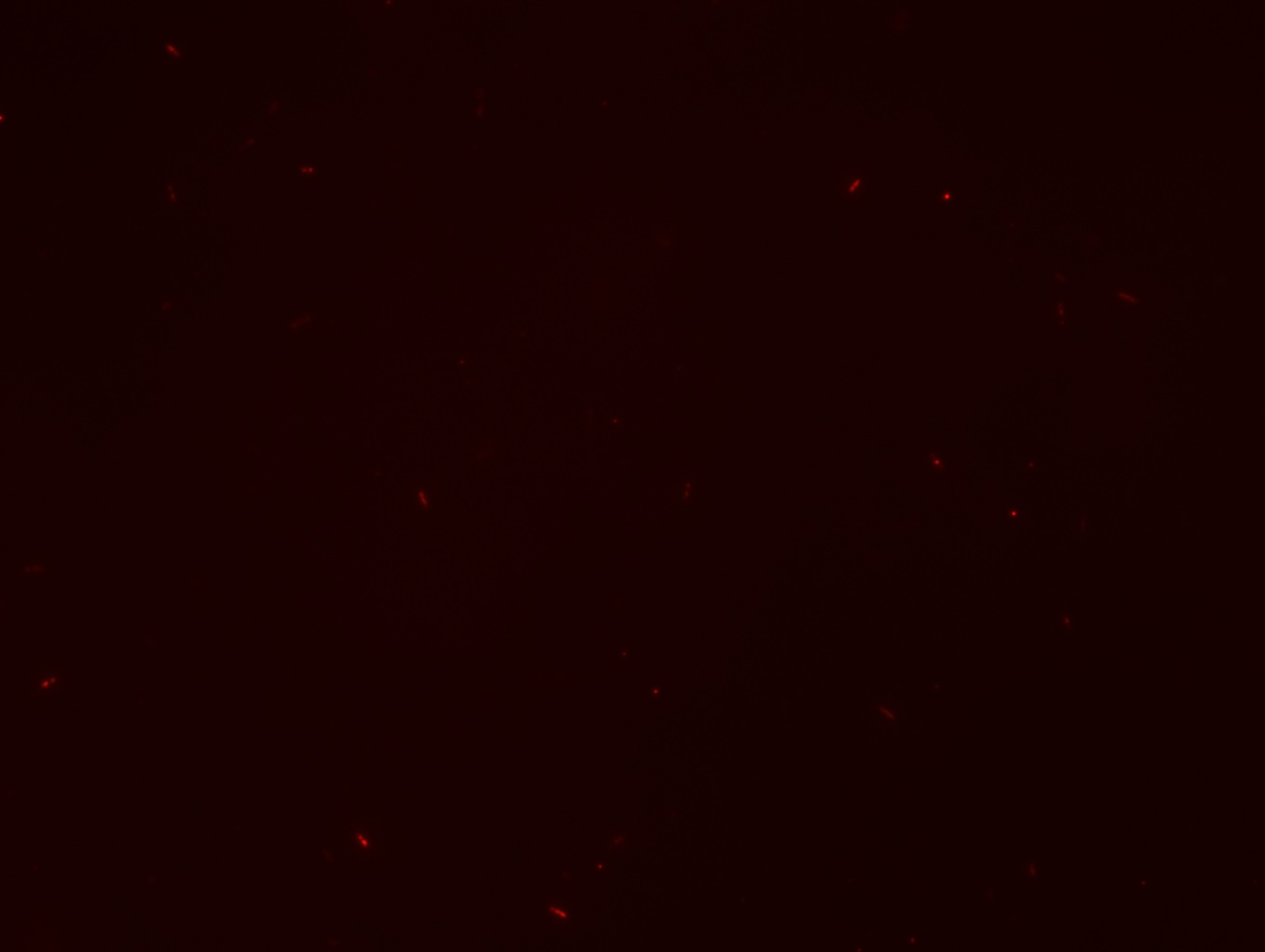

Supplement: File SI2 — Microscopy images of Halomonas sp. CUBES01. [file aem.00603-24-s0002.zip › Microscopy/Glucosamine_3rd_0001.jpg]

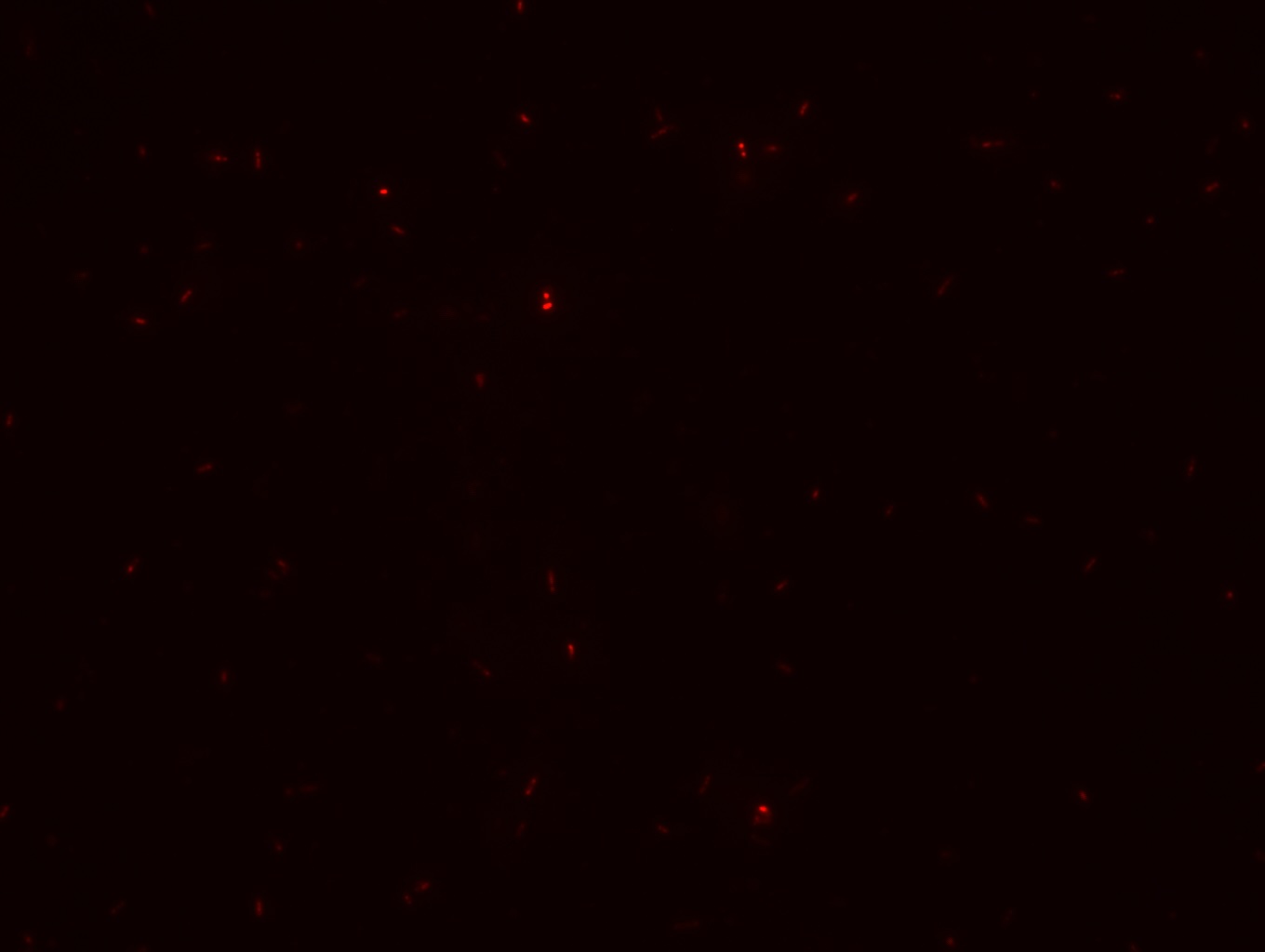

Supplement: File SI2 — Microscopy images of Halomonas sp. CUBES01. [file aem.00603-24-s0002.zip › Microscopy/Fructose_1st_0001.jpg]

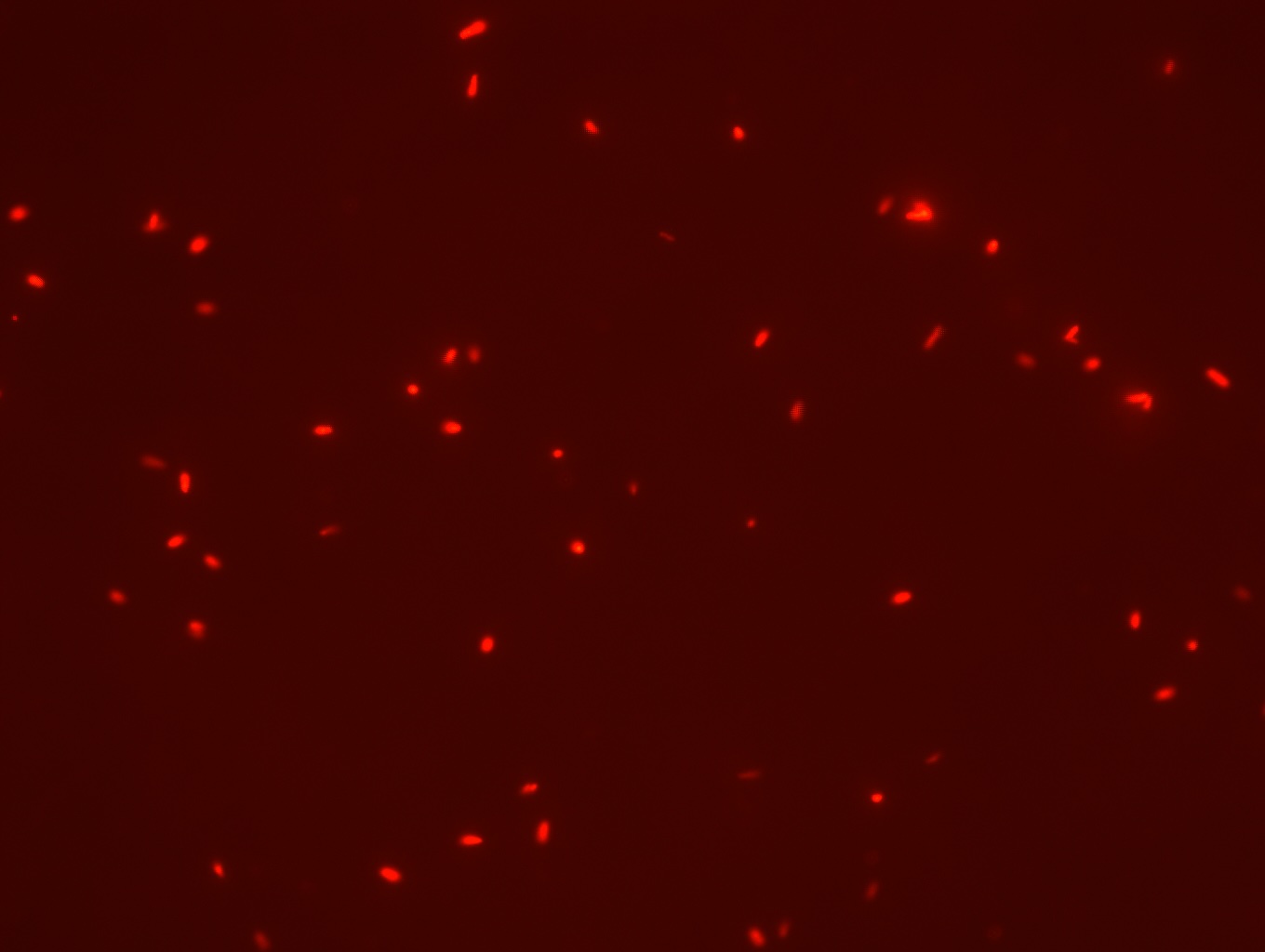

Supplement: File SI2 — Microscopy images of Halomonas sp. CUBES01. [file aem.00603-24-s0002.zip › Microscopy/Glycerol_3rd_0001.jpg]

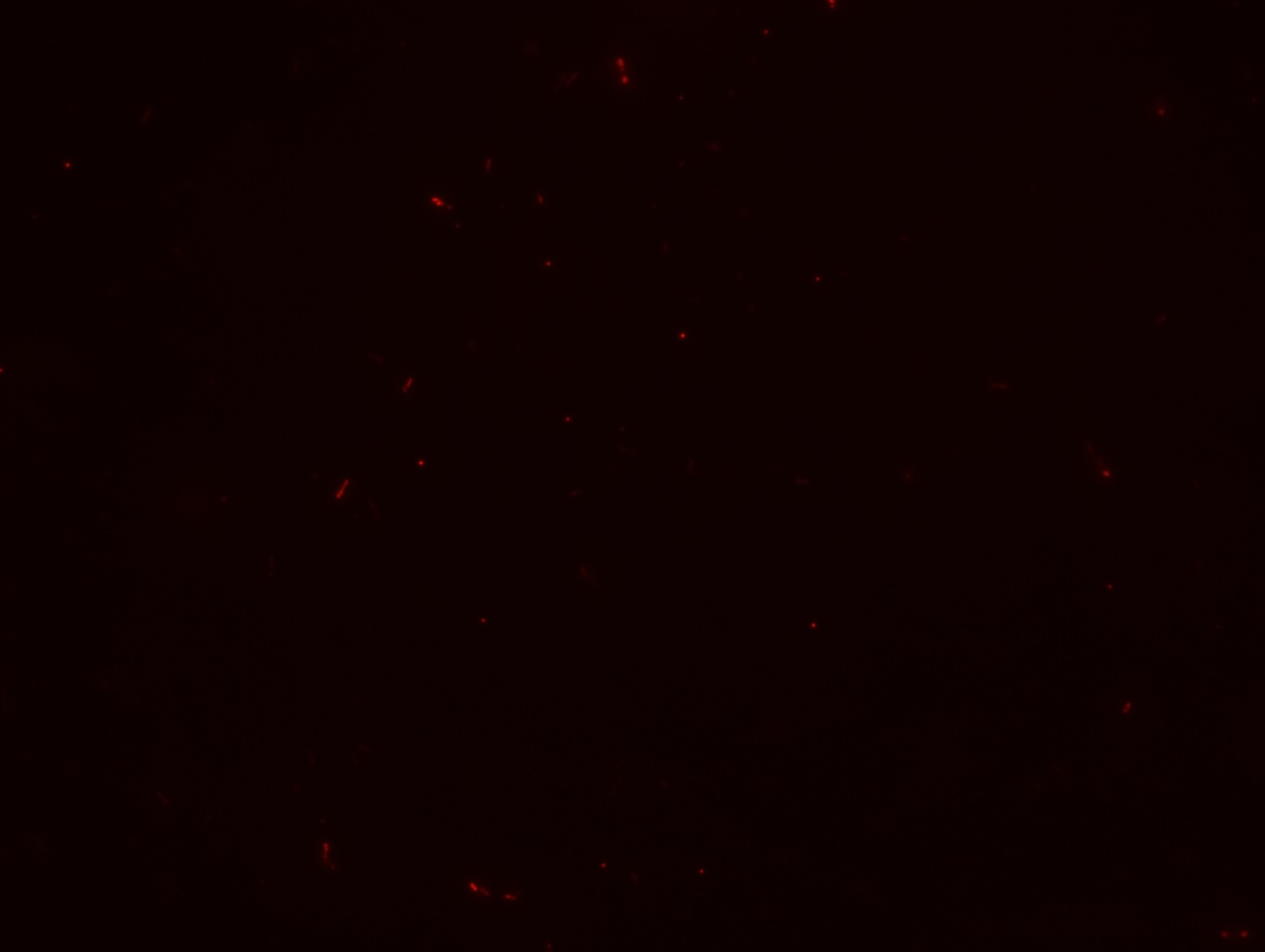

Supplement: File SI2 — Microscopy images of Halomonas sp. CUBES01. [file aem.00603-24-s0002.zip › Microscopy/Glucosamine_2nd_0003.jpg]

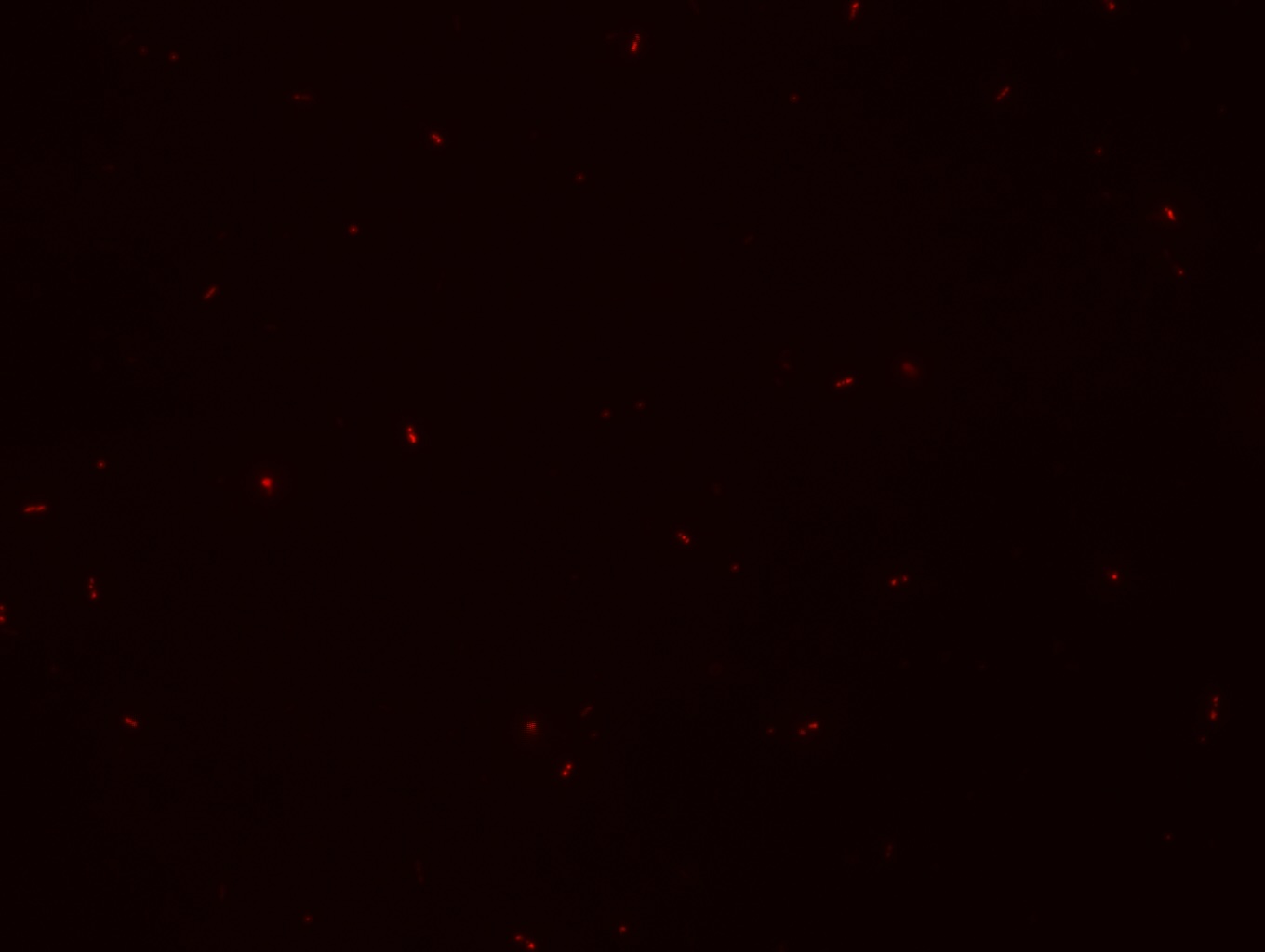

Supplement: File SI2 — Microscopy images of Halomonas sp. CUBES01. [file aem.00603-24-s0002.zip › Microscopy/Glucosamine_2nd_0002.jpg]

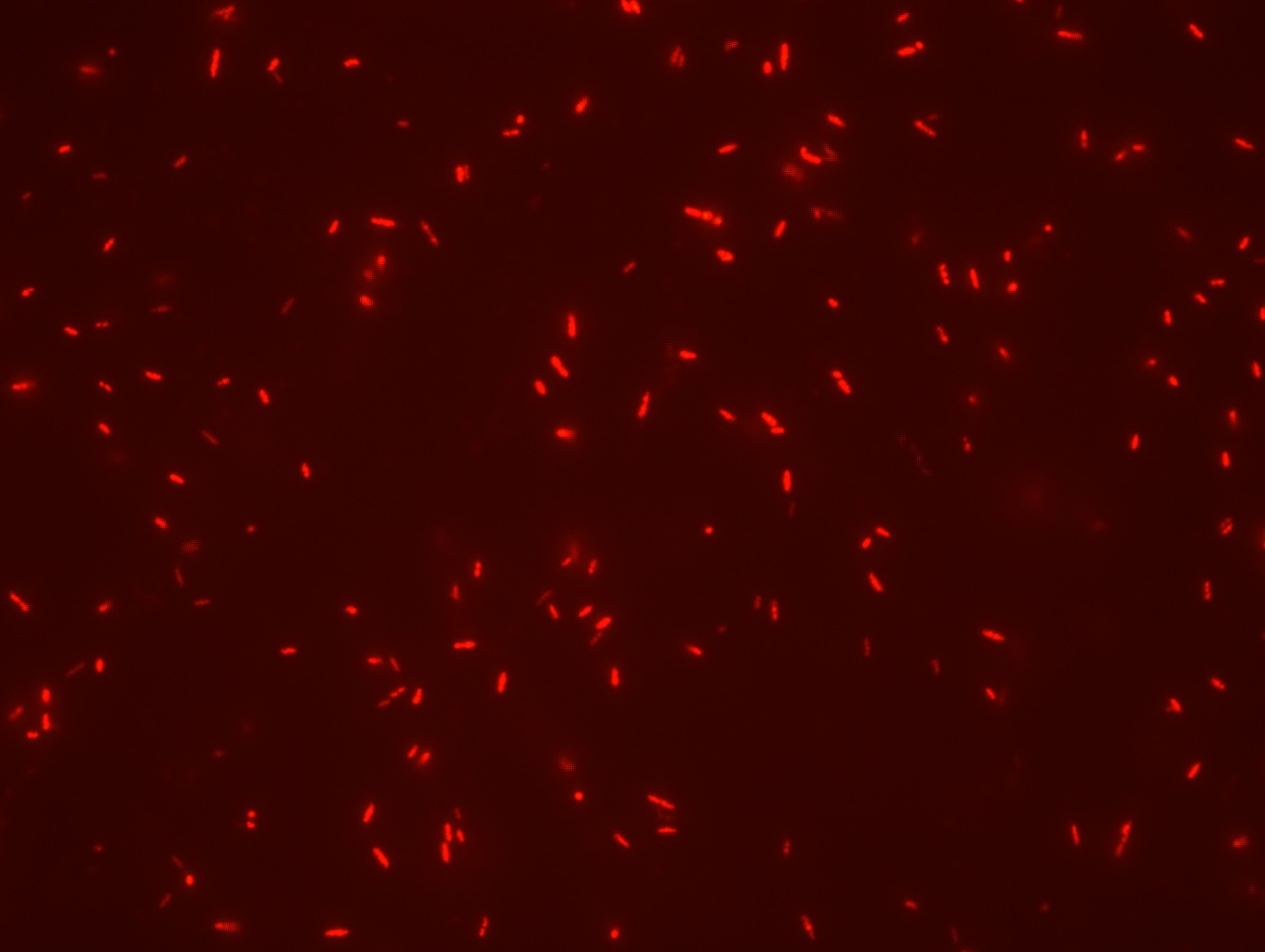

Supplement: File SI2 — Microscopy images of Halomonas sp. CUBES01. [file aem.00603-24-s0002.zip › Microscopy/Glycerol_2nd_0002.jpg]

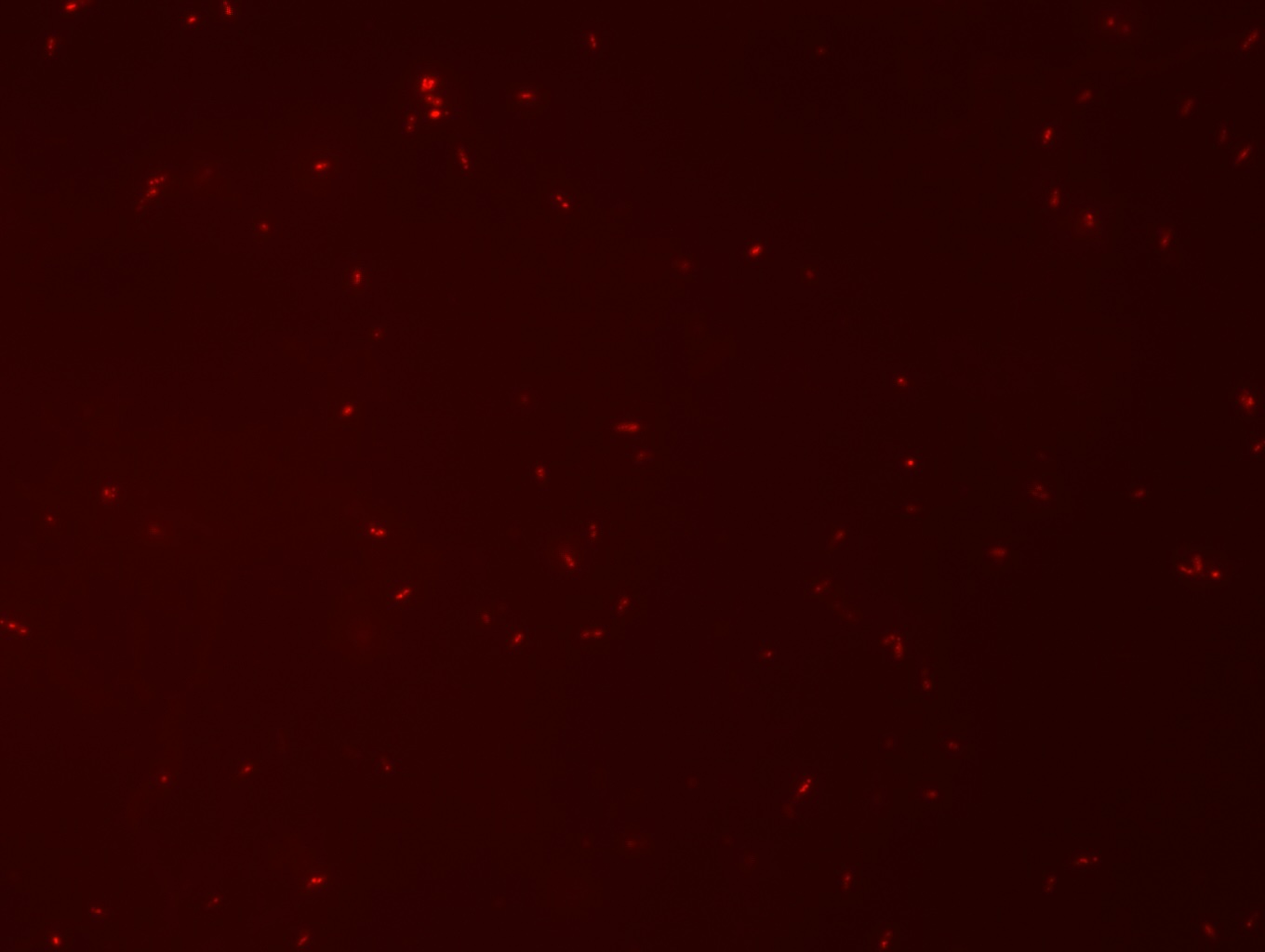

Supplement: File SI2 — Microscopy images of Halomonas sp. CUBES01. [file aem.00603-24-s0002.zip › Microscopy/Glucose_1st_0003.jpg]

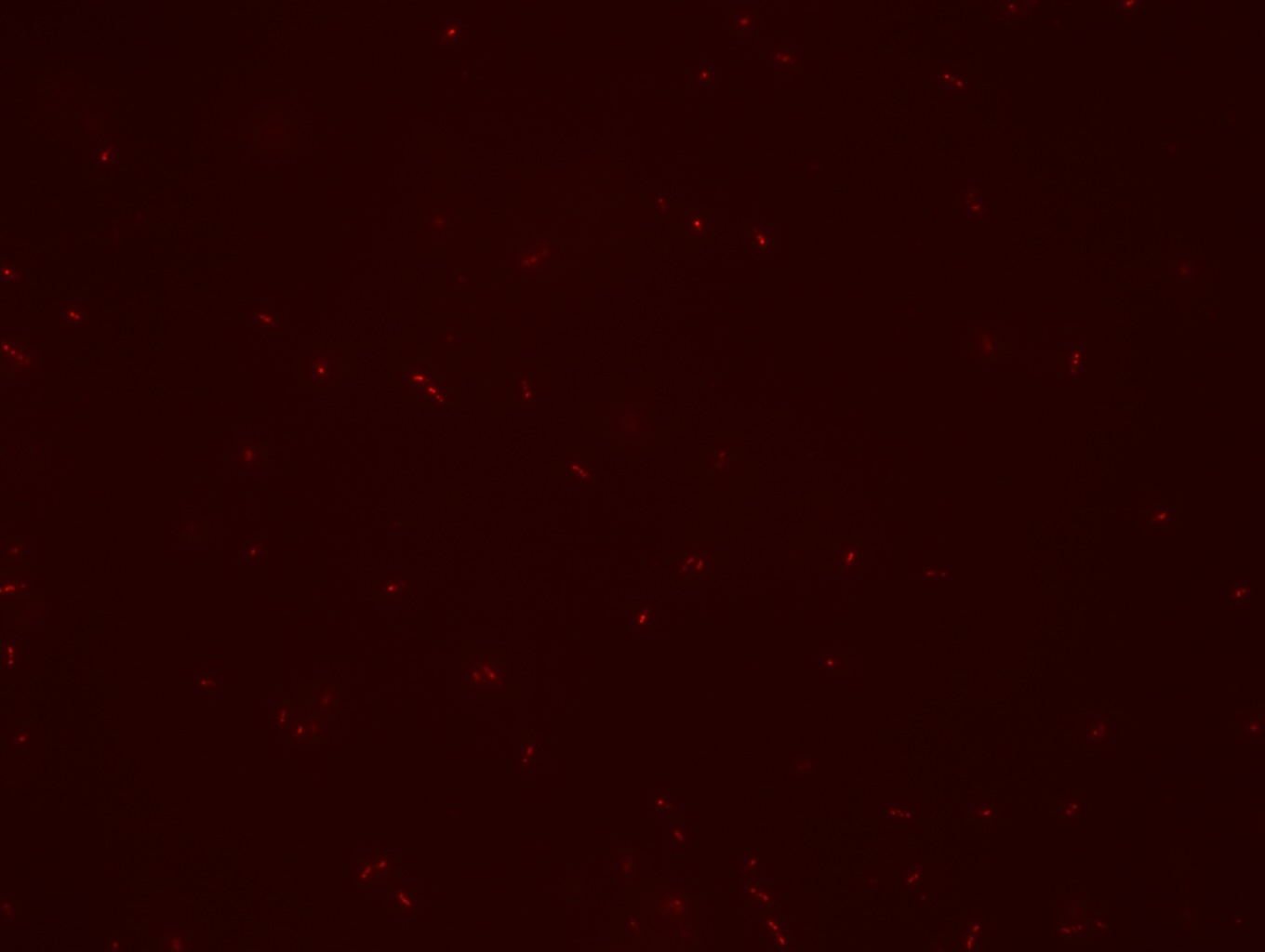

Supplement: File SI2 — Microscopy images of Halomonas sp. CUBES01. [file aem.00603-24-s0002.zip › Microscopy/Glucose_1st_0001.jpg]

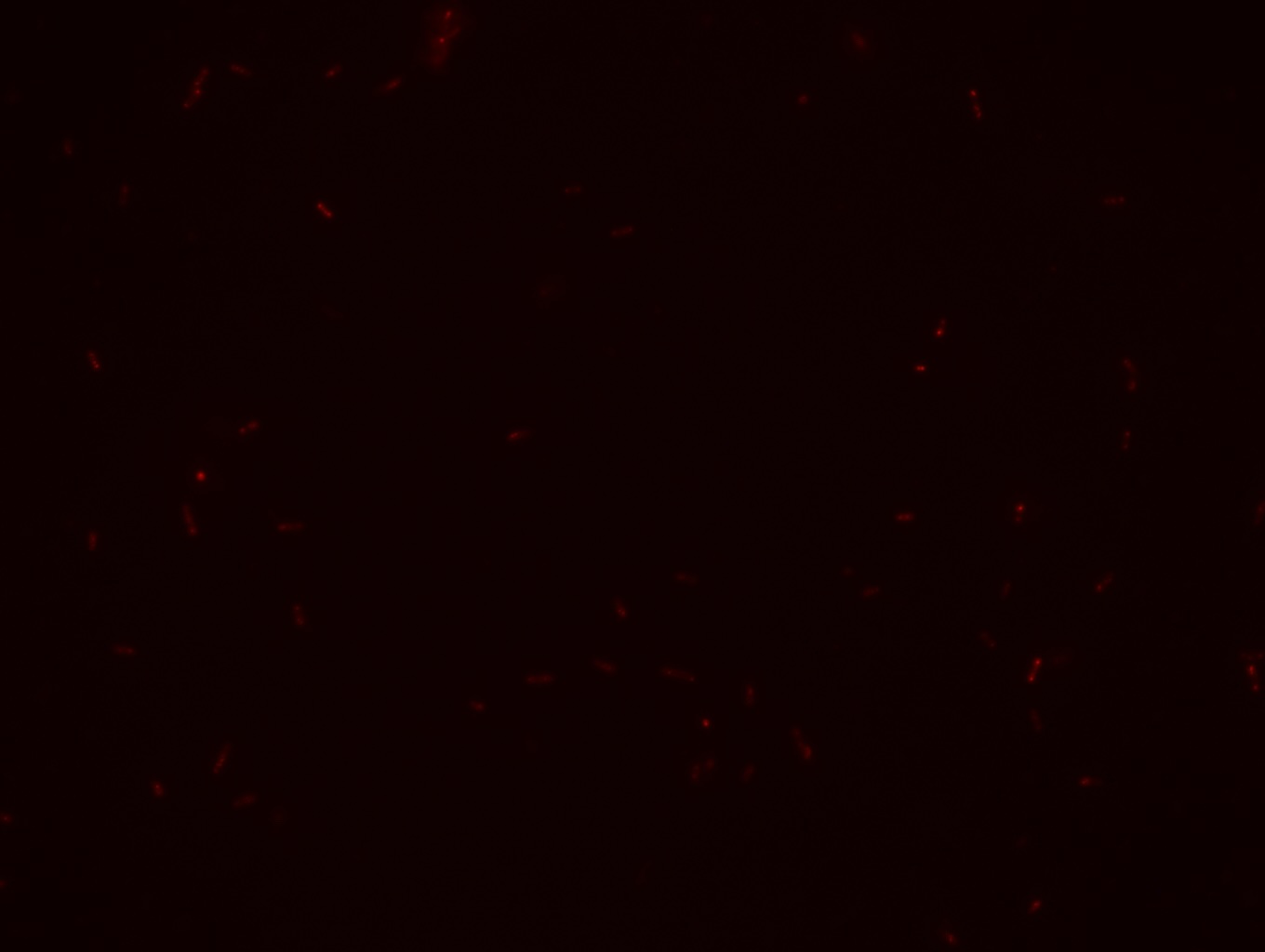

Supplement: File SI2 — Microscopy images of Halomonas sp. CUBES01. [file aem.00603-24-s0002.zip › Microscopy/Acetate_1st_0002.jpg]

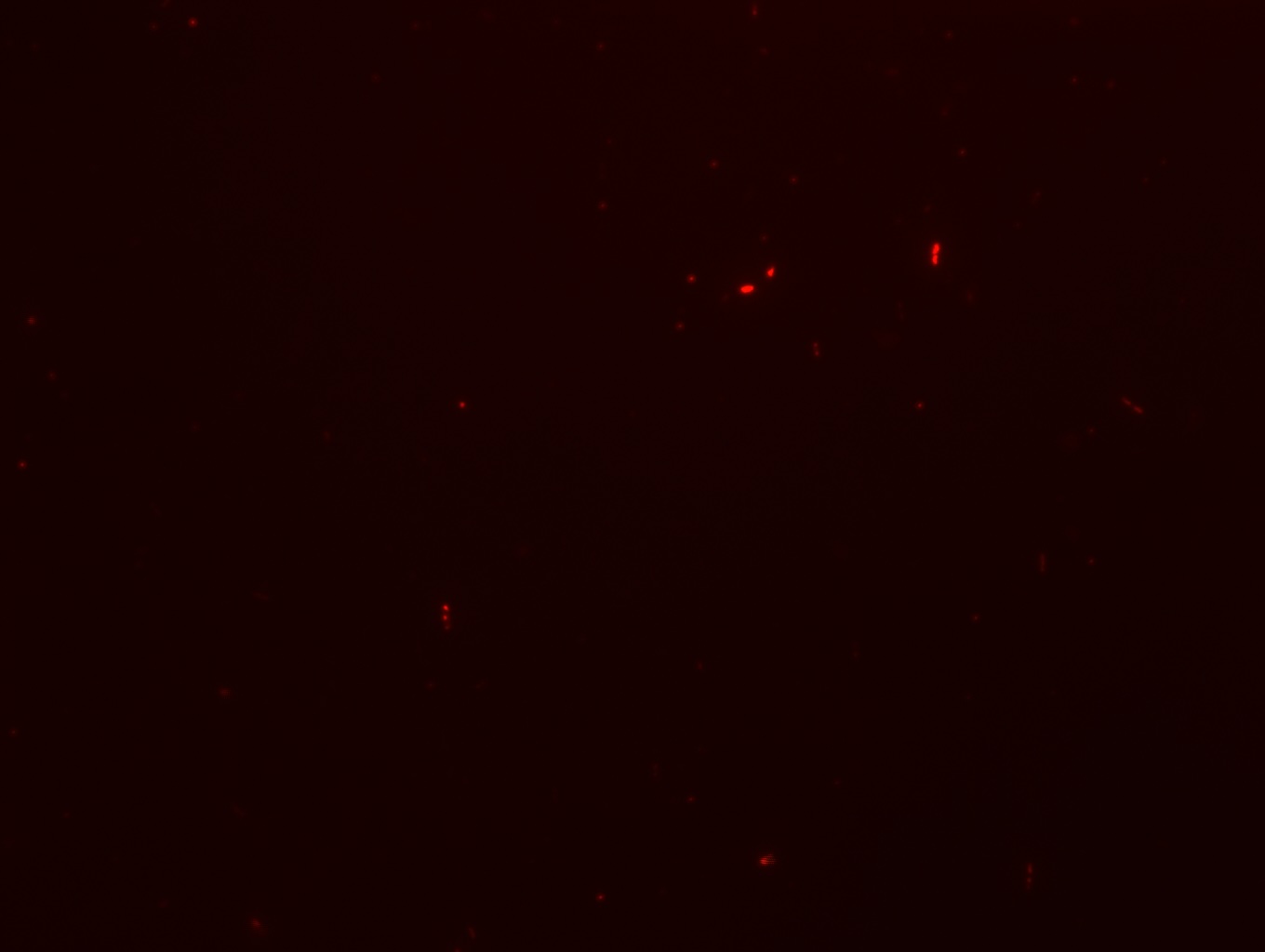

Supplement: File SI2 — Microscopy images of Halomonas sp. CUBES01. [file aem.00603-24-s0002.zip › Microscopy/Glucosamine_3rd_0002.jpg]

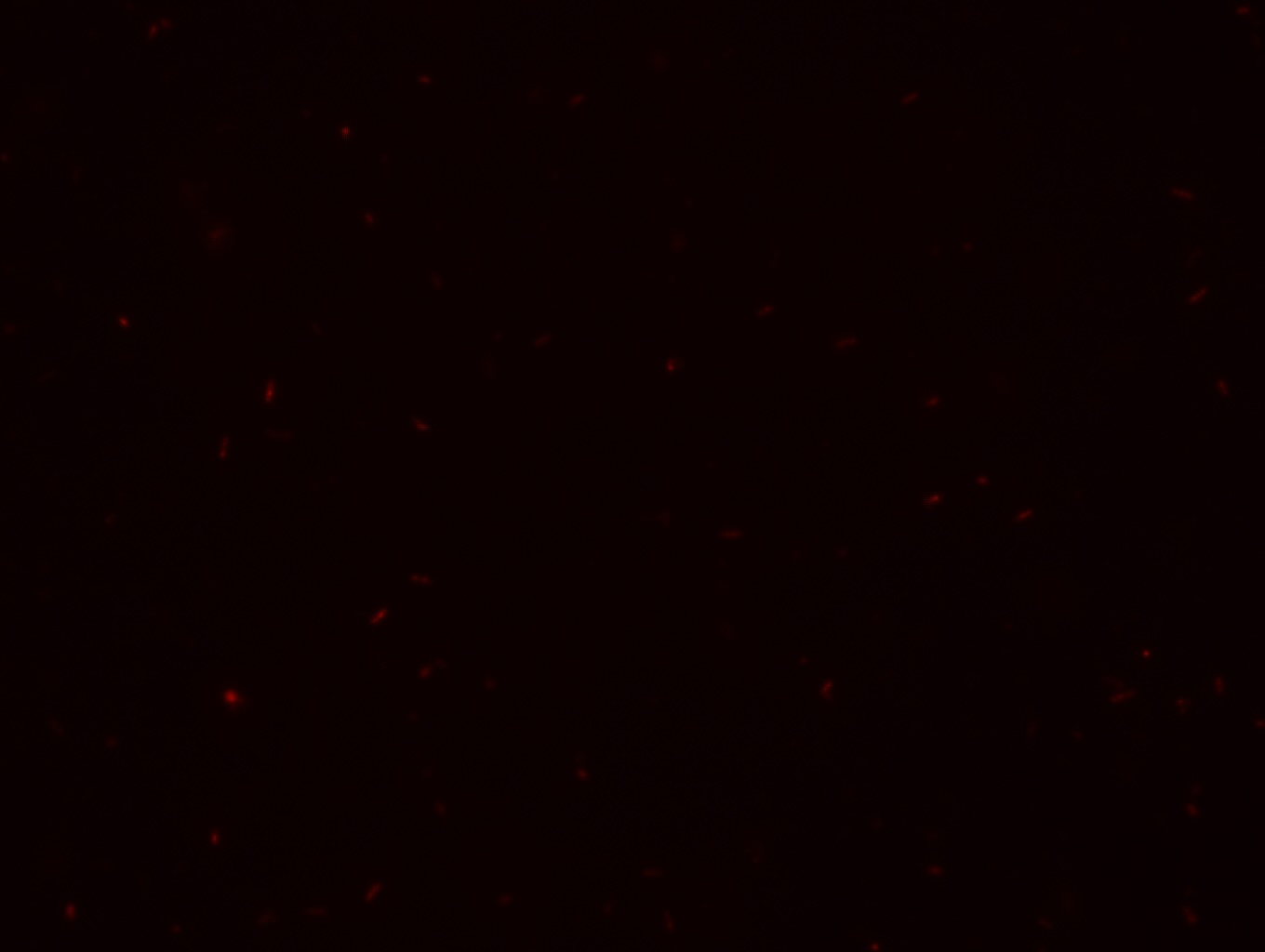

Supplement: File SI2 — Microscopy images of Halomonas sp. CUBES01. [file aem.00603-24-s0002.zip › Microscopy/Fructose_1st_0002.jpg]

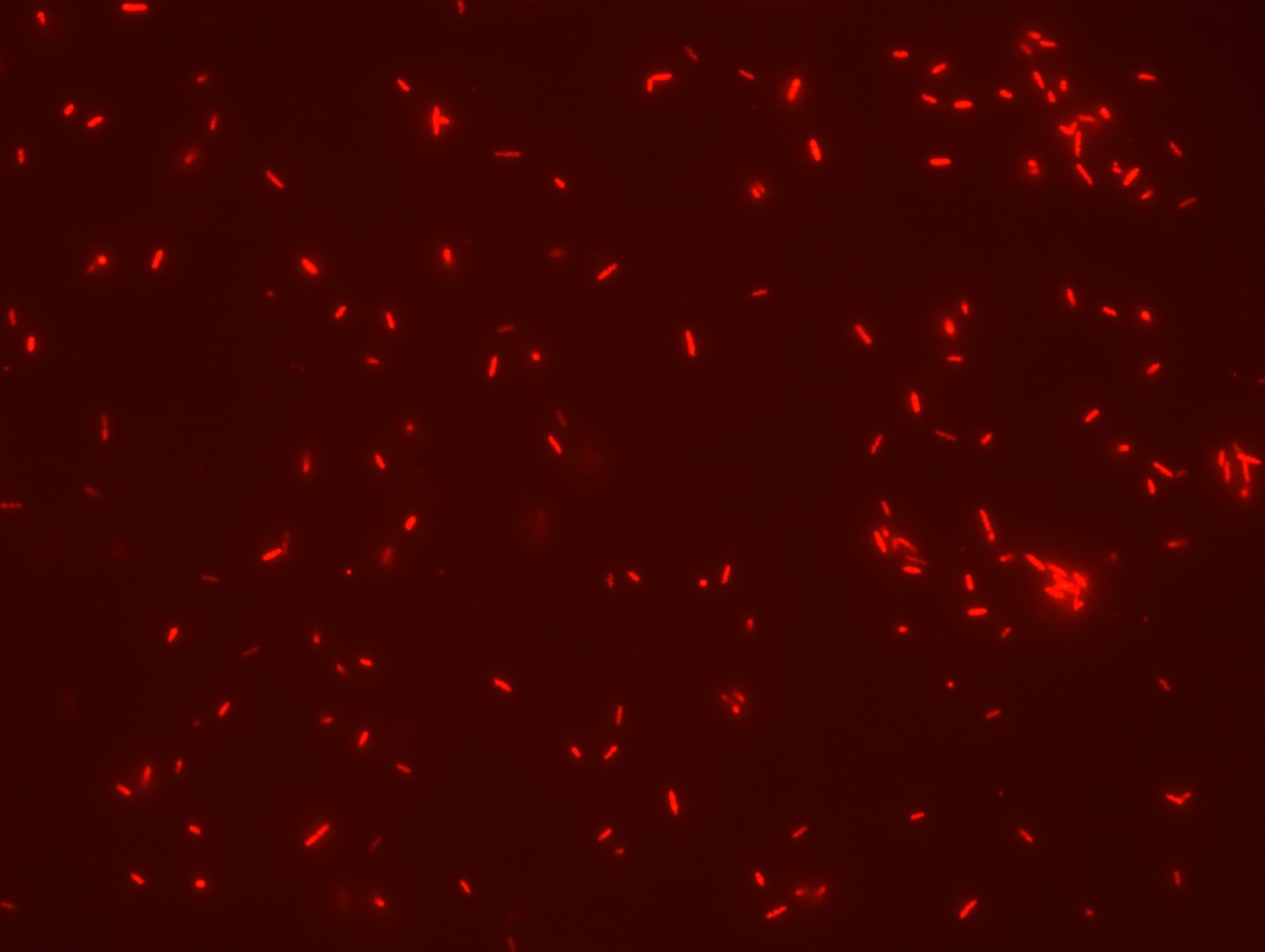

Supplement: File SI2 — Microscopy images of Halomonas sp. CUBES01. [file aem.00603-24-s0002.zip › Microscopy/Glycerol_3rd_0002.jpg]

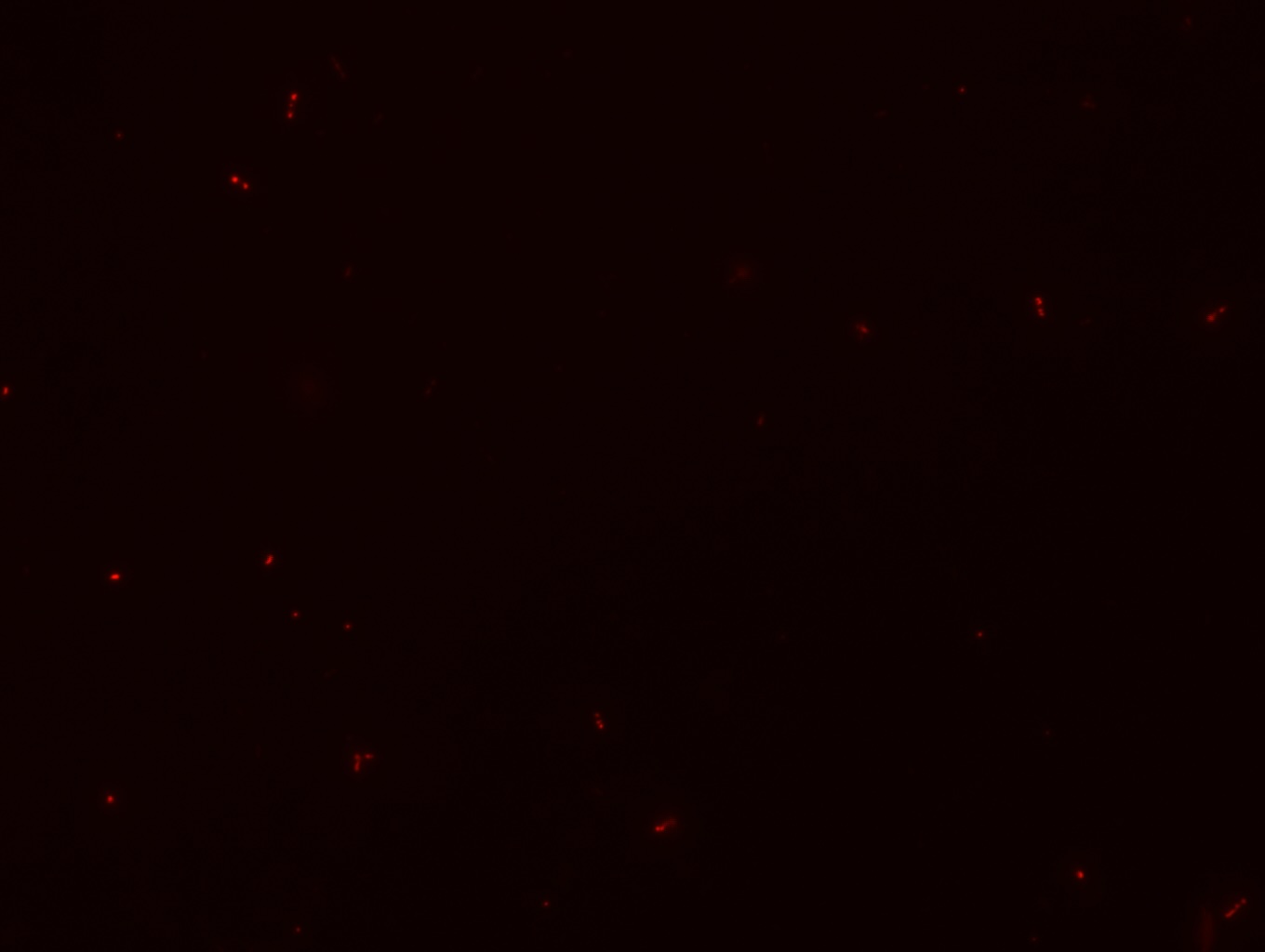

Supplement: File SI2 — Microscopy images of Halomonas sp. CUBES01. [file aem.00603-24-s0002.zip › Microscopy/Glucosamine_2nd_0001.jpg]

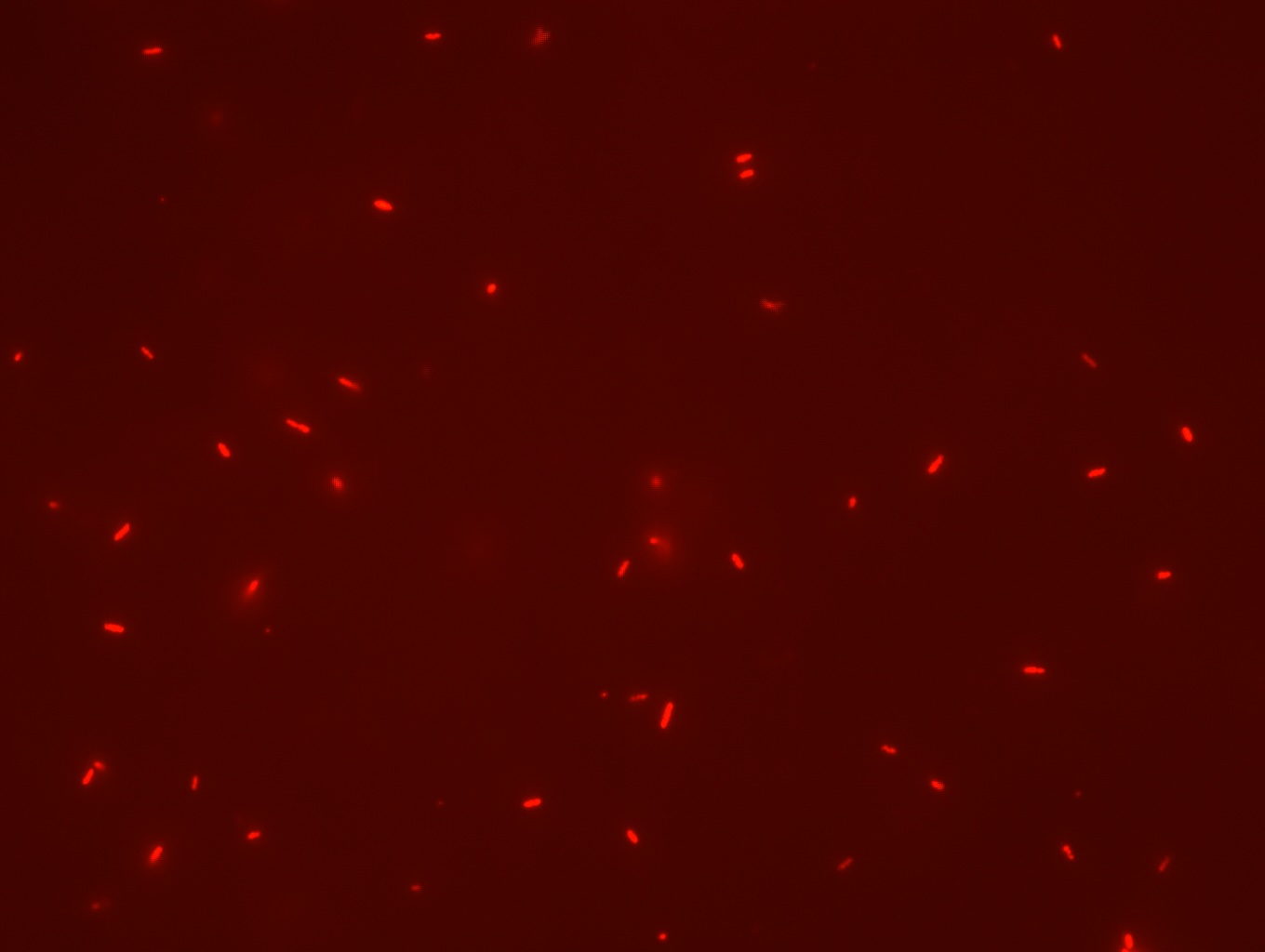

Supplement: File SI2 — Microscopy images of Halomonas sp. CUBES01. [file aem.00603-24-s0002.zip › Microscopy/Glycerol_3rd_0003.jpg]

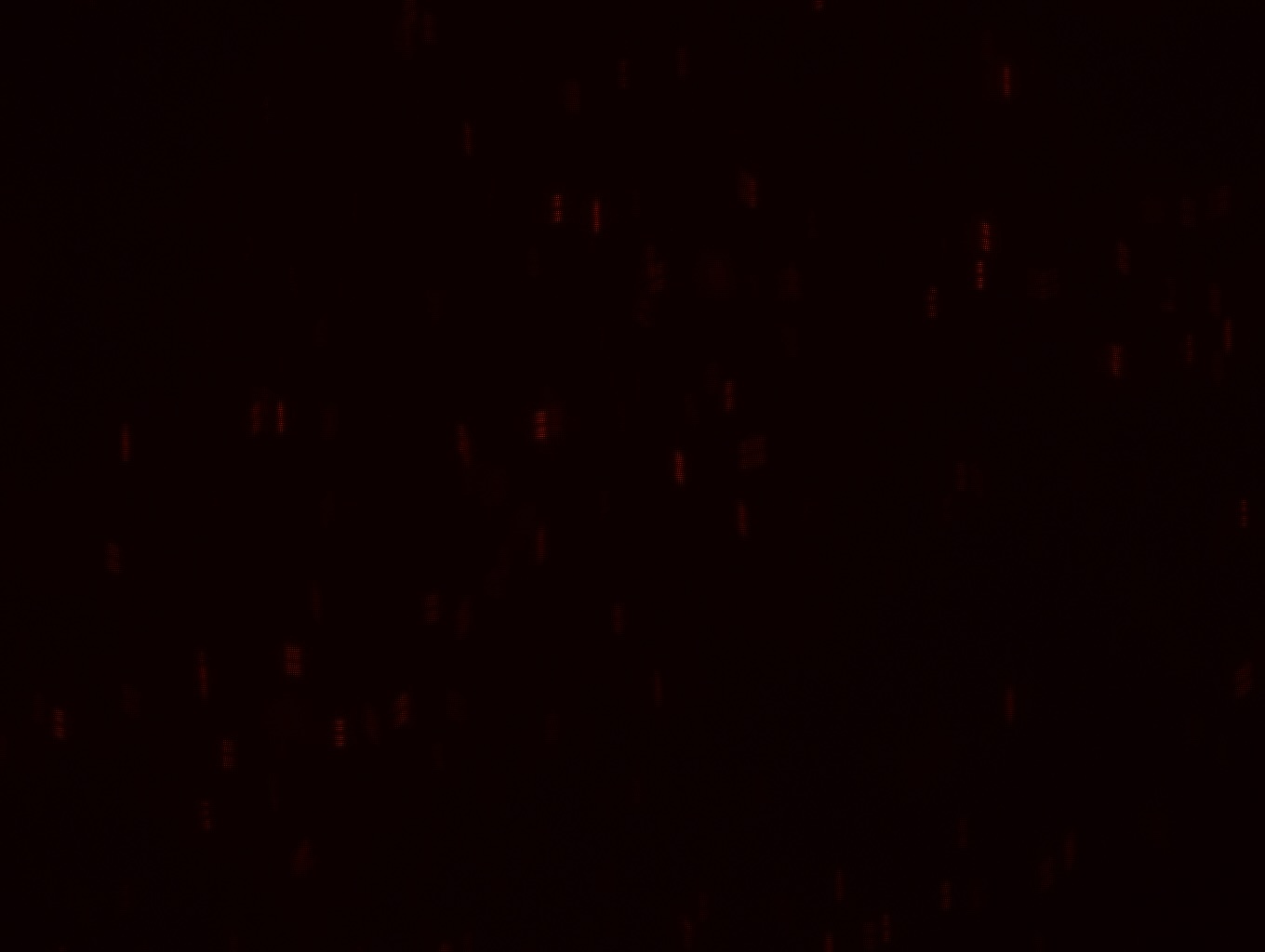

Supplement: File SI2 — Microscopy images of Halomonas sp. CUBES01. [file aem.00603-24-s0002.zip › Microscopy/Fructose_1st_0003.jpg]

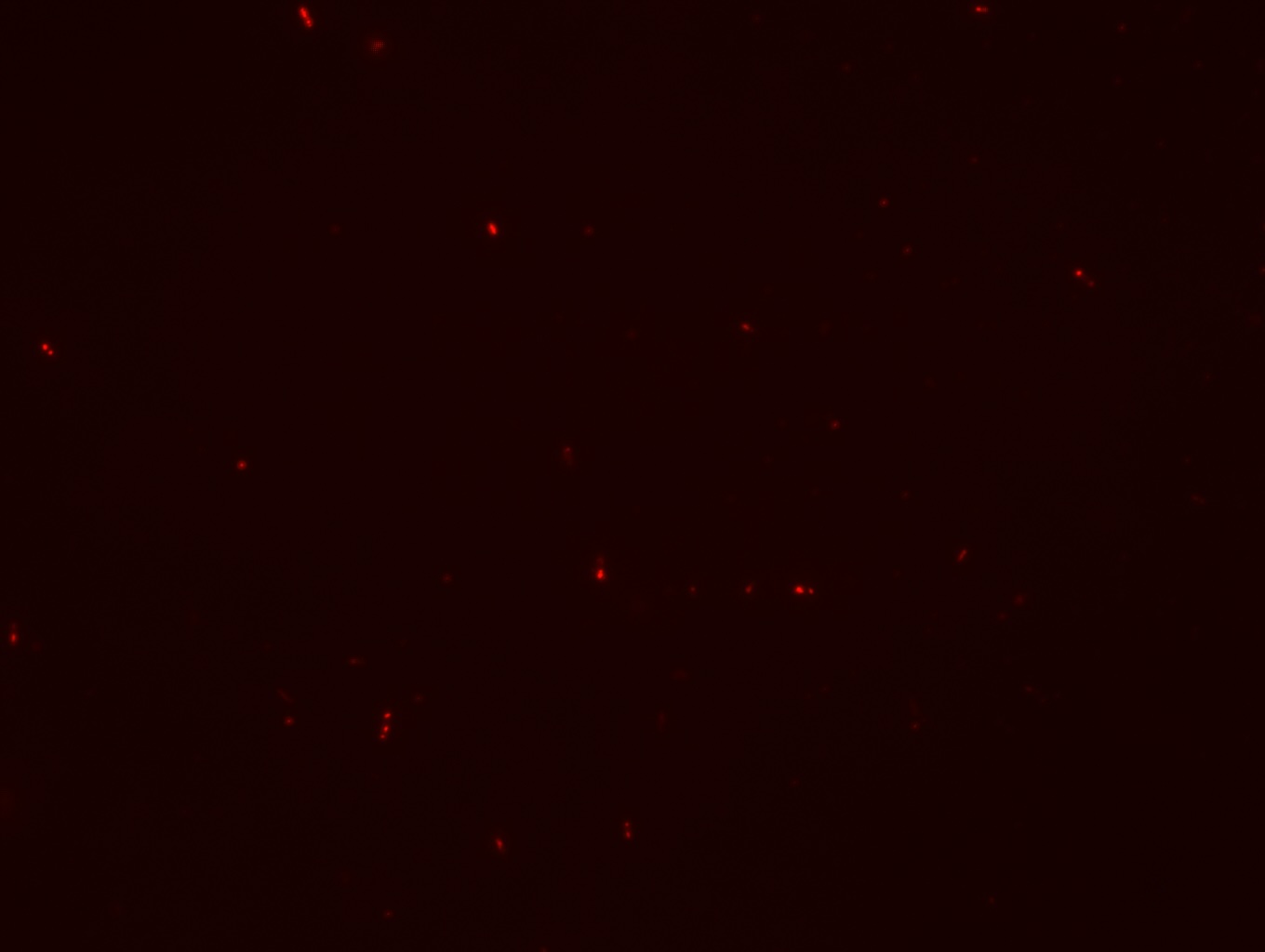

Supplement: File SI2 — Microscopy images of Halomonas sp. CUBES01. [file aem.00603-24-s0002.zip › Microscopy/Glucosamine_3rd_0003.jpg]

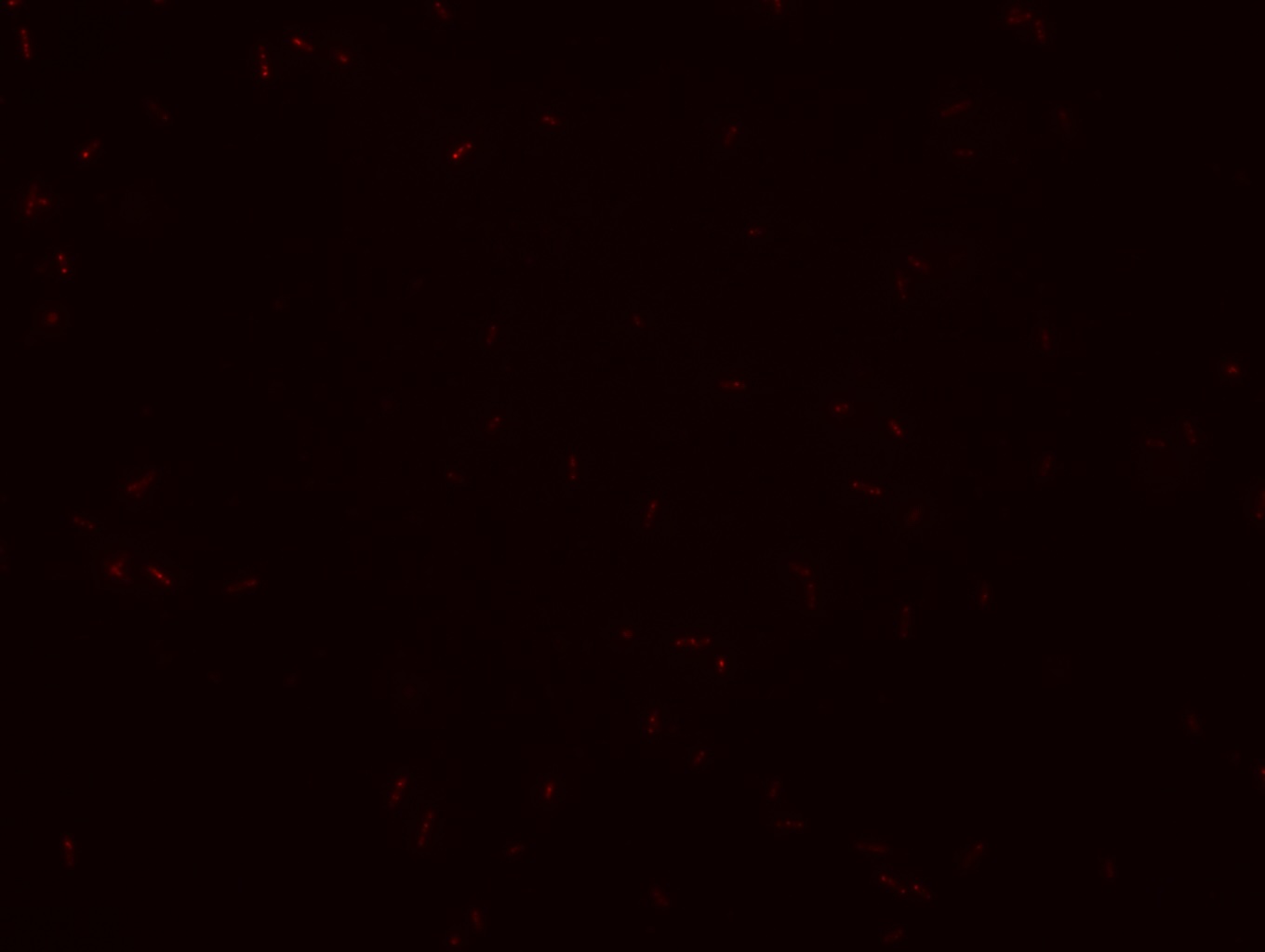

Supplement: File SI2 — Microscopy images of Halomonas sp. CUBES01. [file aem.00603-24-s0002.zip › Microscopy/Acetate_1st_0003.jpg]

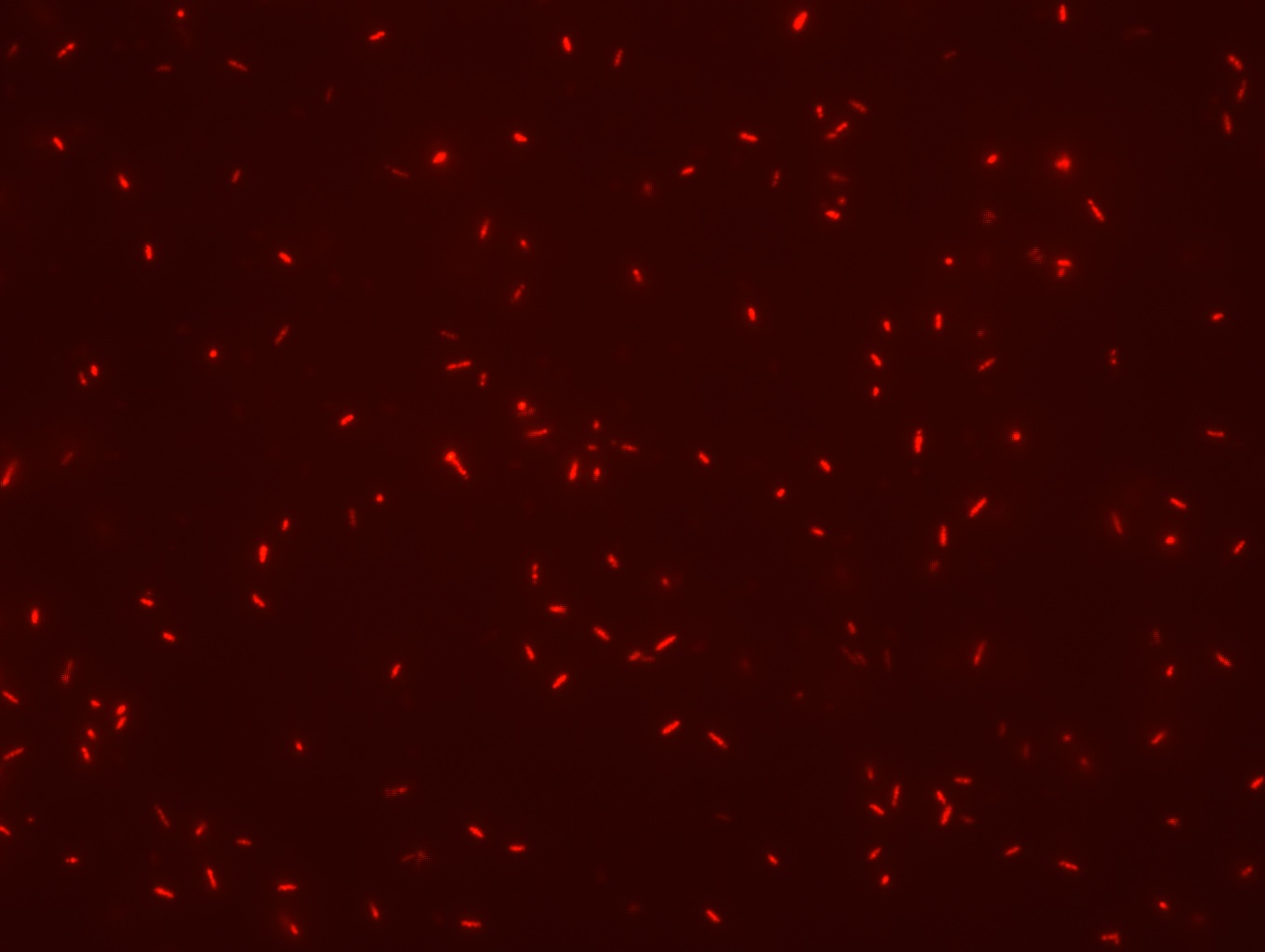

Supplement: File SI2 — Microscopy images of Halomonas sp. CUBES01. [file aem.00603-24-s0002.zip › Microscopy/Glycerol_2nd_0001.jpg]

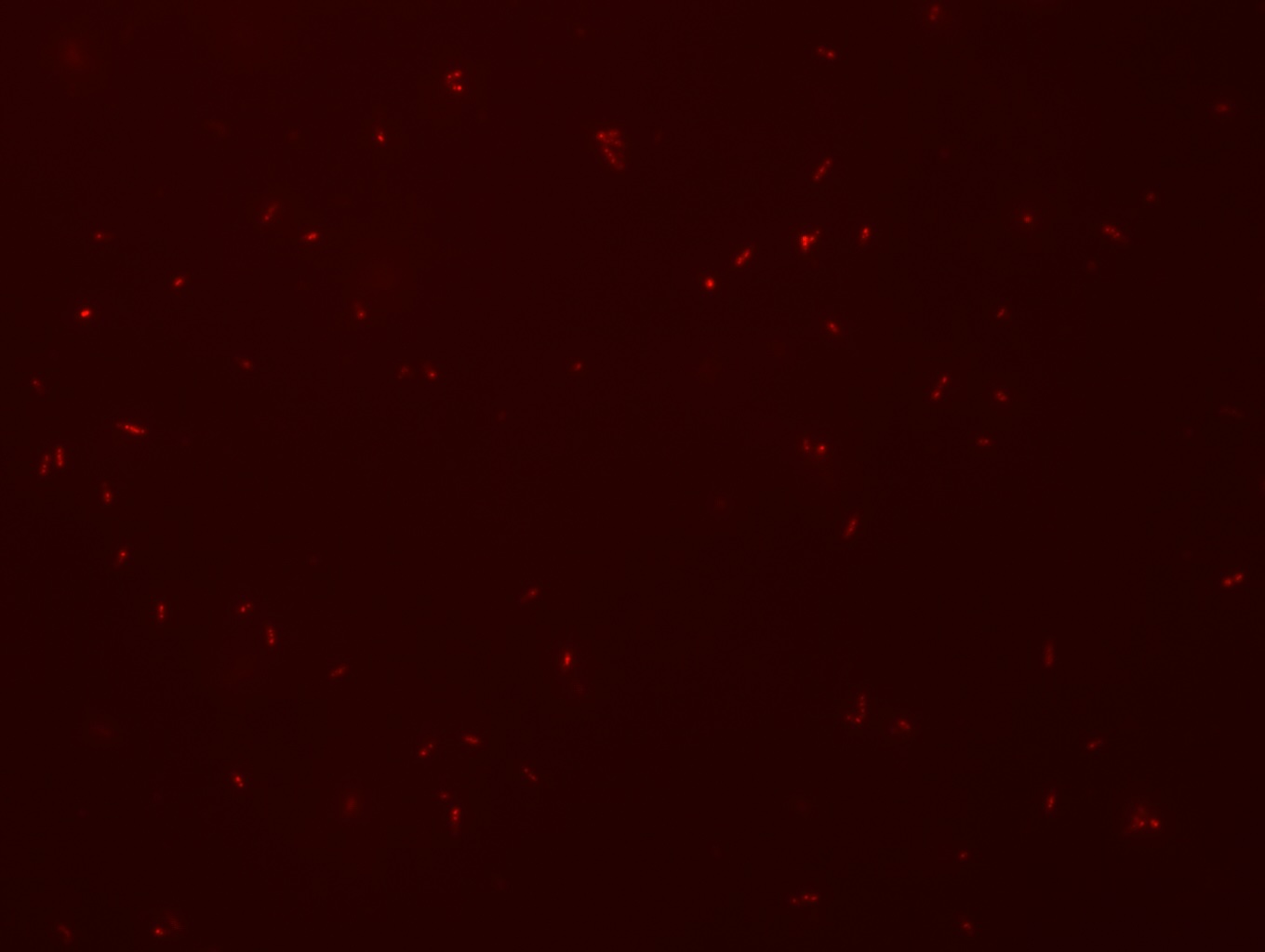

Supplement: File SI2 — Microscopy images of Halomonas sp. CUBES01. [file aem.00603-24-s0002.zip › Microscopy/Glucose_1st_0004.jpg]

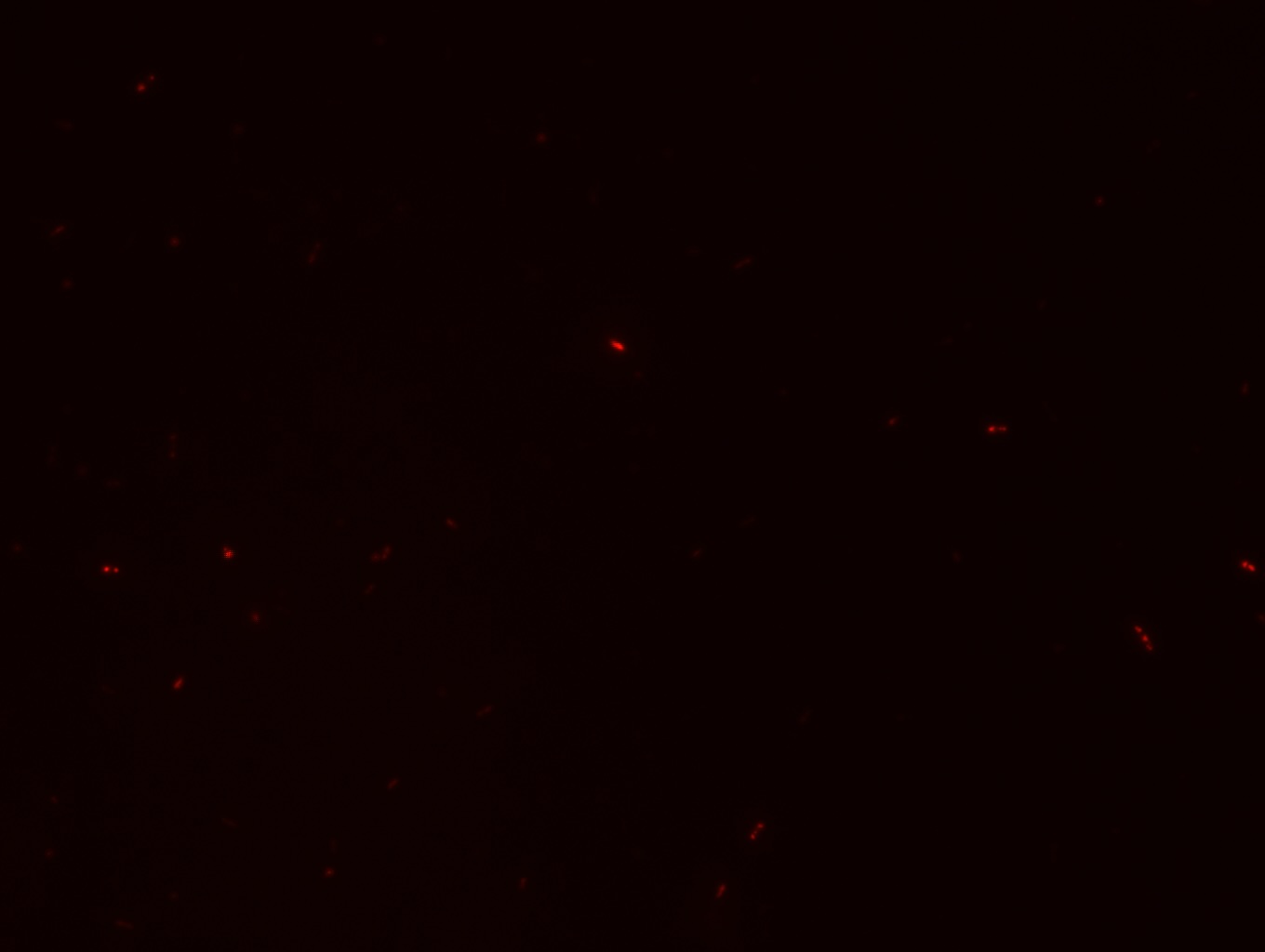

Supplement: File SI2 — Microscopy images of Halomonas sp. CUBES01. [file aem.00603-24-s0002.zip › Microscopy/Glucosamine_2nd_0004.jpg]

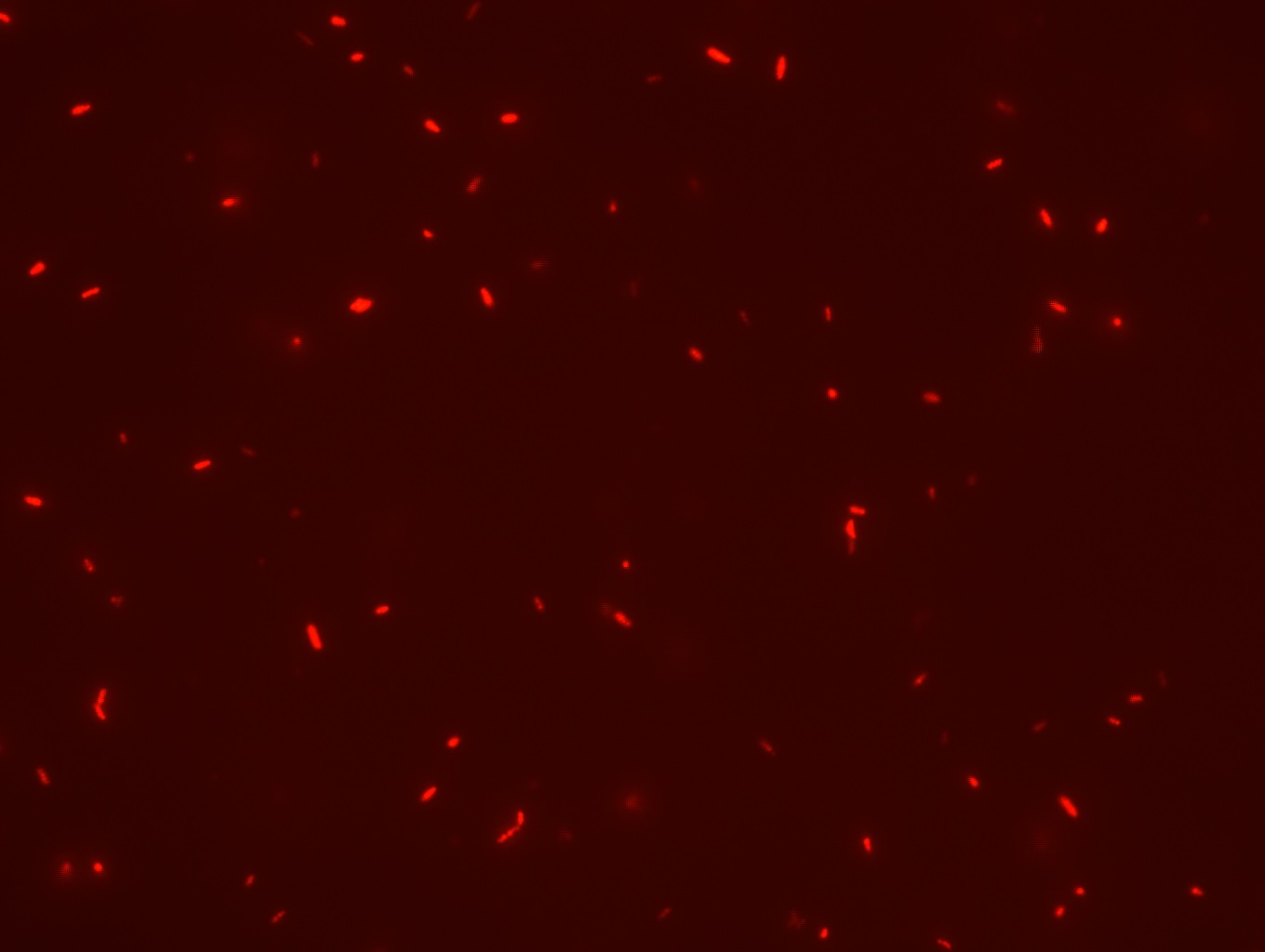

Supplement: File SI2 — Microscopy images of Halomonas sp. CUBES01. [file aem.00603-24-s0002.zip › Microscopy/Glycerol_2nd_0004.jpg]

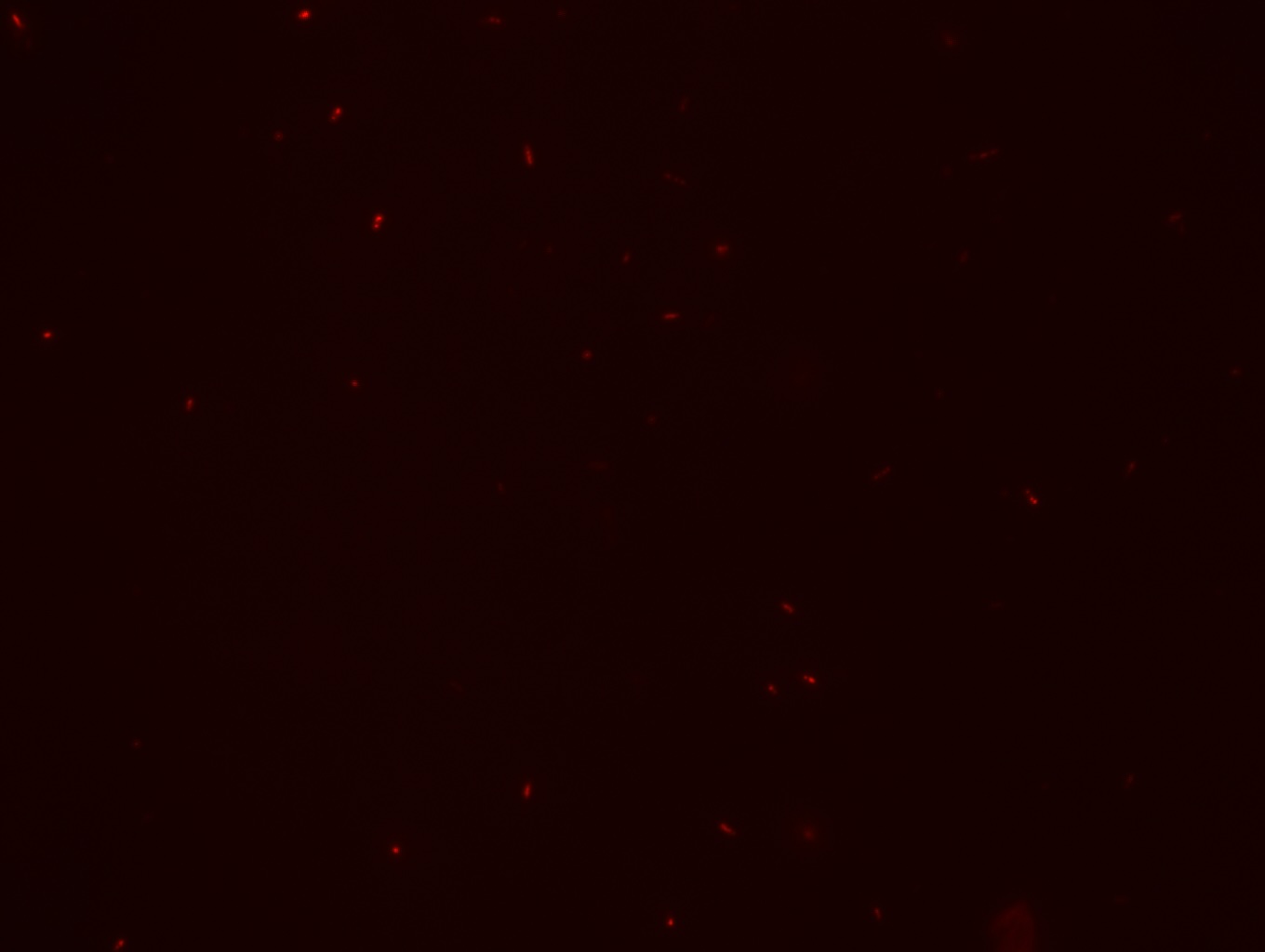

Supplement: File SI2 — Microscopy images of Halomonas sp. CUBES01. [file aem.00603-24-s0002.zip › Microscopy/Glucosamine_3rd_0004.jpg]

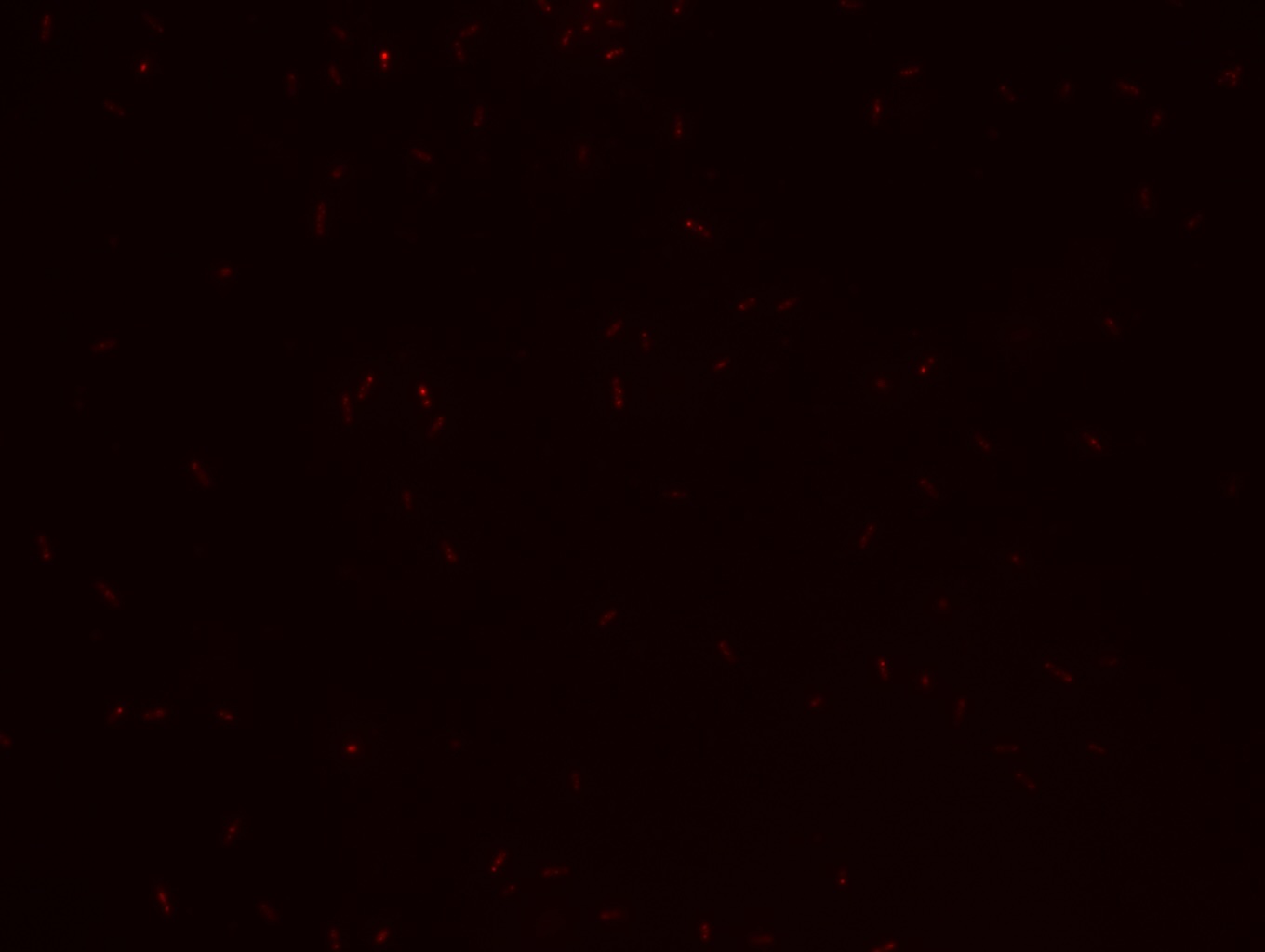

Supplement: File SI2 — Microscopy images of Halomonas sp. CUBES01. [file aem.00603-24-s0002.zip › Microscopy/Acetate_1st_0004.jpg]

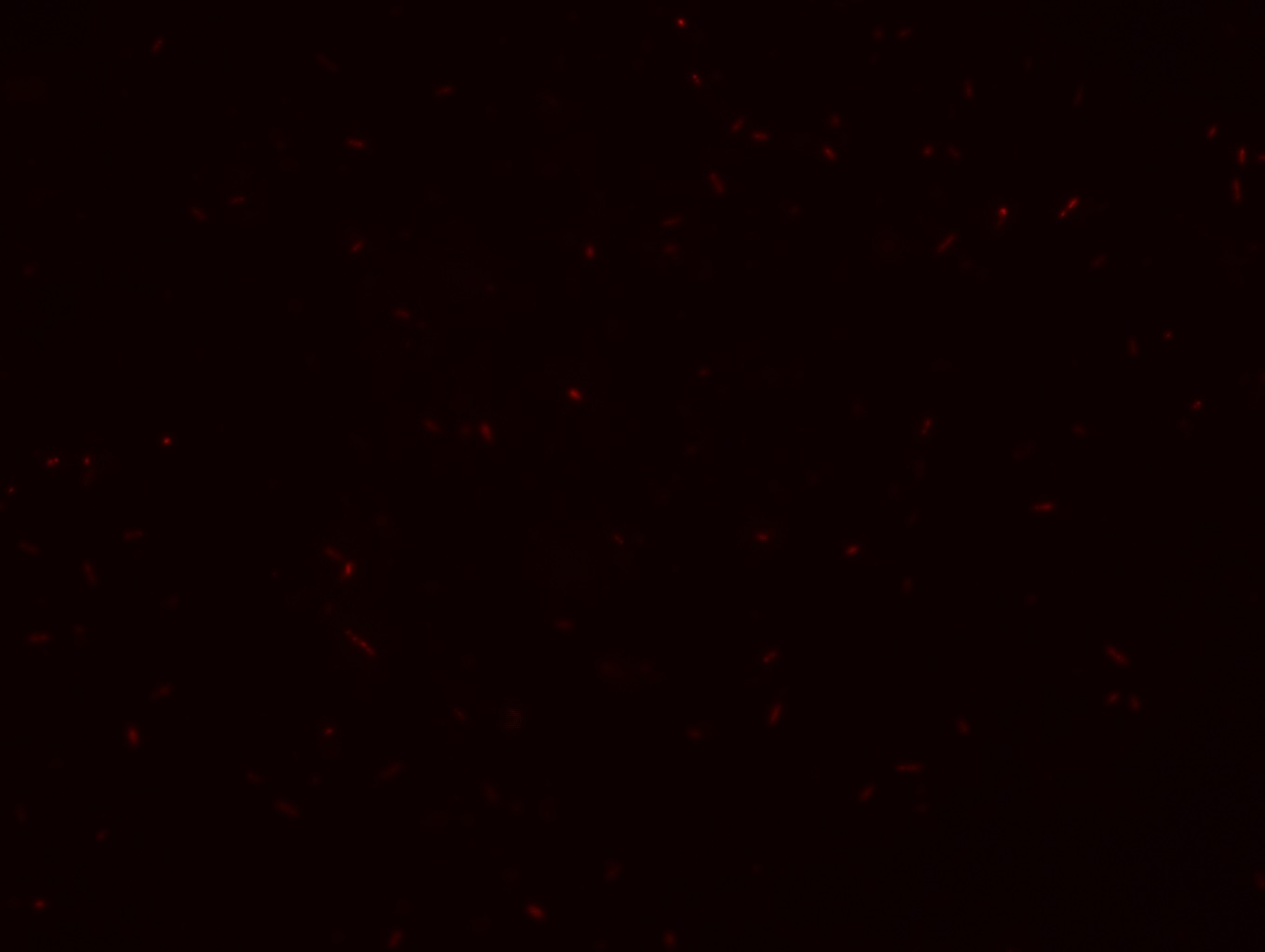

Supplement: File SI2 — Microscopy images of Halomonas sp. CUBES01. [file aem.00603-24-s0002.zip › Microscopy/Fructose_1st_0004.jpg]

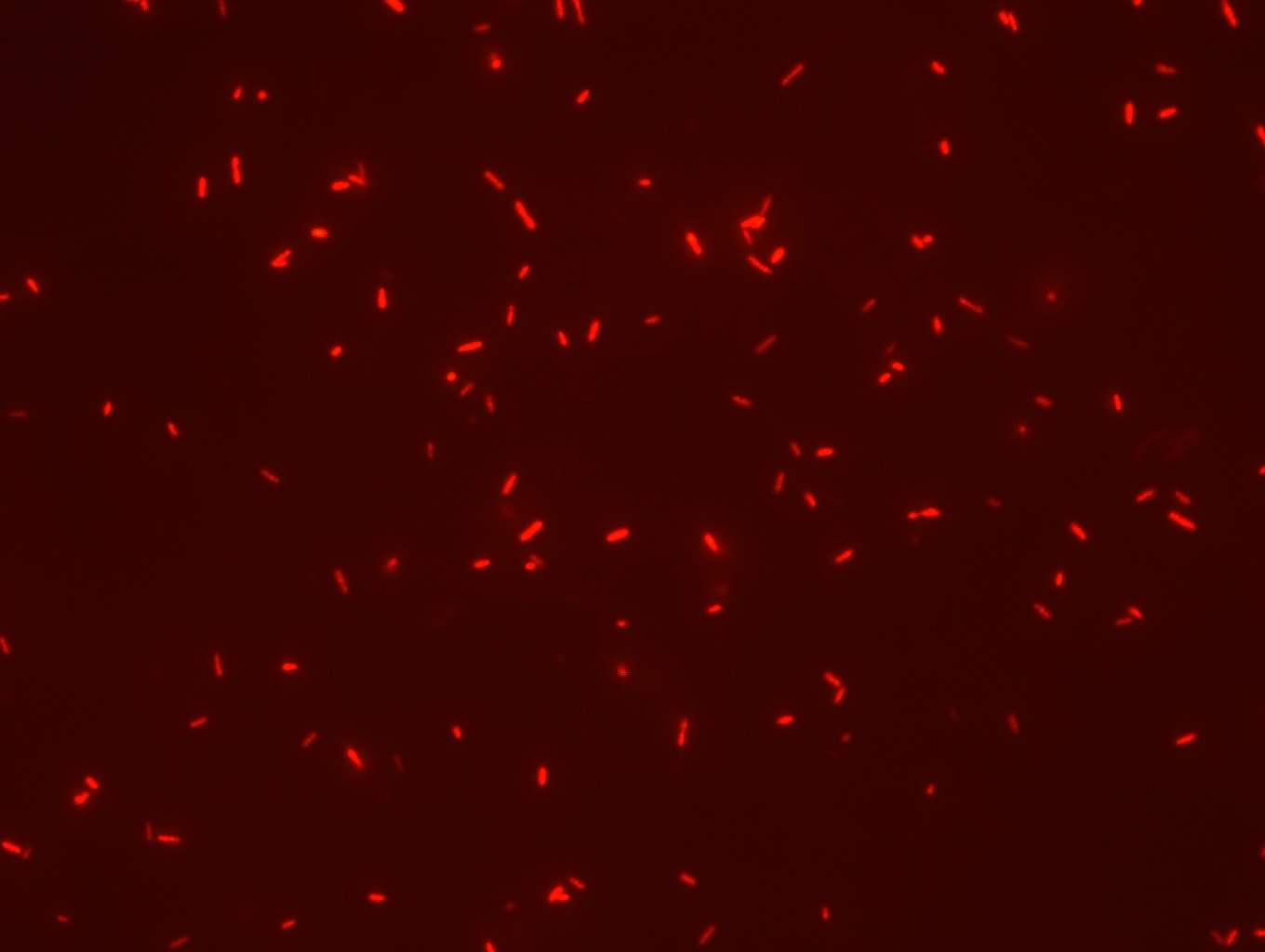

Supplement: File SI2 — Microscopy images of Halomonas sp. CUBES01. [file aem.00603-24-s0002.zip › Microscopy/Glycerol_3rd_0004.jpg]

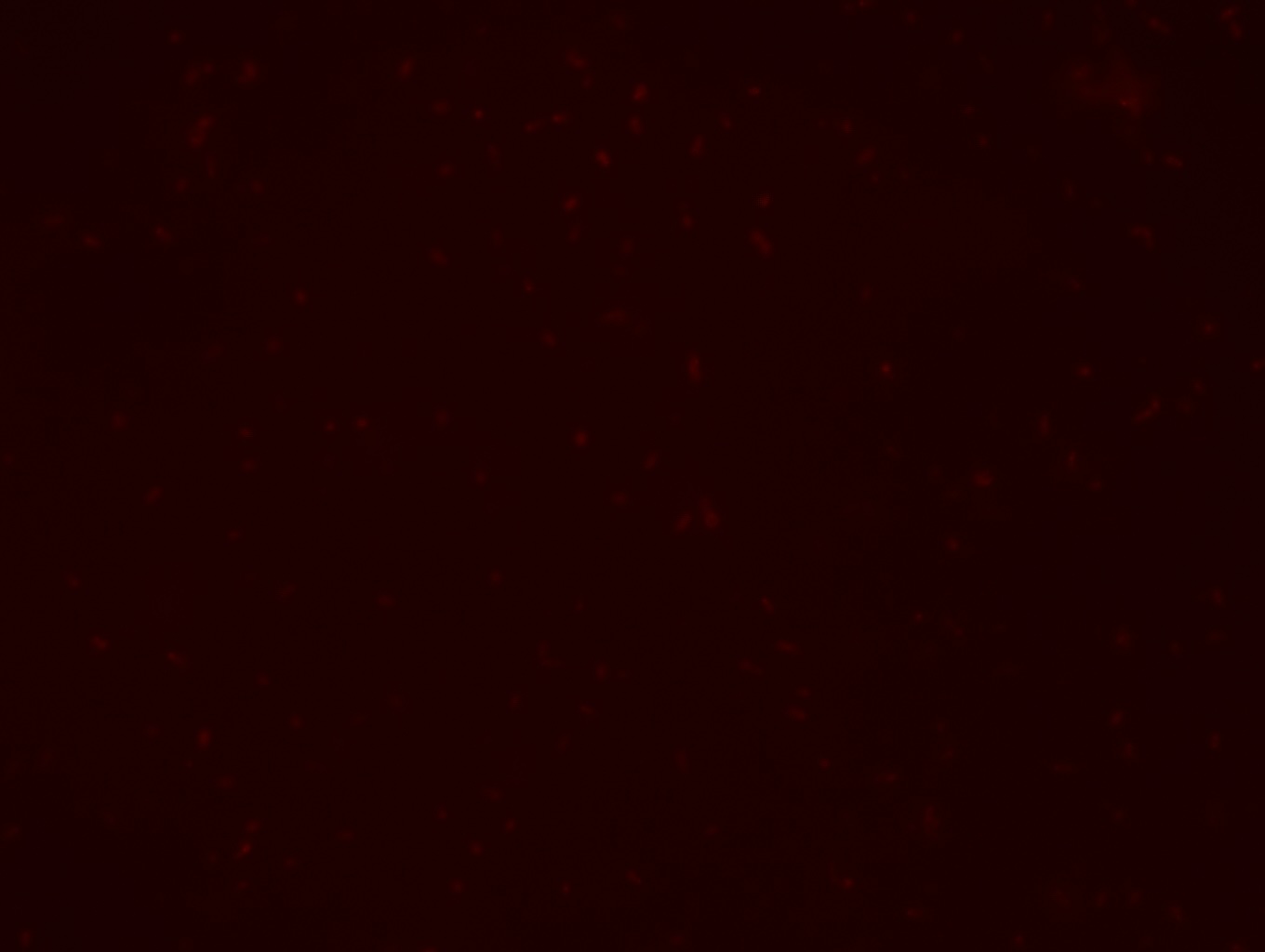

Supplement: File SI2 — Microscopy images of Halomonas sp. CUBES01. [file aem.00603-24-s0002.zip › Microscopy/NB_2nd_0003.jpg]

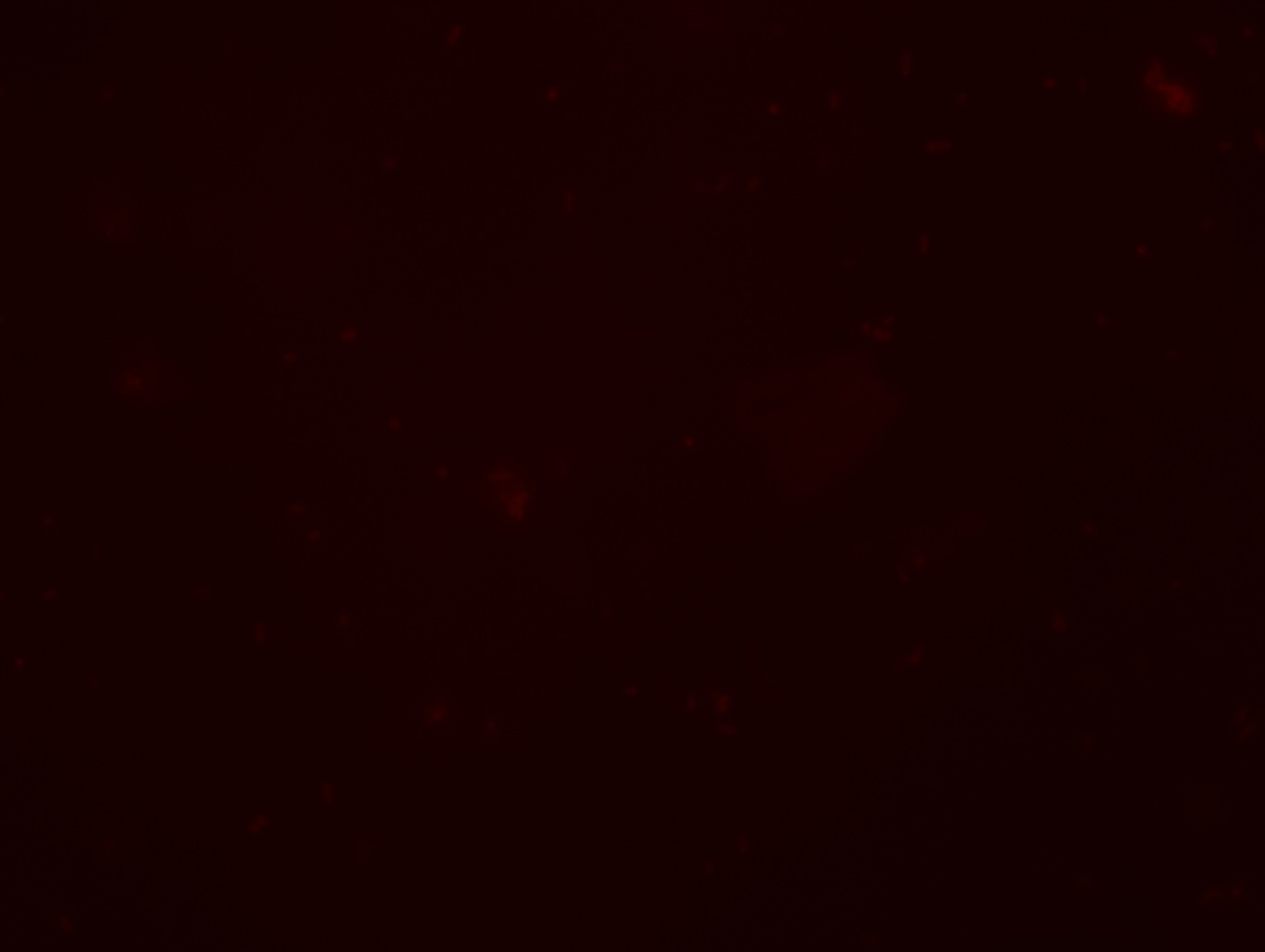

Supplement: File SI2 — Microscopy images of Halomonas sp. CUBES01. [file aem.00603-24-s0002.zip › Microscopy/NB_3rd_0001.jpg]

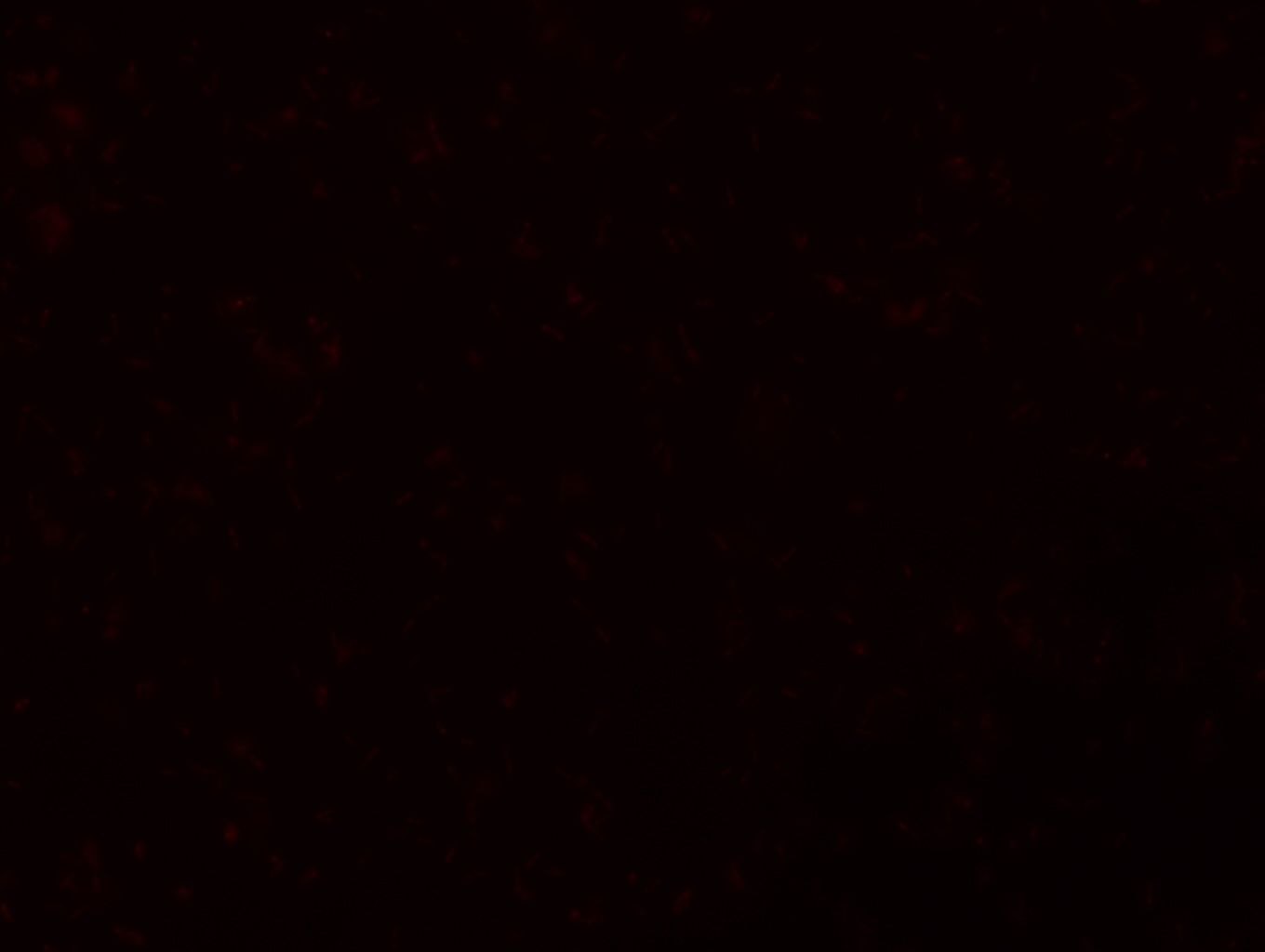

Supplement: File SI2 — Microscopy images of Halomonas sp. CUBES01. [file aem.00603-24-s0002.zip › Microscopy/Propionate_1st_0001.jpg]

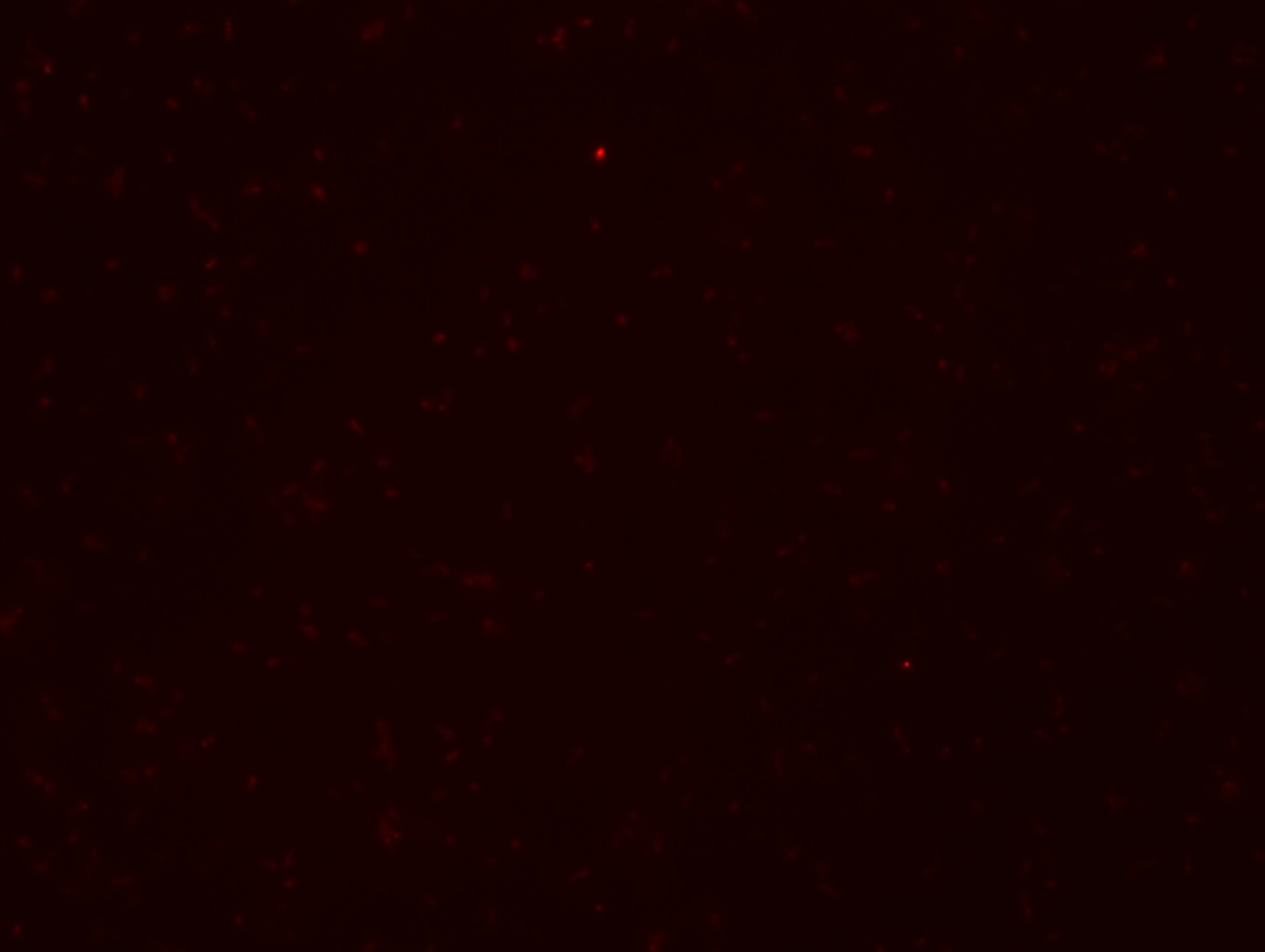

Supplement: File SI2 — Microscopy images of Halomonas sp. CUBES01. [file aem.00603-24-s0002.zip › Microscopy/NB_2nd_0002.jpg]

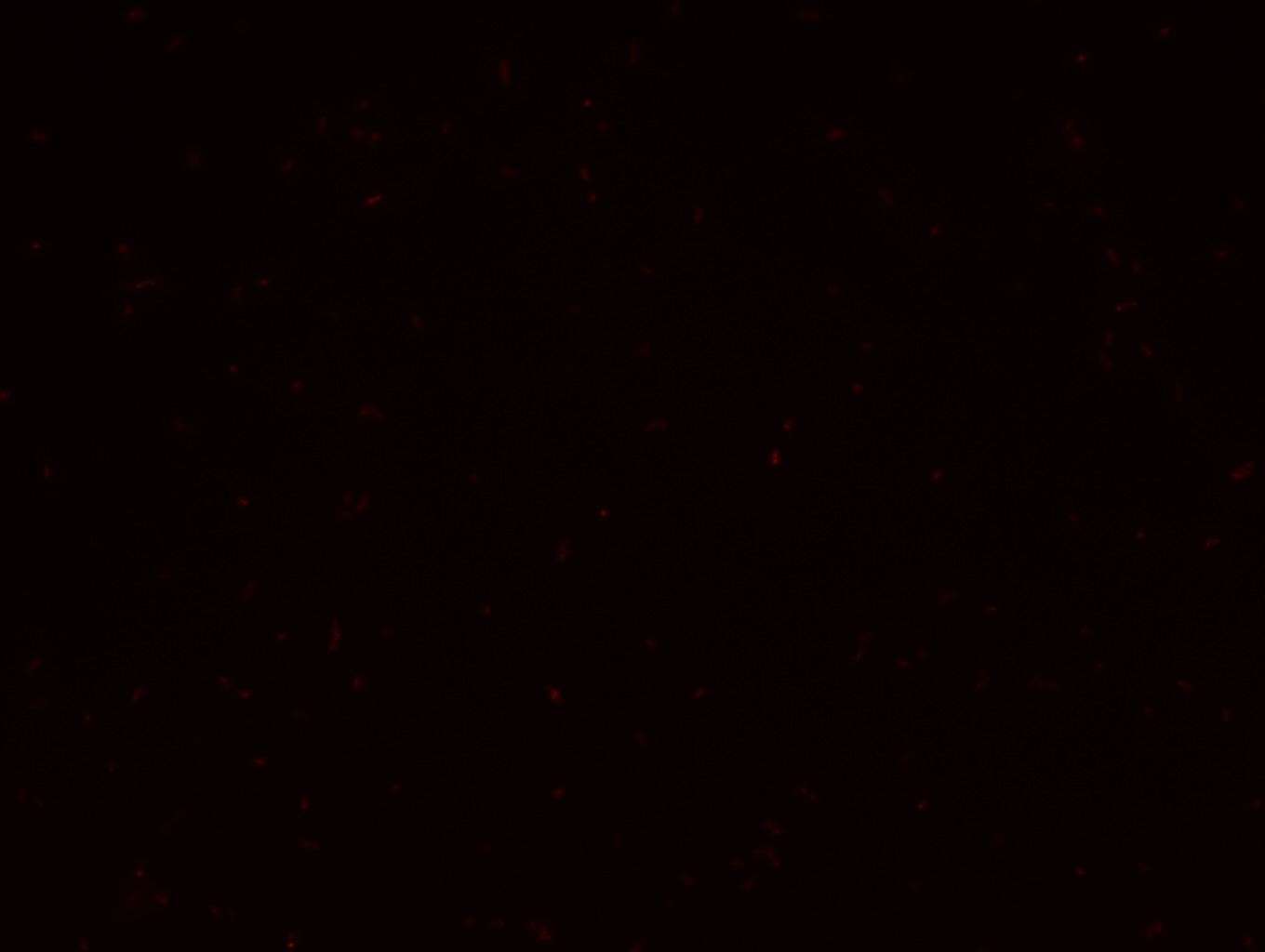

Supplement: File SI2 — Microscopy images of Halomonas sp. CUBES01. [file aem.00603-24-s0002.zip › Microscopy/Acetyl-Glucosamine_1st_0001.jpg]

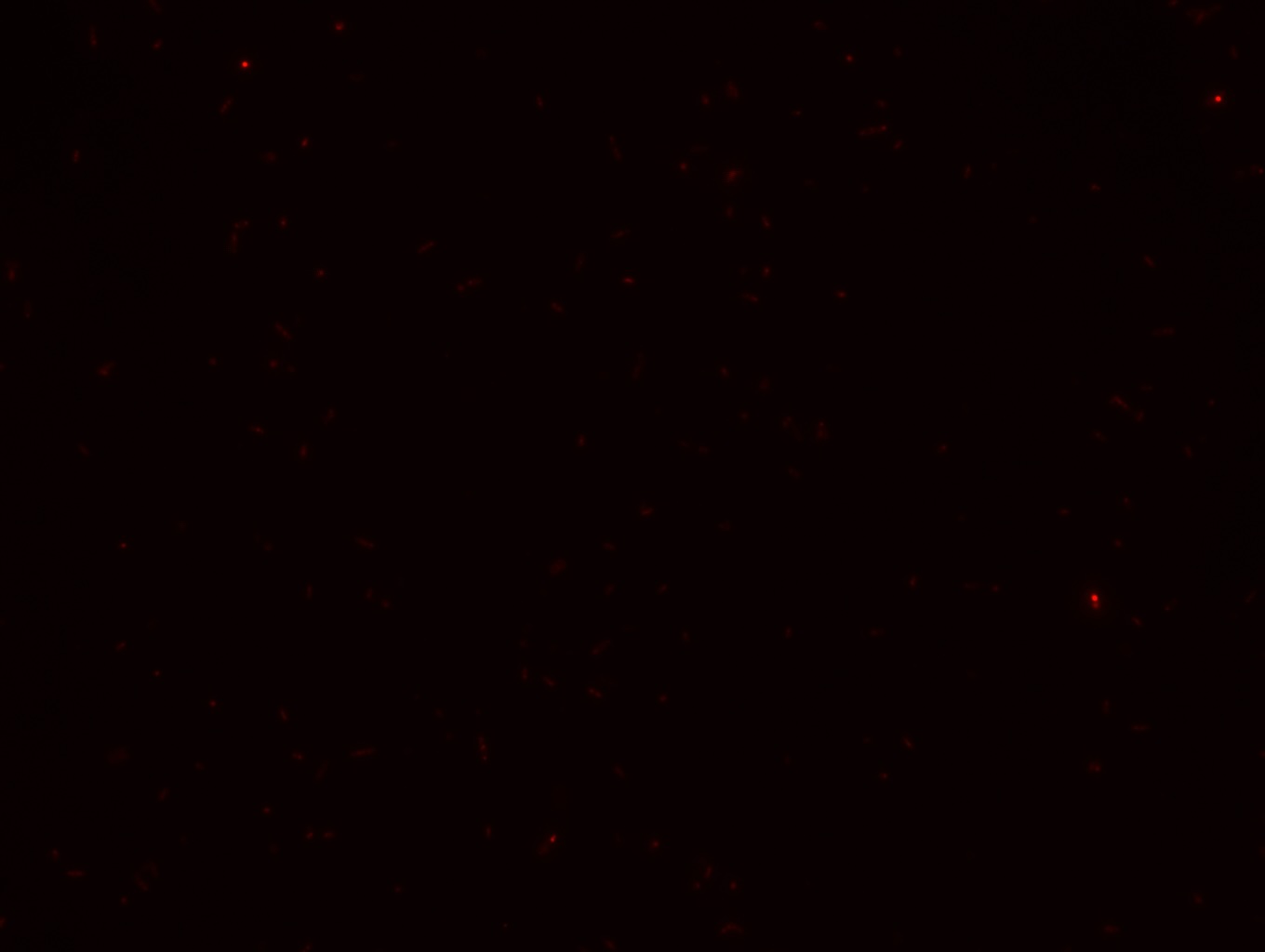

Supplement: File SI2 — Microscopy images of Halomonas sp. CUBES01. [file aem.00603-24-s0002.zip › Microscopy/Acetyl-Glucosamine_1st_0003.jpg]

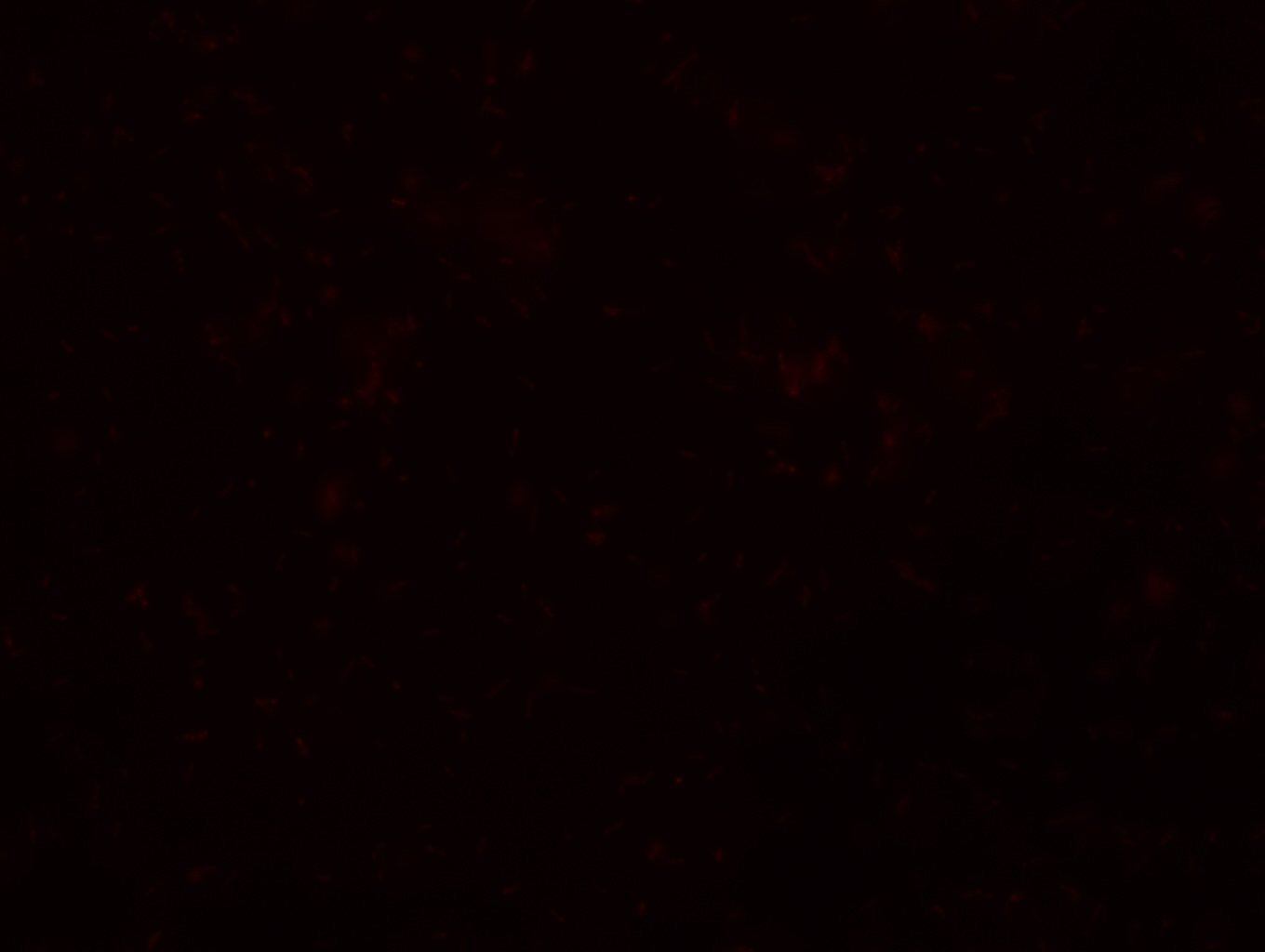

Supplement: File SI2 — Microscopy images of Halomonas sp. CUBES01. [file aem.00603-24-s0002.zip › Microscopy/Propionate_1st_0002.jpg]

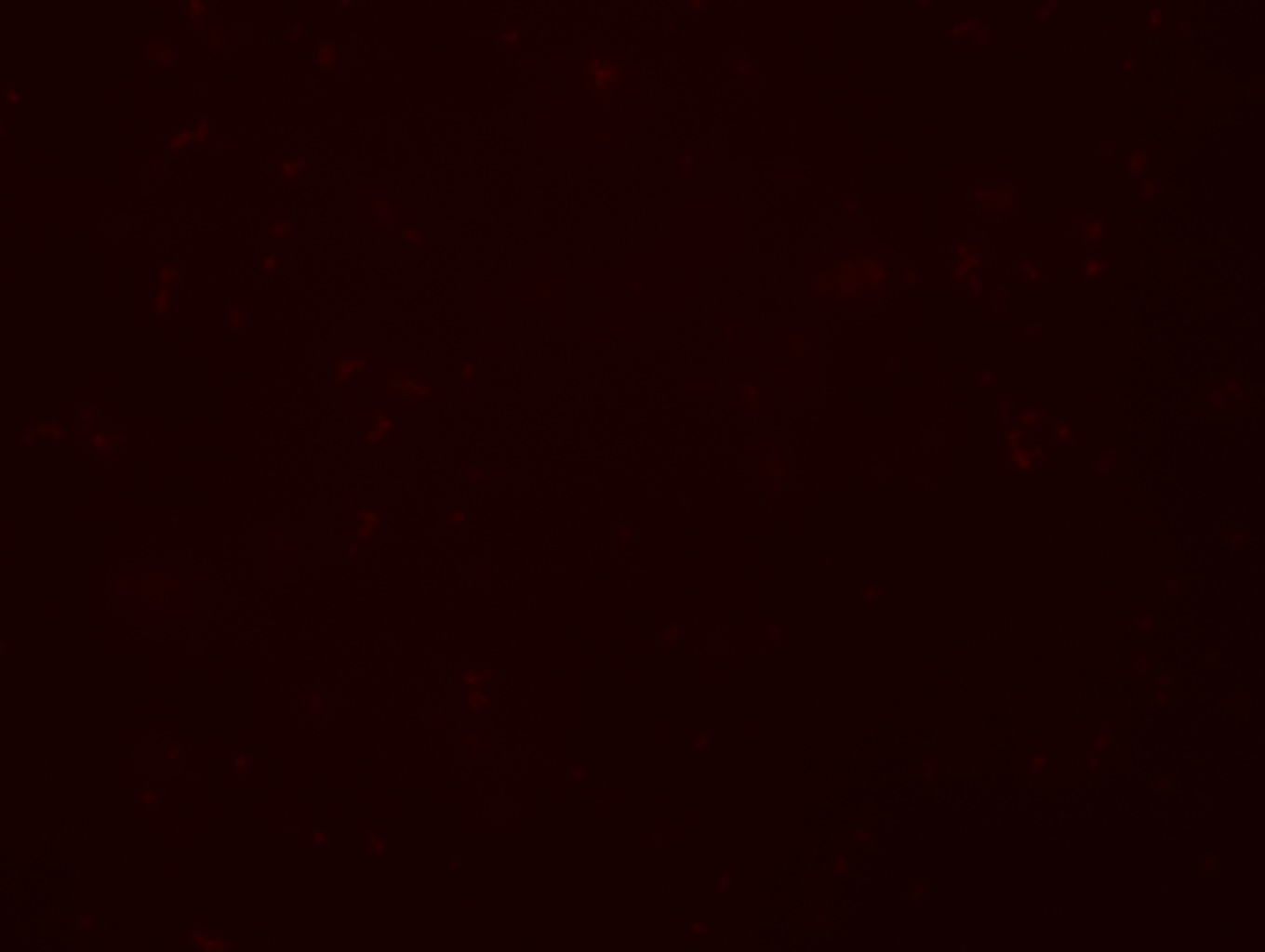

Supplement: File SI2 — Microscopy images of Halomonas sp. CUBES01. [file aem.00603-24-s0002.zip › Microscopy/NB_3rd_0002.jpg]

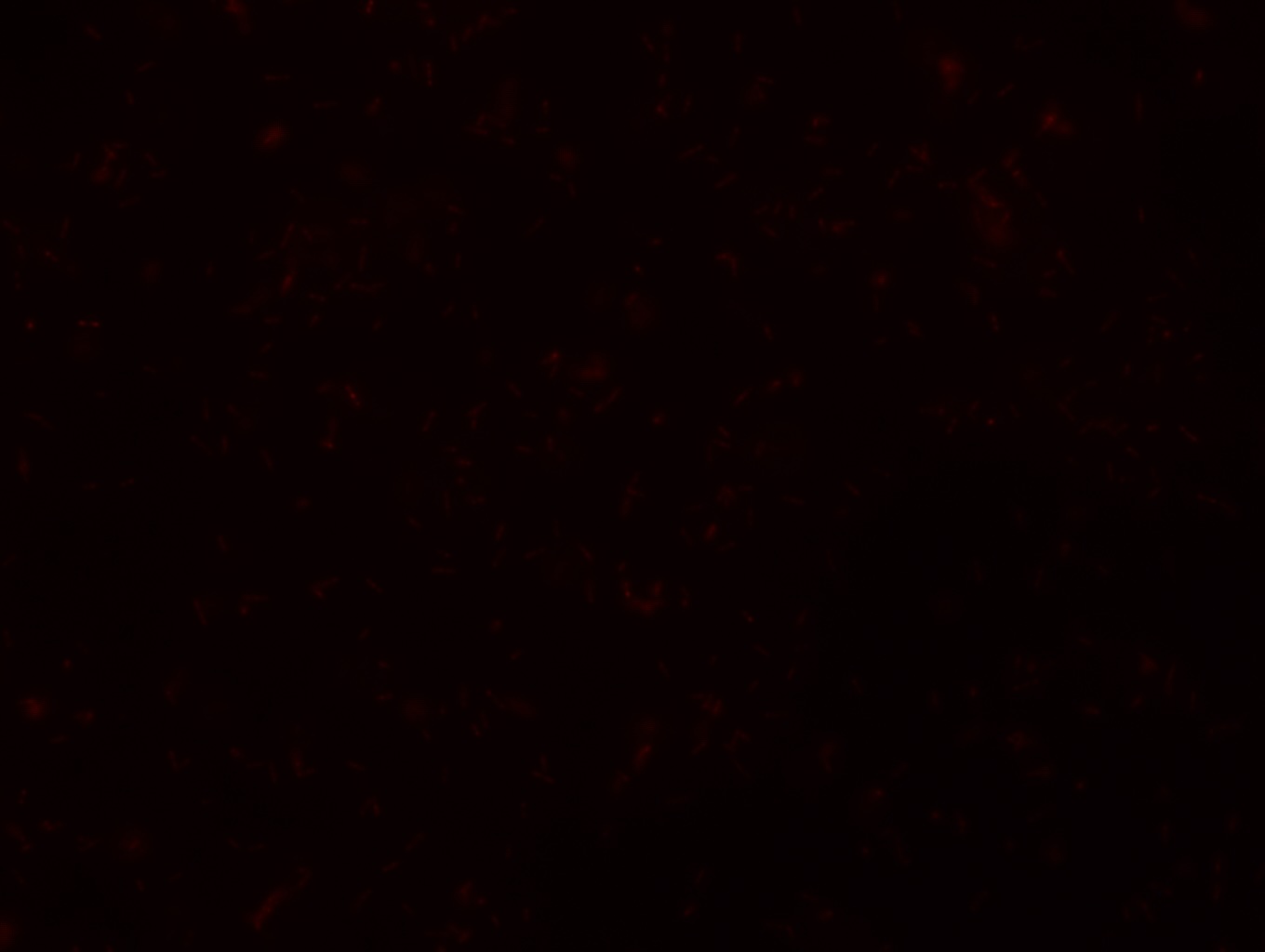

Supplement: File SI2 — Microscopy images of Halomonas sp. CUBES01. [file aem.00603-24-s0002.zip › Microscopy/Propionate_1st_0003.jpg]

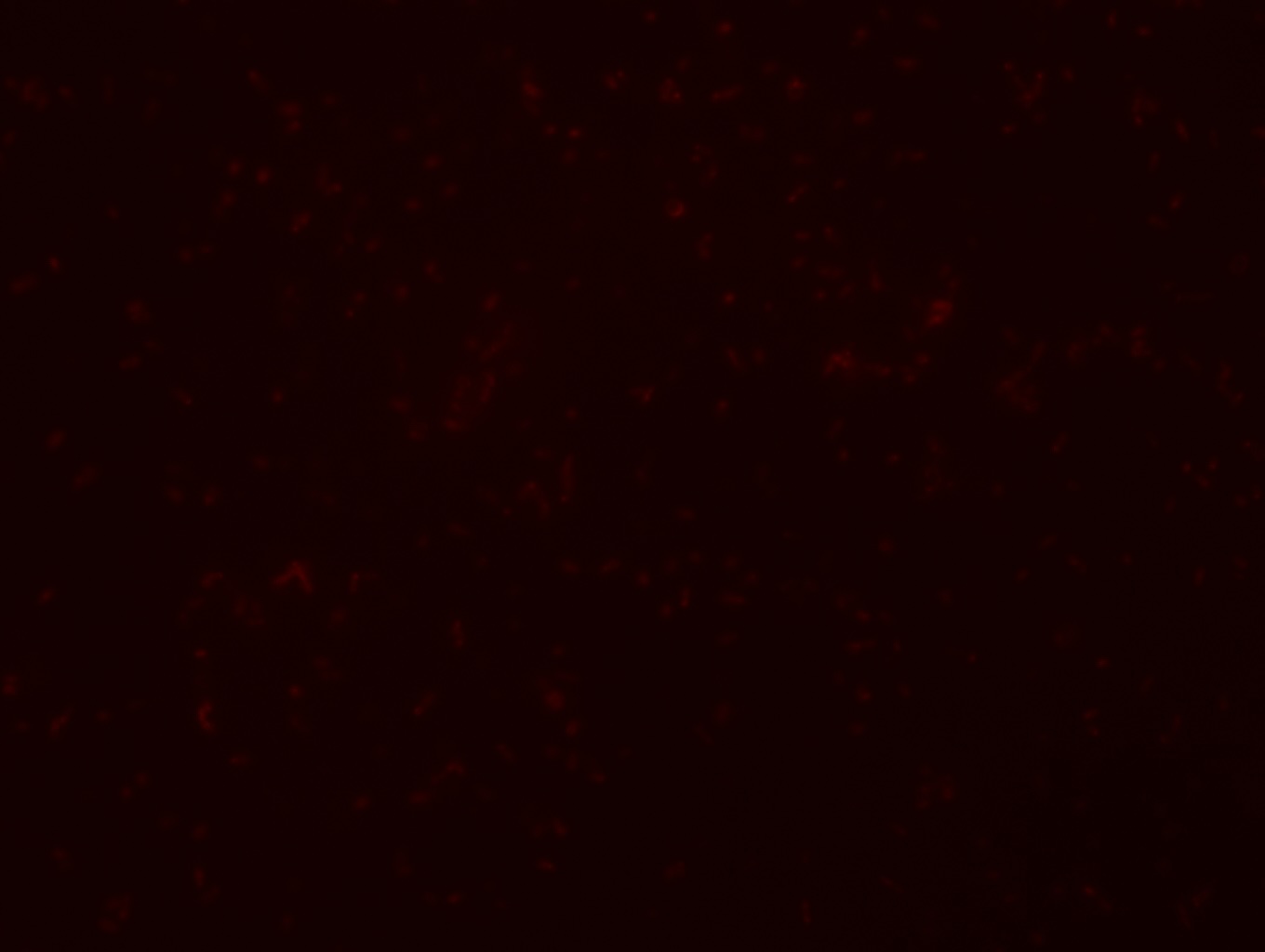

Supplement: File SI2 — Microscopy images of Halomonas sp. CUBES01. [file aem.00603-24-s0002.zip › Microscopy/NB_2nd_0001.jpg]

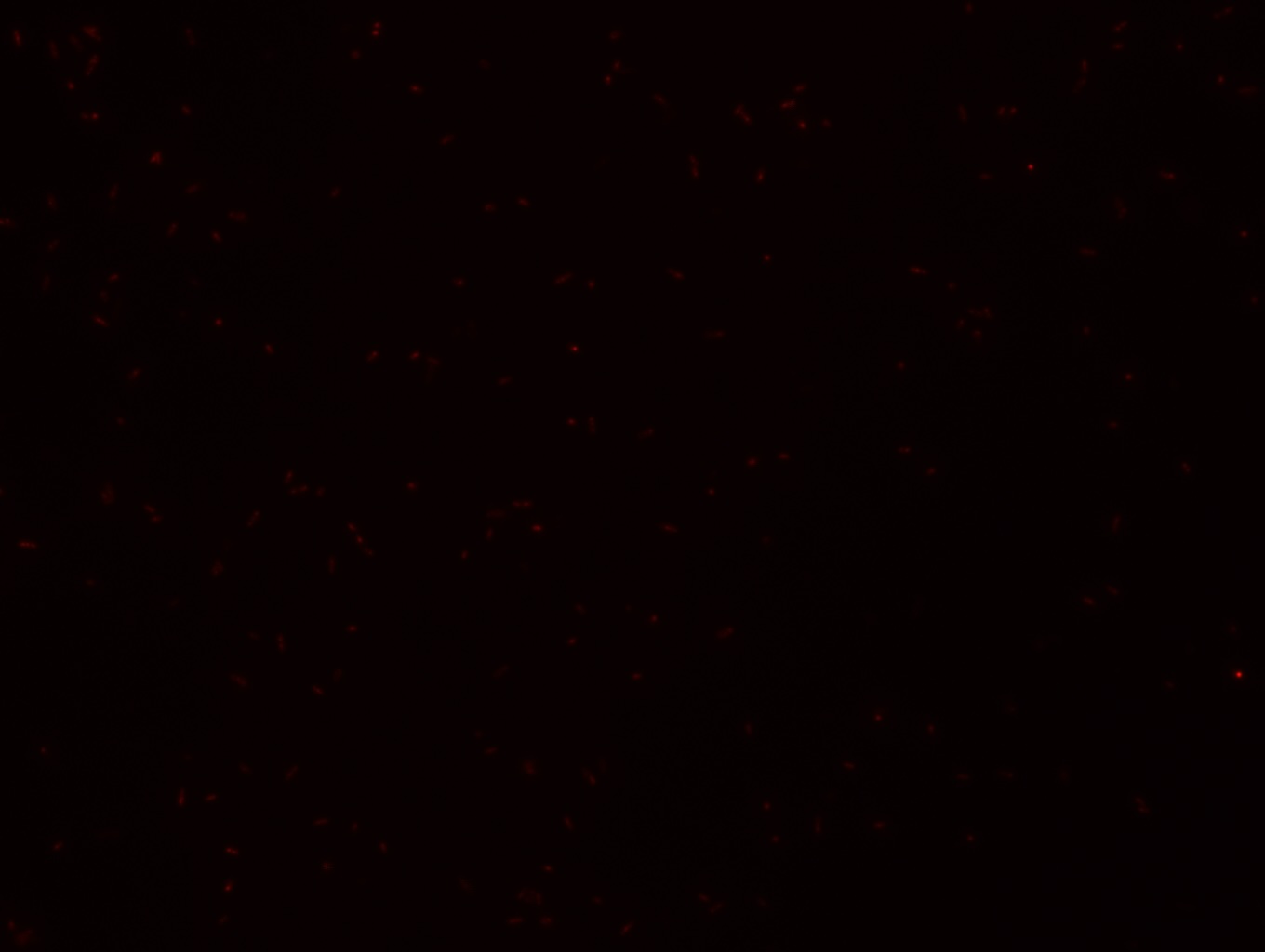

Supplement: File SI2 — Microscopy images of Halomonas sp. CUBES01. [file aem.00603-24-s0002.zip › Microscopy/Acetyl-Glucosamine_1st_0002.jpg]

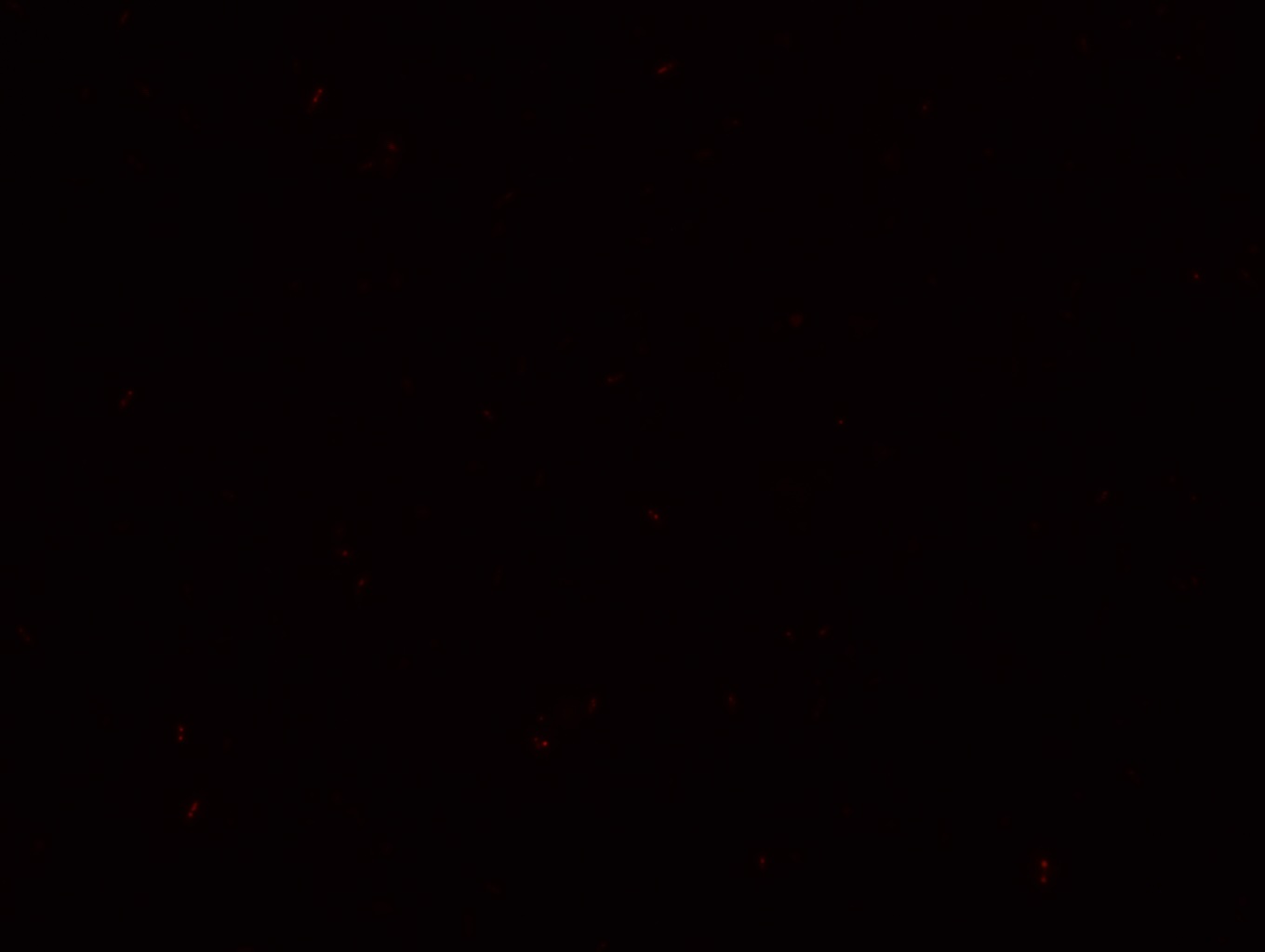

Supplement: File SI2 — Microscopy images of Halomonas sp. CUBES01. [file aem.00603-24-s0002.zip › Microscopy/Sucrose_1st_0002.jpg]

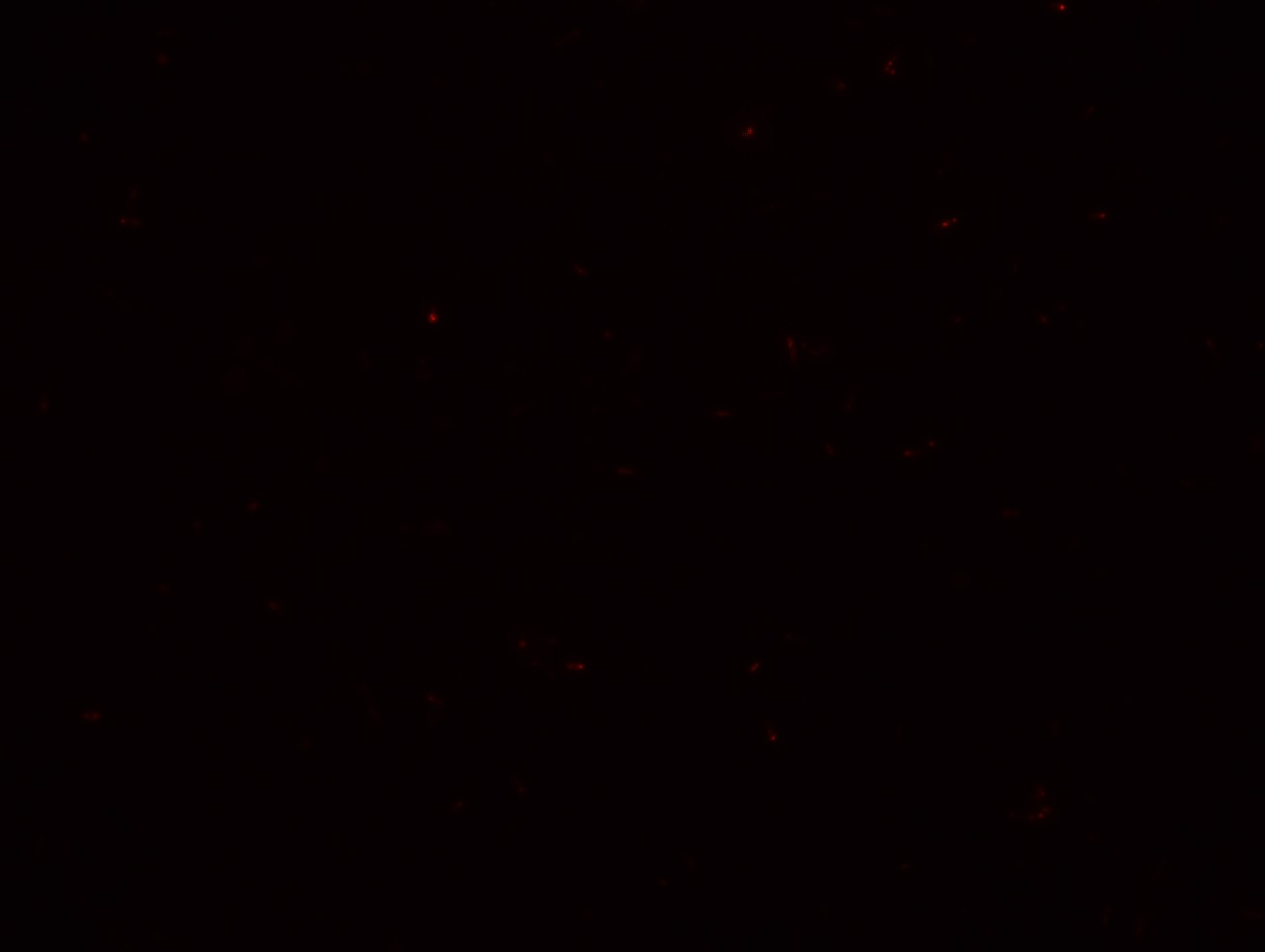

Supplement: File SI2 — Microscopy images of Halomonas sp. CUBES01. [file aem.00603-24-s0002.zip › Microscopy/Sucrose_1st_0003.jpg]
