# Supplementary material for: Isolation and characterization of a Halomonas species for non-axenic growth-associated production of bio-polyesters from sustainable feedstocks
Source: Appl Environ Microbiol. 2024 Jul 26;90(8):e00603-24. doi: 10.1128/aem.00603-24 (PMC11338360; doi:10.1128/aem.00603-24)
Supplement: Supplemental material — Tables S1 to S9; Fig. S1 to S9. [file aem.00603-24-s0003.pdf]

AMERICAN SOCIETY FOR MICROBIOLOGY
Journals.ASM.org

SUPPORTING INFORMATION: Isolation and Characterization of a *Halomonas* Species for Non-Axenic Growth-Associated Production of Bio-Polyesters from Sustainable Feedstocks

Sung-Geun Woo[1,2,*], Nils J. H. Averesch[1,2,*], Aaron J. Berliner[1,3], Joerg S. Deutzmann[2], Vince E. Pane[1,4], Sulogna Chatterjee[1,2], Craig S. Criddle[1,2]

[1]Center for the Utilization of Biological Engineering in Space (CUBES), Berkeley, California 94720, United States

[2]Department of Civil and Environmental Engineering, Stanford University, Stanford, California 94305, United States

[3]Department of Bioengineering, University of California Berkeley, Berkeley, California 94704, United States

[4]Department of Chemistry, Stanford University, Stanford, California 94305, United States

[*]SGW: wsg135@stanford.edu, NJHA: nils.averesch@stanford.edu

First and Second Author are Co-Senior Authors.

SUPPLEMENTARY TABLES

**Fatty acid**	*Halomonas* sp. CUBES01	*H. gomseomensis* M12^T	*H. janggokensis* M24^T	*H. venusta* DSM 4743^T	*H. lutesence* Q1U^T	*H. meridiana* DSM 5425^T	*H. lysinitropha* 3(2)^T	*Halomonas* sp. NA10-65
$C_{10:0}$	2.1	2.0	2.1	2.2	N/A	N/A	4.8	N/A
$C_{12:0}$	N/A	N/A	N/A	1.2	N/A	1.0	N/A	N/A
$C_{12:0}$ 3-OH	5.7	10.0	8.3	6.6	5.4	6.3	13.5	N/A
$C_{14:0}$	N/A	N/A	N/A	2.6	4.2	3.3	N/A	N/A
$C_{16:1}$ ω7c*	11.7	9.7	7.6	11.5	14.8	10.4	16.7	17.3
$C_{16:0}$	15.6	16.5	12.4	14.0	10.1	14.8	29.0	29.9
$C_{17:0}$ cyclo	N/A	N/A	N/A	N/A	N/A	N/A	3.8	N/A
$C_{18:1}$ ω7c	54.9	57.4	61.8	61.9	58.6	60.5	10.3	28.2
$C_{18:0}$	N/A	N/A	N/A	N/A	1.2	N/A	N/A	N/A
$C_{19:0}$ cyclo ω8c	6.8	3.6	6.3	N/A	N/A	2.2	20.5	N/A

TABLE 1 Fatty acid composition of *Halomonas* sp. CUBES01 and related species. Data for *H. gomseomensis* M12^T, *H. janggokensis* M24^T, and *H. venusta* DSM 4743^T were obtained from Kim *et al.*, 2007 [1], for *H. lutesence* Q1U^T and *H. meridiana* DSM 5425^T from Wang *et al.*, 2016 [2], for *H. lysinitropha* 3(2)^T from Ramezani *et al.*, 2020 [3], and for *Halomonas* sp. NA 10-65 from Baxter & Butler, 2020 [4]. The fatty acid profile of CUBES01 was determined by DSMZ as a service (Analysis of Cellular Fatty Acids). Values are percentages of total fatty acids; N/A indicates not detected or below 1%. * As per the Microbial Identification System the fatty acids C16:1 ω7c and C16:1 ω6c are described as non-separable and are therefore commonly lumped into a summed feature. However, in the here characterized *Halomonas* sp. only C16:1 ω7c was identified.

**Characteristic**	*Halomonas* sp. CUBES01	*H. lutesence* Q1U^T	*H. venusta* DSM 4743^T	*H. meridiana* DSM 5425^T
Oxidase Activity	+	+	+	+
Reduction of Nitrate	-	+	+	-
Hydrolysis of Gelatin	-	-	-	-
**Substrate:**				
L-Arabinose	+	-	-	-
D-Cellobiose	-	+	+	-
D-Fructose	+	-	-	-
D-Galactose	+	-	+	+
D-Glucose	+	+	-	+
Lactose	-	-	-	-
Maltose	+	-	-	+
D-Mannose	-	+	+	-
D-Mannitol	+	+	+	+
D-Raffinose	-	-	-	-
D-Rhamnose	-	+	-	-
Ribose	-	-	+	-
Sorbitol	-	-	+	-
Sucrose	+	+	+	+
D-Trehalose	+	+	+	+
Xylose	-	-	-	-
Glycerol	+	+	-	+

TABLE 2 Analytical Profile Index (API®) tests 20NE and 50CHB performed on *Halomonas* sp. CUBES01 in comparison to related species. Data for *H. lutesence* Q1U^T, *H. venusta* DSM 4743^T, and *H. meridiana* DSM 5425^T were obtained from Wang *et al.*, 2016 [2]. Phenotypes of CUBES01 were determined by DSMZ as a service when grown on M1 medium with 10% sodium chloride at 28°C for 48 hours.

Antibiotic	Sensitivity
Ampicillin	++
Oxacillin	++
Penicillin G	++
Aztreonam	++
Ticarcillin	++
Cefotaxime	++
Ceftazidime	++
Ceftriaxone	++
Cefiderocol	++
Piperacillin/Tazobactam	++
Imipenem	++
Meropenem	++
Ciprofloxacin	++
Levofloxacin	++
Moxifloxacin	++
Ofloxacin	++
Amikacin	+
Gentamicin	++
Tetracycline	++
Tigecycline	++
Teicoplanin	-
Vancomycin	+
Polymyxin B	++
Colistin sulphate	++
Clindamycin	++
Erythromycin	++
Fosfomycin	+
Kanamycin	+
Chloramphenicol	++

Linezolid	++
Nitrofurantoin	++
Quinupristin/Dalfopristin	++
Rifampicin	++
Trimethoprim-sulfamethoxazole (1:19)	++
Ampicillin	++
Oxacillin	++
Penicillin G	++
Aztreonam	++
Ticarcillin	++

TABLE 3: **Susceptibility of *Halomonas* sp. CUBES01 to different antibiotics.** Phenotypes of CUBES01 were determined by DSMZ as a service when grown on M1 medium with 10% sodium chloride at 30°C for 48 hours. The diameter of inhibition zone is interpreted into no inhibition (-), 0.1-1.6 cm (+), and > 1.6 cm (++), respectively.

**Antibiotic**	**Inhibition**
Chloramphenicol	yes
Neomycin	yes
Streptomycin	yes
Spectinomycin	yes

TABLE 4 **Susceptibility of *Halomonas* sp. CUBES01 to antibiotics not screened previously.** Streaks of cells were grown overnight at 30°C on Nutrient Broth with 4% sodium chloride; the respective antibiotic were added in concentrations common for bacterial cultures (Chloramphenicol: 50 μg/mL, Neomycin: 50 μg/mL, Streptomycin: 100 μg/mL, Spectinomycin: 50 μg/mL). Inhibition of growth was identified qualitatively as yes/no in comparison to a no-antibiotic positive control.

21

Plasmid	Size (Kbp)	Characteristics
pTJS140 [5]	9.34	RK2/RP4 oriV, Stm^R / Spm^R
pBBR1MCS [6]	5.93	pBBR1 oriV, Kan^R / Neo^R
pCM66T [7]	7.34	RK2/RP4 oriV, Kan^R / Neo^R

TABLE 5 Plasmid-vectors used in the present study. oriV: origin of vegetative replication, Stm^R: streptomycin resistance, Spm^R: spectinomycin resistance, Kan^R: kanamycin resistance, Neo^R: neomycin resistance

22

Primer	Sequence (5′-3′)	Target
8 forward [8]	AGAGTTTGATCCTGGCTCAG	Bacterial 16S rRNA
1492 reverse [8]	GGTTACCTTGTTACGACT	Bacterial 16S rRNA
araC forward	GCTCAAGCAGATTTATCGCCAGCAGC	pBBR1MCS / pTJS140
araC reverse	GCTGCTGGCGATAAATCTGCTTGAGC	pBBR1MCS / pTJS140
spc forward	GCTATCTTGCTGACAAAAGCAAGAGAACATAGC	pTJS140
spc reverse	GCTATGTTCTCTTGCTTTTGTCAGCAAGATAGC	pTJS140
neoR forward	GCGATAGAAGGCGATGCGCTGC	pBBR1MCS
neoR reverse	GCAGCGCATCGCCTTCTATCGC	pBBR1MCS
kanR forward	GCATCCATGTTGGAATTTAATCGC	pCM66T
kanR reverse	GCGATTAAATTCCAACATGGATGC	pCM66T

TABLE 6 Primers used in the present study for amplification of 16S rRNA and confirmation of self-replicating plasmids in *Halomonas* sp. CUBES01 via PCR.

	Microorganism	GenBank Accession #
1	*Halomonas sp. CUBES01*	OQ359097.1
2	*Halomonas gomseomensis M12*	NR_042488.1
3	*Halomonas arcis AJ282*	NR_044115.1
4	*Halomonas janggokensis M24*	NR_042489.1
5	*Halomonas maris QX-1*	MT372903.1
6	*Halomonas populi MC*	MK045667.1
7	*Halomonas azerica TBZ9*	MN900573.1
8	*Halomonas subterranea ZG16*	NR_044116.1
9	*Halomonas zhaodongensis NEAU-ST10-25*	NR_125612.1
10	*Halomonas sedimenti QX-2*	MT372904.1
11	*Halomonas songnenensis NEAU-ST10-39*	NR_126242.1
12	*Halomonas hydrothermalis Slthf2*	NR_027220.1
13	*Halomonas venusta DSM 4743*	NR_042069.1
14	*Halomonas johnsoniae T68687*	NR_115090.1
15	*Halomonas titanicae BH1*	NR_117300.1
16	*Halomonas alkaliphila 18bAG*	NR_042256.1
17	*Halomonas axialensis Althf1*	NR_027219.1
18	*Halomonas meridiana DSM 5425*	NR_042066.1
19	*Halomonas aquamarina DSM 30161*	NR_042063.1
20	*Halomonas lutescens Q1U*	NR_152713.1
21	*Halomonas lionensis RHS90*	OM070354.1
22	*Halomonas hamiltonii W1025*	NR_115089.1
23	*Halomonas andesensis LC6*	NR_116073.1
24	*Halomonas sulfidaeris ATCC BAA-803*	AP019514.1
25	*Halomonas piezotolerans NBT06E8*	MN435603.1
26	*Halomonas nanhaiensis YIM M 13059*	NR_118488.1
27	*Halomonas glaciei DD 39*	NR_114866.1
28	*Halomonas massiliensis Marseille-P2426*	NR_169359.1
29	*Halomonas malpeensis YU-PRIM-29*	NR_163650.1

	Microorganism	GenBank Accession #
30	*Halomonas stevensii S18214*	NR_115088.1
31	*Halomonas vilamensis SV325*	NR_116358.1
32	*Halomonas boliviensis LC1*	NR_029080.1
33	*Halomonas alkaliantarctica CRSS*	NR_114902.1
34	*Halomonas neptunia Eplume1*	NR_027218.1
35	*Halomonas salicampi BH103*	NR_145910.1
36	*Halomonas profundus AT1214*	NR_114956.1
37	*Halomonas zhanjiangensis JSM 78169*	NR_104283.1
38	*Halomonas magadiensis 21M1*	NR_044880.1
39	*Halomonas olivaria TYRC17*	DQ645593.1
40	*Halomonas rituensis TQ8S*	MH071181.2
41	*Halomonas montanilacus PYC7W*	NR_174257.1
42	*Halomonas sediminis YIM*	NR_116180.1
43	*Halomonas urumqiensis BZ-SZ-Xj27*	NR_149235.1
44	*Halomonas zhuhanensis ZH2S*	MH071182.2
45	*Halomonas subglaciescola ACAM 12*	LT670847.1
46	*Halomonas profundi MT13*	CP077941.1
47	*Halomonas boliviensis LC2*	AY245450.1
48	*Halomonas lysinitropha 3(2)*	KU886576.1
49	*Halomonas sp. TD01*	JF340230.1
50	*Halomonas utahensis DSM 3051*	NR_042068.1
51	*Zymobacter palmae T109*	D14555.1

TABLE 7: ***Halomonas* species and Genbank IDs** used in phylogenetic comparison.

	Substrate	Sample #	OD600	CDW [g]	Volume [mL]	Biomass [g/L]	Factor	Average	Deviation
	Nutrient Broth	1	1.6625	0.0878	100	0.88	0.53		
	(3×)	2	3.75	0.14295	50	2.86	0.76	0.7	0.15
		3	4.35	0.1058	30	3.53	0.81		
	Glucose	1	2.25	0.091475	100	0.91	0.41		
		2	4.38	0.1186	50	2.37	0.54	0.52	0.10
		3	6.36	0.11475	30	3.83	0.60		
	Sucrose	1	1.206	0.061325	100	0.61	0.51		
		2	4.83	0.13315	50	2.66	0.55	0.53	0.02
		3	6.825	0.11045	30	3.68	0.54		
	Acetate	1	1.335	0.076575	100	0.77	0.57		
		2	4.01	0.1168	50	2.34	0.58	0.6	0.03
		3	4.81	0.0918	30	3.06	0.64		
	Glycerol	1	1.515	0.0692	100	0.69	0.46		
		2	4.19	0.1367	50	2.73	0.65	0.55	0.10
23		3	5.07	0.08275	30	2.76	0.54		
	Fructose	1	1.21	0.060325	100	0.60	0.50		
		2	1.96	0.0734	50	1.47	0.75	0.67	0.15
		3	2.51	0.05715	30	1.91	0.76		
	Glucosamine	1	1.17	0.0571	100	0.57	0.49		
		2	5.46	0.14625	50	2.93	0.54	0.51	0.02
		3	5.85	0.08895	30	2.97	0.51		
	Acetyl-	1	1.528	0.071075	100	0.71	0.47		
	Glucosamine	2	3.3	0.12015	50	2.40	0.73	0.61	0.13
		3	4.74	0.0919	30	3.06	0.65		
	Propionate	1	1.052	0.05325	100	0.53	0.51		
		2	3.42	0.10705	50	2.14	0.63	0.6	0.08
		3	3.78	0.07535	30	2.51	0.66		
	Average of 1st samples							0.49	0.05
	Average of 2nd samples							0.64	0.09
	Average of 3rd samples							0.63	0.10
	Average of all samples							0.59	0.07

TABLE 8 Correlation of biomass concentration (in optical density) to cell dry weight.

**Phenotype**	**Specific Value**
Salinity range (optimum)	~ 40 to 100 g/L (58 g/L) sodium chloride
pH range (optimum)	~ 7.2 to 9.8 (8.8)
Optimum Cultivation Temperature	30°C
Cell Shape and Dimensions	Rod, 1 to 4 μm (long) and 0.8 to 1 μm (wide)

**Substrate**	**Maximum growth rate** $[\mathbf{h^{-1}}]$	**Maximum per-biomass PHB-yield** **[%]** $\mathbf{g_{PHB}/g_{biomass}}$	**Maximum PHB production-rate** $[\mathbf{g_{PHB}/g_{biomass} \times h^{-1}}]$
Nutrient Broth	0.25±0.021	3±1	0.007±0.003
Sucrose	0.17±0.009	45±14	0.077±0.034
Glucose	0.15±0.007	32±6	0.029±0.008
Fructose	0.09±0.004	35±2	0.052±0.004
Glycerol	0.10±0.005	59±1	0.059±0.002
Propionate	0.07±0.003	18±1	0.013±0.001
Acetate	0.11±0.008	59±2	0.065±0.004
Glucosamine	0.08±0.004	8±1	0.007±0.001
Acetyl-Glucosamine	0.08±0.002	18±3	0.015±0.004

TABLE 9 Select phenotypical properties of *Halomonas* sp. CUBES01. Values for salinity, pH, and temperature were obtained when cultivating the strain on Nutrient Broth (with the respective other parameters optimized). Specific growth rates, as well as PHB production yields and -rates, were obtained when cultivating CUBES01 on chemically-defined (minimal) medium, complemented with the indicated substrate.

SUPPLEMENTARY FIGURES

Codon	AA	Contig 1	Contig 2	Δ
TTT	F	46.7	43.7	3.0
TTC	F	**53.4**	**56.3**	3.0
TTA	L	7.8	9.3	1.5
TTG	L	19.4	19.5	0.1
CTT	L	17.8	21.4	3.6
CTC	L	16.3	12.1	4.2
CTA	L	7.6	10.7	3.1
CTG	L	**31.1**	**27.0**	4.1
ATT	I	27.1	25.1	2.0
ATC	I	**49.9**	**46.8**	3.0
ATA	I	23.1	28.1	5.0
ATG	M	**100.0**	**100.0**	0.0
GTT	V	22.9	24.7	1.8
GTC	V	29.8	28.4	1.5
GTA	V	16.1	15.9	0.2
GTG	V	**31.2**	**31.1**	0.2
TCT	S	9.9	9.0	0.9
TCC	S	12.6	14.2	1.6
TCA	S	18.3	18.2	0.1
TCG	S	24.5	24.6	0.2
AGT	S	8.7	7.4	1.2
AGC	S	**26.1**	**26.6**	0.5
CCT	P	18.7	18.9	0.2
CCC	P	21.9	22.2	0.4
CCA	P	28.1	**31.1**	3.0
CCG	P	**31.4**	27.8	3.6
ACT	T	14.8	12.9	1.9
ACC	T	**34.8**	**35.9**	1.1
ACA	T	17.7	19.5	1.8
ACG	T	32.8	31.7	1.1

Codon	AA	Contig 1	Contig 2	Δ
GCT	A	20.5	22.6	2.0
GCC	A	30.2	**28.1**	2.1
GCA	A	18.8	23.6	4.8
GCG	A	**30.5**	25.7	4.7
TAT	Y	48.0	46.5	1.5
TAC	Y	**52.0**	**53.5**	1.5
TAA	*	21.2	18.9	2.4
TAG	*	20.4	21.5	1.1
TGA	*	**58.4**	**59.7**	1.3
CAT	H	49.3	**51.0**	1.7
CAC	H	**50.7**	49.0	1.7
CAA	Q	39.2	**56.0**	16.8
CAG	Q	**60.8**	44.1	16.8
AAT	N	42.1	44.9	2.8
AAC	N	**57.9**	**55.1**	2.8
AAA	K	46.3	39.8	6.5
AAG	K	**53.8**	**60.2**	6.5
GAT	D	**52.1**	**53.9**	1.8
GAC	D	47.9	46.1	1.8
GAA	E	**53.5**	**54.3**	0.8
GAG	E	46.5	45.7	0.8
TGT	C	30.3	30.1	0.2
TGC	C	**69.7**	**69.9**	0.2
TGG	W	**100.0**	**100.0**	0.0
CGT	R	14.4	15.5	1.2
CGC	R	**27.9**	**26.9**	0.9
CGA	R	17.8	20.0	2.2
CGG	R	20.5	20.7	0.2
AGA	R	7.1	7.0	0.1
AGG	R	12.4	9.9	2.5
GGT	G	22.5	19.6	2.9
GGC	G	**41.6**	**42.6**	1.0
GGA	G	14.4	16.7	2.3
GGG	G	21.6	21.2	0.4

FIG 1 Codon Usage Bias (CUB) of *Halomonas* sp. CUBES01, based on the annotated open-reading frames differentiated by contig 1 and contig 2.
The Δ is the absolute percentage difference between the two individual contigs.

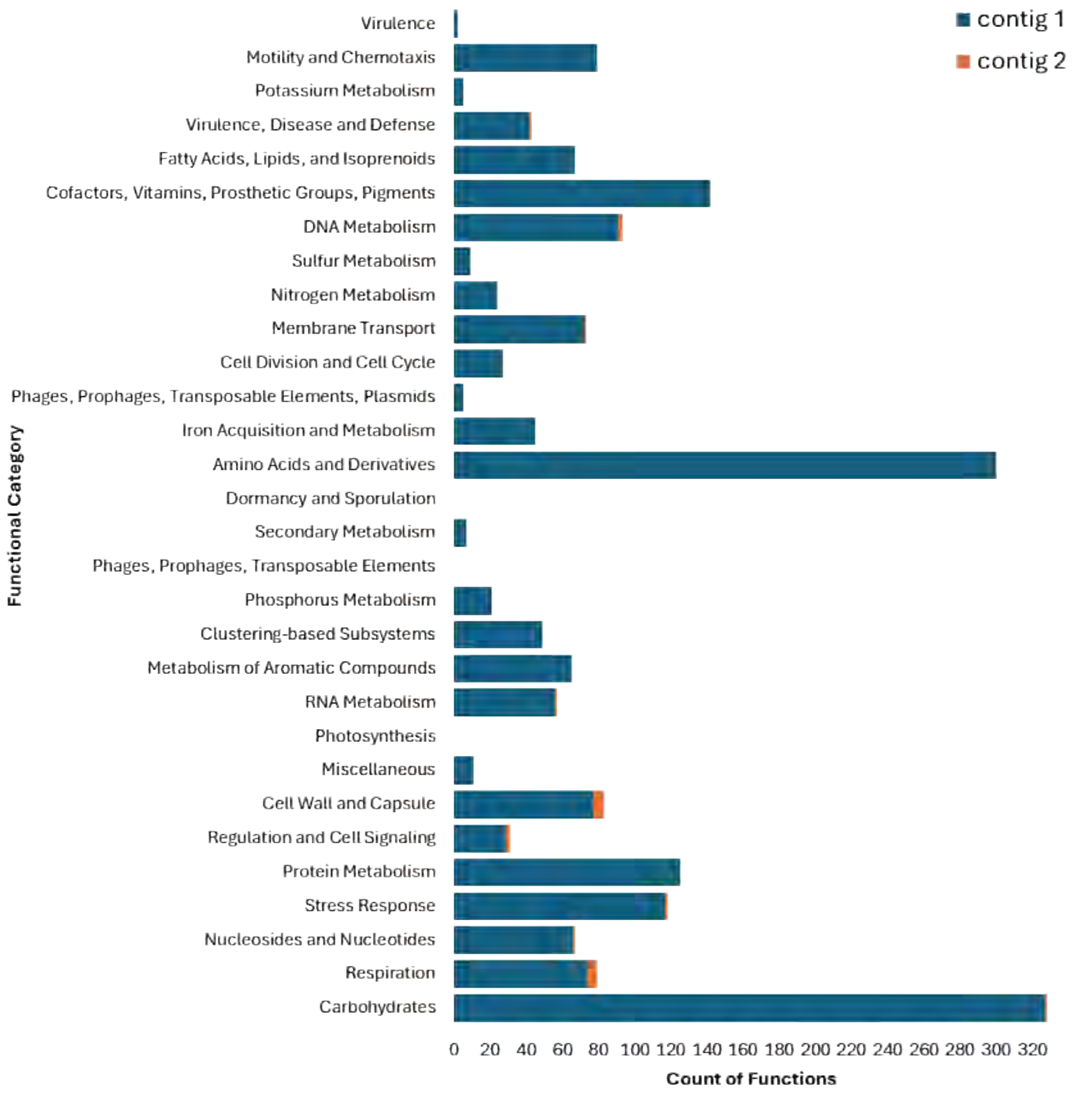

FIG 2 SEED functions identified for the sequenced genome of CUBES01 as annotated for contig 1 and contig 2 with RASTtk v1.073 [9] using default parameters for the bacteria domain (B) with KBase [10], distinguished by functional category. The total feature count was 1934 and 21, respectively.

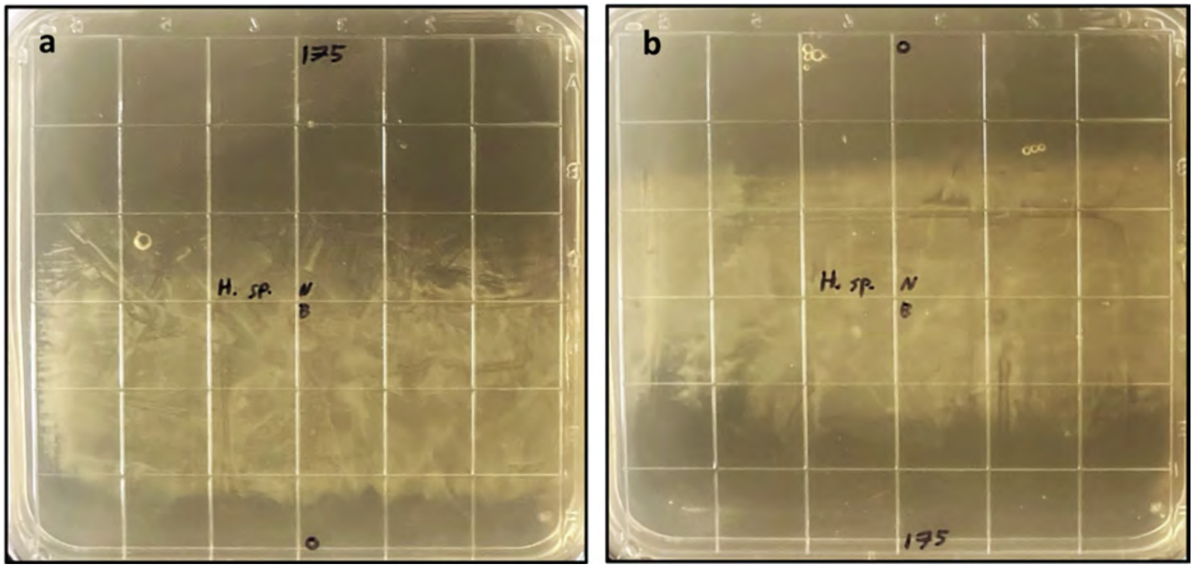

FIG 3 Gradient-agar plates (Nutrient Broth) showing growth of *Halomonas* sp. CUBES01 over a sodium chloride concentration ranging from 175 g/L (top) to 0 g/L (bottom) in **(a)** and 0 g/L (top) to 175 g/L (bottom) in **(b)**.

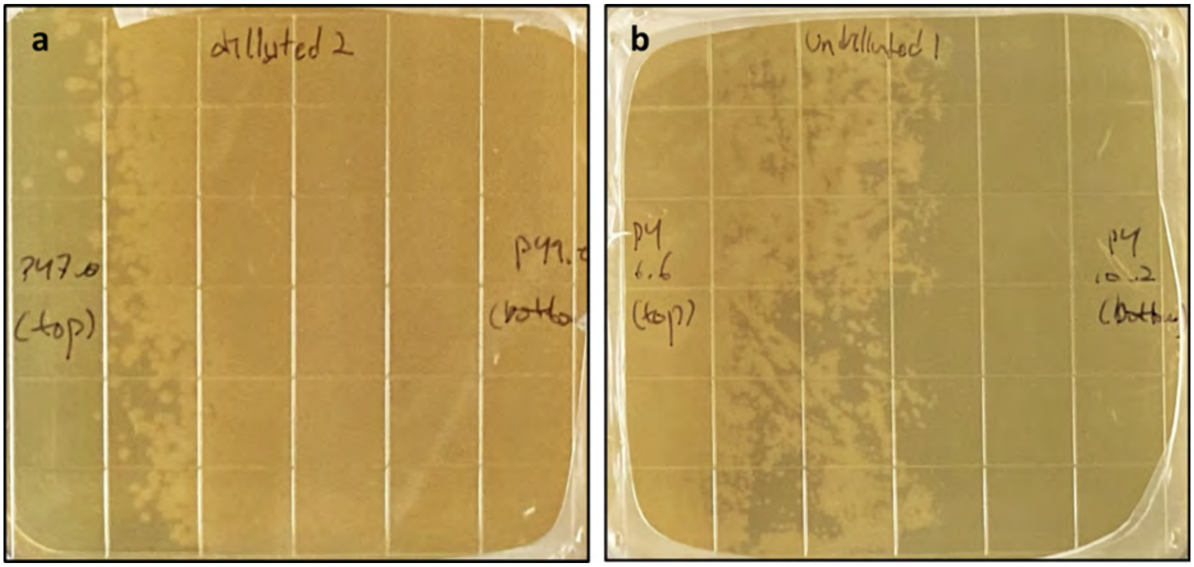

FIG 4 Gradient-agar plates (Nutrient Broth with 1 M sodium chloride) showing the lower **(a)** and upper limiting pH **(b)** for growth of *Halomonas* sp. CUBES01. While in **(a)** a horizontal pH gradient from approx. 7.0 (left) to 9.0 (right) was achieved, **(b)** has a pH ranging from approx. 6.6 (left) to 10.2 (right).

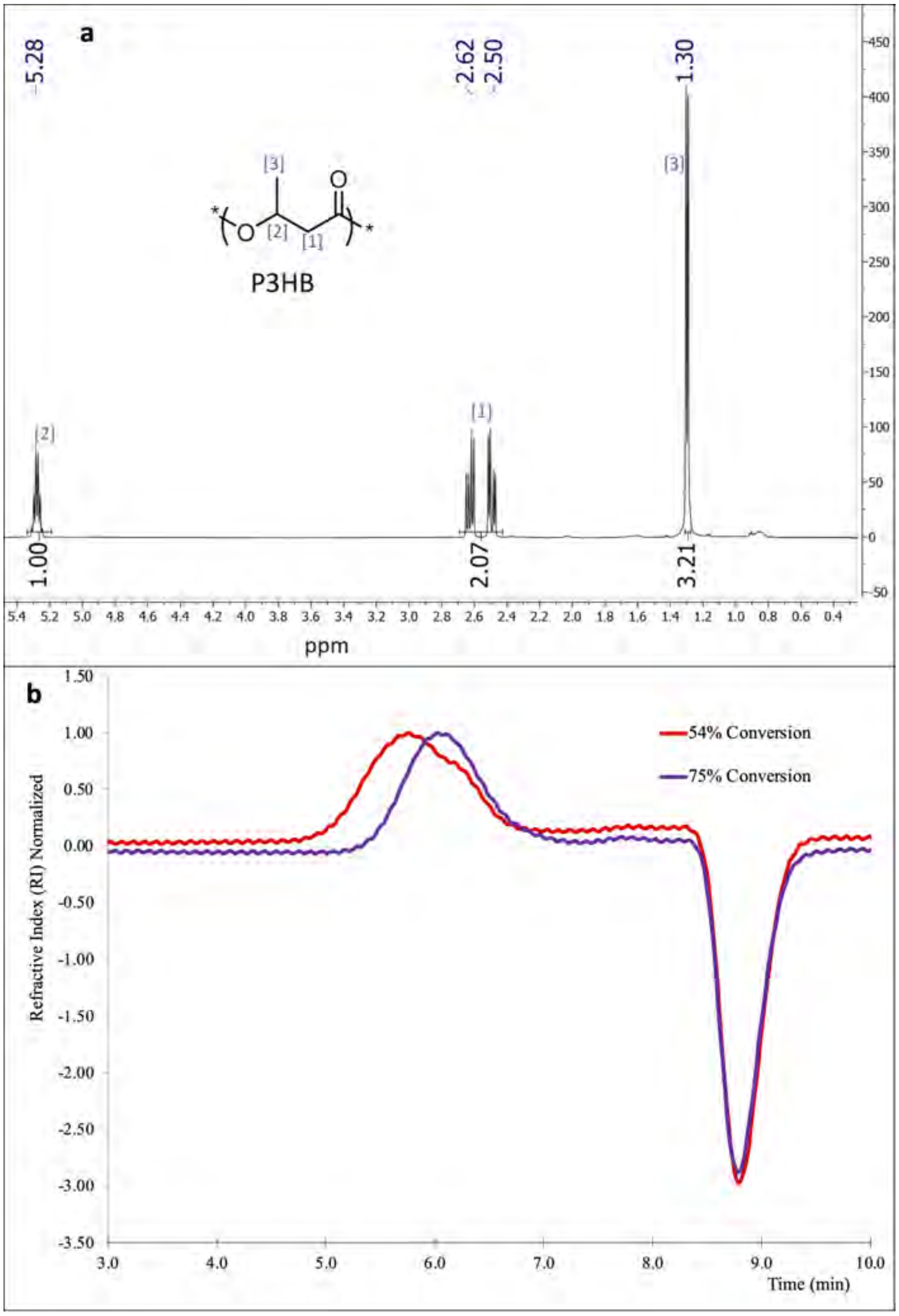

FIG 5 NMR and GPC analyses of polyester obtained from *Halomonas* sp. CUBES01 when grown on a semi-minimal medium (modified MM-G as per Chen *et al.*, 2017 [11]) with acetate as the primary substrate. **(a)** Nuclear Magnetic Resonance spectrum for protons (^{1}H-NMR) of the obtained bio-polyester. The poly(hydroxyalkanoate) was composed of 3-hydroxybutyrate repeat-units, according to the sextet resonance at a chemical shift of 5.25 ppm.
(b) Elugram of Gel-Permeation Chromatography with the bio-polyester.

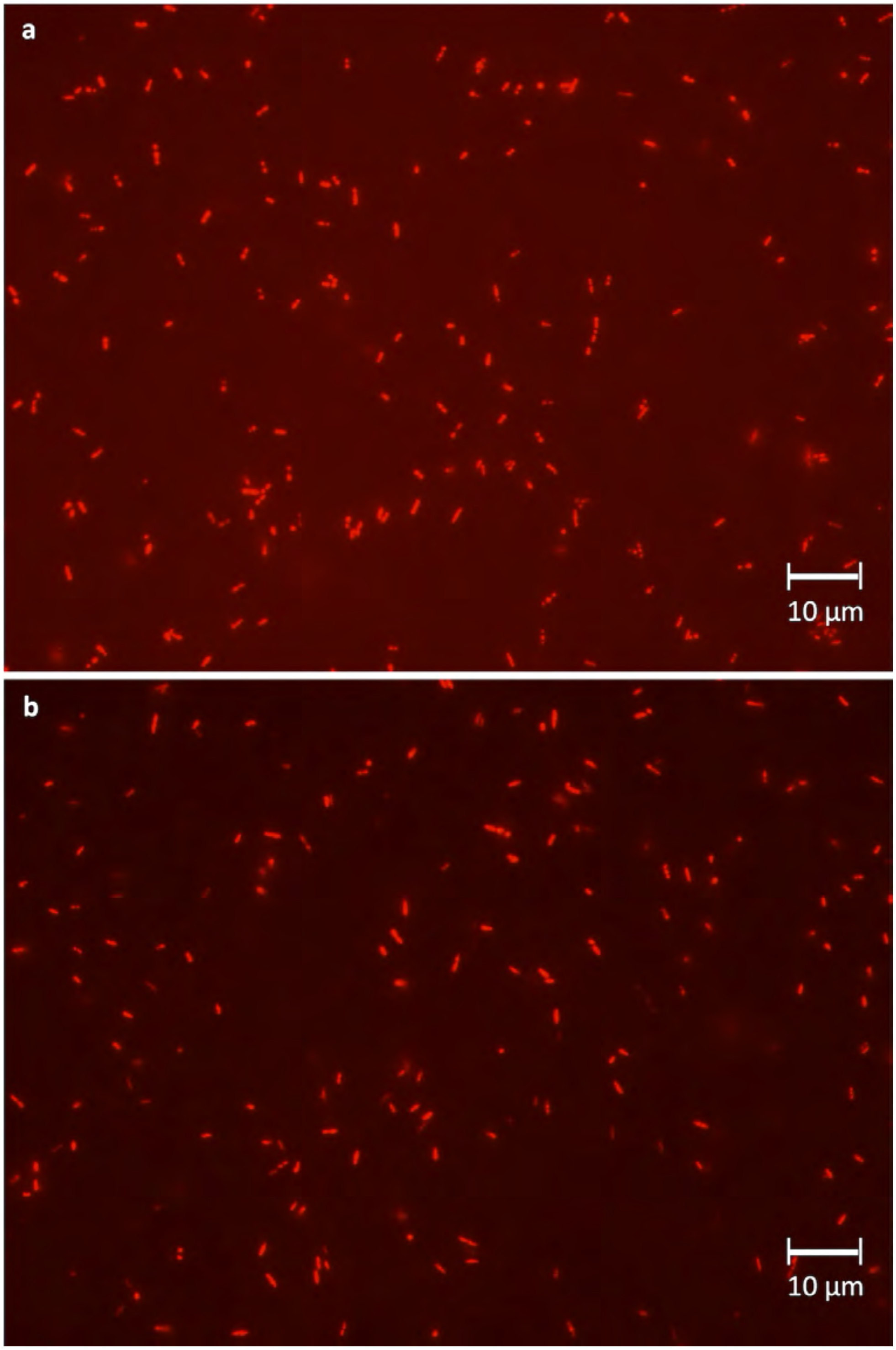

FIG 6 Microscopy of *Halomonas* sp. CUBES01 cells stained with Nile red. The samples were obtained from late exponential growth-phase, during cultivation on chemically-defined medium with sucrose **(a)** and glycerol **(b)**, corresponding to FIG 4. Additional microscopy images are provided in SI3.

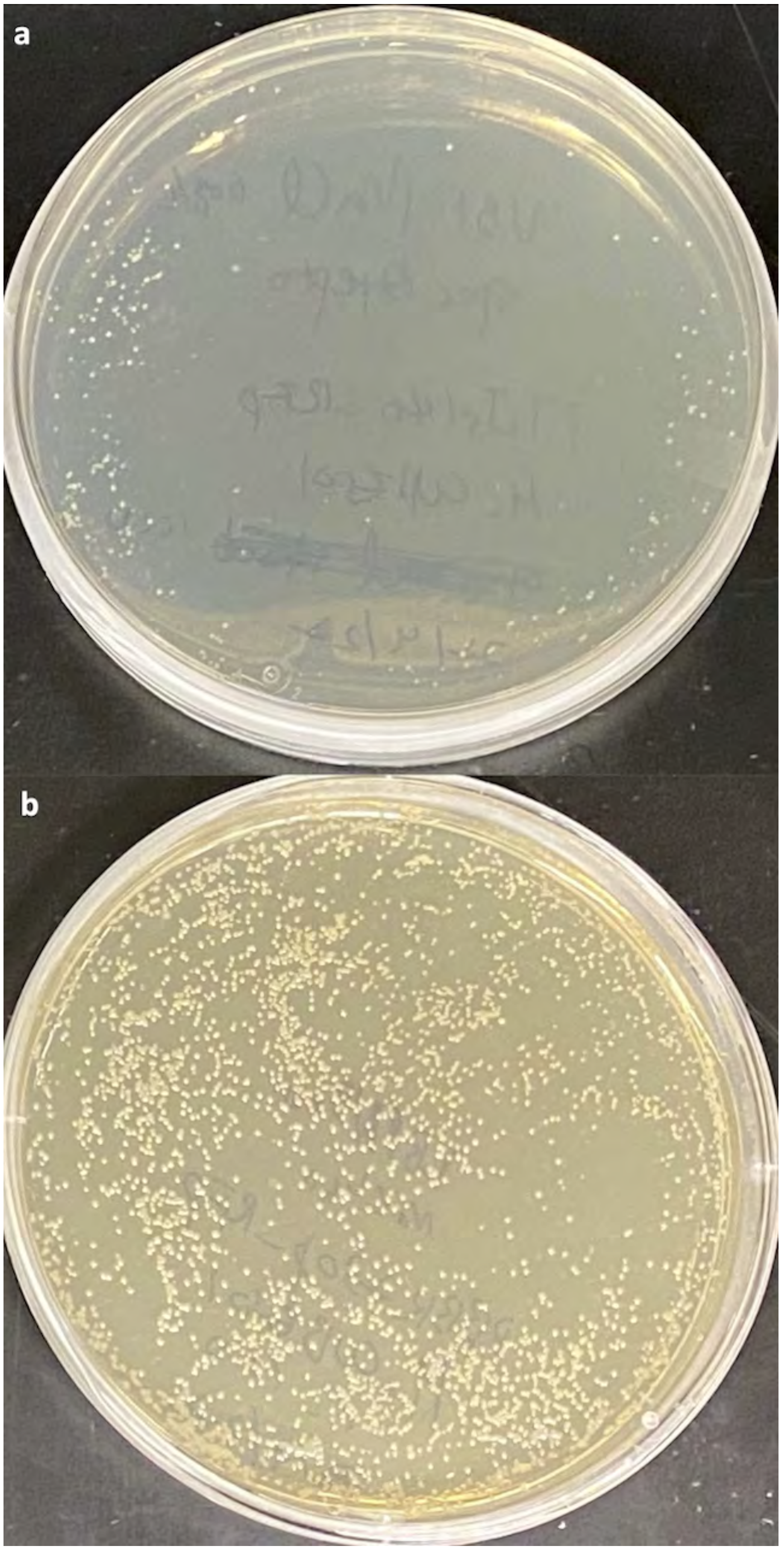

FIG 7 Transconjugants of *Halomonas* sp. CUBES01. Colonies of transformed cells carrying **(a)** pTJS140 selected on the Strep^R/Spec^R plate (1:10-diluted); **(b)** pBBR1MCS selected on the Kan^R/Neo^R plate (1:100-diluted).

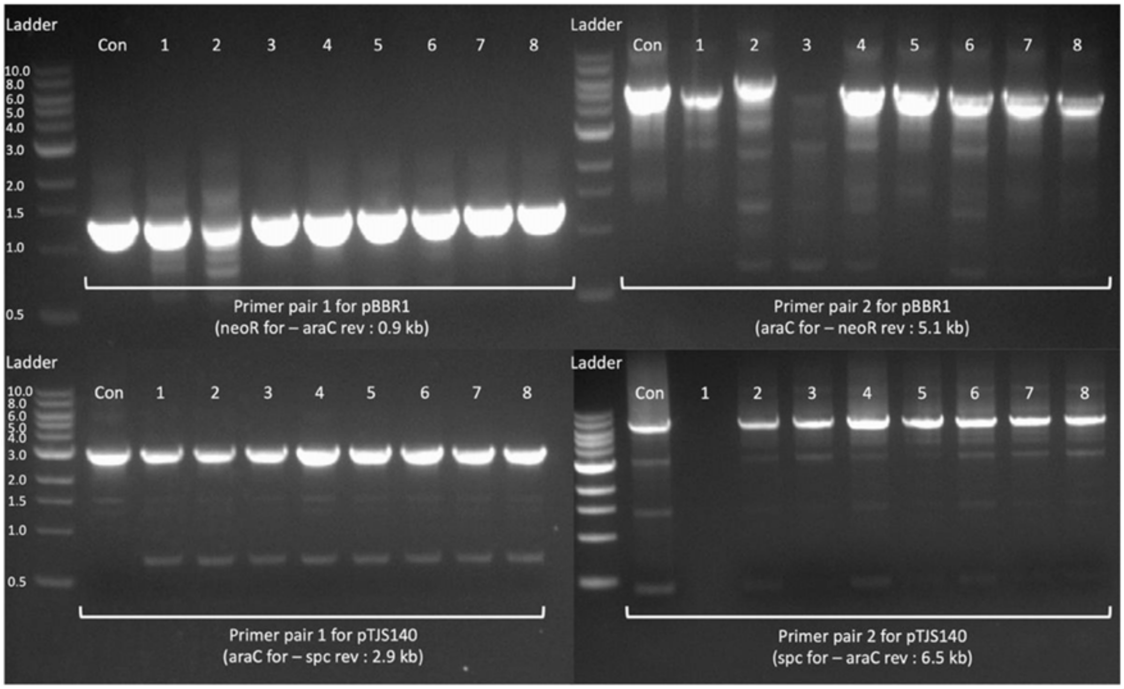

FIG 8 Gel Electrophoresis of amplicons from confirmation PCR of *Halomonas* sp. CUBES01 mutants bearing the plasmids pBBR1MCS (top) or pTJS140 (bottom). The expected sizes of amplicons were 0.9 kbp and 5.1 kbp for pBBR1MS, and 2.9 kbp and 6.5 kbp for pTJS140. Combinations of primers correspond to full sequence coverage of each plasmid, confirming that the vectors were, in fact, maintained and intact.

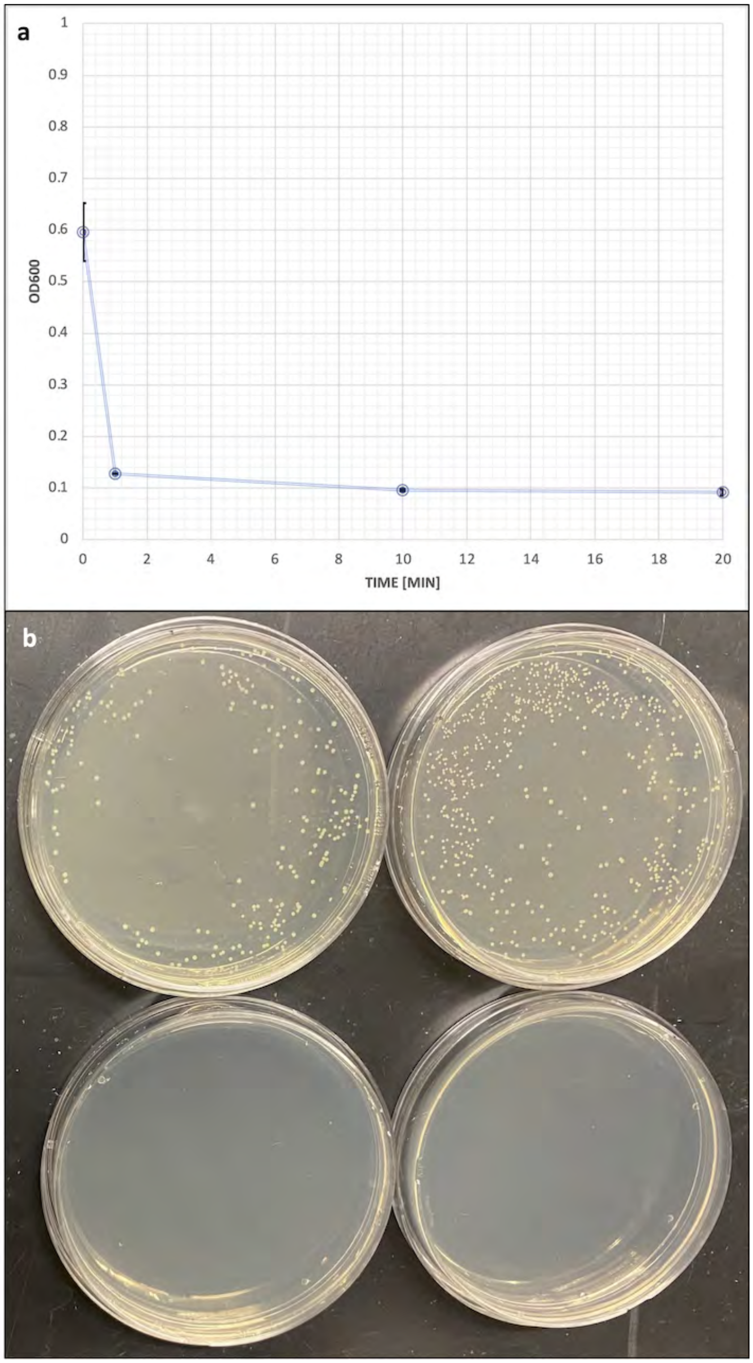

FIG 9 Osmolysis test with *Halomonas* sp. CUBES01. **(a)** Change of optical density (OD600) over time during osmolysis of *Halomonas* cells in salt-free water. **(b)** Cell viability test (10^4-fold dilution) on solid growth medium (Nutrient Broth with 1 M sodium chloride) by colony formation as a measure of osmolysis. Top row are agar-plates containing samples from the original culture (OD600 of 0.6), as opposed to the bottom row containing samples after exposure of the cells to deionized water for 1 minute.

References

[1] Kwang Kyu Kim, Long Jin, Hee Chan Yang, and Sung-Taik Lee. Halomonas gomseomensis sp. nov., Halomonas janggokensis sp. nov., Halomonas salaria sp. nov. and Halomonas denitrificans sp. nov., moderately halophilic bacteria isolated from saline water. *International Journal of Systematic and Evolutionary Microbiology*, 57(4): 675–681, April 2007. ISSN 1466-5026, 1466-5034. doi: 10.1099/ijs.0.64767-0. URL https://www.microbiologyresearch.org/content/journal/ijsem/10.1099/ijs.0.64767-0.

[2] Tianying Wang, Xuexin Wei, Yuhua Xin, Junli Zhuang, Shuangquan Shan, and Jianli Zhang. Halomonas lutescens sp. nov., a halophilic bacterium isolated from a lake sediment. *International Journal of Systematic and Evolutionary Microbiology*, 66(11): 4697–4704, November 2016. ISSN 1466-5026, 1466-5034. doi: 10.1099/ijsem.0.001413. URL https://www.microbiologyresearch.org/content/journal/ijsem/10.1099/ijsem.0.001413.

[3] Mohaddaseh Ramezani, Mohammad Pourmohyadini, Mahdi Moshtaghi Nikou, Somaye Makzum, Peter Schumann, Dominique Clermont, Alexis Criscuolo, Mohammad Ali Amoozegar, Peter Kämpfer, and Cathrin Spröer. Halomonas lysinitropha sp. nov., a novel halophilic bacterium isolated from a hypersaline wetland. *International Journal of Systematic and Evolutionary Microbiology*, 70(12): 6098–6105, December 2020. ISSN 1466-5026, 1466-5034. doi: 10.1099/ijsem.0.004504. URL https://www.microbiologyresearch.org/content/journal/ijsem/10.1099/ijsem.0.004504.

[4] Bonnie K. Baxter and Jaimi K. Butler, editors. *Great Salt Lake Biology: A Terminal Lake in a Time of Change*. Springer International Publishing, Cham, 2020. ISBN 978-3-030-40351-5 978-3-030-40352-2. doi: 10.1007/978-3-030-40352-2. URL http://link.springer.com/10.1007/978-3-030-40352-2.

[5] Thomas J. Smith, Susan E. Slade, Nicolas P. Burton, J. Colin Murrell, and Howard Dalton. Improved System for Protein Engineering of the Hydroxylase Component of Soluble Methane Monooxygenase. *Applied and Environmental Microbiology*, 68(11):

5265–5273, November 2002. ISSN 0099-2240, 1098-5336. doi: 10.1128/AEM.68.11.5265-5273.2002. URL https://journals.asm.org/doi/10.1128/AEM.68.11.5265-5273.2002.

[6] Michael E. Kovach, Philip H. Elzer, D. Steven Hill, Gregory T. Robertson, Michael A. Farris, R.Martin Roop, and Kenneth M. Peterson. Four new derivatives of the broad-host-range cloning vector pBBR1MCS, carrying different antibiotic-resistance cassettes. *Gene*, 166(1):175–176, December 1995. ISSN 03781119. doi: 10.1016/0378-1119(95)00584-1. URL https://linkinghub.elsevier.com/retrieve/pii/0378111995005841.

[7] Christopher J Marx and Mary E Lidstrom. Development of improved versatile broad-host-range vectors for use in methylotrophs and other Gram-negative bacteria. *Microbiology*, 147(8):2065–2075, August 2001. ISSN 1350-0872, 1465-2080. doi: 10.1099/00221287-147-8-2065. URL https://www.microbiologyresearch.org/content/journal/micro/10.1099/00221287-147-8-2065.

[8] P. A. Eden, T. M. Schmidt, R. P. Blakemore, and N. R. Pace. Phylogenetic Analysis of Aquaspirillum magnetotacticum Using Polymerase Chain Reaction-Amplified 16S rRNA-Specific DNA. *International Journal of Systematic Bacteriology*, 41(2):324–325, April 1991. ISSN 0020-7713, 1465-2102. doi: 10.1099/00207713-41-2-324. URL https://www.microbiologyresearch.org/content/journal/ijsem/10.1099/00207713-41-2-324.

[9] Thomas Brettin, James J. Davis, Terry Disz, Robert A. Edwards, Svetlana Gerdes, Gary J. Olsen, Robert Olson, Ross Overbeek, Bruce Parrello, Gordon D. Pusch, Maulik Shukla, James A. Thomason, Rick Stevens, Veronika Vonstein, Alice R. Wattam, and Fangfang Xia. RASTtk: A modular and extensible implementation of the RAST algorithm for building custom annotation pipelines and annotating batches of genomes. *Scientific Reports*, 5(1):8365, February 2015. ISSN 2045-2322. doi: 10.1038/srep08365. URL https://www.nature.com/articles/srep08365.

[10] Adam P Arkin, Robert W Cottingham, Christopher S Henry, Nomi L Harris, Rick L Stevens, Sergei Maslov, Paramvir Dehal, Doreen Ware, Fernando Perez, Shane Canon, Michael W Sneddon, Matthew L Henderson, William J Riehl, Dan Murphy-Olson,

Stephen Y Chan, Roy T Kamimura, Sunita Kumari, Meghan M Drake, Thomas S Brettin, Elizabeth M Glass, Dylan Chivian, Dan Gunter, David J Weston, Benjamin H Allen, Jason Baumohl, Aaron A Best, Ben Bowen, Steven E Brenner, Christopher C Bun, John-Marc Chandonia, Jer-Ming Chia, Ric Colasanti, Neal Conrad, James J Davis, Brian H Davison, Matthew DeJongh, Scott Devoid, Emily Dietrich, Inna Dubchak, Janaka N Edirisinghe, Gang Fang, José P Faria, Paul M Frybarger, Wolfgang Gerlach, Mark Gerstein, Annette Greiner, James Gurtowski, Holly L Haun, Fei He, Rashmi Jain, Marcin P Joachimiak, Kevin P Keegan, Shinnosuke Kondo, Vivek Kumar, Miriam L Land, Folker Meyer, Marissa Mills, Pavel S Novichkov, Taeyun Oh, Gary J Olsen, Robert Olson, Bruce Parrello, Shiran Pasternak, Erik Pearson, Sarah S Poon, Gavin A Price, Srividya Ramakrishnan, Priya Ranjan, Pamela C Ronald, Michael C Schatz, Samuel M D Seaver, Maulik Shukla, Roman A Sutormin, Mustafa H Syed, James Thomason, Nathan L Tintle, Daifeng Wang, Fangfang Xia, Hyunseung Yoo, Shinjae Yoo, and Dantong Yu. KBase: The United States Department of Energy Systems Biology Knowledgebase. *Nature Biotechnology*, 36(7):566–569, August 2018. ISSN 1087-0156, 1546-1696. doi: 10.1038/nbt.4163. URL https://www.nature.com/articles/nbt.4163.

[11] Xiangbin Chen, Jin Yin, Jianwen Ye, Haoqian Zhang, Xuemei Che, Yiming Ma, Mengyi Li, Lin-Ping Wu, and Guo-Qiang Chen. Engineering Halomonas bluephagenesis TD01 for non-sterile production of poly(3-hydroxybutyrate-co-4-hydroxybutyrate). *Bioresource Technology*, 244:534–541, November 2017. ISSN 09608524. doi: 10.1016/j.biortech.2017.07.149. URL https://linkinghub.elsevier.com/retrieve/pii/S0960852417312658.
