# Supplementary material for: Isolation and characterization of a Halomonas species for non-axenic growth-associated production of bio-polyesters from sustainable feedstocks
Source: Appl Environ Microbiol. 2024 Jul 26;90(8):e00603-24. doi: 10.1128/aem.00603-24 (PMC11338360; doi:10.1128/aem.00603-24)

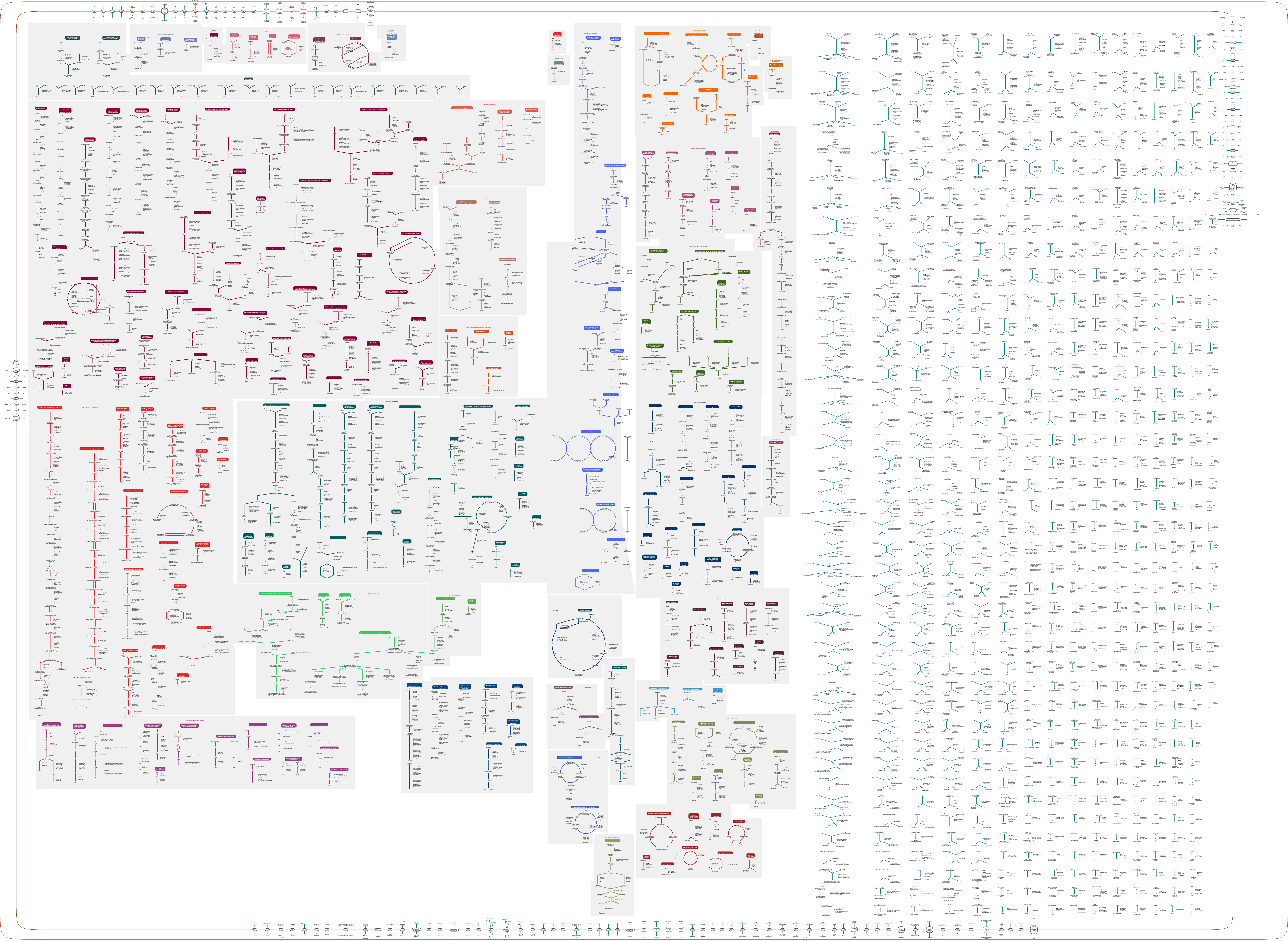

Supplement: File SI1 — Metabolic map of Halomonas sp. CUBES01. [file aem.00603-24-s0001.pdf]
